# A ruthenium(ii)-catalyzed C–H allenylation-based approach to allenoic acids†
†Electronic supplementary information (ESI) available. CCDC 1877103. For ESI and crystallographic data in CIF or other electronic format see DOI: 10.1039/c9sc00603f


**DOI:** 10.1039/c9sc00603f

**Published:** 2019-05-10

**Authors:** Xiaoyan Wu, Junjie Fan, Chunling Fu, Shengming Ma

**Affiliations:** a Laboratory of Molecular Recognition and Synthesis , Department of Chemistry , Zhejiang University , Hangzhou 310027 , Zhejiang , People's Republic of China . Email: masm@sioc.ac.cn

## Abstract

A Ru(ii)-catalyzed direct access to various functionalized allenoic acids *via* C–H allenylation of readily available aryl carboxylic acids with propargylic acetates is reported. Axially chiral allenoic acids could be obtained in high ee by using optically active propargylic acetates through a chirality transfer strategy.

## Abstract 2

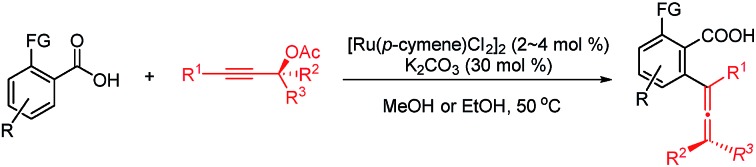
An efficient Ru-catalyzed approach to tetrasubstituted allenes from benzoic acids and propargylic acetates has been developed.

## 

Allene moieties are not only present in natural products but also in precious building blocks due to their unique structures and multiple reactive sites.^1^ Allene chemistry has experienced an explosion during the last few decades.^2^ Thus, the synthesis of functionalized allenes is of crucial importance. Of particular interest is the synthesis of synthetically versatile allenoic acids.^3^ A common approach to 2,3-allenoic acids is the hydrolysis of 2,3-allenoates, which suffers from poor step-economy and a selectivity issue of forming allenoic acids and 3-alkynoic acids (Scheme 1a).^4^ The only example of the oxidation of allenols is realized through microbial transformation (Scheme 1b).^5^ Pd- or Ni-catalyzed carboxylation of propargylic compounds with CO in the presence of water (Scheme 1c)^6^ and the carboxylation of 2-alkynyl bromides or allenylmetallic reagents with CO_2_ (Scheme 1d)^7^ have also been reported. Crabbé homologation of *o*-methoxycarbonylphenylacetylene with paraformaldehyde results in the formation of methyl 2-propadienylbenzoate, which undergoes hydrolysis to afford the corresponding allenoic acid (Scheme 1e).^8^ The limitations are harsh conditions and the use of toxic carbon monoxide, stoichiometric amounts of reductants and limited substrates. On the other hand, C–H activation has been proven to be a powerful tool in synthetic chemistry because of the atom- and step-economy.^9^ The synthesis of allenes based on C–H activation is undoubtedly an ideal strategy.^10^ We reasoned that the most straightforward approach to allenoic acids would be the use of benzoic acids with carboxylic acid acting as an inherent directing group.^11^ Herein, we wish to report the realization of Ru-catalyzed synthesis of allenoic acids *via* direct C–H allenylation of benzoic acids (Scheme 1f).

**Scheme 1:**
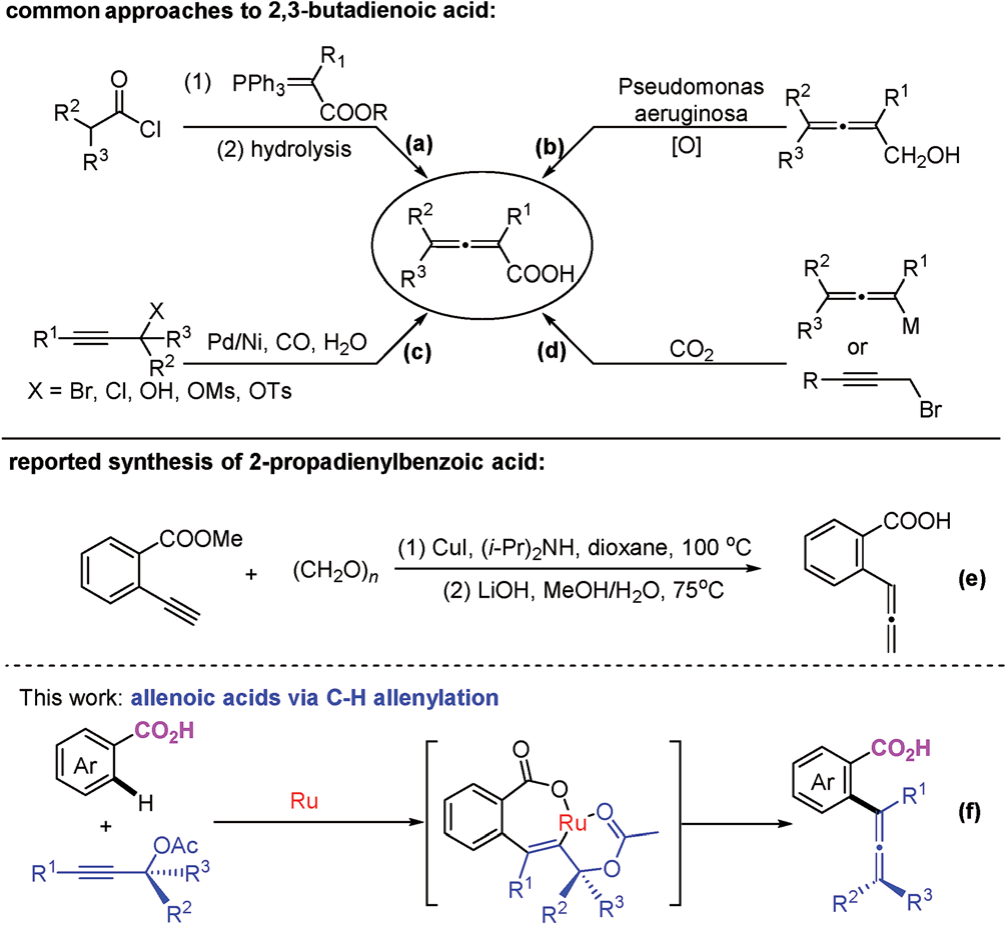
Approaches to allenoic acids.

Our initial attempt began with benzoic acid **1a** and propargylic acetate **2a** in the presence of [Ru(*p*-cymene)Cl_2_]_2_ and NaOAc at 50 °C using toluene as the solvent. To our delight, the monoallenylation product **3aa** was generated in 7% yield together with 67% recovery of **2a** (Table 1, entry 1). We then investigated the effect of the solvent (Table 1, entries 2–7). The reaction could proceed in dioxane, DCE, CH_3_CN, THF, and even in water, albeit affording the monoallenylation product **3aa** and the double allenylation product **4aa** in rather low yields (Table 1, entries 2–6). To our surprise, the yield could be greatly improved when the reaction was conducted in MeOH: 36% yield of the monoallenylation product **3aa** and 16% yield of the double allenylation product **4aa** were obtained (Table 1, entry 7). We next examined a series of additives as shown in entries 8–13: when K_2_CO_3_ was employed, the reaction gave a 64% combined yield of **3aa** and **4aa** (Table 1, entry 13). The reaction in EtOH under optimal conditions led to increased combined yield but a lower selectivity of **3aa**/**4aa** (Table 1, entries 13–14). When 2.6 equiv. of benzoic acid were used, the yield was the highest with a selectivity of 58/14 (Table 1, entry 17). When the reaction was conducted in air, the influence was negligible (Table 1, entry 19). In the absence of the base, we only observed the decomposition of propargylic acetate.

**Table 1:** Optimization of the *ortho*-allenylation of benzoic acid **1a**^*a*^

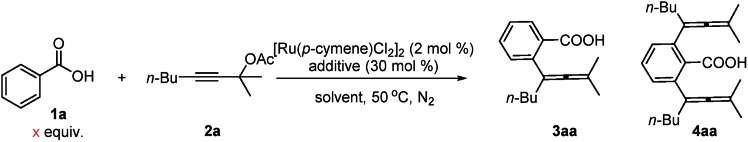						
Entry	*x*	Solvent	Additive	Time (h)	Combined yield (**3aa**/**4aa**)^*b*^ (%)	Recovery of **2a**^*b*^ (%)
1	1.5	Toluene	NaOAc	46	7 (7/0)	67
2	1.5	Dioxane	NaOAc	46	15 (12/3)	20
3	1.5	DCE	NaOAc	46	14 (14/0)	24
4	1.5	CH_3_CN	NaOAc	46	27 (23/4)	26
5	1.5	THF	NaOAc	46	30 (23/7)	6
6	1.5	H_2_O	NaOAc	46	19 (12/7)	23
7	1.5	MeOH	NaOAc	46	52 (36/16)	13
8	1.5	MeOH	NaOAc	12	57 (39/18)	29
9	1.5	MeOH	K_3_PO_4_	12	41 (30/11)	7
10	1.5	MeOH	*t*-BuONa	12	22 (18/4)	21
11	1.5	MeOH	Na_2_CO_3_	12	57 (40/17)	18
12	1.5	MeOH	Cs_2_CO_3_	12	60 (42/18)	20
13	1.5	MeOH	K_2_CO_3_	12	64 (45/19)	27
14	1.5	EtOH	K_2_CO_3_	12	69 (36/33)	9
15	2.0	MeOH	K_2_CO_3_	12	66 (48/18)	16
16	2.4	MeOH	K_2_CO_3_	12	63 (50/13)	16
17	2.6	MeOH	K_2_CO_3_	12	72 (58/14)	20
18	3.0	MeOH	K_2_CO_3_	12	61 (52/9)	21
19^*c*^	2.6	MeOH	K_2_CO_3_	12	67 (56/11)	18
20^*c*^	2.6	MeOH	—	12	0	42

^*a*^The reaction was conducted with **1a**, **2a** (0.2 mmol), [Ru(*p*-cymene)Cl_2_]_2_ (0.004 mmol), and an additive (0.06 mmol) in a solvent (0.5 mL) at 50 °C.

^*b*^Determined by ^1^H NMR analysis using CH_2_Br_2_ as the internal standard.

^*c*^In air.

We further investigated the effect of the leaving group (LG) by studying the reaction of benzoic acid **1a** with several propargylic alcohol derivatives and found that OAc was still the best leaving group (Table 2).

**Table 2:** Effect of the leaving groups

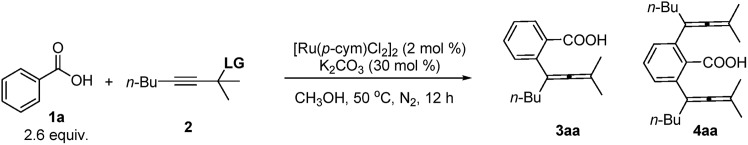			
Entry	LG	Combined yield (**3aa**/**4aa**)^*a*^ (%)	Recovery of **2a**^*a*^ (%)
1	OMe (**2a_1_**)	0 (—/—)	59
2	OCO_2_Me (**2a_2_**)	54 (40/14)	9
3	OCOEt (**2a_3_**)	57 (47/10)	20
4	OBoc (**2a_4_**)	61 (47/14)	21
5	OAc (**2a**)	72 (58/14)	20

^*a*^Determined by ^1^H NMR analysis using CH_2_Br_2_ as the internal standard.

With the optimized reaction conditions in hand, the scope of the reaction was investigated at the 1.0 mmol scale (eqn (1) and (2) and Table 3). The parent benzoic acid **1a** afforded the monoallenylation product **3aa** in 55% yield together with the diallenylation product **4aa** in 10% yield (eqn (1)). 4-Bromobenzoic acid **1n** was converted to the monoallenylation product **3na** (45% yield) and the bisallenylation product **4ma** (26% yield) under the standard conditions (eqn (2)).1
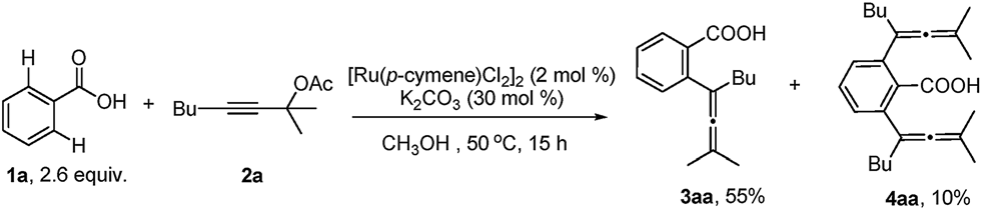

2
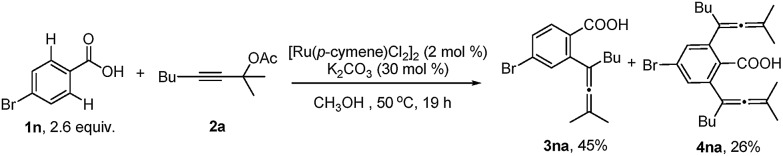



**Table 3:** Reaction scope^*a*^

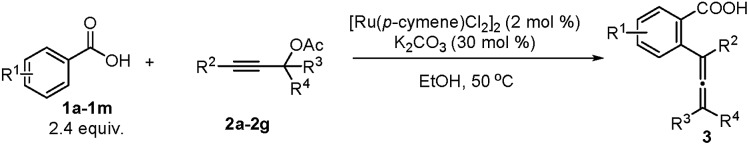
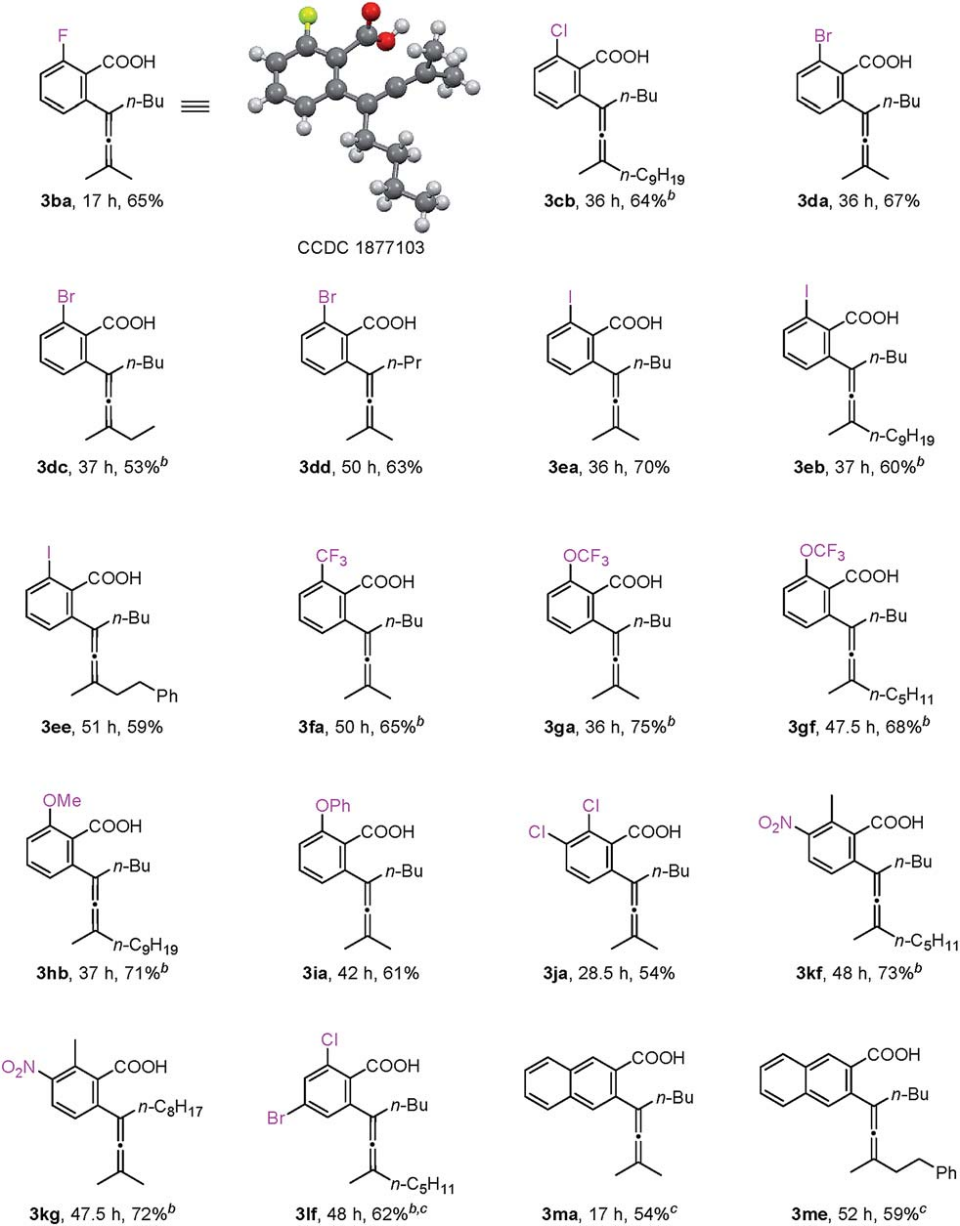

^*a*^The reaction was conducted with **1** (2.4 mmol), **2** (1.0 mmol), [Ru(*p*-cymene)Cl_2_]_2_ (0.02 mmol), and K_2_CO_3_ (0.3 mmol) in EtOH (2.5 mL) at 50 °C.

^*b*^4 mol% [Ru(*p*-cymene)Cl_2_]_2_.

^*c*^5.0 mL of EtOH as the solvent.

For mono-*o*-substituted benzoic acids, electron withdrawing groups, such as halogen atoms (including fluorine, chlorine, bromine, and iodine), CF_3_, and OCF_3_, were all well tolerated (Table 3, **3ba–3gf**). The structure of **3ba** was confirmed by X-ray diffraction study.^12^ Mono-*o*-substituted benzoic acids containing the electron-donating groups methoxy and phenoxy afforded allenylation products **3hb** in 71% yield and **3ia** in 61% yield, respectively. 2,3-Dichlorobenzoic acid (**3ja**), 2-methyl-3-nitrobenzoic acid (**3kf** and **3kg**) and 2-chloro-4-bromobenzoic acid (**3lf**) were also allenylated in moderate to good yields. Notably, when the reaction was conducted with β-naphthoic acid, which has more than one C–H bond, 3-allenylation products **3ma** and **3me** were obtained exclusively. The scope of C–H allenylation with regard to propargylic acetates was also investigated affording **3cb**, **3nb**, **3dc**, **3dd**, **3ee**, **3le**, **3kf**, **3gf**, or **3kg** smoothly.

A gram scale reaction using 2-bromobenzoic acid **1d** with **2g** afforded the allenylation product **3dg** in 67% yield (eqn (3)).3
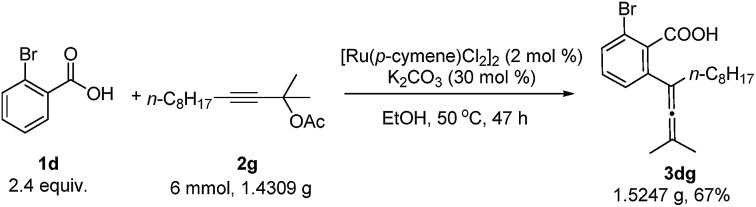



In addition, when the optically active acetate (*S*)-**2f** (99% ee) was applied, axially chiral allenoic acids (*S*_a_)-**3gf** (99% ee) and (*S*_a_)-**3kf** (97% ee) could be obtained with highly efficient chirality transfer (Scheme 2a). This method may open a new avenue for developing practical and synthetically useful methodologies for the synthesis of optically active allenoic acids. Meanwhile, this result indicated that the coordination of acetates and ruthenium species dictated the regioselectivity of alkyne insertion and the stereoselectivity of β-OAc elimination.

**Scheme 2:**
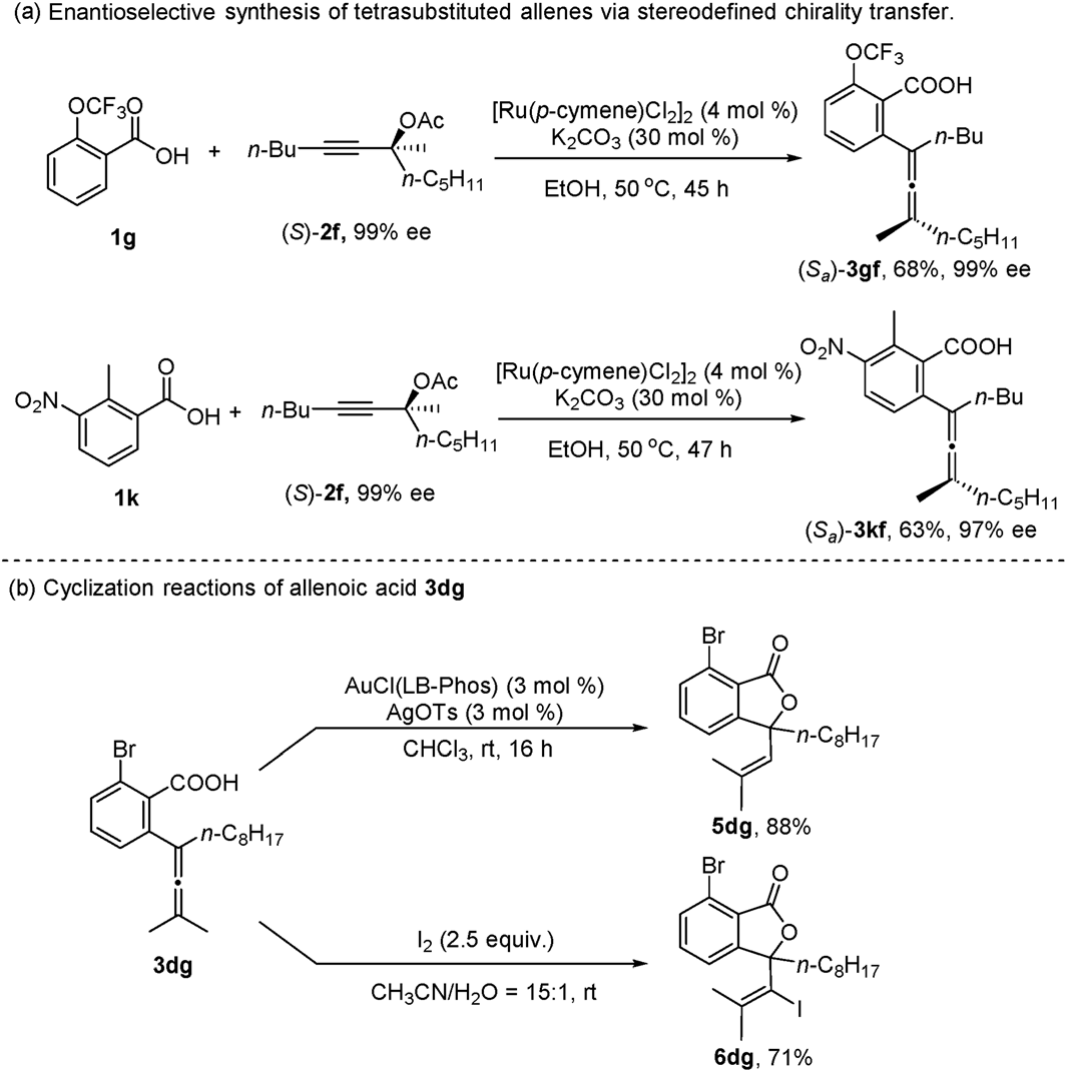
Synthetic applications.

To further explore the synthetic utility of this method, several synthetic applications were studied (Scheme 2b). The allenoic acid **3dg** was easily transformed into the lactone **5dg** by treatment with AuCl(LB-Phos) and AgOTs.^13^ This allenoic acid may also undergo an iodolactonization reaction with iodine to afford **6dg** in 71% yield.

To gain insight into the mechanism of this methodology, several control experiments were carried out. Firstly, when 2-fluorobenzoic acid **1b** was added to a mixture of CD_3_OD and D_2_O (2 : 1), the corresponding benzoic acid **D-1b** with 95% deuterium incorporation was obtained (Scheme 3a), indicating that the C–H activation step was reversible in the catalytic system. Subsequently, the parallel reactions of **1b** and **D-1b** with **2a** were conducted. We measured the reaction rate (*k*) of both **1b** and **D-1b** by monitoring the concentration of the product **3ba** by NMR from 2.5 h to 8 h (Scheme 3b and Fig. 1). Then, the primary kinetic isotope effect of 3.6 was observed. These results suggest that C–H bond cleavage is the rate-determining step.^14^

**Scheme 3:**
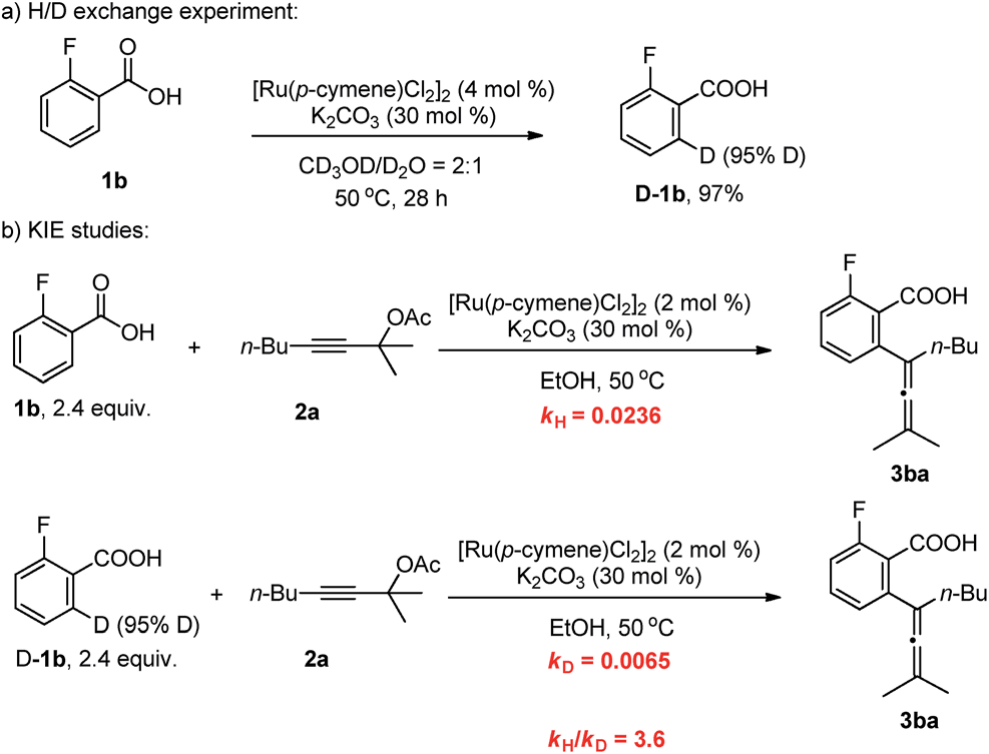
Mechanistic studies.

**Fig. 1:**
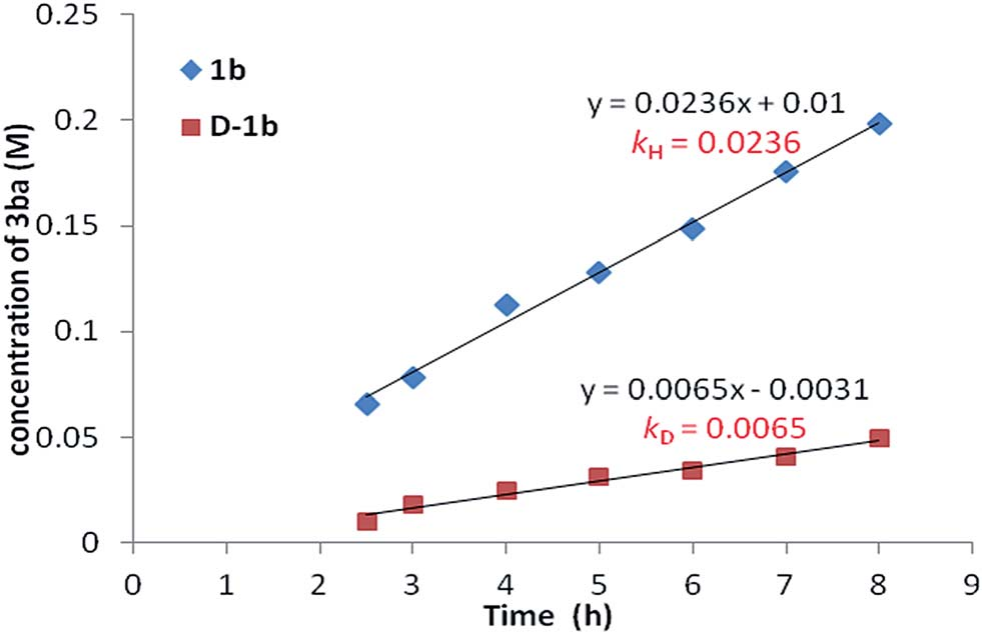
Plot of the concentrations of **3ba***vs.* time.

In addition, a first-order dependence of the initial rate on the amount of the Ru catalyst was established (Fig. 2a, see the ESI† for details). The reaction orders of each reactant were also measured by using 2-fluorobenzoic acid **1b** and propargylic acetate **2a**. Both **1b** and **2a** follow the first-order reaction rate law, according to the linear relationship with ln([**1b**]) *vs.* reaction time: ln([**1b**]) = –*k*_1_*t* + ln[**1b_0_**] (Fig. 2b) and ln([**2a**]) = –*k*_2_*t* + ln[**2a_0_**] (Fig. 2c). Based on these data, we may give the rate equation as d[**3ba**]/d*t* = *k*[Ru]·[**1b**]·[**2a**].

**Fig. 2:**
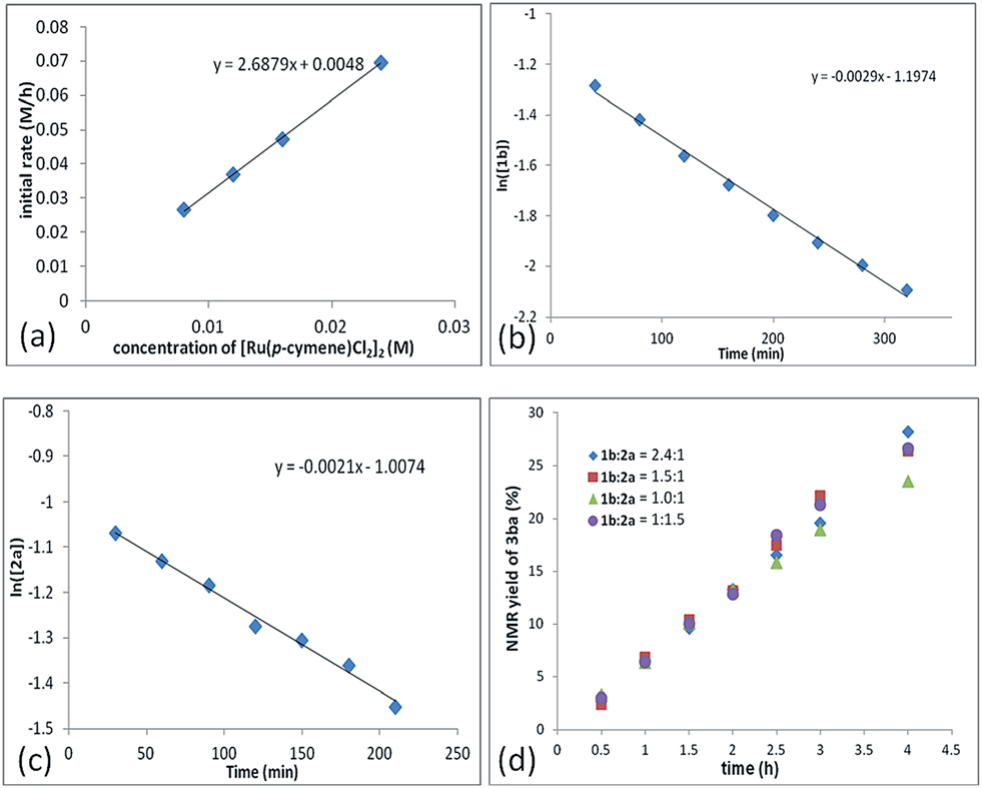
The dependence of the initial reaction rate on [Ru(*p*-cymene)Cl_2_]_2_ (a), 2-fluorobenzoic acid **1b** (b), and propargylic acetate **2a** (c). NMR yield of **3ba***vs.* time with different molar ratios of **1b** and **2a** (d).

To further understand the role of benzoic acid on the reaction, four experiments were conducted by using different molar ratios of 2-fluorobenzoic acid **1b***vs.* propargylic acetate **2a** (Fig. 2d). The yield *vs.* time profile is almost the same in the initial four hours, indicating that the loading of benzoic acid has a very limited effect on the formation of the final product; the excess benzoic acid did not accelerate the formation of the product greatly.^15^ In addition, we observed that the reaction failed to afford the expected product in the absence of k_2_CO_3_, indicating a CMD process for the C–H cleavage. However, due to its catalytic nature, the role of benzoic acid as a Brønsted acid to promote the insertion process from **Int 2** to **Int 3** cannot be excluded.^16^

Based on these investigations above, the proposed catalytic cycle is illustrated in Scheme 4. Firstly, the C–H activation step leads to the formation of the cyclic intermediate **Int 1***via* a CMD process. Subsequently, **Int 2** is generated by the coordination of the carbonyl unit in acetate with the Ru in the cycloruthenated species, which subsequently undergoes the *syn*-insertion of a C–C triple bond to afford **Int 3**. After a *syn*-β-OAc elimination step, the allenylation product was generated and the ruthenium species was released to restart the cycle. It should be noted that the acetate plays an important role in the *syn*-insertion as well as the *syn*-β-OAc elimination step.

**Scheme 4:**
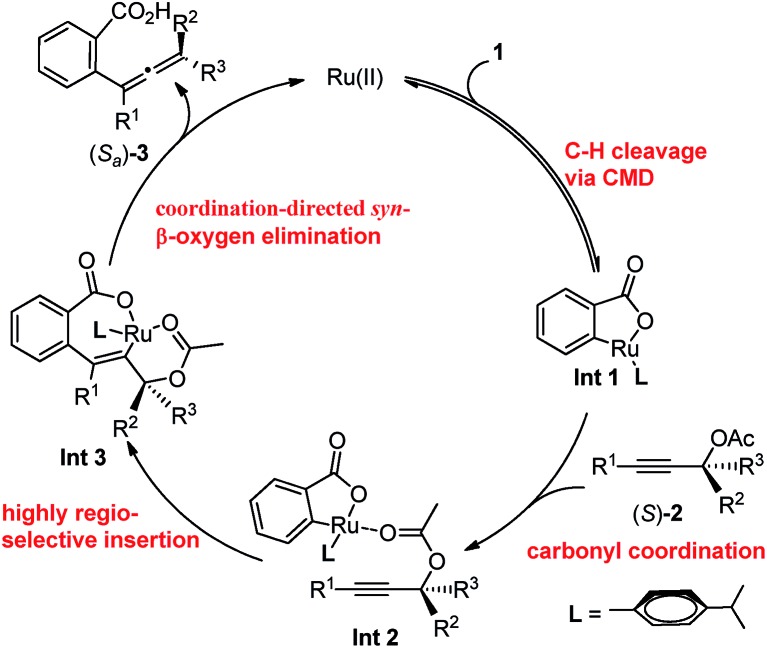
A possible mechanism.

## Conclusions

In conclusion, we have established a new strategy to access allenoic acids, which is based on ruthenium catalysed carboxylic acid-directed C–H allenylation of benzoic acids with propargylic acetates. The reaction is compatible with air and synthetically useful functional groups such as Cl, Br, I, and OCF_3_ are all tolerated. Optically active allenoic acids could also be prepared through highly efficient chirality transfer. The formed allenoic acids could be transformed to lactones efficiently under mild conditions.

## Conflicts of interest

There are no conflicts to declare.

## Supplementary Material

Supplementary information

Crystal structure data
