# Supplementary material for: A ruthenium(ii)-catalyzed C–H allenylation-based approach to allenoic acids
Source: Chem Sci. 2019 May 10;10(25):6316–21. doi: 10.1039/c9sc00603f (PMC6598647; doi:10.1039/c9sc00603f)
Supplement: Supplementary file 1 [file SC-010-C9SC00603F-s001.pdf]

Electronic Supplementary Material (ESI) for Chemical Science.
This journal is © The Royal Society of Chemistry 2019

Ruthenium(II)-Catalyzed C-H Allenylation-Based Approach to Allenoic Acids

Xiaoyan Wu, Junjie Fan, Chunling Fu, and Shengming Ma*

Laboratory of Molecular Recognition and Synthesis, Department of Chemistry, Zhejiang University, Hangzhou 310027, Zhejiang, P. R. China.

E-mail: masm@sioc.ac.cn

Supporting Information

Table of Contents

General experimental methods	S2
Synthesis of new starting materials	S3-S7
Ru(II)-catalyzed *ortho*-C-H allenylation of benzoic acids	S7-S27
Synthetic applications	S28-S29
Mechanistic studies	S30-S38
References	S39
^{1}H NMR, ^{13}C NMR, and HPLC spectra of the compounds prepared	S40-S124

General Information

^{1}H NMR, ^{13}C NMR, and ^{19}F NMR spectra were recorded in $CDCl_3$ using a Bruker AM 300 MHz NMR spectrometer (^{1}H at 300 MHz, ^{13}C at 75 MHz, ^{19}F at 282 MHz) or a Bruker AM 400 MHz NMR spectrometer (^{1}H at 400 MHz, ^{13}C at 100 MHz, ^{19}F at 376 MHz) using TMS (^{1}H, $\delta = 0$), residual $CHCl_3$ (7.26 ppm) in $CDCl_3$, and $CFCl_3$ (^{19}F $CFCl_3$, $\delta = 0$) as the internal standards, respectively. IR spectra were recorded with a Perkin–Elmer 983G instrument. Elemental analyses were measured with a Carlo-Erba EA1110 elementary analysis instrument. Mass spectrometry was performed with an HP 5989A system. High-resolution mass spectrometry was determined with a Finnigan MAT 8430 or Bruker APEXIII instrument. [Ru(*p*-cymene)Cl_2]$_2$ was purchased from *J&K Scientific*. The boiling range of the petroleum ether was 60-90 ^{o}C unless noted otherwise. Other commercially available chemicals including benzoic acids were purchased and used without additional purification unless noted otherwise. Propargylic acetates were prepared according to the literature procedures.[1] The apparatus used in this study is shown as follows:

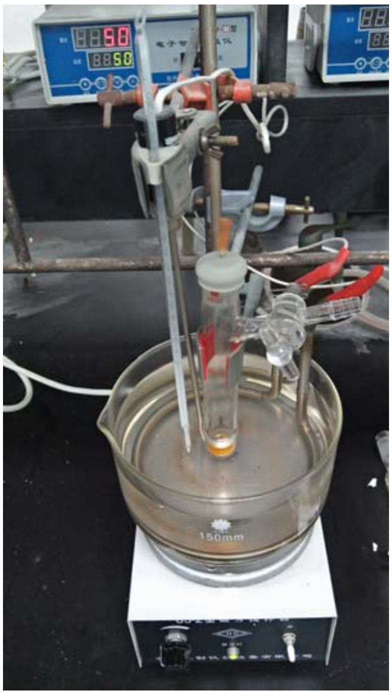

Synthesis of new starting materials

1. Synthesis of 3-methylnon-4-yn-3-yl acetate **2c**. [1] (wxy-2-155)

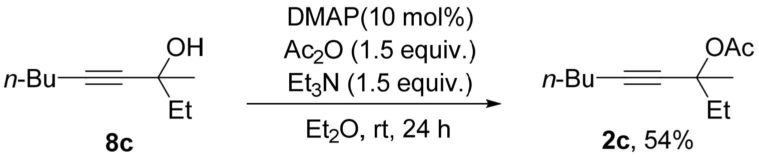

Typical Procedure I: To a dried round flask were added DMAP (0.3065 g, 3.0 mmol), Et_3N (6.4 mL, *d* = 0.73 g/mL, 4.6720 g, 46.1 mmol), **8c** (5.8880 g, 30 mmol)/Et_2O (50 mL), and Ac_2O (4.3 mL, *d* = 1.08 g/mL, 4.6440 g, 45.5 mmol) sequentially. The reaction was complete after 24 h as monitored by TLC (eluent: petroleum ether/ethyl acetate = 25/1). To the resulting mixture was added an aqueous solution of saturated NH_4Cl. The organic phase was separated and the aqueous phase was extracted with 30 mL of ethyl acetate. The combined organic phase then washed with brine and dried over anhydrous Na_2SO_4. After filtration, evaporation of the solvent and chromatography on silica gel (eluent: petroleum ether/ethyl acetate = 25/1, 1500 mL) afforded **2c** (3.1611 g, 54%) as a liquid: ^{1}H NMR (300 MHz, $CDCl_3$) δ 2.22 (t, *J* = 6.9 Hz, 2 H, CH_2), 2.01 (s, 3 H, OAc), 2.00-1.88 (m, 1 H, one proton of CH_2), 1.86-1.73 (m, 1 H, one proton of CH_2), 1.62 (s, 3 H, CH_3), 1.54-1.33 (m, 4 H, $CH_2 \times 2$), 1.00 (t, *J* = 7.5 Hz, 3 H, CH_3), 0.91 (t, *J*= 7.2 Hz, 3 H, CH_3); ^{13}C NMR (75 MHz, $CDCl_3$) δ 169.3, 85.6, 80.2, 76.3, 34.6, 30.7, 26.2, 22.0, 21.8, 18.3, 13.5, 8.5; IR (neat) *ν* (cm^{-1}) 2961, 2937, 2875, 2241, 1746, 1464, 1368, 1329, 1304, 1242, 1164, 1138, 1117, 1038, 1015; MS (EI): *m/z* (%) 196 (M^+, 3.47), 154.2 (M^+- Ac, 99.63), 43 (100); HRMS Calcd for $C_{12}H_{20}O_2$ (M^+): 196.1463; Found: 196.1462.

2. Synthesis of 7-methylhexadec-5-yn-7-yl acetate **2b**. [1] (wxy-2-158)

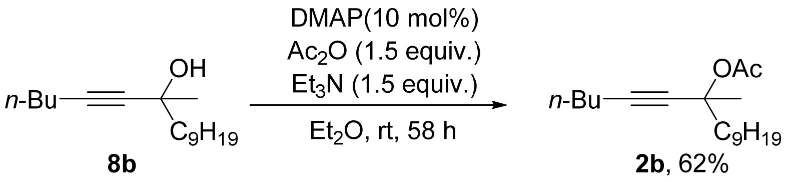

Following **Typical Procedure I**, the reaction of **8b** (5.8912 g, 20 mmol), DMAP (204.5 mg, 2 mmol), Et_3N (4.2 mL, d = 0.73 g/mL, 3.066 g, 30.3 mmol,), and Ac_2O (2.9 mL, d = 1.08 g/mL, 3.132 g, 30.7 mmol) in 35 mL Et_2O afforded **2b** (3.6521 g, 62%) (eluent: petroleum ether/ethyl acetate = 25/1, 1500 mL) as a liquid: ^{1}H NMR (300 MHz, $CDCl_3$) δ 2.21 (t, J = 6.9 Hz, 2 H, CH_2), 2.00 (s, 3 H, OAc), 1.98-1.85 (m, 1 H, one proton of CH_2), 1.81-1.65 (m, 1 H, one proton of CH_2), 1.63 (s, 3 H, CH_3), 1.52-1.34 (m, 6 H, CH_2 × 3), 1.34-1.19 (m, 12 H, CH_2 × 6), 0.94-0.82 (m, 6 H, CH_3 × 2); ^{13}C NMR (75 MHz, $CDCl_3$) δ 169.3, 85.5, 80.6, 76.0, 41.7, 31.9, 30.7, 29.52, 29.50, 29.3, 26.7, 24.2, 22.6, 22.0, 21.8, 18.4, 14.0, 13.5; IR (neat) v (cm^{-1}) 2955, 2927, 2856, 2245, 1747, 1467, 1367, 1328, 1237, 1166, 1015; MS (EI): m/z (%) 294 (M$^+$, 42.04), 252 (100); HRMS Calcd for $C_{19}H_{34}O_2$ (M$^+$): 294.2559; Found: 294.2557.

3. Synthesis of 3-methyl-1-phenylnon-4-yn-3-yl acetate **2e**. [1] (wxy-2-189)

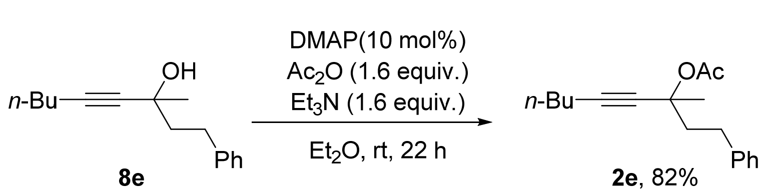

Following **Typical Procedure I**, the reaction of **8e** (0.9217 g, 4 mmol), DMAP (41.0 mg, 0.4 mmol), Et_3N (0.9 mL, d = 0.73 g/mL, 0.657 g, 6.5 mmol,), and Ac_2O (0.6 mL, d = 1.08 g/mL, 0.648 g, 6.4 mmol) in 10 mL Et_2O afforded **2e** (0.8510 g, 82%) (eluent: petroleum ether/ethyl acetate = 30/1, 800 mL) as a liquid: ^{1}H NMR (300 MHz, $CDCl_3$) δ 7.33-7.14 (m,

5 H, ArH), 2.81 (t, J = 8.4 Hz, 2 H, CH_2), 2.32-2.19 (m, 3 H, CH_2 and one proton of CH_2), 2.11-2.00 (m, 1 H, one proton of CH_2), 1.99 (s, 3 H, OAc), 1.70 (s, 3 H, CH_3), 1.58-1.36 (m, 4 H, CH_2 × 2), 0.92 (t, J = 7.2 Hz, 3 H, CH_3); ^{13}C NMR (75 MHz, $CDCl_3$) δ 169.3, 141.7, 128.4, 128.3, 125.8, 86.0, 80.1, 75.5, 43.6, 30.8, 30.6, 26.8, 21.9, 21.8, 18.3, 13.5; IR (neat) v (cm^{-1}) 3092, 3063, 3027, 2953, 2934, 2873, 2244, 1747, 1742, 1739, 1733, 1604, 1498, 1455, 1369, 1236, 1169, 1088, 1064, 1015; MS (EI): m/z (%) 272 (M^+, 1.98), 181 (100); HRMS Calcd for $C_{18}H_{24}O_2$ (M^+): 272.1776; Found: 272.1776.

4. Synthesis of (S)-6-methyldodec-7-yn-6-yl acetate (*S*)-**2f**.[1,2] (wxy-3-155)

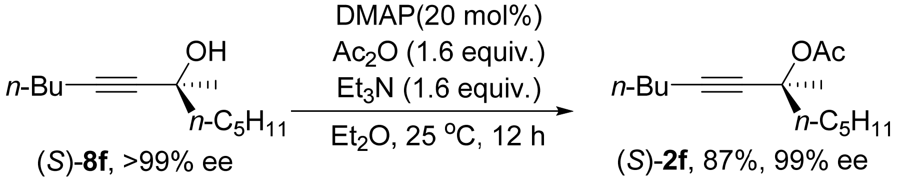

Compound (*S*)-**8f**[2] was prepared by preparative HPLC separation of racemic 6-methyldodec-7-yn-6-ol **8f**: >99% ee (HPLC conditions: Chiralcel AD-H column, hexane/i-PrOH = 99/1, 1.0 mL/min, λ = 214 nm, t_R(major) = 9.8 min, t_R(minor) = 10.7 min); (*S*)-**2f** was prepared following **Typical Procedure I**: the reaction of (*S*)-**8f** (393.7 mg, 2.0 mmol), DMAP (41.0 mg, 0.4 mmol), Et_3N (0.42 mL, d = 0.73 g/mL, 306.6 mg, 3.1 mmol), and Ac_2O (0.3 mL, d = 1.08 g/mL, 324.0 mg, 3.2 mmol) in 2.0 mL Et_2O afforded (*S*)-**8f** (416.1 mg, 87%) (eluent: petroleum ether/ethyl acetate = 50/1, 500 mL) as an oil: 99% ee (HPLC conditions: Chiralcel OZ-H column, *n*-hexane/*i*-PrOH = 100/1, 1.0 mL/min, λ = 214 nm, t_R(major) = 11.4 min, t_R(minor) = 15.9 min); $[\alpha]_D^{20}$ = -31.1 (c = 0.92, $CHCl_3$); ^1H NMR (300 MHz, $CDCl_3$) δ 2.21 (t, J = 7.1 Hz, 2 H, CH_2), 2.01 (s, 3 H, CH_3), 1.98-1.83 (m, 1 H, one proton of CH_2), 1.81-1.68 (m, 1 H, one proton of CH_2), 1.63 (s, 3 H, CH_3), 1.55-1.21 (m,

10 H, $CH_2 \times 5$), 0.90 (t, J = 7.2 Hz, 6 H, $CH_3 \times 2$); ^{13}C NMR (75 MHz, $CDCl_3$) δ 169.3, 85.5, 80.5, 76.0, 41.6, 31.7, 30.7, 26.7, 23.9, 22.5, 22.1, 21.8, 18.4, 13.9, 13.5; IR (neat) v (cm^{-1}) 2958, 2934, 2873, 2249, 1747, 1467, 1367, 1240, 1160, 1122, 1049, 1013; MS (EI): m/z (%) 238 (M^+, 0.93), 43 (100); HRMS Calcd for $C_{15}H_{26}O_2$ $(M+Na)^+$: 261.1830; Found: 261.1827.

Ru(II)-Catalyzed C-H Allenylation of Benzoic Acids

1. Synthesis of 2-(2-methylocta-2,3-dien-4-yl)benzoic acid **3aa** and 2,6-bis(2-methylocta-2,3-dien-4-yl)benzoic acid **4aa**. (wxy-2-083, wxy-1-160)

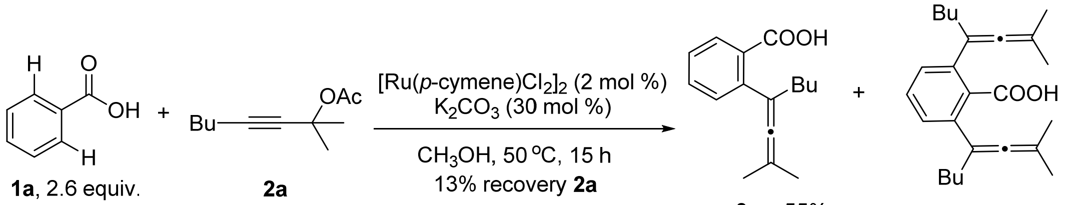

1a, 2.6 equiv. **2a** **3aa**, 55% **4aa**, 10%

Typical Procedure II: To a dried Schlenk tube were sequentially added benzoic acid **1a** (317.4 mg, 2.6 mmol), K_2CO_3 (41.9 mg, 0.3 mmol), [Ru(p-cymene)Cl_2]$_2$ (12.4 mg, 0.02 mmol), 2-methyloct-3-yn-2-yl acetate **2a** (182.5 mg, 1 mmol), and CH_3OH (2.5 mL) in open air atmosphere. The reaction tube was put into an oil bath preheated to 50 °C. The reaction was complete after being stirred for 15 h as monitored by TLC. After filteration through a short column of silica gel eluted with ethyl acetate (20 mL × 3) and concentration in vacuo, the crude residual was purified by chromatography on silica gel [eluent: petroleum ether/ethyl acetate = 10/1 (500 mL) to petroleum ether/ethyl acetate = 20/1 (1000 mL)] to afford **3aa** (134.3 mg, 55%) and **4aa** (18.9 mg, 10%). 13% recovery of **2a** was determined by ^1H NMR analysis of the crude product using 35 μL of CH_2Br_2 as the internal standard.

3aa: oil; ^1H NMR (300 MHz, $CDCl_3$) δ 11.80 (bs, 1 H, COOH), 7.81 (dd, J_1 = 7.8 Hz, J_2 =

1.1 Hz, 1 H, ArH), 7.45 (td, J_1 = 7.5 Hz, J_2 = 1.5 Hz, 1 H, ArH), 7.36-7.24 (m, 2 H, ArH), 2.34 (t, J = 7.1 Hz, 2 H, CH_2), 1.71 (s, 6 H, 2 × CH_3), 1.52-1.33 (m, 4 H, 2 × CH_2), 0.91 (t, J = 7.1 Hz, 3 H, CH_3); ^{13}C NMR (75 MHz, $CDCl_3$) δ 200.8, 174.8, 141.6, 131.8, 130.1, 129.6, 129.5, 126.3, 103.3, 96.9, 33.3, 30.1, 22.2, 20.1, 14.0; IR (neat) v (cm^{-1}) 3527-2082 (COOH), 1957, 1695, 1598, 1570, 1487, 1451, 1407, 1377, 1362, 1299, 1264, 1138, 1085; MS (EI): *m/z* (%) 244 (M^+, 6.53), 187 (100); HRMS Cacld. for $C_{16}H_{20}O_2$ (M^+): 244.1463; Found: 244.1465.

4aa: oil; ^{1}H NMR (300 MHz, $CDCl_3$) δ 11.05 (bs, 1 H, COOH), 7.31 (dd, J_1 = 8.3 Hz, J_2 = 7.1 Hz, 1 H, ArH), 7.14 (d, J = 7.2 Hz, 2 H, ArH) , 2.28 (t, J = 7.2 Hz, 4 H, 2 × CH_2), 1.71 (s, 12 H, 4 × CH_3), 1.50-1.30 (m, 8 H, 4 × CH_2), 0.90 (t, J = 7.1 Hz, 6 H, 2 × CH_3); ^{13}C NMR (75 MHz, $CDCl_3$) δ 200.7, 173.6, 138.4, 131.6, 129.0, 126.7, 102.1, 96.9, 33.9, 30.0, 22.3, 20.5, 14.0; IR (neat) v (cm^{-1}) 3402-2211 (COOH), 1959, 1699, 1576, 1456, 1377, 1362, 1286, 1188, 1131; MS (EI): *m/z* (%) 366 (M^+, 100.00); HRMS Cacld. for $C_{25}H_{34}O_2$ (M^+): 366.2559; Found: 366.2558.

2. Synthesis of 2-fluoro-6-(2-methylocta-2,3-dien-4-yl)benzoic acid **3ba**. (wxy-2-132)

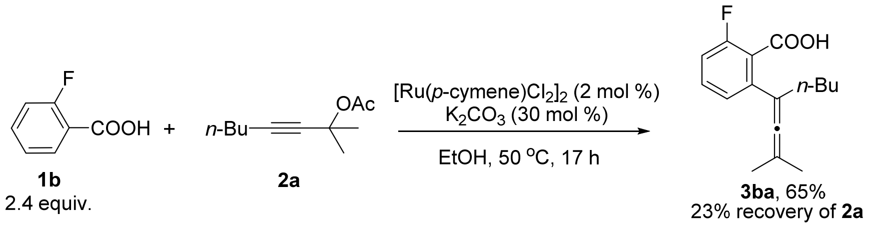

Following **Typical Procedure II**, the reaction of **1b** (336.2 mg, 2.4 mmol), **2a** (182.5 mg, 1 mmol), **2b** (336.2 mg, 1.0 mmol), K_2CO_3 (41.5 mg, 0.3 mmol), and [Ru(*p*-cymene)Cl_2]$_2$ (12.2 mg, 0.02 mmol) in 2.5 mL of EtOH afforded **3ba** (170.4 mg, 65%) as a solid (eluent:

petroleum ether/ethyl acetate = 20/1, 1500 mL): m.p. 85.9-86.0 °C (petroleum ether/ethyl acetate); 23% recovery of **2b** was determined by ^{1}H NMR analysis of the crude product using 35 μL CH_2Br_2 as the internal standard. ^{1}H NMR (300 MHz, $CDCl_3$) δ 12.20 (bs, 1 H, COOH), 7.35 (td, J_1 = 8.1 Hz, J_2 = 6.0 Hz, 1 H, ArH), 7.14 (d, J = 7.2 Hz, 1 H, ArH), 6.97 (dt, J_1 = 8.7 Hz, J_2 = 0.6 Hz, 1 H, ArH), 2.38 (t, J = 7.2 Hz, 2 H, CH_2), 1.75 (s, 6 H, 2 × CH_3), 1.53-1.32 (m, 4 H, 2 × CH_2), 0.92 (t, J = 7.1 Hz, 3 H, CH_3); ^{13}C NMR (75 MHz, $CDCl_3$) δ 202.2, 172.9, 159.6 (d, J = 248.2 Hz), 140.1 (d, J = 2.0 Hz), 131.0 (d, J = 9.0 Hz), 122.6 (d, J = 2.8 Hz), 120.5 (d, J = 15.9 Hz), 113.4 (d, J = 21.4 Hz), 100.9 (d, J = 2.0 Hz), 99.3, 32.2, 30.0, 22.2, 19.7, 14.0; ^{19}F NMR (282 MHz, $CDCl_3$) δ -116.21; IR (neat) v (cm^{-1}) 3300-2200 (COOH), 1955, 1704, 1699, 1609, 1575, 1456, 1404, 1362, 1296, 1262, 1236, 1125, 1057; Raman v (cm^{-1}) 1950, 1609; MS (EI): m/z (%) 262 (M^+, 14.38), 205 (100); Anal. Calcd. for $C_{16}H_{19}FO_2$ (%): C, 73.26; H, 7.30; Found: C, 72.89; H, 7.19.

3. Synthesis of 2-chloro-6-(7-methylhexadeca-5,6-dien-5-yl)benzoic acid **3cb**. (wxy-2-195)

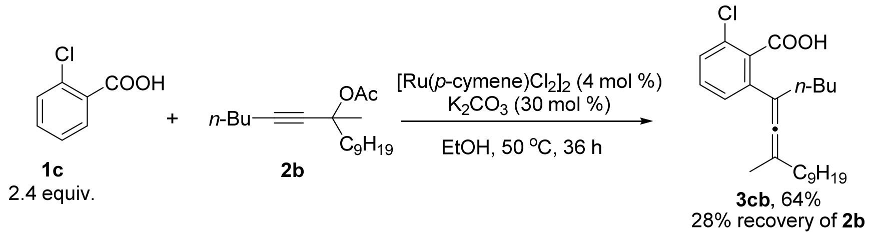

Following **Typical Procedure II**, the reaction of **1c** (375.4 mg, 2.4 mmol), **2b** (295.0 mg, 1.0 mmol), K_2CO_3 (41.5 mg, 0.3 mmol), and [Ru(p-cymene)Cl_2]$_2$ (24.4 mg, 0.04 mmol) in 2.5 mL of EtOH afforded **3cb** (249.2 mg, 64%) as an oil (eluent: petroleum ether/ethyl acetate/HOAc = 500/30/4, 1500 mL). 28% recovery of **2b** was determined by ^{1}H NMR analysis of the crude product using 35 μL CH_2Br_2 as the internal standard. ^{1}H NMR (300

MHz, $CDCl_3$) δ 11.35 (bs, 1 H, COOH), 7.33-7.20 (m, 3 H, ArH), 2.44-2.28 (m, 2 H, CH_2), 1.99 (m, 2 H, CH_2), 1.77 (s, 3 H, CH_3), 1.51-1.31 (m, 6 H, CH_2 × 3), 1.31-1.11 (m, 12 H, CH_2 × 6), 0.91 (t, J = 6.9 Hz, 3 H, CH_3), 0.87 (t, J = 6.6 Hz, 3 H, CH_3); ^{13}C NMR (75 MHz, $CDCl_3$) δ 201.3, 173.7, 139.9, 131.9, 130.9, 130.2, 127.3, 125.8, 103.2, 102.3, 34.0, 33.0, 31.9, 30.1, 29.6, 29.5, 29.29, 29.27, 27.3, 22.6, 22.3, 18.3, 14.1, 13.9; IR (neat) v (cm^{-1}) 3535-2138 (COOH), 1952, 1704, 1700, 1588, 1564, 1464, 1398, 1287, 1189, 1154, 1129; MS (EI): *m/z* (%) 392 [M^+(^{37}Cl), 2.62], 390 [M^+(^{35}Cl), 7.42], 333 (100); HRMS Calcd for $C_{24}H_{35}O_2{}^{35}Cl$ (M^+): 390.2326; Found: 390.2327.

4. Synthesis of 2-bromo-6-(2-methylocta-2,3-dien-4-yl)benzoic acid **3da**. (wxy-2-190)

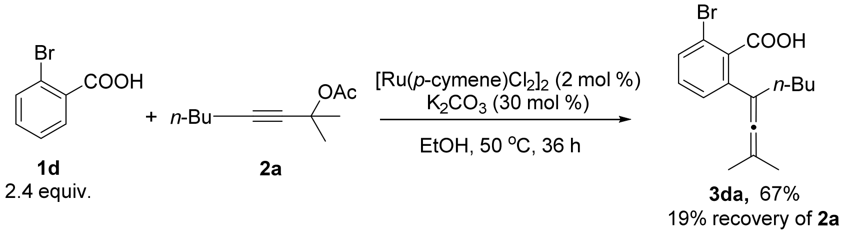

Following **Typical Procedure II**, the reaction of **1d** (482.4 mg, 2.4 mmol), **2a** (182.5 mg, 1.0 mmol), K_2CO_3 (41.5 mg, 0.3 mmol), and [Ru(p-cymene)Cl_2]$_2$ (12.3 mg, 0.02 mmol) in 2.5 mL of EtOH afforded **3da** (216.0 mg, 67%) as a solid (first round eluent: petroleum ether/ethyl acetate/AcOH = 500/30/4, 1000 mL, the impure part was further purified in second round, eluent: petroleum ether/ethyl acetate/AcOH = 500/30/4, 1000 mL): m.p. 85.2-85.3 °C (petroleum ether/DCM). 19% recovery of **2a** was determined by ^{1}H NMR analysis of the crude product using 35 μL CH_2Br_2 as the internal standard. ^{1}H NMR (300 MHz, $CDCl_3$) δ 11.15 (bs, 1 H, COOH), 7.45 (dd, J_1 = 7.8 Hz, J_2 = 1.2 Hz, 1 H, ArH), 7.28 (dd, J_1 = 8.0 Hz, J_2 = 1.4 Hz, 1 H, ArH), 7.22 (t, J = 7.5 Hz, 1 H, ArH), 2.34 (t, J = 7.2 Hz, 2 H,

CH_2), 1.75 (s, 6 H, CH_3 × 2), 1.51-1.30 (m, 4 H, CH_2 × 2), 0.91 (t, *J* = 7.2 Hz, 3 H, CH_3); ^{13}C NMR (75 MHz, $CDCl_3$) δ 201.4, 174.1, 140.1, 134.0, 130.6, 130.5, 126.5, 119.3, 101.2, 98.7, 33.0, 29.9, 22.2, 20.3, 14.0; IR (neat) *v* (cm^{-1}) 3471-2168 (COOH), 1955, 1705, 1588, 1557, 1443, 1288, 1186, 1150, 1124; MS (EI): *m/z* (%) 324 [M^+(^{81}Br), 1.85], 322 [M^+(^{79}Br), 2.45], 265 (100); Anal. Calcd. for $C_{16}H_{19}BrO_2$ (%): C, 59.45; H, 5.93; Found: C, 59.42; H, 6.00.

5. Synthesis of 2-bromo-6-(3-methylnona-3,4-dien-5-yl)benzoic acid **3dc**. (wxy-2-186, wxy-3-016)

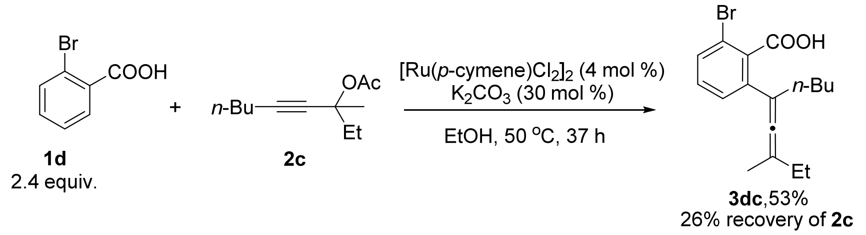

Following **Typical Procedure II**, the reaction of **1d** (478.9 mg, 2.4 mmol), **2c** (196.3 mg, 1.0 mmol), K_2CO_3 (41.5 mg, 0.3 mmol), and [Ru(*p*-cymene)Cl_2]$_2$ (24.5 mg, 0.04 mmol) in 2.5 mL of EtOH afforded **3dc** (179.9 mg, 53%) as an oil (eluent: petroleum ether/ethyl acetate = 10/1, 1000 mL). 26% recovery of **2c** was determined by ^{1}H NMR analysis of the crude product using 35 μL CH_2Br_2 as the internal standard. ^{1}H NMR (300 MHz, $CDCl_3$) δ 11.02 (bs, 1 H, COOH), 7.45 (dd, J_1 = 7.5 Hz, J_2 = 0.9 Hz, 1 H, ArH), 7.32-7.17 (m, 2 H, ArH), 2.35 (t, *J* = 7.1 Hz, 2 H, CH_2), 2.12-1.86 (m, 2 H, CH_2), 1.78 (s, 3 H, CH_3), 1.54-1.30 (m, 4 H, CH_2 × 2), 0.99 (t, *J* = 7.4 Hz, 3 H, CH_3), 0.91 (t, *J* = 6.9 Hz, 3 H, CH_3); ^{13}C NMR (75 MHz, $CDCl_3$) δ 200.6, 174.1, 140.1, 134.0, 130.52, 130.46, 126.6, 119.2, 104.7, 103.1, 33.2, 30.0, 27.2, 22.2, 18.5, 13.9, 12.1; IR (neat) *v* (cm^{-1}) 3561-2142 (COOH), 1953, 1700, 1587, 1558, 1455, 1446, 1398, 1376, 1287, 1185, 1152, 1124, 1057; MS (EI): *m/z* (%) 338

[M$^+$(^{81}Br), 7.88], 336 [M$^+$(^{79}Br), 8.92], 279 (100); HRMS Calcd for $C_{17}H_{21}O_2{}^{79}Br$ (M$^+$): 336.0725; Found: 336.0726.

6. Synthesis of 2-bromo-6-(2-methylhepta-2,3-dien-4-yl)benzoic acid **3dd**. (wxy-3-022)

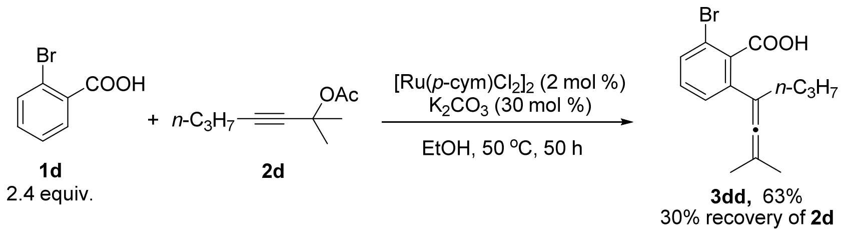

Following **Typical Procedure II**, the reaction of **1d** (481.4 mg, 2.4 mmol), **2d** (168.2 mg, 1.0 mmol), K_2CO_3 (41.4 mg, 0.3 mmol), and [Ru(p-cymene)Cl_2]$_2$ (12.5 mg, 0.02 mmol) in 2.5 mL of EtOH afforded **3dd** (195.1 mg, 63%) as a solid (eluent: petroleum ether /ethyl acetate = 15/1, 1000 mL): m.p. 95.5-99.1 ^{o}C (determined without recrystallization. Recrystallization is not possible). 30% recovery of **2d** was determined by ^{1}H NMR analysis of the crude product using 35 μL CH_2Br_2 as the internal standard. ^{1}H NMR (300 MHz, $CDCl_3$) δ 10.64 (bs, 1 H, COOH), 7.45 (dd, J_1 = 7.8 Hz, J_2 = 1.5 Hz, 1 H, ArH), 7.29 (dd, J_1 = 7.8 Hz, J_2 = 1.5 Hz, 1 H, ArH), 7.23 (t, J = 7.8 Hz, 1 H, ArH), 2.32 (t, J = 7.5 Hz, 2 H, CH_2), 1.75 (s, 6 H, CH_3 × 2), 1.55-1.40 (m, 2 H, CH_2), 0.96 (t, J = 7.4 Hz, 3 H, CH_3); ^{13}C NMR (75 MHz, $CDCl_3$) δ 201.5, 173.9, 140.1, 134.0, 130.6, 130.5, 126.5, 119.3, 101.1, 98.7, 35.4, 21.0, 20.3, 13.7; IR (neat) v (cm^{-1}) 3587-2125 (COOH), 1957, 1699, 1588, 1558, 1447, 1294, 1182, 1152, 1124; MS (EI): m/z (%) 310 [M$^+$(^{81}Br), 1.70], 308 [M$^+$(^{79}Br), 2.03], 263 (100); Anal. Calcd. for $C_{15}H_{17}BrO_2$ (%): C, 58.27; H, 5.54; Found: C, 58.38; H, 5.71.

7. Synthesis of 2-iodo-6-(2-methylocta-2,3-dien-4-yl)benzoic acid **3ea**. (wxy-2-174)

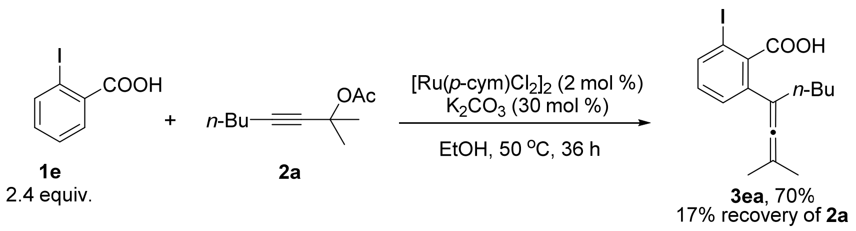

Following **Typical Procedure II**, the reaction of **1e** (595.2 mg, 2.4 mmol), **2a** (182.4 mg, 1.0 mmol), K_2CO_3 (41.5 mg, 0.3 mmol), and [Ru(*p*-cymene)Cl_2]$_2$ (12.3 mg, 0.02 mmol) in 2.5 mL of EtOH afforded **3ea** (258.7 mg, 70%) as a solid (first round eluent: petroleum ether/ethyl acetate/HOAc = 50/30/4, 1500 mL, the impure part was further purified in second round, eluent: petroleum ether/ethyl acetate/HOAc = 50/30/4, 1500 mL): m. p. 106.1-106.8 ^{o}C (petroleum ether/DCM). 17% recovery of **2a** was determined by ^{1}H NMR analysis of the crude product using 35 μL CH_2Br_2 as the internal standard. ^{1}H NMR (300 MHz, $CDCl_3$) δ 11.62 (bs, 1 H, COOH), 7.70 (d, *J* = 8.1 Hz, 1 H, ArH), 7.29 (d, *J* = 7.8 Hz, 1 H, ArH), 7.04 (t, *J* = 7.8 Hz, 1 H, ArH), 2.32 (t, *J* = 7.2 Hz, 2 H, CH_2), 1.75 (s, 6 H, CH_3 × 2), 1.51-1.30 (m, 4 H, CH_2 × 2), 0.90 (t, *J* = 7.1 Hz, 3 H, CH_3); ^{13}C NMR (75 MHz, $CDCl_3$) δ 201.1, 175.3, 139.8, 138.0, 137.2, 130.6, 127.5, 101.5, 98.3, 92.1, 33.1, 29.8, 22.1, 20.5, 14.0; IR (neat) *v* (cm^{-1}) 3500-2000 (COOH), 1699, 1584, 1551, 1443, 1395, 1299, 1184, 1146, 1395, 1299, 1184, 1146, 1122; MS (EI): *m/z* (%) 370 (M^+, 1.66), 313 (100); Anal. Calcd. for $C_{16}H_{19}IO_2$ (%): C, 51.91; H, 5.17; Found: C, 51.89; H, 5.17.

8. Synthesis of 2-iodo-6-(7-methylhexadeca-5,6-dien-5-yl)benzoic acid **3eb**. (wxy-2-179, wxy-3-017)

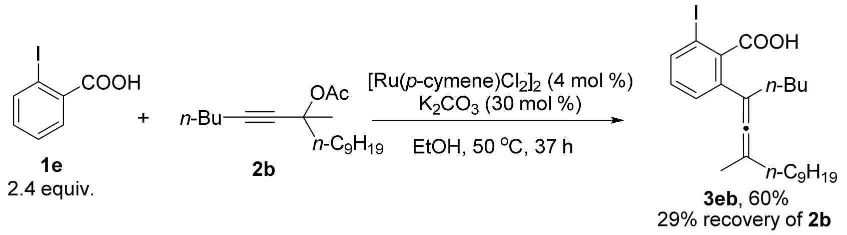

Following **Typical Procedure II**, the reaction of **1e** (595.3 mg, 2.4 mmol), **2b** (294.6 mg, 1.0 mmol), K_2CO_3 (41.5 mg, 0.3 mmol), and [Ru(*p*-cymene)Cl_2]$_2$ (24.5 mg, 0.04 mmol) in 2.5 mL of EtOH afforded **3eb** (287.5 mg, 60%) as an oil (eluent: petroleum ether/ethyl acetate = 10/1, 1000 mL). 29% recovery of **2b** was determined by ^{1}H NMR analysis of the crude product using 35 μL CH_2Br_2 as the internal standard. ^{1}H NMR (300 MHz, $CDCl_3$) δ 11.87 (bs, 1 H, COOH), 7.68 (d, J = 7.8 Hz, 1 H, ArH), 7.27 (d, J = 7.5 Hz, 1 H, ArH), 7.02 (t, J = 8.0 Hz, 1 H, ArH), 2.34 (t, J= 7.1 Hz, 2 H, CH_2), 1.98 (t, J= 7.1 Hz, 2 H, CH_2), 1.79 (s, 3 H, CH_3), 1.60-1.10 (m, 18 H, CH_2 × 9), 0.99-0.75 (m, 6 H, CH_3 × 2); ^{13}C NMR (75 MHz, $CDCl_3$) δ 200.8, 175.3, 139.9, 138.0, 137.1, 130.5, 127.5, 102.8, 102.6, 92.2, 34.0, 33.2, 31.9, 30.0, 29.6, 29.5, 29.2, 27.3, 22.6, 22.2, 18.8, 14.1, 14.0; IR (neat) v (cm^{-1}) 3550-2100 (COOH), 1953, 1704, 1581, 1551, 1456, 1378, 1286; MS (EI): *m/z* (%) 482 (M$^+$, 14.26), 425 (100); HRMS Calcd for $C_{24}H_{35}O_2I$ (M$^+$): 482.1682; Found: 482.1679.

9. Synthesis of 2-iodo-6-(3-methyl-1-phenylnona-3,4-dien-5-yl)benzoic acid **3ee**. (wxy-2-196)

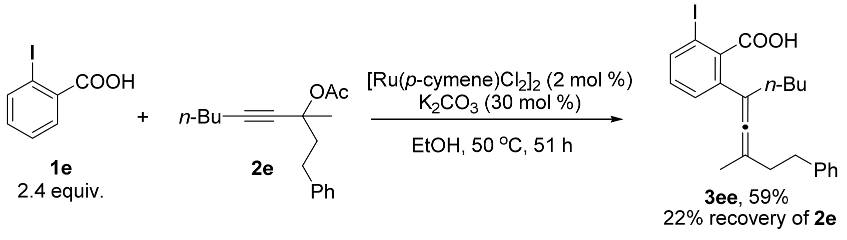

Following **Typical Procedure II**, the reaction of **1e** (593.0 mg, 2.4 mmol), **2e** (268.9 mg,

1.0 mmol), K_2CO_3 (41.8 mg, 0.3 mmol), and [Ru(*p*-cymene)Cl_2]$_2$ (12.3 mg, 0.02 mmol) in 2.5 mL of EtOH afforded **3ee** (270.1 mg, 59%) as an oil (eluent: petroleum ether/ethyl acetate /HOAc = 500/30/4，1500 mL). 22% recovery of **2e** was determined by ^{1}H NMR analysis of the crude product using 35 μL CH_2Br_2 as the internal standard. ^{1}H NMR (300 MHz, $CDCl_3$) δ 11.09 (bs, 1 H, COOH), 7.70 (dd, J_1 = 8.1 Hz, J_2 = 1.1 Hz, 1 H, ArH), 7.26-7.06 (m, 6 H, ArH), 7.02 (t, J = 7.8 Hz, 1 H, ArH), 2.80-2.61 (m, 2 H, CH_2), 2.39-2.18 (m, 4 H, CH_2 × 2), 1.82 (s, 3 H, CH_3), 1.41-1.24 (m, 4 H, CH_2 × 2), 0.88 (t, J = 7.1 Hz, 3 H, CH_3); ^{13}C NMR (75 MHz, $CDCl_3$) δ 200.9, 175.1, 141.8, 139.7, 137.9, 137.3, 130.7, 128.3, 128.1, 127.6, 125.7, 103.6, 102.1, 92.2, 35.6, 33.6, 33.2, 29.9, 22.2, 19.0, 14.0; IR (neat) *v* (cm^{-1}) 3578-2142 (COOH), 1946, 1700, 1581, 1552, 1495, 1454, 1286, 1188, 1145, 1122; MS (EI): *m/z* (%) 461 (M^++1, 1.04), 460 (M^+, 3.92), 91.2 (100); HRMS Calcd for $C_{23}H_{25}O_2I$ (M^+): 460.0899; Found: 460.0902.

10. Synthesis of 2-(2-methylocta-2,3-dien-4-yl)-6-(trifluoromethyl)benzoic acid **3fa**. (wxy-3-037)

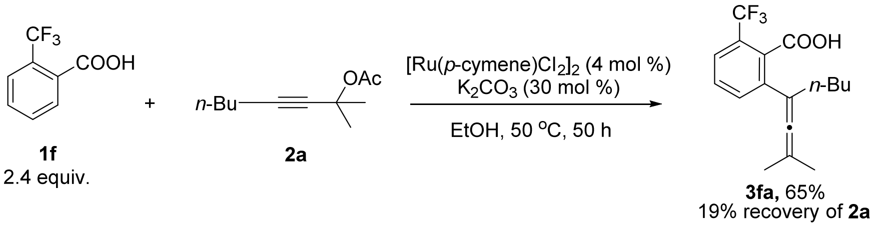

Following **Typical Procedure II**, the reaction of **1f** (456.0 mg, 2.4 mmol), **2a** (182.7 mg, 1.0 mmol), K_2CO_3 (41.7 mg, 0.3 mmol), and [Ru(*p*-cymene)Cl_2]$_2$ (24.5 mg, 0.04 mmol) in 2.5 mL of EtOH afforded **3fa** (203.9 mg, 65%) as a solid (eluent: petroleum ether/ethyl acetate = 10/1, 1300 mL): m.p. 92.0-93.0 °C (petroleum ether/DCM); 19% recovery of **2a**

was determined by ^{1}H NMR analysis of the crude product using 35 μL CH_2Br_2 as the internal standard. ^{1}H NMR (300 MHz, $CDCl_3$) δ 11.49 (bs, 1 H, COOH), 7.62-7.46 (m, 3 H, ArH), 2.36 (t, J = 7.2 Hz, 2 H, CH_2), 1.76 (s, 6 H, CH_3 × 2), 1.55-1.35 (m, 4 H, CH_2 × 2), 0.94 (t, J = 7.1 Hz, 3 H, CH_3); ^{13}C NMR (75 MHz, $CDCl_3$) δ 201.1, 174.2, 140.0, 131.9, 130.4 (q, J = 2.1 Hz), 129.5, 127.7 (q, J = 31.7 Hz), 124.1 (q, J = 4.8 Hz), 123.5 (q, J = 287.7 Hz), 101.0, 98.4, 33.7, 29.8, 22.2, 20.1, 13.9; ^{19}F NMR (282 MHz, $CDCl_3$) δ -59.7; IR (neat) ν (cm^{-1}) 3557-2155 (COOH), 1961, 1714, 1700, 1597, 1583, 1464, 1398, 1363, 1319, 1291, 1190, 1169, 1138, 1066; MS (EI): *m/z* (%) 312 (M^+, 3.00), 251 (100); Anal. Calcd. for $C_{17}H_{19}F_3O_2$ (%): C, 65.37; H, 6.13; Found: C, 65.34; H, 6.15.

11. Synthesis of 2-(2-methylocta-2,3-dien-4-yl)-6-(trifluoromethoxy)benzoic acid **3ga**. (wxy-3-036)

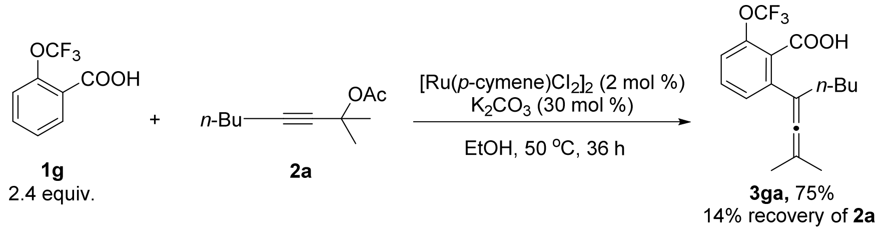

Following **Typical Procedure II**, the reaction of **1g** (494.5 mg, 2.4 mmol), **2a** (182.3 mg, 1.0 mmol), K_2CO_3 (41.9 mg, 0.3 mmol), and [Ru(p-cymene)Cl_2]$_2$ (12.4 mg, 0.02 mmol) in 2.5 mL of EtOH afforded **3ga** (247.5 mg, 75%) as a solid (eluent: petroleum ether/ethyl acetate = 10/1, 1200 mL): m.p. 83.9-84.3 °C (petroleum ether/DCM); 14% recovery of **2a** was determined by ^{1}H NMR analysis of the crude product using 35 μL CH_2Br_2 as the internal standard. ^{1}H NMR (300 MHz, $CDCl_3$) δ 11.93 (bs, 1 H, COOH), 7.39 (t, J = 8.1 Hz, 1 H, ArH), 7.28 (d, J = 8.1 Hz, 1 H, ArH), 7.17 (d, J = 8.1 Hz, 1 H, ArH), 2.38 (t, J = 7.1 Hz, 2 H,

CH_2), 1.75 (s, 6 H, $CH_3 \times 2$), 1.53-1.31 (m, 4 H, $CH_2 \times 2$), 0.92 (t, J= 7.1 Hz, 3 H, CH_3); ^{13}C NMR (75 MHz, $CDCl_3$) δ 201.9, 172.9, 146.1 (q, J= 1.4 Hz), 140.5, 130.5, 125.8, 125.5, 120.5 (q, J= 257.4 Hz), 117.6 (q, J= 1.4 Hz), 100.8, 99.2, 32.5, 29.9, 22.2, 19.8, 13.9; ^{19}F NMR (282 MHz, $CDCl_3$) δ -57.3 (s, 3 F); IR (neat) ν (cm^{-1}) 3566-2146 (COOH), 1957, 1714, 1699, 1604, 1575, 1464, 1456, 1404, 1363, 1259, 1214, 1168, 1133, 1065, 1029; MS (EI): *m/z* (%) 328 (M^+, 8.21), 271 (100); Anal. Calcd. for $C_{17}H_{19}F_3O_3$ (%): C, 62.19; H, 5.83; Found: C, 62.07; H, 5.89.

12. Synthesis of 2-(7-methyldodeca-5,6-dien-5-yl)-6-(trifluoromethoxy)benzoic acid **3gf**. (wxy-3-048)

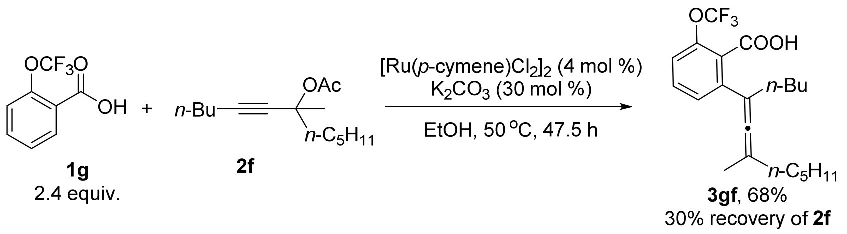

Following **Typical Procedure II**, the reaction of **1g** (494.8 mg, 2.4 mmol), **2f** (238.1 mg, 1.0 mmol), K_2CO_3 (41.7 mg, 0.3 mmol), and [Ru(*p*-cymene)Cl_2]$_2$ (24.6 mg, 0.04 mmol) in 2.5 mL of EtOH afforded **3gf** (262.1 mg, 68%) as an oil (eluent: petroleum ether/ethyl acetate = 9/1, 1000 mL); 30% recovery of **2f** was determined by ^{1}H NMR analysis of the crude product using 35 μL CH_2Br_2 as the internal standard. ^{1}H NMR (300 MHz, $CDCl_3$) δ 12.16 (bs, 1 H, COOH), 7.39 (t, J= 8.0 Hz, 1 H, ArH), 7.27 (d, J= 7.8 Hz, 1 H, ArH), 7.17 (d, J= 8.1 Hz, 1 H, ArH), 2.46-2.25 (m, 2 H, CH_2), 1.98 (t, J= 7.4 Hz, 2 H, CH_2), 1.76 (s, 3 H, CH_3), 1.55-1.30 (m, 6 H, $CH_2 \times 3$), 1.30-1.11 (m, 4 H, $CH_2 \times 2$), 0.91 (t, J= 7.2 Hz, 3 H, CH_3), 0.81 (t, J= 6.8 Hz, 3 H, CH_3); ^{13}C NMR (75 MHz, $CDCl_3$) δ 201.6, 172.8, 146.1 (q, J = 1.4 Hz),

140.8, 130.5, 125.8, 125.7, 120.5 (q, J = 257.2 Hz), 117.7 (q, J = 1.4 Hz), 103.3, 102.2, 33.9, 32.7, 31.4, 30.1, 27.0, 22.5, 22.2, 17.9, 13.92, 13.89; ^{19}F NMR (282 MHz, $CDCl_3$) δ -57.4; IR (neat) ν (cm^{-1}) 3550-2100 (COOH), 1953, 1714, 1700, 1604, 1575, 1467, 1404, 1259, 1216, 1171, 1133, 1064; MS (EI): *m/z* (%) 384 (M^+, 33.82), 385 (M^+ + 1, 7.98), 327 (100); HRMS Calcd for $C_{21}H_{27}O_3F_3$ (M^+): 384.1912; Found: 384.1914.

13. Synthesis of 2-methoxy-6-(7-methylhexadeca-5,6-dien-5-yl)benzoic acid **3hb**. (wxy-2-181, wxy-3-015)

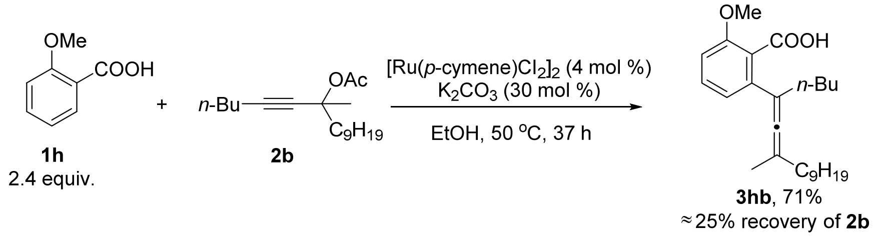

Following **Typical Procedure II**, the reaction of **1h** (365.5 mg, 2.4 mmol), **2b** (294.4 mg, 1.0 mmol), K_2CO_3 (41.5 mg, 0.3 mmol), and [Ru(*p*-cymene)Cl_2]$_2$ (24.5 mg, 0.04 mmol) in 2.5 mL of EtOH afforded **3hb** (274.5 mg, 71%) as an oil (eluent: petroleum ether/ethyl acetate /HOAc = 500/50/2, 1000 mL). About 25% recovery of **2b** was determined by ^{1}H NMR analysis of the crude product using 35 μL CH_2Br_2 as the internal standard. ^{1}H NMR (300 MHz, $CDCl_3$) δ 11.98 (bs, 1 H, COOH), 7.30 (t, J= 8.1 Hz, 1 H, ArH), 6.95 (d, J = 7.5 Hz, 1 H, ArH), 6.79 (d, J = 8.4 Hz, 1 H, ArH), 3.83 (s, 3 H, OMe), 2.37 (t, J= 7.2 Hz, 2 H, CH_2), 2.08-1.89 (m, 2 H, CH_2), 1.79 (s, 3 H, CH_3), 1.51-1.31 (m, 6 H, CH_2 × 3), 1.31-1.14 (m, 12 H, CH_2 × 6), 0.95-0.80 (m, 6 H, CH_3 × 2); ^{13}C NMR (75 MHz, $CDCl_3$) δ 201.2, 174.7, 156.4, 138.7, 130.2, 122.1, 119.5, 108.9, 102.8, 102.4, 55.8, 34.0, 32.7, 31.9, 30.2, 29.6, 29.5, 29.32, 29.27, 27.4, 22.6, 22.3, 18.1, 14.05, 13.95; IR (neat) ν (cm^{-1}) 3523-2138 (COOH),

1949, 1699, 1595, 1580, 1471, 1296, 1267, 1126, 1089, 1066; MS (EI): *m/z* (%) 386 (M^+, 25.55), 329 (100); HRMS Calcd for $C_{25}H_{38}O_3$ (M^+): 386.2821; Found: 386.2823.

14. Synthesis of 2-(2-methylocta-2,3-dien-4-yl)-6-phenoxybenzoic acid **3ia**. (wxy-3-011)

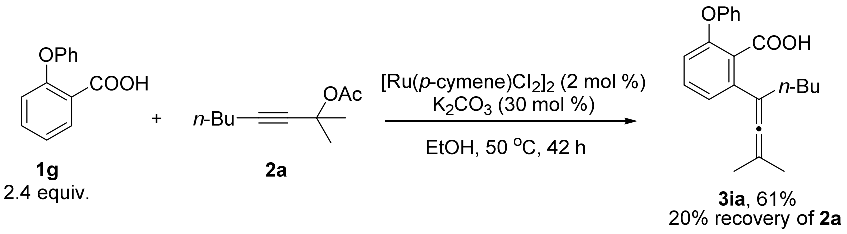

Following **Typical Procedure II**, the reaction of **1i** (514.2 mg, 2.4 mmol), **2a** (182.2 mg, 1.0 mmol), K_2CO_3 (42.0 mg, 0.3 mmol), and [Ru(*p*-cymene)Cl_2]$_2$ (12.5 mg, 0.02 mmol) in 2.5 mL of EtOH afforded **3ia** (203.8 mg, 61%) as a solid (eluent: petroleum ether/ethyl acetate = 10/1, 1000 mL): m.p. 126.8-127.1 ^{o}C (petroleum ether/DCM). 20% recovery of **2a** was determined by ^{1}H NMR analysis of the crude product using 35 μL CH_2Br_2 as the internal standard. ^{1}H NMR (300 MHz, $CDCl_3$) δ 12.29 (bs, 1 H, COOH), 7.35-7.25 (m, 2 H, ArH), 7.21 (t, *J* = 8.0 Hz, 1 H, ArH), 7.13-6.95 (m, 4 H, ArH), 6.66 (d, *J* = 8.1 Hz, 1 H, ArH), 2.36 (t, *J* = 7.1 Hz, 2 H, CH_2), 1.60 (s, 6 H, CH_3 × 2), 1.50-1.25 (m, 4 H, CH_2 × 2), 0.88 (t, *J* = 7.1 Hz, 3 H, CH_3); ^{13}C NMR (75 MHz, $CDCl_3$) δ 202.1, 174.5, 156.9, 154.7, 138.6, 130.2, 129.7, 124.6, 123.7, 121.3, 119.6, 115.8, 100.6, 99.5, 32.1, 30.0, 22.2, 19.7, 14.0; IR (neat) *v* (cm^{-1}) 3540-2258 (COOH), 1953, 1703, 1595, 1575, 1490, 1456, 1294, 1257, 1211, 1162, 1128, 1066; MS (EI): *m/z* (%) 336 (M^+, 1.34); 279 (100); Anal. Calcd. for $C_{22}H_{24}O_3$ (%): C, 78.54; H, 7.19; Found: C, 78.55; H, 7.10.

15. Synthesis of 2,3-dichloro-6-(2-methylocta-2,3-dien-4-yl)benzoic acid **3ja**. (wxy-2-182)

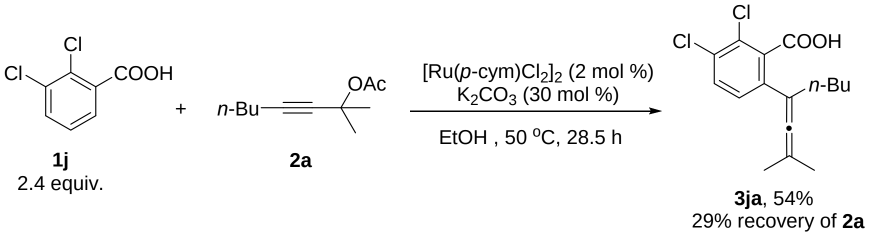

Following **Typical Procedure II**, the reaction of **1j** (458.4 mg, 2.4 mmol), **2a** (182.5 mg, 1.0 mmol), K_2CO_3 (41.7 mg, 0.3 mmol), and [Ru(*p*-cymene)Cl_2]$_2$ (12.2 mg, 0.02 mmol) in 2.5 mL of EtOH afforded **3ja** (203.5 mg, 54%) as a solid (first round eluent: petroleum ether/ethyl acetate/HOAc = 500/30/4, 1500 mL; The impure part was further purified in second round, eluent: petroleum ether/ethyl acetate/HOAc = 500/40/4, 1500 mL): m.p. 103.1-103.9 ^{o}C, (petroleum ether/DCM). 29% recovery of **2a** was determined by ^{1}H NMR analysis of the crude product using 35 μL CH_2Br_2 as the internal standard. ^{1}H NMR (300 MHz, $CDCl_3$) δ 11.58 (bs, 1 H, COOH), 7.46 (d, *J* = 8.4 Hz, 1 H, ArH), 7.20 (d, *J* = 8.7 Hz, 1 H, ArH), 2.33 (t, *J* = 7.2 Hz, 2 H, CH_2), 1.75 (s, 6 H, CH_3 × 2), 1.50-1.30 (m, 4 H, CH_2 × 2), 0.91 (t, *J* = 7.1 Hz, 3 H, CH_3); ^{13}C NMR (75 MHz, $CDCl_3$) δ 201.6, 172.9, 137.9, 133.6, 131.1, 130.9, 129.2, 126.7, 100.4, 99.3, 32.7, 29.8, 22.1, 20.1, 13.9; IR (KBr) *ν* (cm^{-1}) 3351-2129 (COOH), 1955, 1704, 1549, 1458, 1412, 1375, 1362, 1276, 1251, 1180, 1059, 1025; MS (EI): *m/z* (%) 316 [M^+($^{37}Cl^{37}Cl$), 0.43], 314 [M^+($^{37}Cl^{35}Cl$), 1.61], 312 [M^+($^{35}Cl^{35}Cl$), 1.94], 255 (100); Anal. Calcd. for $C_{16}H_{18}Cl_2O_2$ (%): C, 61.36; H, 5.79; Found: C, 61.26; H, 5.81.

16. Synthesis of 2-methyl-6-(7-methyldodeca-5,6-dien-5-yl)-3-nitrobenzoic acid **3kf**. (wxy-3-152)

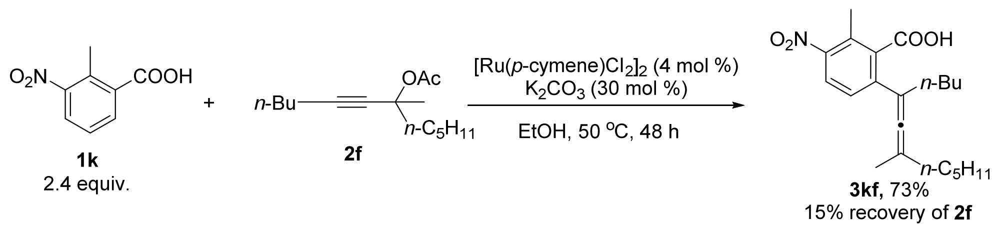

Following **Typical Procedure II**, the reaction of **1k** (434.9 mg, 2.4 mmol), **2a** (238.0 mg, 1.0 mmol), K_2CO_3 (41.9 mg, 0.3 mmol), and [Ru(*p*-cymene)Cl_2]$_2$ (24.5 mg, 0.04 mmol) in 2.5 mL of EtOH afforded **3kf** (260.1 mg, 73%) as an oil (eluent: petroleum ether/ethyl acetate = 9/1, 1500 mL); 15% recovery of **2a** was determined by ^{1}H NMR analysis of the crude product using 35 μL CH_2Br_2 as the internal standard. ^{1}H NMR (400 MHz, $CDCl_3$) δ 11.89 (bs, 1 H, COOH), 7.91 (d, *J* = 8.4 Hz, 1 H, ArH), 7.34 (d, *J* = 8.8 Hz, 1 H, ArH), 2.57 (s, 3 H, CH_3), 2.46-2.31 (m, 2 H, CH_2), 2.00 (t, *J* = 8.0 Hz, 2 H, CH_2), 1.77 (s, 3 H, CH_3), 1.53-1.33 (m, 6 H, CH_2 × 3), 1.33-1.18 (m, 4 H, CH_2 × 2), 0.92 (t, *J* = 7.2 Hz, 3 H, CH_3), 0.83 (t, *J* = 6.8 Hz, 3 H, CH_3); ^{13}C NMR (100 MHz, $CDCl_3$) δ 201.3, 174.4, 148.3, 143.0, 134.8, 130.0, 126.2, 125.3, 103.2, 102.5, 33.8, 32.9, 31.3, 30.0, 26.9, 22.4, 22.1, 18.2, 16.5, 13.9, 13.8; IR (neat) *v* (cm^{-1}) 3578-2138 (COOH), 1951, 1704, 1592, 1580, 1525, 1463, 1347, 1281, 1131; MS (EI): *m/z* (%) 360 (M^+ + 1, 57.36), 359 (M^+, 82.16), 41 (100); HRMS Calcd for $C_{21}H_{30}NO_4$ (M+H)$^+$: 360.2175; Found: 360.2169.

17. Synthesis of 2-methyl-6-(2-methyldodeca-2,3-dien-4-yl)-3-nitrobenzoic acid **3kg**. (wxy-3-023)

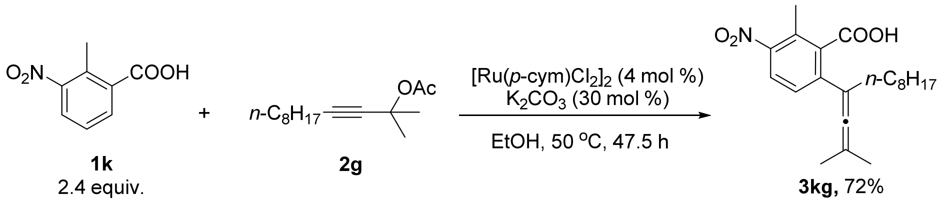

Following **Typical Procedure II**, the reaction of **1k** (434.7 mg, 2.4 mmol), **2g** (238.9 mg,

1.0 mmol), K_2CO_3 (41.5 mg, 0.3 mmol), and [Ru(*p*-cymene)Cl_2]$_2$ (24.5 mg, 0.04 mmol) in 2.5 mL of EtOH afforded **3kg** (258.8 mg, 72%) as an oil (eluent: petroleum ether/ethyl acetate/AcOH = 450/50/2, 1300 mL); ^{1}H NMR (300 MHz, $CDCl_3$) δ 10.78 (bs, 1 H, COOH), 7.91 (d, *J* = 8.7 Hz, 1 H, ArH), 7.34 (d, *J* = 8.7 Hz, 1 H, ArH), 2.55 (s, 3 H, CH_3), 2.37 (t, *J* = 7.1 Hz, 2 H, CH_2), 1.75 (s, 6 H, CH_3 × 2), 1.52-1.18 (m, 12 H, CH_2 × 6), 0.87 (t, *J* = 6.6 Hz, 3 H, CH_3); ^{13}C NMR (75 MHz, $CDCl_3$) δ 201.7, 174.2, 148.3, 142.9, 134.9, 130.1, 126.1, 125.3, 101.4, 98.9, 33.1, 31.8, 29.4, 29.3, 29.1, 27.7, 22.6, 20.0, 16.5, 14.0; IR (neat) *v* (cm^{-1}) 3712-2116 (COOH), 1955, 1704, 1700, 1593, 1581, 1525, 1520, 1348, 1279, 1130; MS (EI): *m/z* (%) 360 (M^+ + 1, 18.98), 359 (M^+, 73.54), 43 (100); HRMS Calcd for $C_{21}H_{29}NO_4$ (M^+): 359.2097; Found: 359.2098.

18. Synthesis of 4-bromo-2-chloro-6-(7-methyldodeca-5,6-dien-5-yl)benzoic acid **3lf**. (wxy-3-153)

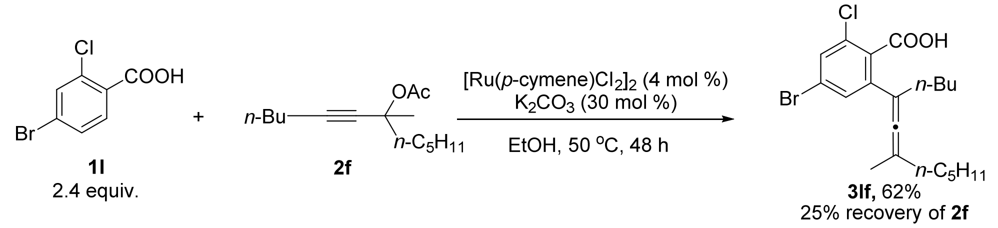

Following **Typical Procedure II**, the reaction of **1l** (564.0 mg, 2.4 mmol), **2f** (240.0 mg, 1.0 mmol), K_2CO_3 (41.8 mg, 0.3 mmol), and [Ru(*p*-cymene)Cl_2]$_2$ (24.6 mg, 0.04 mmol) in 5.0 mL of EtOH afforded **3lf** (256.9 mg, 62%) as an oil (eluent: petroleum ether/ethyl acetate = 10/1, 1500 mL); 25% recovery of **2f** was determined by ^{1}H NMR analysis of the crude product using 35 μL CH_2Br_2 as the internal standard. ^{1}H NMR (400 MHz, $CDCl_3$) δ 11.98 (bs, 1 H, COOH), 7.45 (d, *J* = 1.6 Hz, 1 H, ArH), 7.39 (d, *J* = 1.6 Hz, 1 H, ArH), 2.40-2.25 (m, 2

H, CH_2), 2.06-1.90 (m, 2 H, CH_2), 1.75 (s, 3 H, CH_3), 1.50-1.30 (m, 6 H, $CH_2 \times 3$), 1.30-1.17 (m, 4 H, $CH_2 \times 2$), 0.91 (t, J = 7.0 Hz, 3 H, CH_3), 0.83 (t, J = 7.0 Hz, 3 H, CH_3); ^{13}C NMR (100 MHz, $CDCl_3$) δ 201.6, 173.2, 141.5, 131.8, 130.7, 129.8, 128.9, 123.7, 103.9, 101.6, 33.9, 32.6, 31.4, 30.0, 26.9, 22.5, 22.2, 18.2, 14.0, 13.9; IR (neat) ν (cm^{-1}) 3561-2194 (COOH), 1952, 1708, 1573, 1548, 1456, 1397, 1367, 1285, 1186, 1133; MS (EI): *m/z* (%) 416 [M^+(^{81}Br^{37}Cl), 6.24], 414 [M^+(^{81}Br^{35}Cl) and/or M^+(^{79}Br^{37}Cl), 24.07], 412 [M^+(^{79}Br^{35}Cl), 20.38], 41 (100); HRMS Calcd for $C_{20}H_{27}{}^{79}Br^{35}ClO_2$ $(M+H)^+$: 413.0883; Found: 413.0877.

19. Synthesis of 3-(2-methylocta-2,3-dien-4-yl)-2-naphthoic acid **3ma**. (wxy-2-134, wxy-2-139)

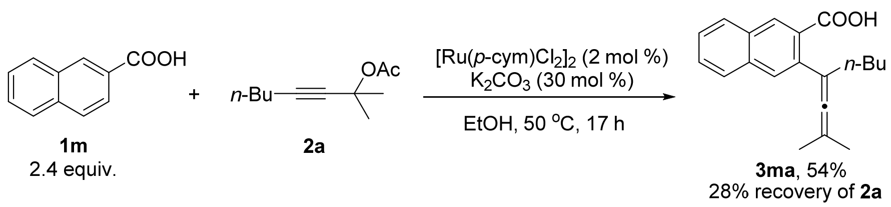

Following **Typical Procedure II**, the reaction of **1m** (413.2 mg, 2.4 mmol), K_2CO_3 (41.5 mg, 0.3 mmol), **2a** (182.3 mg, 1.0 mmol), and [Ru(*p*-cymene)Cl_2]$_2$ (12.5 mg, 0.02 mmol) in 5.0 mL of EtOH afforded **3ma** (157.8 mg, 54%) (eluent: petroleum ether/ethyl acetate/HOAc = 500/40/4, 1500 mL): oil; 28% recovery of **2a** was determined by ^{1}H NMR analysis of the crude product using 35 μL CH_2Br_2 as the internal standard. ^{1}H NMR (300 MHz, $CDCl_3$) δ 11.33 (bs, 1 H, COOH), 8.39 (s, 1 H, ArH), 7.88 (d, J = 8.1 Hz, 1 H, ArH), 7.82 (d, J = 8.1 Hz, 1 H, ArH), 7.76 (s, 1 H, ArH), 7.55 (td, J_1 = 7.4 Hz, J_2 = 1.3 Hz, 1 H, ArH), 7.48 (td, J_1 = 7.5 Hz, J_2 = 1.3 Hz, 1 H, ArH), 2.45 (t, J = 7.2 Hz, 2 H, CH_2), 1.76 (s, 6 H, $2 \times CH_3$), 1.60-1.39 (m, 4 H, $2 \times CH_2$), 0.94 (t, J = 7.2 Hz, 3 H, CH_3); ^{13}C NMR (75 MHz, $CDCl_3$) δ 201.5, 174.7, 137.2, 134.8, 131.5, 131.1, 128.6, 128.4, 128.2, 127.9, 127.5, 126.3, 103.4, 97.1, 33.4, 30.2,

22.3, 20.2, 14.1; IR (neat) *v* (cm^{-1}) 3617-2090 (COOH), 1953, 1699, 1695, 1682, 1629, 1590, 1464, 1447, 1404, 1361, 1286, 1214, 1138, 1082; MS (EI): m/z (%) = 294 (M^+, 1.41), 237 (100); HRMS Calcd for $C_{20}H_{22}O_2$ (M^+): 294.1620; Found: 294.1618.

20. Synthesis of 3-(6-methyl-8-phenylocta-4,5-dien-4-yl)-2-naphthoic acid **3me**. (wxy-1-197)

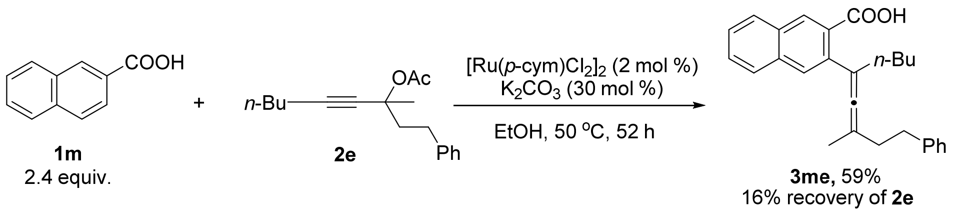

Following **Typical Procedure II**, the reaction of **1m** (413.6 mg, 2.4 mmol), **2e** (272.0 mg, 1.0 mmol), K_2CO_3 (41.6 mg, 0.3 mmol), and [Ru(*p*-cymene)Cl_2]$_2$ (12.5 mg, 0.02 mmol) in 5.0 mL of EtOH afforded **3me** (228.3 mg, 59%) as an oil (eluent: petroleum ether/ethyl acetate/AcOH = 500/40/4, 1200 mL); 16% recovery of **2e** was determined by ^{1}H NMR analysis of the crude product using 35 μL CH_2Br_2 as the internal standard. ^{1}H NMR (300 MHz, $CDCl_3$) δ 11.54 (bs, 1 H, COOH), 8.39 (s, 1 H, ArH), 7.84 (d, *J* = 8.1 Hz, 1 H, ArH), 7.79 (d, *J* = 8.1 Hz, 1 H, ArH), 7.71 (s, 1 H, ArH), 7.53 (t, *J* = 7.4 Hz, 1 H, ArH), 7.45 (t, *J* = 7.4 Hz, 1 H, ArH), 7.24-7.09 (m, 4 H, ArH), 7.09-6.97 (m, 1 H, ArH), 2.88-2.68 (m, 2 H, CH_2), 2.48-2.25 (m, 4 H, CH_2× 2), 1.82 (s, 3 H, CH_3), 1.55-1.31 (m, 4 H, CH_2 × 2), 0.92 (t, *J* = 6.9 Hz, 3 H, CH_3); ^{13}C NMR (75 MHz, $CDCl_3$) δ 200.8, 174.4, 142.2, 137.3, 134.9, 131.8, 131.2, 128.7, 128.28, 128.25, 128.1, 127.9, 127.5, 126.3, 125.6, 105.8, 100.9, 35.9, 34.0, 33.8, 30.3, 22.4, 18.8, 14.1; IR (neat) *v* (cm^{-1}) 3300-2100 (COOH), 1951, 1703, 1699, 1695, 1683, 1629, 1496, 1454, 1404, 1287, 1214, 1138; MS (EI): *m/z* (%) 384 (M^+, 2.34), 131 (100); HRMS Calcd for $C_{27}H_{28}O_2$ (M^+): 384.2089; Found: 384.2087.

21. Synthesis of 4-bromo-2-(2-methylocta-2,3-dien-4-yl)benzoic acid **3na** 4-bromo-2,6-bis(2-methylocta-2,3-dien-4-yl)benzoic acid **4na**. (wxy-2-135)

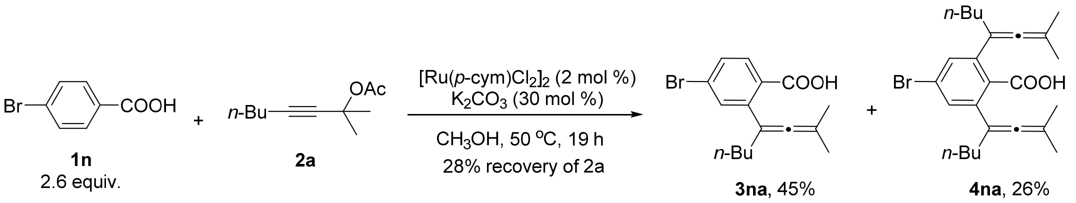

Following **Typical Procedure II**, the reaction of **1n** (522.5 mg, 2.6 mmol), **2a** (182.3 mg, 1.0 mmol), K_2CO_3 (41.9 mg, 0.3 mmol), and [Ru(*p*-cymene)Cl_2]$_2$ (12.4 mg, 0.02 mmol) in 2.5 mL of MeOH afforded **3na** (144.7 mg, 45%) and **4na** (57.0 mg, 26%) (eluent: petroleum ether/ethyl acetate/HOAc = 500/40/4, 2000 mL). 28% recovery of **2a** was determined by ^{1}H NMR analysis of the crude product using 35 μL CH_2Br_2 as the internal standard.

3na: solid; m.p. 69.9-71.9 °C (petroleum ether/DCM); ^{1}H NMR (300 MHz, $CDCl_3$) δ 11.21 (bs, 1 H, COOH), 7.69 (d, J = 8.1 Hz, 1 H, ArH), 7.47 (s, 1 H, ArH), 7.42 (dd, J_1 = 8.1 Hz, J_2 = 1.8 Hz, 1 H, ArH), 2.29 (t, J = 7.1 Hz, 2 H, CH_2), 1.71 (s, 6 H, CH_3 × 2), 1.50-1.30 (m, 4 H, CH_2 × 2), 0.91 (t, J = 7.1 Hz, 3 H, CH_3); ^{13}C NMR (75 MHz, $CDCl_3$) δ 200.9, 173.6, 143.8, 132.6, 131.8, 129.5, 128.2, 126.6, 102.7, 97.7, 33.2, 30.1, 22.2, 20.0, 14.0; IR (neat) *ν* (cm^{-1}) 3583-2181 (COOH), 1957, 1699, 1583, 1557, 1416, 1362, 1296, 1141, 1100; MS (EI): *m/z* (%) 324 [M$^+$(^{81}Br), 0.75], 322 [M$^+$(^{79}Br), 0.83], 265 (100); Anal. Calcd. for $C_{16}H_{19}BrO_2$ (%): C, 59.45; H, 5.93; Found: C, 59.41; H, 5.91.

4na: solid; m.p. 107.9-109.7 °C (petroleum ether/DCM); ^{1}H NMR (300 MHz, $CDCl_3$) δ 7.27 (s, 2 H, ArH), 2.25 (t, J = 7.1 Hz, 4 H, CH_2 × 2), 1.70 (s, 12 H, CH_3 × 4), 1.50-1.28 (m, 8 H, CH_2 × 4), 0.90 (t, J = 7.1 Hz, 6 H, CH_3 × 2); ^{13}C NMR (75 MHz, $CDCl_3$) δ 200.8, 173.8, 140.5, 130.5, 129.6, 123.1, 101.4, 97.6, 33.7, 29.9, 22.2, 20.4, 14.0; IR (neat) *ν* (cm^{-1})

3484-2198 (COOH), 1963, 1704, 1564, 1444, 1362, 1282, 1130; MS (EI): *m/z* (%) 446 [M^+(^{81}Br), 55.06], 444 [M^+(^{79}Br), 49.23], 41 (100); Anal. Calcd. for $C_{25}H_{33}BrO_2$ (%): C, 67.41; H, 7.47; Found: C, 67.06; H, 7.39.

22. Synthesis of 2-bromo-6-(2-methyldodeca-2,3-dien-4-yl)benzoic acid **3dg**. (wxy-3-061)

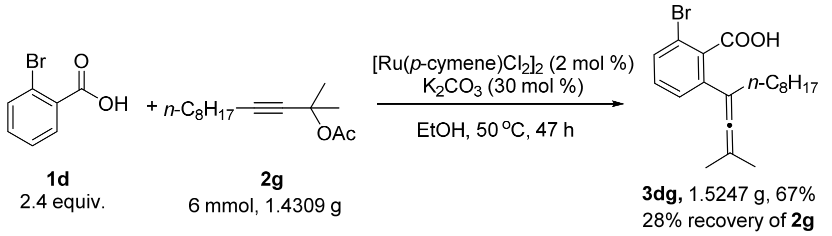

Following **Typical Procedure II**, the reaction of **1d** (2.8936 g, 14.4 mmol), **2g** (1.4309 g, 6.0 mmol), K_2CO_3 (248.9 mg, 1.8 mmol), and [Ru(*p*-cymene)Cl_2]$_2$ (73.5 mg, 0.12 mmol) in 15 mL of EtOH afforded **3dg** (1.5247 g, 67%) as an oil (first round eluent: petroleum ether/ethyl acetate/HOAc = 500/50/4, 2000 mL; The impure part was further purified in second round, eluent: petroleum ether/ethyl acetate/HOAc = 500/50/4, 1500 mL). 28% recovery of **2g** was determined by ^{1}H NMR analysis of the crude product using 210 μL CH_2Br_2 as the internal standard. ^{1}H NMR (300 MHz, $CDCl_3$) δ 9.57 (bs, 1 H, COOH), 7.46 (dd, J_1 = 7.7 Hz, J_2 = 1.4 Hz, 1 H, ArH), 7.29 (dd, J_1 = 7.8 Hz, J_2 = 1.5 Hz, 1 H, ArH), 7.23 (t, J = 7.8 Hz, 1 H, ArH), 2.33 (t, J = 7.1 Hz, 2 H, CH_2), 1.75 (s, 6 H, CH_3 × 2), 1.51-1.15 (m, 12 H, CH_2 × 6), 0.87 (t, J = 6.6 Hz, 3 H, CH_3); ^{13}C NMR (75 MHz, $CDCl_3$) δ 201.4, 173.7, 140.1, 133.9, 130.6, 130.5, 126.5, 119.3, 101.3, 98.7, 33.3, 31.9, 29.5, 29.3, 29.2, 27.8, 22.6, 20.3, 14.1; IR (neat) ν (cm^{-1}) 3557-2159 (COOH), 1958, 1706, 1587, 1557, 1443, 1385, 1361, 1287, 1188, 1152, 1126, 1056; MS (EI): *m/z* (%) 380 [M(^{81}Br)$^+$, 46.19], 378 [M(^{79}Br)$^+$, 49.29], 43 (100); HRMS Calcd for $C_{20}H_{27}O_2{}^{79}Br$ (M^+): 378.1194; Found: 378.1193.

23. Synthesis of (S_a)-2-(7-methyldodeca-5,6-dien-5-yl)-6-(trifluoromethoxy)benzoic acid (S_a)-**3gf**. (wxy-3-156)

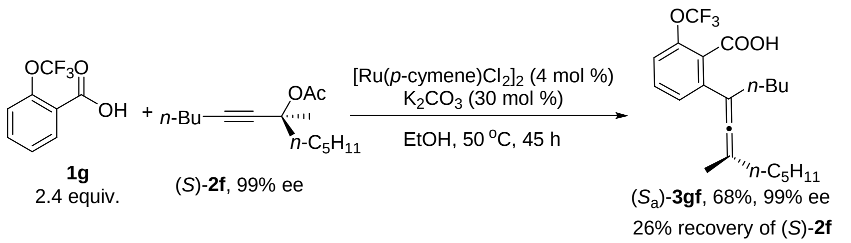

Following **Typical Procedure II**, the reaction of **1g** (148.7 mg, 0.72 mmol), (*S*)-**2f** (71.1 mg, 0.3 mmol, 99% ee), K_2CO_3 (12.3 mg, 0.09 mmol), and [Ru(*p*-cymene)Cl_2]$_2$ (7.5 mg, 0.012 mmol) in 0.8 mL of EtOH afforded (S_a)-**3gf** (78.1 mg, 68%) as an oil (eluent: petroleum ether/ethyl acetate = 10/1, 1000 mL): 99% ee (HPLC conditions: Chiralcel OJ-3 column, CO_2/*i*-PrOH = 98/2, 1.0 mL/min, λ = 254 nm, t_R(major) = 1.34 min, t_R(minor) = 1.55 min); $[\alpha]_D^{20}$ = + 126.3 (*c* = 1.225, $CHCl_3$); 26% recovery of (*S*)-**2f** was determined by ^{1}H NMR analysis of the crude product using 10.5 μL CH_2Br_2 as the internal standard. ^{1}H NMR (400 MHz, $CDCl_3$) δ 11.33 (bs, 1 H, COOH), 7.40 (t, *J* = 8.0 Hz, 1 H, ArH), 7.27 (d, *J* = 7.6 Hz, 1 H, ArH), 7.17 (d, *J* = 8.4 Hz, 1 H, ArH), 2.45-2.28 (m, 2 H, CH_2), 2.05-1.88 (m, 2 H, CH_2), 1.75 (s, 3 H, CH_3), 1.52-1.30 (m, 6 H, CH_2 × 3), 1.30-1.15 (m, 4 H, CH_2 × 2), 0.91 (t, *J* = 7.2 Hz, 3 H, CH_3), 0.81 (t, *J* = 6.8 Hz, 3 H, CH_3); ^{13}C NMR (100 MHz, $CDCl_3$) δ 201.5, 172.6, 146.1, 140.8, 130.5, 125.7, 120.4 (q, *J* = 257.3 Hz), 117.69, 117.65, 103.3, 102.2, 33.9, 32.7, 31.4, 30.1, 26.9, 22.5, 22.2, 17.9, 13.95, 13.92; ^{19}F NMR (376 MHz, $CDCl_3$) δ -57.4; IR (neat) *v* (cm^{-1}) 3467-2151 (COOH), 1952, 1709, 1604, 1575, 1467, 1403, 1258, 1215, 1171, 1064; MS (EI): *m/z* (%) 384 (M^+, 8.24), 323 (100); HRMS Calcd for $C_{21}H_{28}F_3O_3$ $(M+H)^+$: 385.1991; Found: 385.1985.

24. Synthesis of (S_a)-2-methyl-6-(7-methyldodeca-5,6-dien-5-yl)-3-nitrobenzoic acid (S_a)-**3kf**. (wxy-3-157)

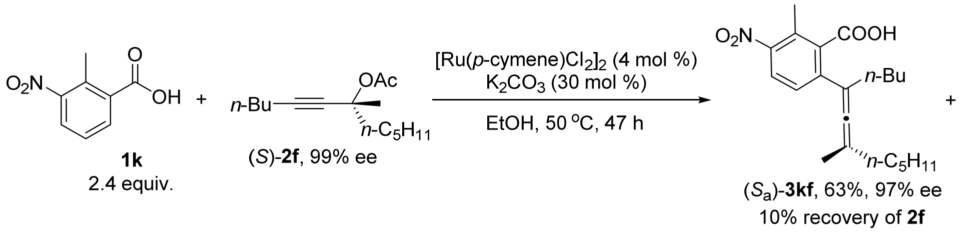

Following **Typical Procedure II**, the reaction of **1k** (130.5 mg, 0.72 mmol), (*S*)-**2f** (71.0 mg, 0.3 mmol, 99% ee), K_2CO_3 (12.5 mg, 0.09 mmol), and [Ru(*p*-cymene)Cl_2]$_2$ (7.3 mg, 0.012 mmol) in 0.8 mL of EtOH afforded (S_a)-**3kf** (67.4 mg, 63%) as an oil (eluent: petroleum ether/ethyl acetate = 9/1, 1500 mL); 97% ee (HPLC conditions: Chiralcel OZ-H column, *n*-hexane/*i*-PrOH = 100/1, 1.0 mL/min, λ = 214 nm, t_R(major) = 38.5 min, t_R(minor) = 27.7 min); $[\alpha]_D^{20}$ = + 69.5 (*c* = 0.85, $CHCl_3$); 10% recovery of (*S*)-**2f** was determined by ^{1}H NMR analysis of the crude product using 10.5 μL CH_2Br_2 as the internal standard. ^{1}H NMR (300 MHz, $CDCl_3$) δ 11.53 (bs, 1 H, COOH), 7.91 (t, *J* = 8.4 Hz, 1 H, ArH), 7.33 (d, *J* = 8.7 Hz, 1 H, ArH), 2.56 (s, 3 H, CH_3), 2.46-2.28 (m, 2 H, CH_2), 1.99 (t, *J* = 7.2 Hz, 2 H, CH_2), 1.76 (s, 3 H, CH_3), 1.53-1.32 (m, 6 H, CH_2 × 3), 1.32-1.12 (m, 4 H, CH_2 × 2), 0.92 (t, *J* = 7.1 Hz, 3 H, CH_3), 0.83 (t, *J* = 6.9 Hz, 3 H, CH_3); ^{13}C NMR (75 MHz, $CDCl_3$) δ 201.3, 174.3, 148.4, 143.1, 134.8, 130.1, 126.3, 125.3, 103.2, 102.5, 33.9, 33.0, 31.4, 30.0, 26.9, 22.4, 22.2, 18.2, 16.5, 13.94, 13.88; IR (neat) *v* (cm^{-1}) 3561-2129 (COOH), 1953, 1742, 1704, 1592, 1581, 1525, 1465, 1347, 1280, 1131; MS (EI): *m/z* (%) 359 (M^+, 4.60), 302 (100); HRMS Calcd for $C_{21}H_{30}NO_4$ (M + H)$^+$: 360.2175; Found: 360.2171.

Synthetic applications

25. Synthesis of 7-bromo-3-(2-methylprop-1-en-1-yl)-3-octylisobenzofuran-1(3*H*)-one **5dg**. (fjj-1-015)

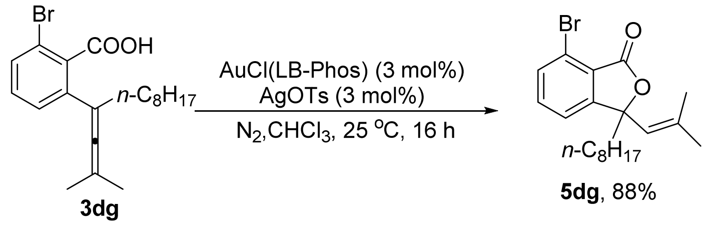

To a dry Schlenk tube were added AgOTs (4.3 mg, 0.015 mmol, weighed in a glove box, 98%), AuCl(LB-Phos) (9.0 mg, 0.015 mmol), and $CHCl_3$ (1.5 mL) under nitrogen atmosphere sequentially. After stirring for 15 min at 25 °C, **3dg** (190.0 mg, 0.5 mmol) and $CHCl_3$ (1 mL) were added. After being continuously stirred at 25 °C for 16 h, the reaction was complete as monitored by TLC. After filtration through a short column of silica gel (eluent: DCM, 10 mL × 3) and evaporation, the crude mixture was purified by column chromatography on silica gel afforded **5dg** (167.3 mg, 88%) (eluent: petroleum ether /ethyl acetate = 200/1, 1500 mL) as an oil: ^{1}H NMR (300MHz, $CDCl_3$) δ 7.64 (d, *J* = 7.8 Hz, 1 H, ArH), 7.50 (t, *J* = 7.7 Hz, 1 H, ArH), 7.32 (d, *J* = 7.8 Hz, 1 H, ArH), 5.42 (s, 1 H, =CH), 2.18-2.03 (m, 1 H, one proton from CH_2), 1.92-1.78 (m, 1 H, one proton from CH_2), 1.74 (s, 3 H, CH_3), 1.60 (s, 3 H, CH_3), 1.40-1.07 (m, 11 H, CH_2 × 5 and one proton from CH_2), 1.04-0.89 (m, 1 H, one proton from CH_2), 0.85 (t, *J* = 6.6 Hz, 3 H, CH_3); ^{13}C NMR (75 MHz, $CDCl_3$) δ 167.7, 156.4, 139.7, 135.1, 133.4, 124.2, 122.5, 120.6, 120.5, 87.0, 41.2, 31.8, 29.4, 29.3, 29.1, 27.4, 23.1, 22.6, 19.1, 14.1; IR (neat) ν (cm^{-1}) 3075, 2926, 2855, 1770, 1668, 1597, 1583, 1462, 1376, 1322, 1235, 1129, 1091, 1045; MS (EI): *m/z* (%) 380 [M(^{81}Br)$^+$, 0.73], 378 [M(^{79}Br)$^+$, 0.62], 265 (100). Anal. Calcd. for $C_{20}H_{27}BrO_2$ (%): C, 63.33; H, 7.17;

Found: C, 63.41; H, 7.22.

26. Iodolactonization reaction of **3dg** with iodine to afford **6dg.** (wxy-3-066)

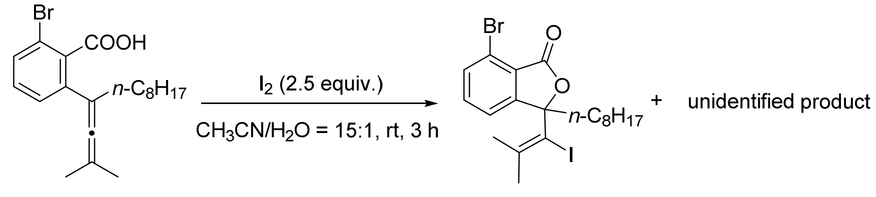

To a dried Schlenk tube were added **3dg** (189.7 mg, 0.5 mmol), CH_3CN (2.5 mL), I_2 (317.0 mg, 1.25 mmol), and H_2O (165 μL) sequentially at rt. After being stirred for 3 h at rt, the reaction was complete as monitored by TLC. A saturated aqueous solution of $Na_2S_2O_3$ (3 mL) and 3 mL of ethyl acetate were added. The organic phase was separated and the aqueous phase was extracted with ethyl acetate (2×5 mL). The combined organic layer was evaporated and purification by flash column chromatography on silica gel [(eluent: petroleum/ethyl acetate = 60/1 (500 mL) to petrpleum/ethyl acetate = 50/1 (200 mL), then petrpleum/ethyl acetate = 10/1 (300 mL)] afforded **6dg** (179.2 mg, 71%, 98% purity) and an unidentified product (18.3 mg).

6dg: oil; ^{1}H NMR (300 MHz, $CDCl_3$) δ 7.92 (d, J = 7.8 Hz, 1 H, ArH), 7.68 (d, J = 7.8 Hz, 1 H, ArH), 7.51 (t, J = 7.8 Hz, 1 H, ArH), 2.70-2.54 (m, 1 H, one proton from CH_2), 2.10 (s, 3 H, CH_3), 2.05 (s, 3 H, CH_3), 2.15-1.93 (m, 1 H, one proton from CH_2), 1.49-1.00 (m, 12 H, $CH_2 \times 6$), 0.86 (t, J = 6.6 Hz, 3 H, CH_3); ^{13}C NMR (75 MHz, $CDCl_3$) δ 166.7, 154.8, 143.2, 134.4, 133.9, 123.7, 123.4, 120.2, 97.9, 89.5, 42.4, 37.0, 31.6, 29.2, 29.1, 29.0, 23.5, 22.8, 22.5, 14.0; IR (neat) ν (cm^{-1}) 3080, 2955, 2926, 2854, 1777, 1770, 1595, 1581, 1460, 1430, 1377, 1367, 1321, 1234, 1181, 1130, 1096, 1046; MS (EI): m/z (%) 506 [M(^{81}Br)$^+$, 1.91], 504 [M(^{79}Br)$^+$, 1.50], 345 (100); HRMS Calcd for $C_{20}H_{26}O_2BrI$ (M^+): 504.0161; Found: 504.0163.

Mechanism studies

(a) H/D exchange experiment. (wxy-4-090)

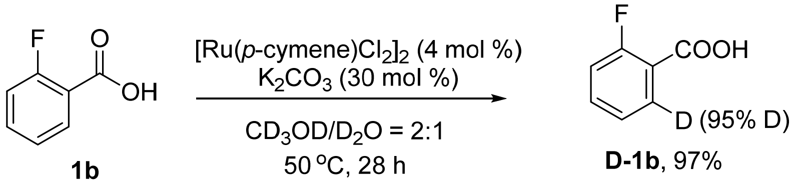

To a dried Schlenk tube were sequentially added 2-fluorobenzoic acid **1b** (420.5 mg, 3.0 mmol), [Ru(*p*-cymene)Cl_2]$_2$ (73.7 mg, 0.12 mmol), and K_2CO_3 (124.6 mg, 0.9 mmol) in open air atmosphere. After being evacuated and backfilled with nitrogen three times, 1.5 mL of CD_3OD and 0.75 mL of D_2O was added. Then, the reaction tube was put into an oil bath preheated to 50 ^{o}C. After 28 h, 10 mL of HCl (2 M) was added, and extracted with ethyl acetate (20 mL × 2). After concentration in vacuo, the crude residual was directly purified by chromatography on silica gel (eluent: petroleum ether/ethyl acetate = 3/1, 300 mL) to afford **D-1b** as a white solid (410.6 mg, 97%, 95% deuterium): m.p. 123.5-123.7 ^{o}C (petroleum ether/diethyl ether); ^{1}H NMR (300 MHz, $CDCl_3$) δ 11.78 (bs, 1 H, ArH), 7.60 (td, J_1 = 8.0 Hz, J_2 = 5.0 Hz, 1 H, ArH), 7.34-7.14 (m, 2 H, ArH), the following signal is disscernible for **1b**: δ 8.06 (td, J_1 = 7.7 Hz, J_2 = 1.8 Hz, 0.05 H, ArH); ^{13}C NMR (75 MHz, $CDCl_3$) δ 170.1, 162.6 (d, J = 260.6 Hz), 135.6 (d, J = 9.7 Hz), 132.5 (t, J = 25.2 Hz), 124.96 (d, J = 3.5 Hz), 117.3, 117.0, the following signals is disscernible for **1b**: δ 132.7, 124.03 (d, J = 4.1 Hz); ^{19}F NMR (282 MHz, $CDCl_3$) δ 108.8, the following signal is disscernible for **1b**: δ 108.7; IR (neat) ν (cm^{-1}) 3600-2047 (COOH), 1695, 1609, 1461, 1412, 1300; MS (EI): *m/z* (%) 141 (M^{+}, 80.37), 124 (100); HRMS Calcd for $C_7H_4DFO_2$ (M^{+}): 141.0336; Found: 141.0337.

(b) Kinetic isotope effect studies: (wxy-4-088A, wxy-4-091)

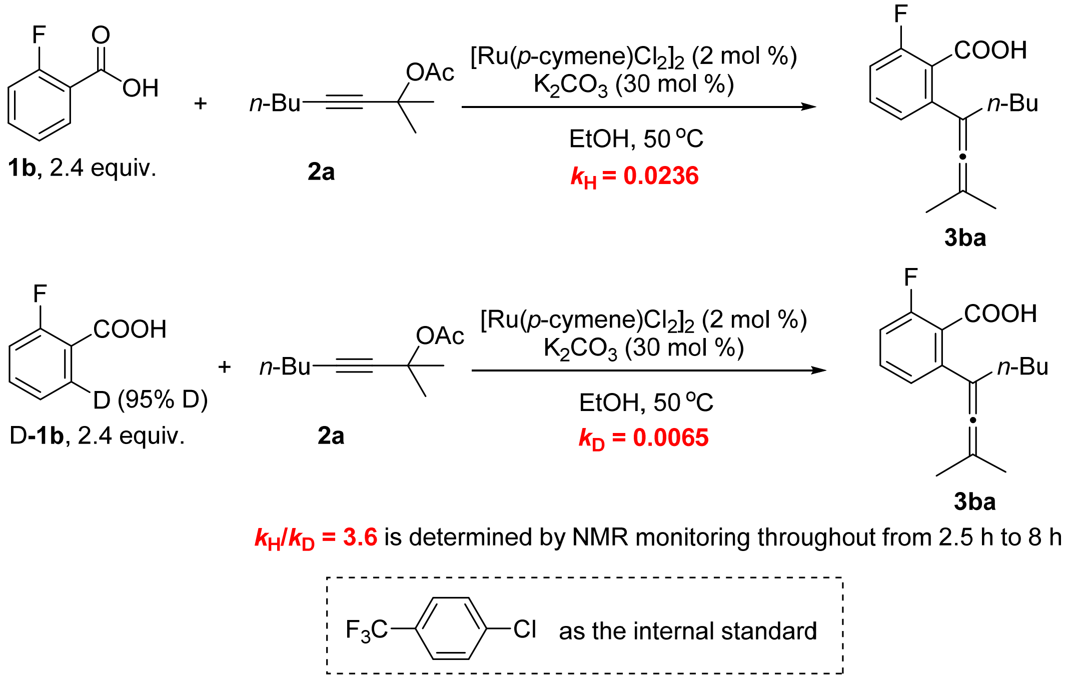

To a dried Schlenk tube were added **1b** (168.2 mg, 1.2 mmol), [Ru(*p*-cymene)Cl_2]$_2$ (6.1 mg, 0.01 mmol), K_2CO_3 (20.7 mg, 0.15 mmol), **2a** (92.0 mg, 0.5 mmol)/EtOH (1.25 mL), 1-chloro-4-(trifluoromethyl)benzene (22.0 μL, *d* = 1.353 g/mL, 29.8mg, 0.165 mmol) sequentially at rt. The reaction tube was put into an oil bath preheated to 50 °C. An aliquot of the resulting mixture was taken for ^{19}F NMR analysis every 30 mins.

In another dried Schlenk tube, the reaction of **D-1b** (169.3 mg, 1.2 mmol), [Ru(*p*-cymene)Cl_2]$_2$ (6.1 mg, 0.01 mmol), K_2CO_3 (20.7 mg, 0.15 mmol), **2a** (91.2 mg, 0.5 mmol), EtOH (1.25 mL) and 1-chloro-4-(trifluoromethyl)benzene (22.0 μL) was conducted at the same scale. The reaction mixture was treated with the same procedure above, an aliquot of the resulting mixture was taken for ^{19}F NMR analysis every 30 mins. After being stirred for 11 h, the reaction residual was concentrated in vacuo and directly purified by chromatography to recover **D-1b** on silica gel (eluent: petroleum ether/ethyl acetate = 20/1, 1000 mL). 14.8 mg of purified **D-1b** was obtained, the deuterium content of D-**1b** was

decreased slightly (93% deuterium). ^{1}H NMR (300 MHz, $CDCl_3$) δ 11.27 (bs, 1 H, ArH),), 7.60 (td, J_1 = 7.8 Hz, J_2 = 4.6 Hz, 1 H, ArH), 7.32-7.12 (m, 2 H, ArH), the following signal is disscernible for **1b**: δ 8.05 (t, J = 7.7 Hz, 0.07 H, ArH); ^{13}C NMR (75 MHz, $CDCl_3$) δ 169.8, 162.7 (d, J = 260.6 Hz), 135.7 (d, J = 9.7 Hz), 132.5 (t, J = 24.8 Hz), 124.0 (d, J = 4.1 Hz), 117.3, 117.1, the following signals is disscernible for **1b**: δ 132.8, 124.1 (d, J = 4.1 Hz); ^{19}F NMR (282 MHz, $CDCl_3$) δ 108.73, the following signal is disscernible for **1b**: δ 108.67.

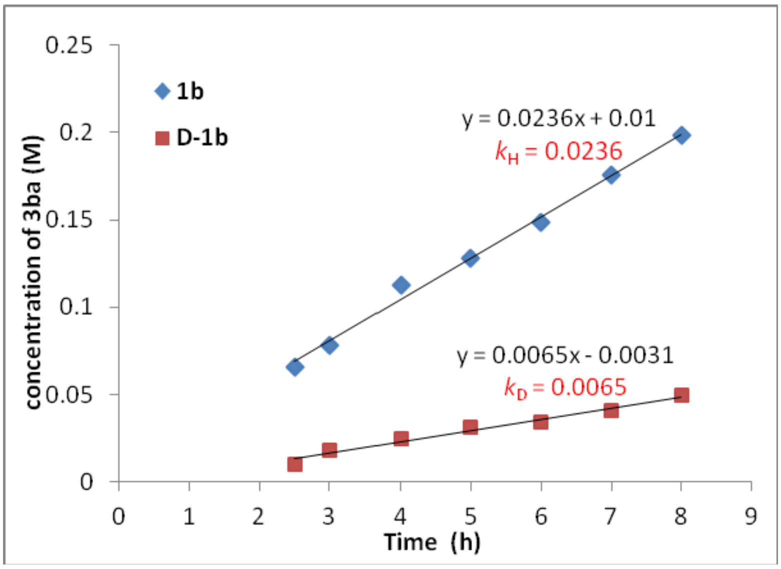

Figure S1. Plot of the concentrations of **3ba** over time.

The NMR yield and concentration of **3ba** over time are listed blew:

time (h)	**1b was** used as substrate		[D]-**1b was** used as substrate	
	NMR yield of **3ba** (%)	[**3ba**] (M)	NMR yield of **3ba** (%)	[**3ba**] (M)
2.5	16.5	0.066	2.6	0.0104
3	19.6	0.0784	4.5	0.018
4	28.2	0.1128	6.1	0.0244
5	32.1	0.1284	7.8	0.0312
6	37.2	0.1488	8.5	0.034
7	43.9	0.1756	10.3	0.0412
8	49.6	0.1984	12.4	0.0496

(c) Determination of the order for 2-fluorobenzoic acid **1b.** (wxy-3-182)

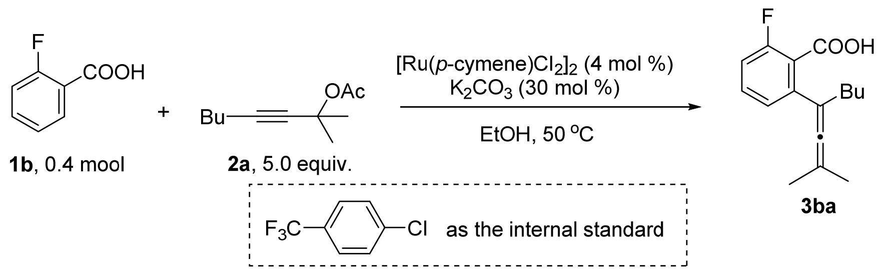

To a dried Schlenk tube were added 2-fluorobenzoic acid **1b** (56.1 mg, 0.4 mmol), [Ru(*p*-cymene)Cl_2]$_2$ (9.9 mg, 0.016 mmol), K_2CO_3 (16.7 mg, 0.12 mmol), **2a** (365.0 mg, 2.0 mmol)/EtOH (1 mL), and 1-chloro-4-(trifluoromethyl)benzene (18 μL, *d* = 1.353 g/mL, 24.4 mg, 0.135 mmol) sequentially at rt. Then, the reaction tube was put into an oil bath preheated to 50 °C. An aliquot of the resulting mixture was taken for ^{19}F NMR analysis every 40 mins.

Time (min)	Recovery of **1b** (%)	[**1b**] (M)	ln([**1b**])
40	69.1	0.2764	-1.28591
80	60.5	0.242	-1.41882
120	52.5	0.21	-1.56065
160	46.8	0.1872	-1.67558
200	41.4	0.1656	-1.79818
240	37.2	0.1488	-1.90515
280	34.0	0.136	-1.9951
320	30.8	0.1232	-2.09395

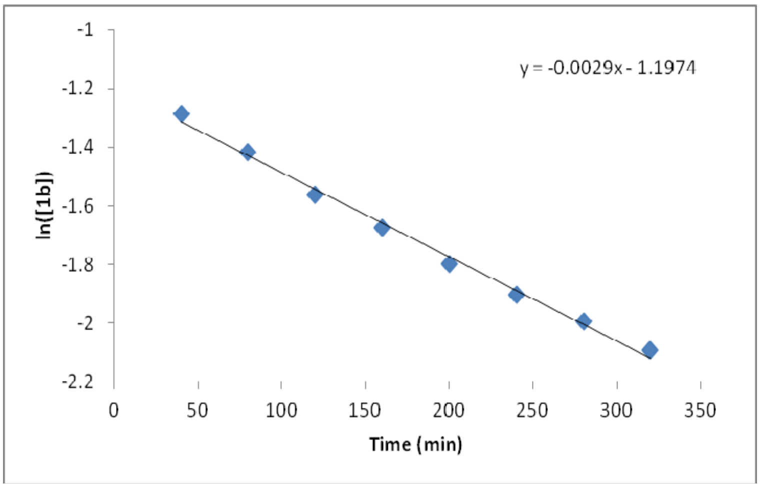

Figure S2. A first-order dependence of initial rate on **1b**.

(d) Determination of the order for propargylic acetate **2a.** (wxy-3-185)

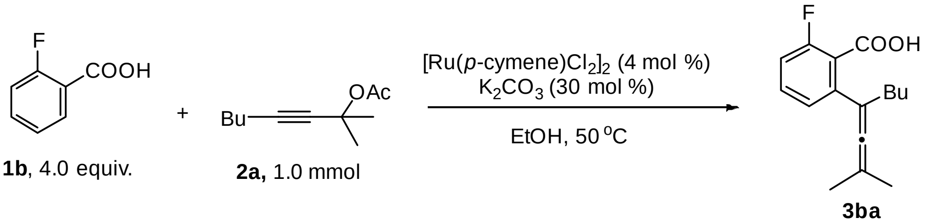

Seven parallel experiments were carried out following the procedure below:

To a dried Schlenk tube were added 2-fluorobenzoic acid **1b** (112.1 mg, 0.8 mmol), [Ru(*p*-cymene)Cl_2]$_2$ (4.9 mg, 0.008 mmol), K_2CO_3 (8.3 mg, 0.06 mmol), and **2a** (36.5 mg, 0.2 mmol)/EtOH (0.5 mL) sequentially at rt. The reaction tube was put into an oil bath preheated to 50 °C. After being stirred for corresponding reaction time, the reaction mixture was filtrated through a short column of silica gel eluted with ethyl acetate (20 mL × 2) and concentration in vacuo. To the reaction residue was added 7 μL of CH_2Br_2 and analyzed with ^{1}H NMR measurement.

	Time (min)	Recovery of **2a** (%)	[**2a**] (M)	ln([**2a**])
wxy-3-185-G	30	85.9	0.3434	-1.06886
wxy-3-185-F	60	80.6	0.3224	-1.13196
wxy-3-185-E	90	76.4	0.3056	-1.18548
wxy-3-185-D	120	69.8	0.2794	-1.27511
wxy-3-185-C	150	67.8	0.271	-1.30564
wxy-3-185-B	180	64.1	0.2564	-1.36102
wxy-3-185-A	210	58.5	0.234	-1.45243

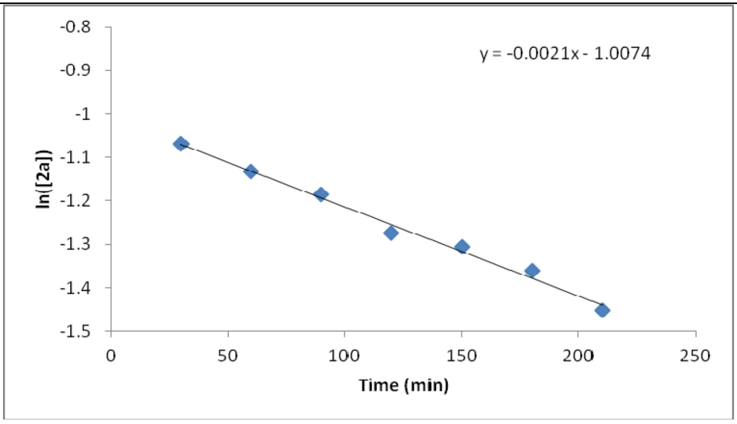

Figure S3. A first-order dependence of initial rate on **2a**.

(e) the dependency of the reaction rate on concentration of the ruthenium catalyst. (wxy-4-088A, wxy-4-088B, wxy-4-089A, wxy-4-089B)

Following **Typical Procedure II**, Four experiments with different concentration of $[Ru(p\text{-cymene})Cl_2]_2$ were carried out. The usage amount of starting material are listed blew:

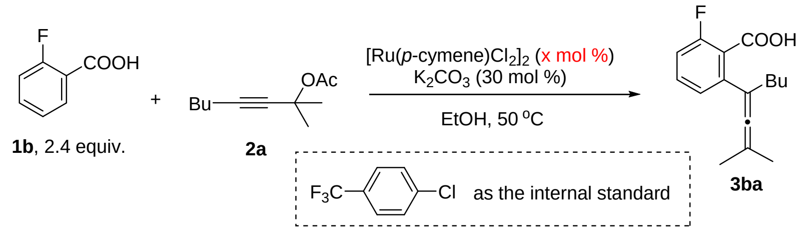

	$[Ru(p\text{-cymene})Cl_2]_2$	**2a** (1.0 equiv.)	**1b** (2.4 equiv.)	K_2CO_3 (30 mol%)	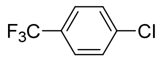	EtOH
2 mol% cat. (WXY-4-088A)	6.1 mg, 0.01 mmol	92.0 mg, 0.5 mmol	168.2 mg, 1.2 mmol	20.7 mg, 0.15 mmol	22.0 µL, 0.165 mmol	1.25 mL
3 mol% cat. (WXY-4-088B)	9.2 mg, 0.015 mmol	92.0 mg, 0.5 mmol	168.2 mg, 1.2 mmol	20.7 mg, 0.15 mmol	22.0 µL, 0.165 mmol	1.25 mL
4 mol% cat. (WXY-4-089A)	12.2 mg, 0.02 mmol	91.3 mg, 0.5 mmol	168.1 mg, 1.2 mmol	20.7 mg, 0.15 mmol	22.0 µL, 0.165 mmol	1.25 mL
6 mol% cat. (WXY-4-089B)	18.4 mg, 0.03 mmol	91.2 mg, 0.5 mmol	168.1 mg, 1.2 mmol	20.8 mg, 0.15 mmol	22.0 µL, 0.165 mmol	1.25 mL

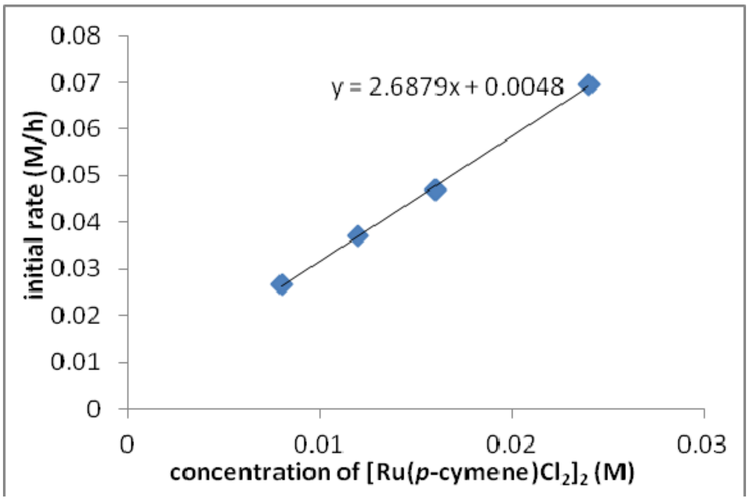

Figure S4. A first-order dependence of initial rate on the amount of $[Ru(p\text{-cymene})Cl_2]_2$.

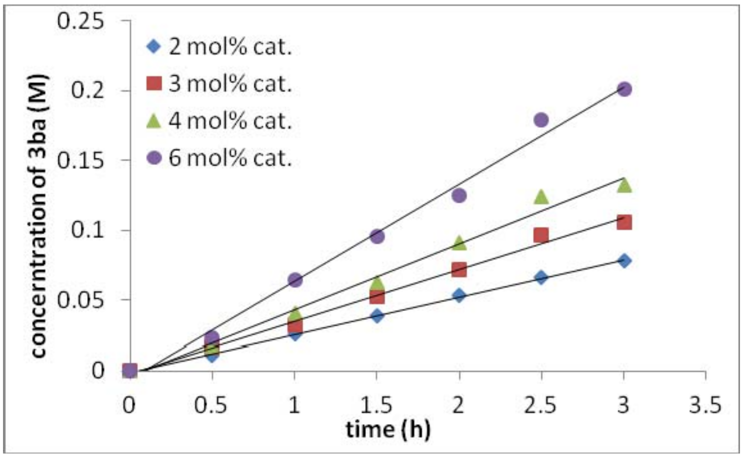

Figure S5. Plot of the concentrations of **3ba** over time with four different initial concentrations of [Ru(p-cymene)Cl_2]$_2$.

The relevant data are listed below:

time (h)	NMR yield of **3ba** (%)			
	(2 mol% cat.) Wxy-4-088A	(3 mol% cat.) Wxy-4-088B	(4 mol% cat.) WXY-4-089A	(6 mol% cat.) WXY-4-089B
0.5	2.7	4.1	4.4	5.8
1	6.5	8.1	10.1	16.1
1.5	9.6	13	15.6	23.9
2	13.3	17.9	22.8	31.2
2.5	16.5	24.2	31	44.7
3	19.6	26.5	33	50.2

(f) the dependency of the reaction rate with different molar ratio of benzoic acid **1b** and propargylic acetate **2a**. (wxy-4-088A, wxy-4-093, wxy-4-113,wxy-4-094)

Following **Typical Procedure II**, Four experiments with different molar ratio of benzoic acid **1b** and propargylic acetate **2a** were carried out. The usage amount of starting material are listed blew:

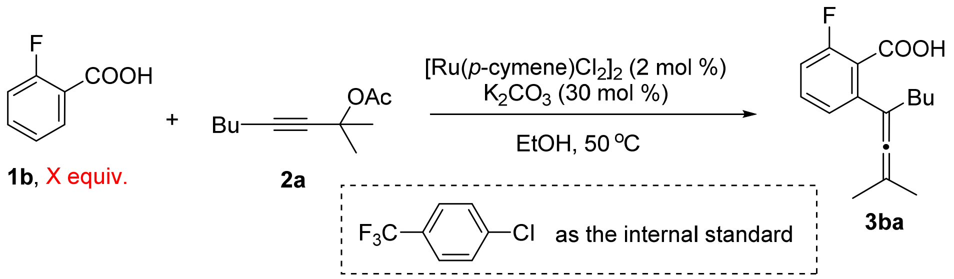

	[Ru(*p*-cymene)Cl_2]$_2$	**2a** (1.0 equiv.)	**1b** (2.4 equiv.)	K_2CO_3 (30 mol%)	F_3C–C_6H_4–Cl	EtOH
**1b**:**2a** = 2.4:1 (WXY-4-088A)	6.1 mg, 0.01 mmol	92.0 mg, 0.5 mmol	168.2 mg, 1.2 mmol	20.7 mg, 0.15 mmol	22.0 μL, 0.165 mmol	1.25 mL
**1b**:**2a** = 1.5:1 (WXY-4-093)	6.2 mg, 0.01 mmol	91.1 mg, 0.5 mmol	105.1 mg, 0.75 mmol	20.9 mg, 0.15 mmol	22.0 μL, 0.165 mmol	1.25 mL
**1b**:**2a** = 1:1 (WXY-4-113)	6.1 mg, 0.01 mmol	91.4 mg, 0.5 mmol	70.1 mg, 0.5 mmol	20.7 mg, 0.15 mmol	22.0 μL, 0.165 mmol	1.25 mL
**1b**:**2a** = 1:1.5 (WXY-4-094)	6.1 mg, 0.01 mmol	136.8 mg, 0.75 mmol	70.1 mg, 0.5 mmol	20.5 mg, 0.15 mmol	22.0 μL, 0.165 mmol	1.25 mL

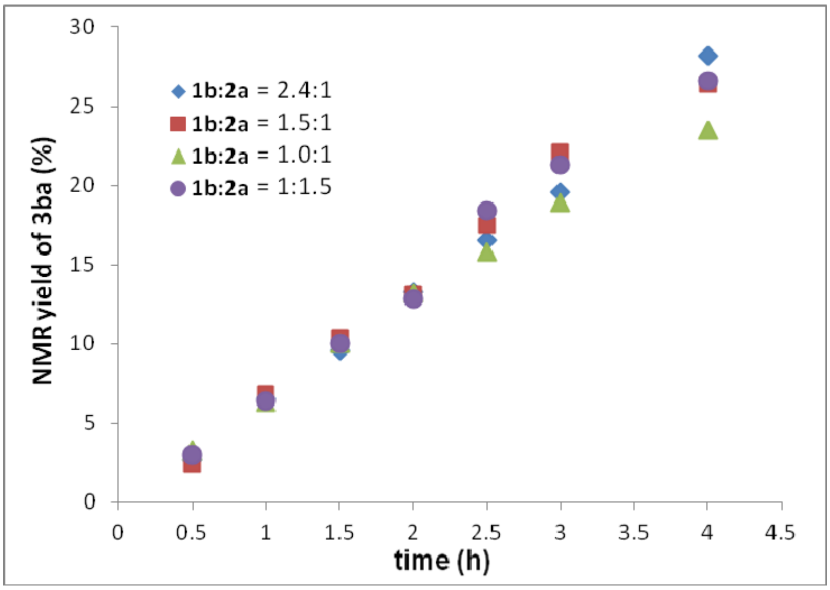

Figure S6. NMR yield of **3ba** vs. time depending on the molar ratio of **1b** and **2a**.

The experimental data are as follows:

time (h)	NMR yield of **3ba (%)**			
	**1b**:**2a** = 2.4:1	**1b**:**2a** = 1.5:1	**1b**:**2a** = 1:1	**1b**:**2a** = 1:1.5
0.5	2.7	2.4	3.3	3
1	6.5	6.8	6.3	6.4
1.5	9.6	10.4	10	10
2	13.3	13.1	13.2	12.8
2.5	16.5	17.5	15.8	18.4
3	19.6	22.1	18.9	21.3
4	28.2	26.4	23.5	26.6

References：

[1] page 186-190, Ph. D. dissertation, S, Wu. Zhejiang University, **2010**.

[2] W, Zhang.; S. Ma. *Chem. Commun.,* 2018, **54**, 6064.

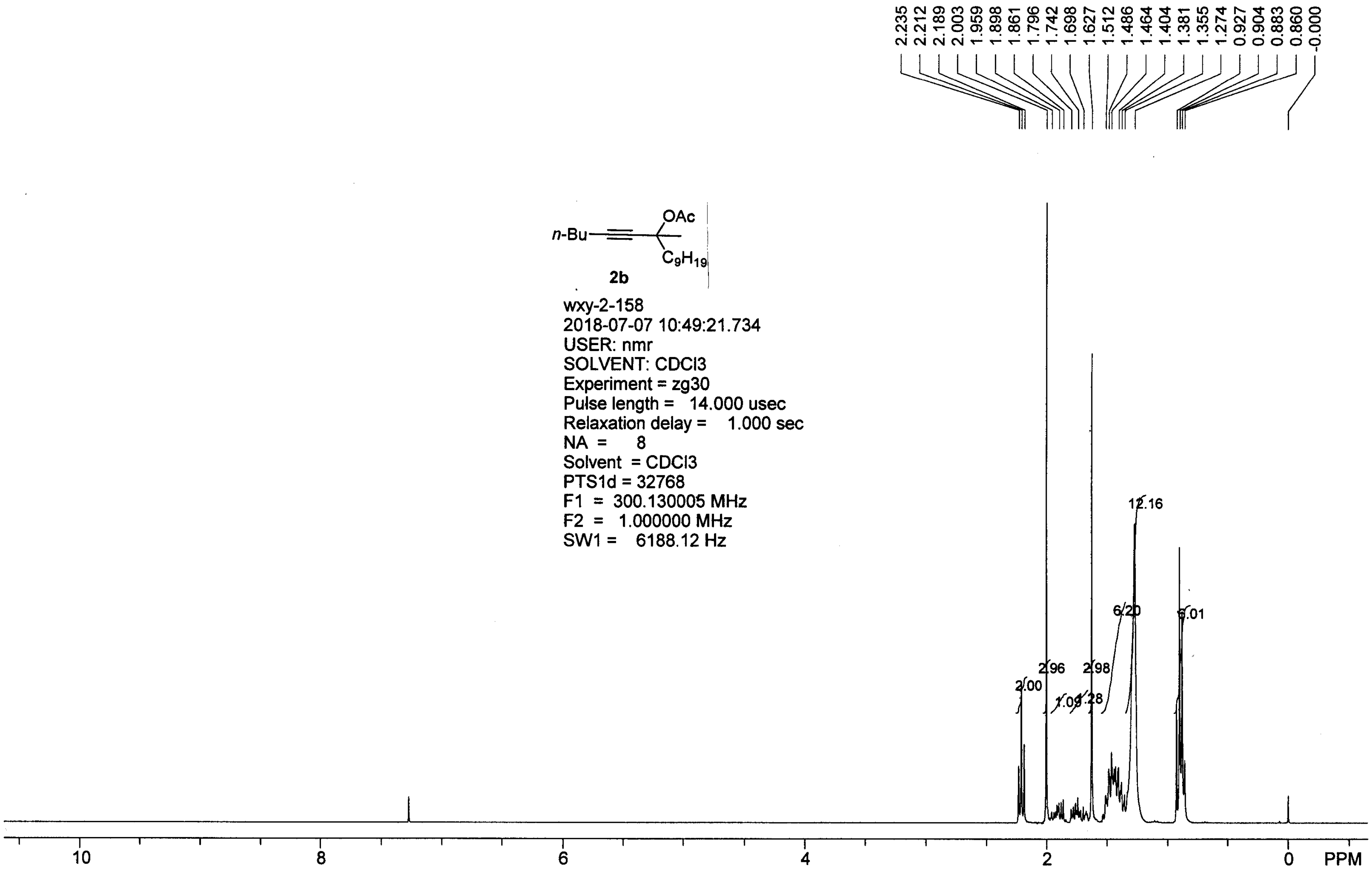
2.235
2.212
2.189
2.003
1.959
1.898
1.861
1.796
1.742
1.698
1.627
1.512
1.486
1.464
1.404
1.381
1.355
1.274
0.927
0.904
0.883
0.860
-0.000
OAc
n-Bu
C9H19
2b
wxy-2-158
2018-07-07 10:49:21.734
USER: nmr
SOLVENT: CDCl3
Experiment = zg30
Pulse length = 14.000 usec
Relaxation delay = 1.000 sec
NA = 8
Solvent = CDCl3
PTS1d = 32768
F1 = 300.130005 MHz
F2 = 1.000000 MHz
SW1 = 6188.12 Hz
12.16
6.20
6.01
2.96
2.98
2.00
1.09
1.28
10
8
6
4
2
0
PPM

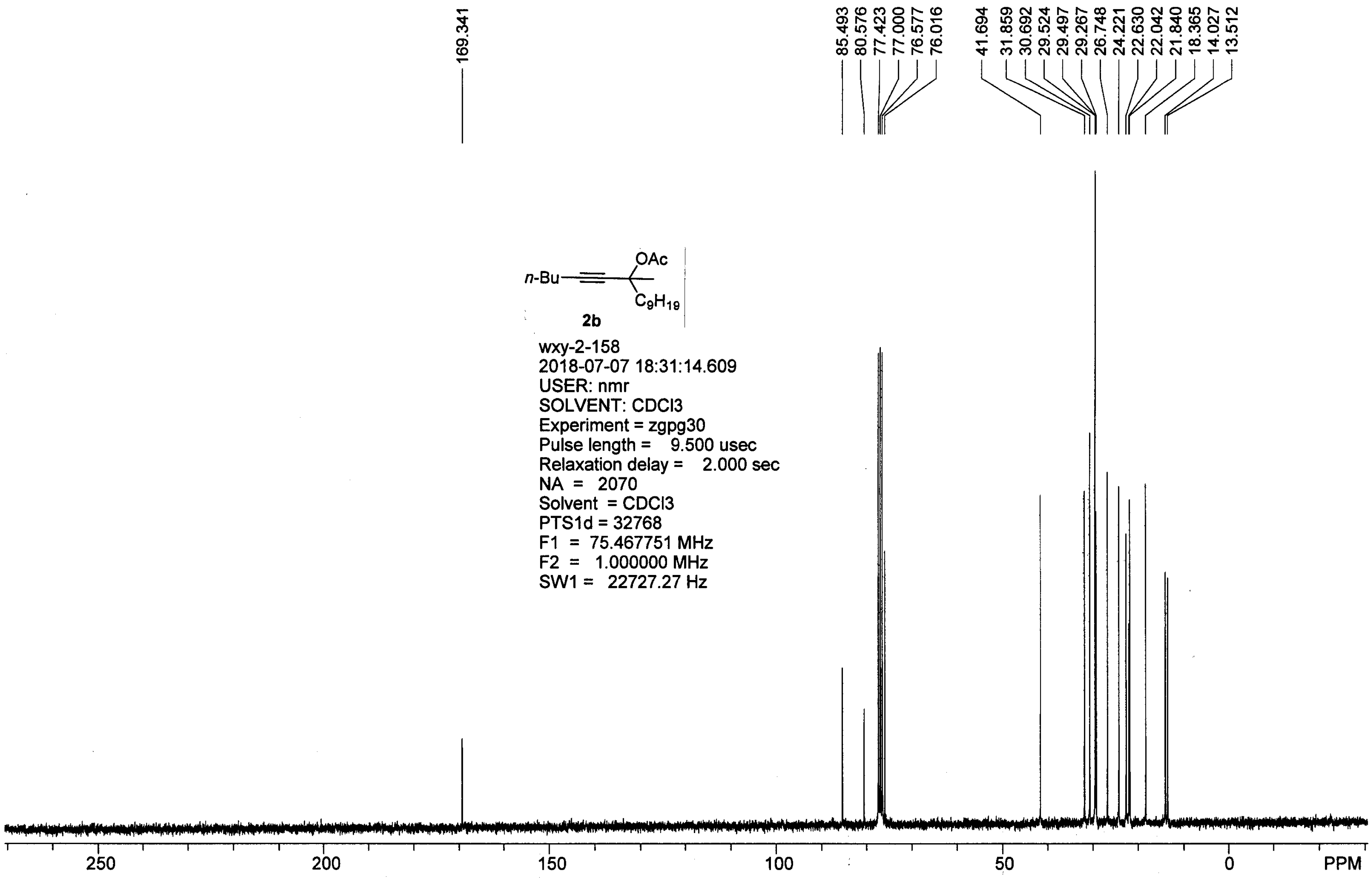
169.341
85.493
80.576
77.423
77.000
76.577
76.016
41.694
31.859
30.692
29.524
29.497
29.267
26.748
24.221
22.630
22.042
21.840
18.365
14.027
13.512
OAc
n-Bu
C9H19
2b
wxy-2-158
2018-07-07 18:31:14.609
USER: nmr
SOLVENT: CDCl3
Experiment = zgpg30
Pulse length = 9.500 usec
Relaxation delay = 2.000 sec
NA = 2070
Solvent = CDCl3
PTS1d = 32768
F1 = 75.467751 MHz
F2 = 1.000000 MHz
SW1 = 22727.27 Hz
250
200
150
100
50
0
PPM

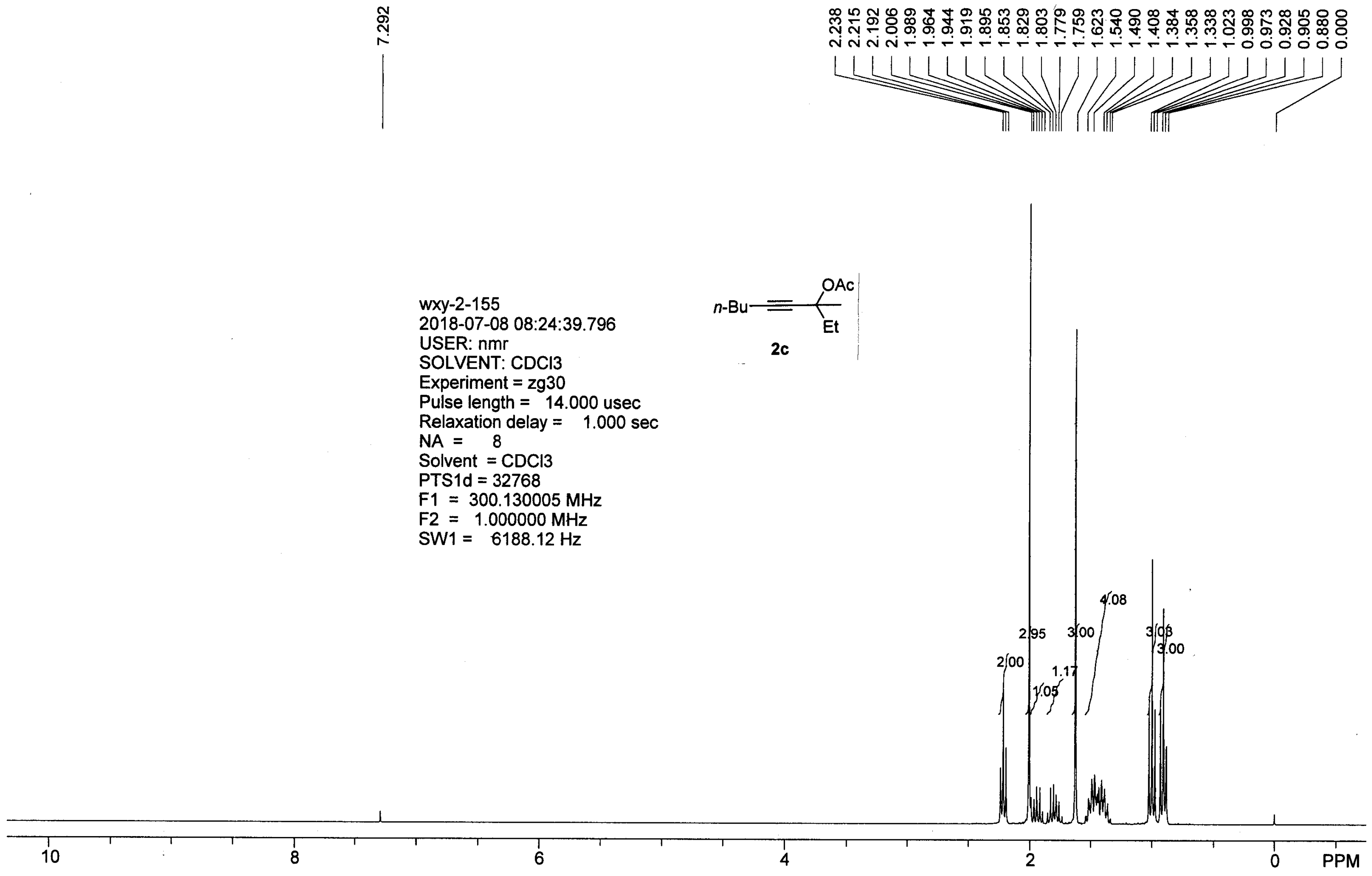

n-Bu
OAc
Et
2c
7.292
2.238
2.215
2.192
2.006
1.989
1.964
1.944
1.919
1.895
1.853
1.829
1.803
1.779
1.759
1.623
1.540
1.490
1.408
1.384
1.358
1.338
1.023
0.998
0.973
0.928
0.905
0.880
0.000
wxy-2-155
2018-07-08 08:24:39.796
USER: nmr
SOLVENT: CDCl3
Experiment = zg30
Pulse length = 14.000 usec
Relaxation delay = 1.000 sec
NA = 8
Solvent = CDCl3
PTS1d = 32768
F1 = 300.130005 MHz
F2 = 1.000000 MHz
SW1 = 6188.12 Hz
2.00
2.95
1.05
1.17
3.00
4.08
3.03
3.00
10
8
6
4
2
0
PPM

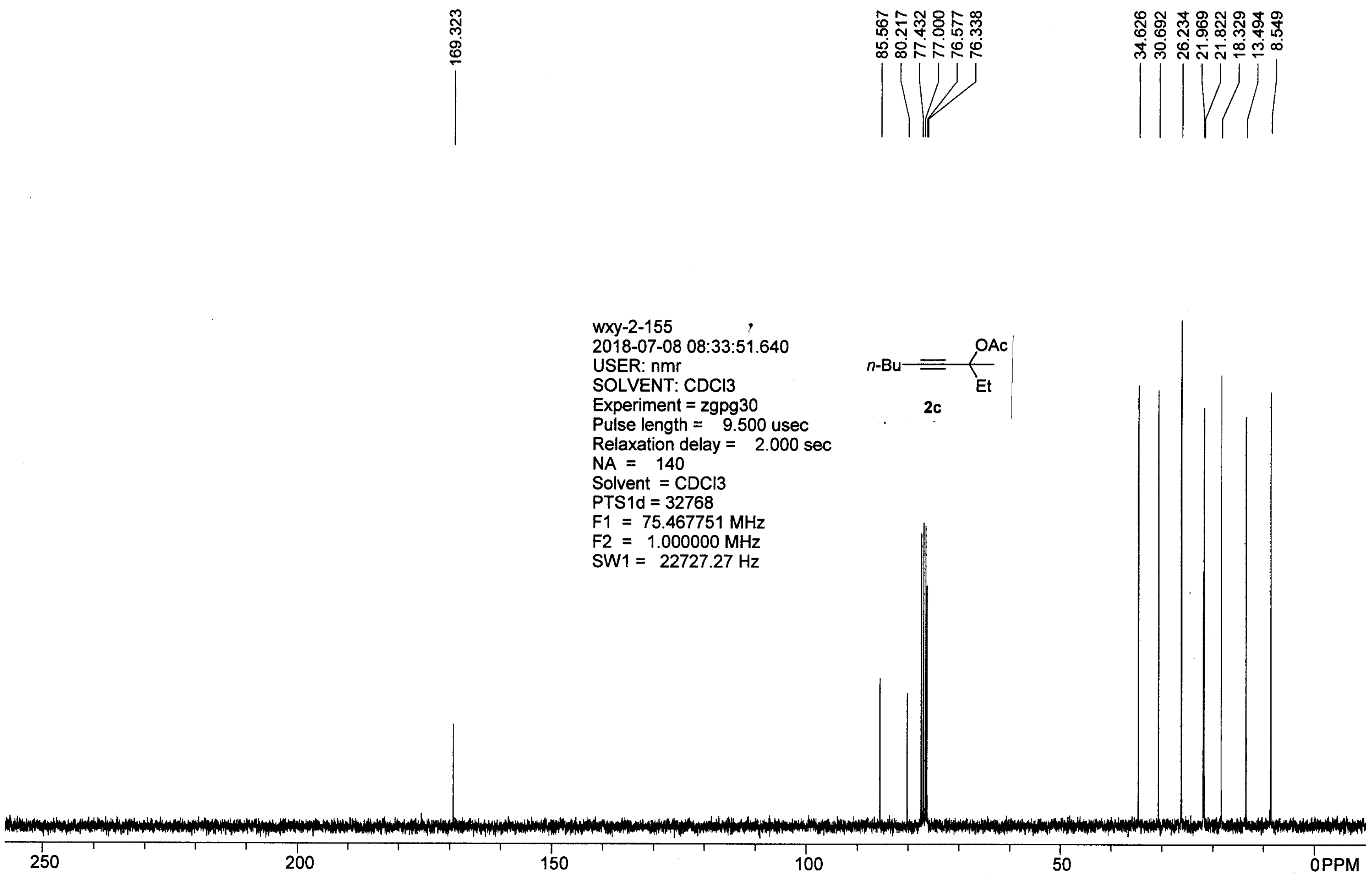
169.323
85.567
80.217
77.432
77.000
76.577
76.338
34.626
30.692
26.234
21.969
21.822
18.329
13.494
8.549
wxy-2-155
2018-07-08 08:33:51.640
USER: nmr
SOLVENT: CDCl3
Experiment = zgpg30
Pulse length = 9.500 usec
Relaxation delay = 2.000 sec
NA = 140
Solvent = CDCl3
PTS1d = 32768
F1 = 75.467751 MHz
F2 = 1.000000 MHz
SW1 = 22727.27 Hz
n-Bu
OAc
Et
2c
250
200
150
100
50
0PPM

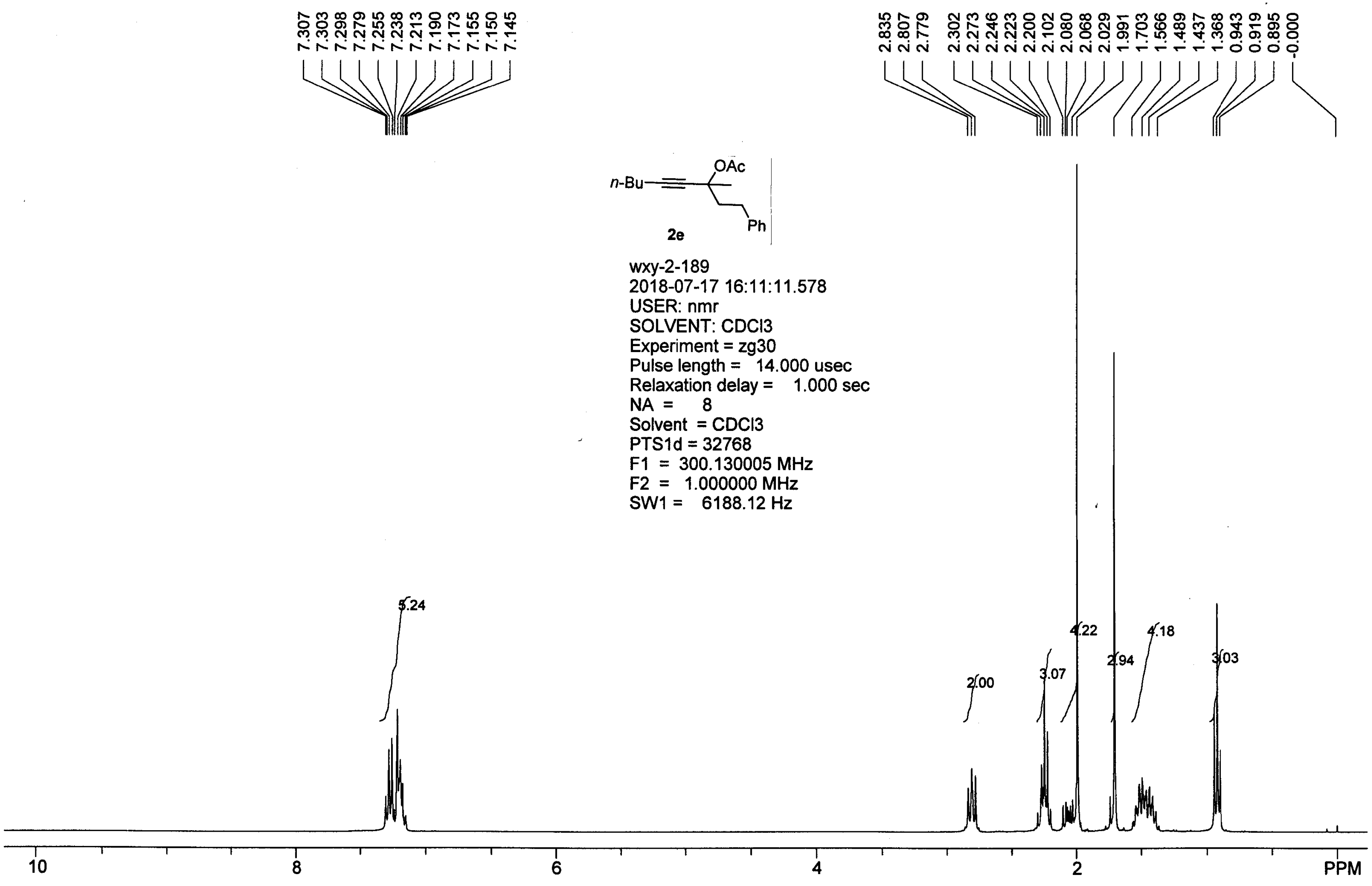
7.307
7.303
7.298
7.279
7.255
7.238
7.213
7.190
7.173
7.155
7.150
7.145
2.835
2.807
2.779
2.302
2.273
2.246
2.223
2.200
2.102
2.080
2.068
2.029
1.991
1.703
1.566
1.489
1.437
1.368
0.943
0.919
0.895
-0.000
n-Bu
OAc
Ph
2e
wxy-2-189
2018-07-17 16:11:11.578
USER: nmr
SOLVENT: CDCl3
Experiment = zg30
Pulse length = 14.000 usec
Relaxation delay = 1.000 sec
NA = 8
Solvent = CDCl3
PTS1d = 32768
F1 = 300.130005 MHz
F2 = 1.000000 MHz
SW1 = 6188.12 Hz
5.24
2.00
3.07
4.22
2.94
4.18
3.03
10
8
6
4
2
PPM

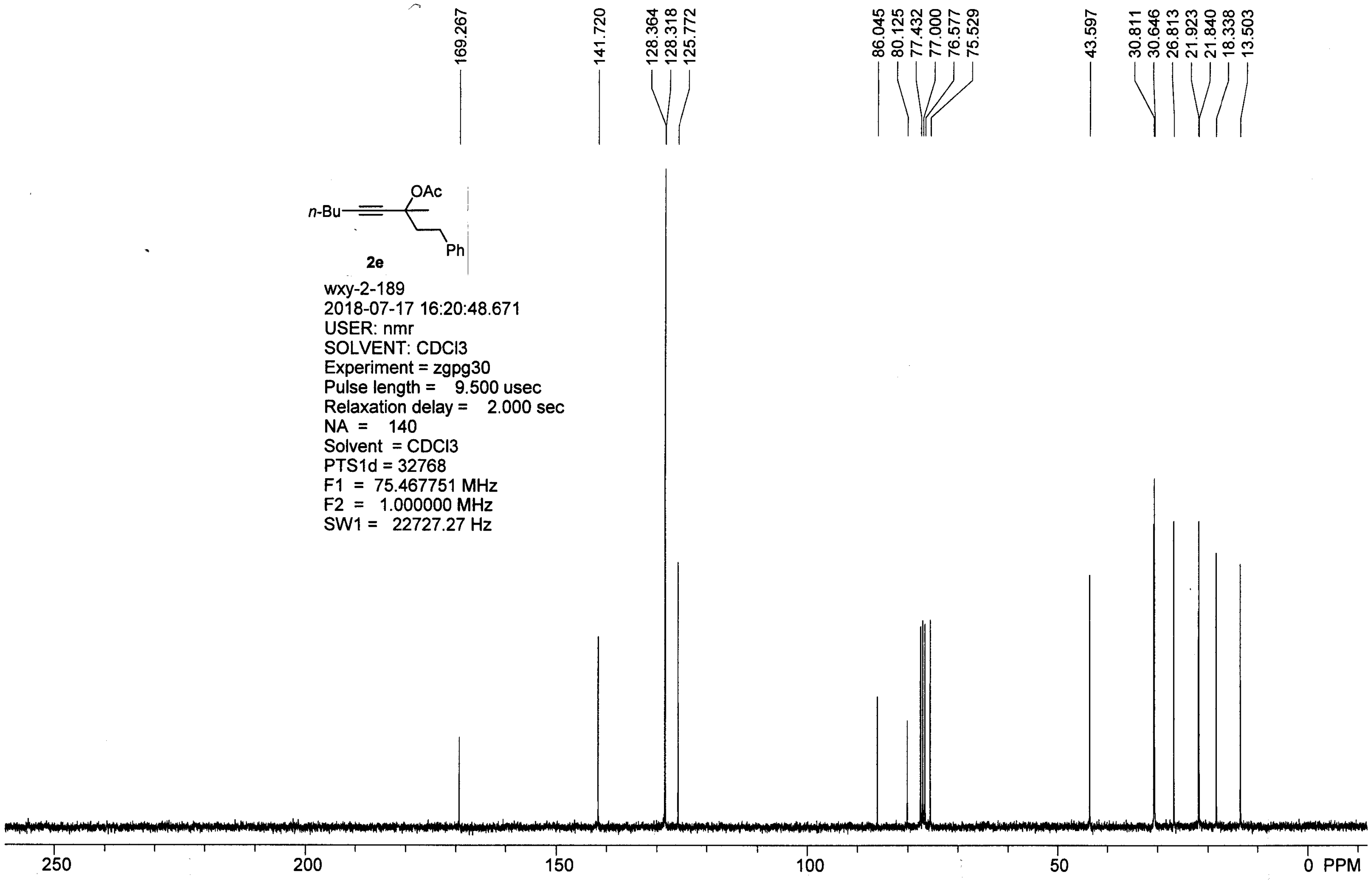
169.267
141.720
128.364
128.318
125.772
86.045
80.125
77.432
77.000
76.577
75.529
43.597
30.811
30.646
26.813
21.923
21.840
18.338
13.503
OAc
n-Bu
Ph
2e
wxy-2-189
2018-07-17 16:20:48.671
USER: nmr
SOLVENT: CDCl3
Experiment = zgpg30
Pulse length = 9.500 usec
Relaxation delay = 2.000 sec
NA = 140
Solvent = CDCl3
PTS1d = 32768
F1 = 75.467751 MHz
F2 = 1.000000 MHz
SW1 = 22727.27 Hz
250
200
150
100
50
0 PPM

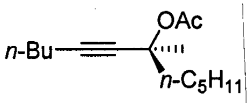

(S)-**2f**

wxy-3-155
2018-12-24 14:10:35.890
USER: nmr
SOLVENT: CDCl3
Experiment = zg30
Pulse length = 14.000 usec
Relaxation delay = 1.000 sec
NA = 8
Solvent = CDCl3
PTS1d = 32768
F1 = 300.130005 MHz
F2 = 1.000000 MHz
SW1 = 6188.12 Hz

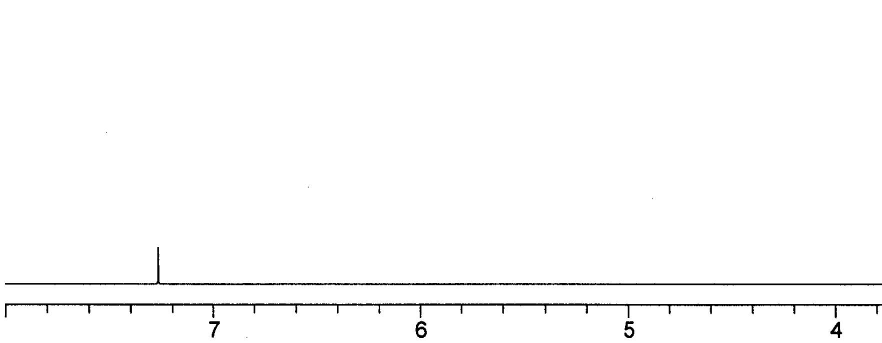
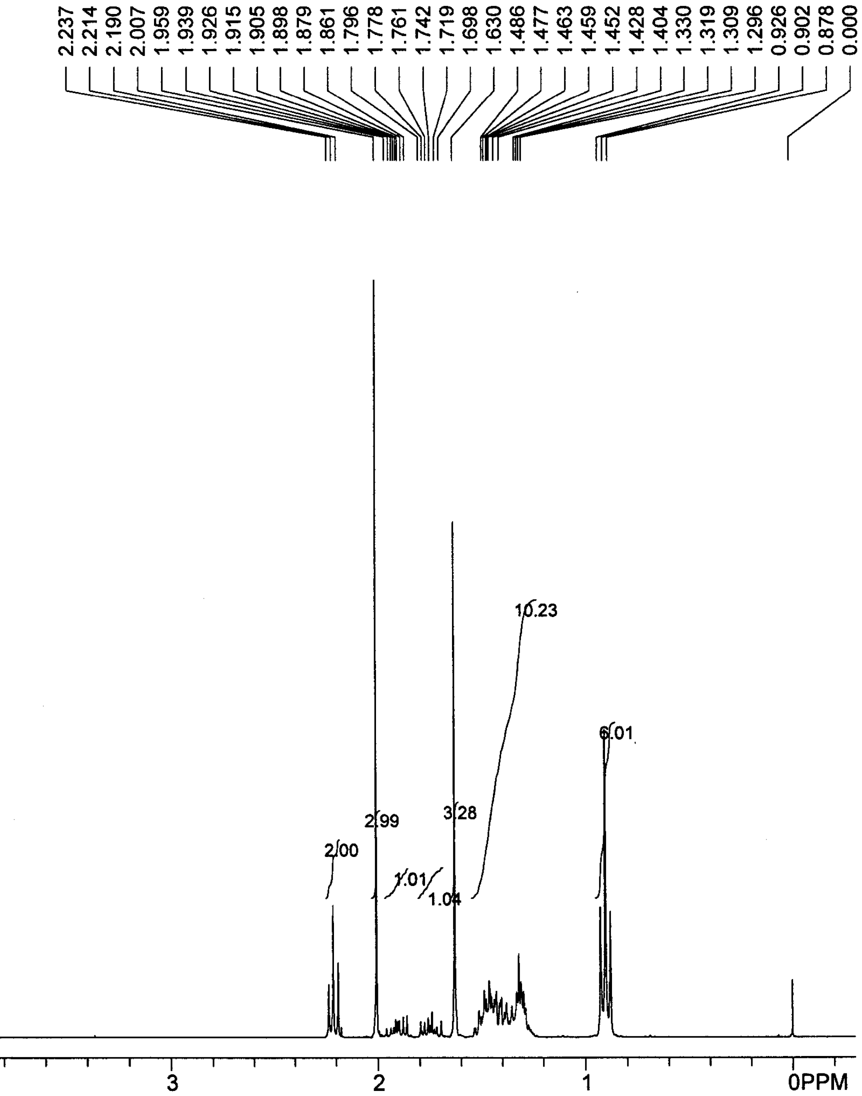

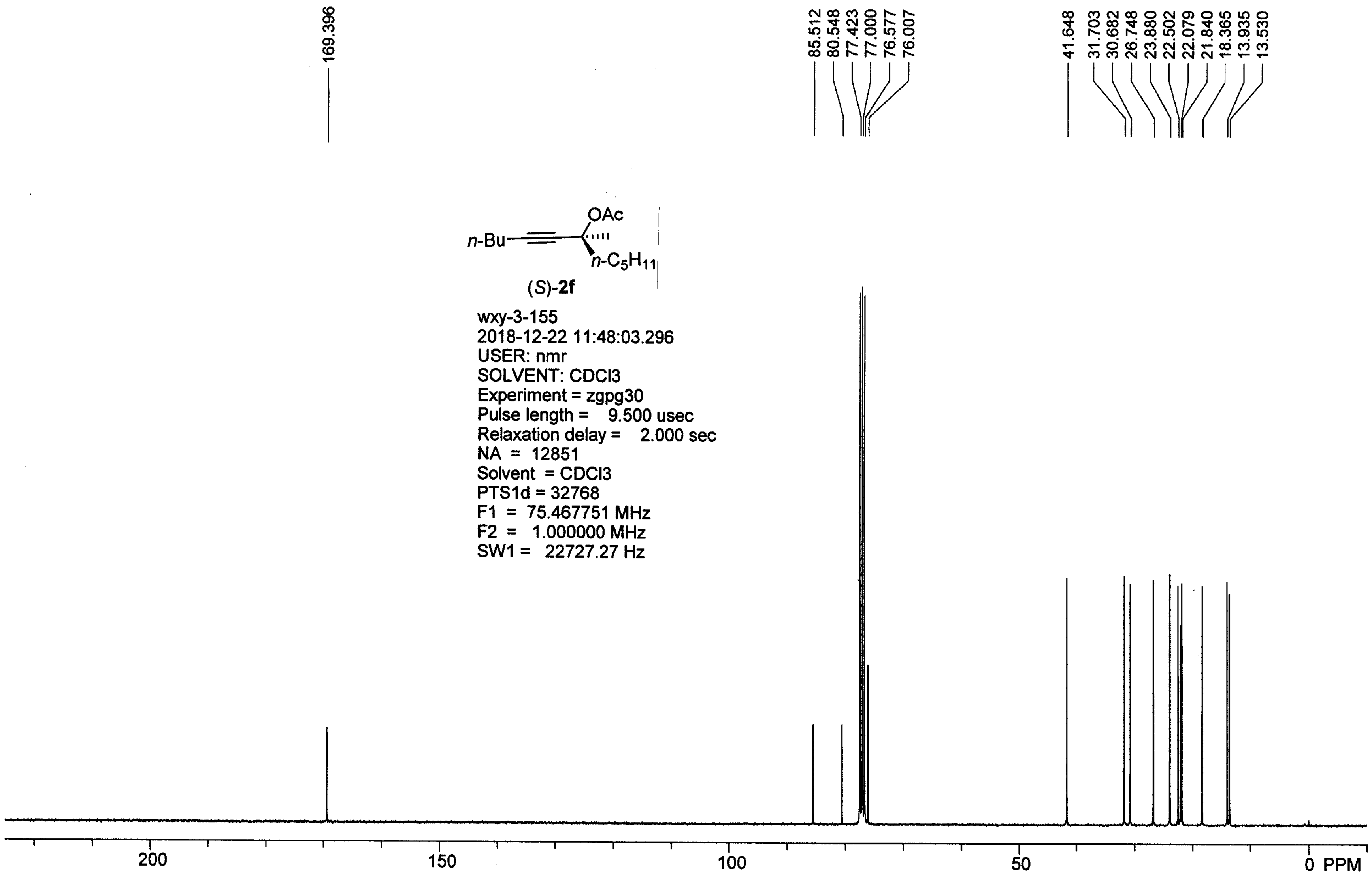
169.396
85.512
80.548
77.423
77.000
76.577
76.007
41.648
31.703
30.682
26.748
23.880
22.502
22.079
21.840
18.365
13.935
13.530
OAc
n-Bu
n-C5H11
(S)-2f
wxy-3-155
2018-12-22 11:48:03.296
USER: nmr
SOLVENT: CDCl3
Experiment = zgpg30
Pulse length = 9.500 usec
Relaxation delay = 2.000 sec
NA = 12851
Solvent = CDCl3
PTS1d = 32768
F1 = 75.467751 MHz
F2 = 1.000000 MHz
SW1 = 22727.27 Hz
200
150
100
50
0 PPM

中国科学院上海有机化学研究所

Project Name: defaults for copy

Reported by User: Breeze user (Breeze)

Breeze 2 HPLC System

SAMPLE INFORMATION

Sample Name:	wxy-3-155-rac-oz-h-100-0-1-214	Acquired By:	Breeze
Sample Type:	未知	Date Acquired:	2018/12/28 18:01:32 CST
Vial:	999	Acq. Method:	zgj100
Injection #:	5	Date Processed:	2018/12/28 18:37:06 CST
Injection Volume:	10.00 ul	Channel Name:	W2489 ChA
Run Time:	20.00 Minutes	Channel Desc.:	W2489 ChA 214nm
Column Type:		Sample Set Name:	

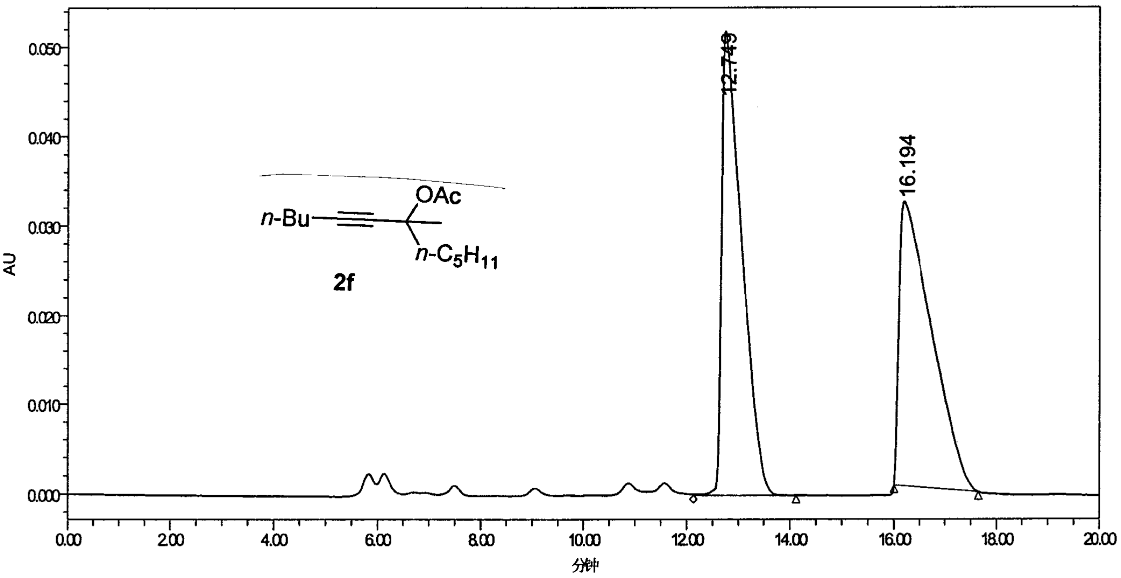

	RT (min)	Area (磺sec)	%Area	Height (磺)	% Height
1	12.749	1410827	50.01	51966	62.00
2	16.194	1410533	49.99	31849	38.00

中国科学院上海有机化学研究所

Project Name: defaults for copy

Reported by User: Breeze user (Breeze)

Breeze 2 HPLC System

SAMPLE INFORMATION

Sample Name:	wxy-3-155-oz-h-100-0-1-214	Acquired By:	Breeze
Sample Type:	未知	Date Acquired:	2018/12/28 17:25:08 CST
Vial:	999	Acq. Method:	zgj100
Injection #:	4	Date Processed:	2019/1/8 17:26:46 CST
Injection Volume:	10.00 ul	Channel Name:	W2489 ChA
Run Time:	20.00 Minutes	Channel Desc.:	W2489 ChA 214nm
Column Type:		Sample Set Name:	

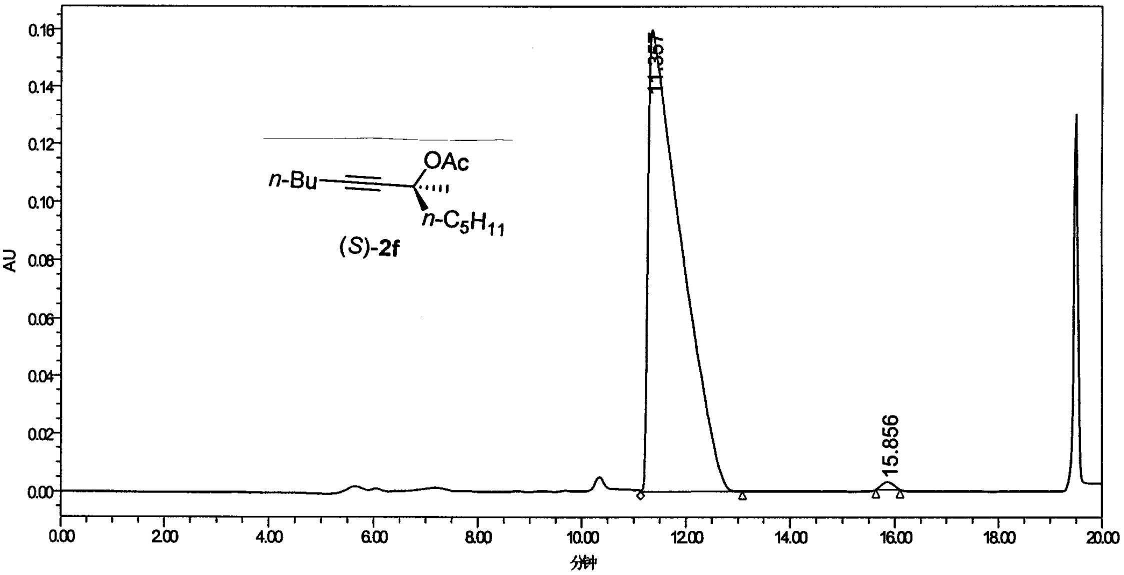

	RT (min)	Area ([illegible]sec)	% Area	Height ([illegible])	% Height
1	11.357	6972839	99.40	160112	98.36
2	15.856	41964	0.60	2662	1.64

Report Method: Individual Report ASC

Page: 1 (共计 1)

Printed: 2019/1/8
17:27:26 PRC

qya-7-024-racemic

实验时间：2018-12-21,9:14:26
谱图文件:D:\浙大智达\N2000\样品\S20181221091426.org
方法文件:D:\浙大智达\N2000\djx.mtd

实验者：wxy
报告时间：2018-12-21,9:57:48
积分方法:面积归一法

实验内容简介：
AD-H,n-hexane/i-PrOH = 99/1,1.0,214

色谱图(S20181221091426.org)

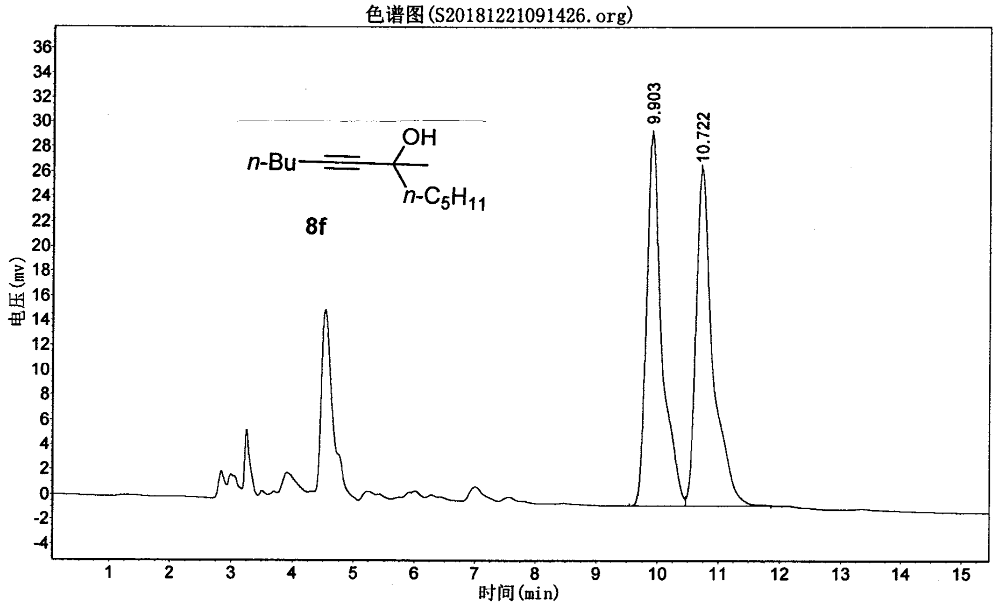

分析结果表

峰号	峰名	保留时间	峰高	峰面积	含量
1		9.903	29890.783	523274.000	49.8870
2		10.722	27129.158	525644.063	50.1130
总计			57019.941	1048918.063	100.0000

qya-7-024-S

实验时间：2018-12-21,8:57:33
谱图文件:D:\浙大智达\N2000\样品\S20181221085733.org
方法文件:D:\浙大智达\N2000\djx.mtd

实验者：wxy
报告时间：2018-12-21,10:04:52
积分方法:面积归一法

实验内容简介：
AD=-H,n-hexane/i-PrOH = 99/1,1.0,214

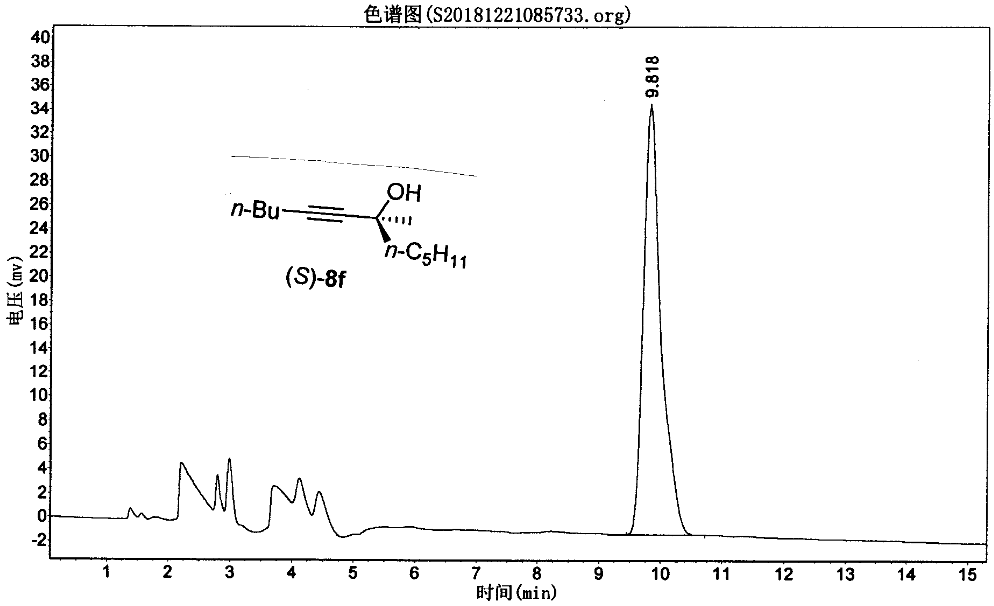

分析结果表

峰号	峰名	保留时间	峰高	峰面积	含量
1		9.818	35601.500	713972.188	100.0000
总计			35601.500	713972.188	100.0000

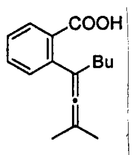

3aa

wxy-2-083-2
2018-05-30 21:57:25.484
USER: nmr
SOLVENT: CDCl3
Experiment = zg30
Pulse length = 14.000 usec
Relaxation delay = 1.000 sec
NA = 8
Solvent = CDCl3
PTS1d = 32768
F1 = 300.130005 MHz
F2 = 1.000000 MHz
SW1 = 6188.12 Hz

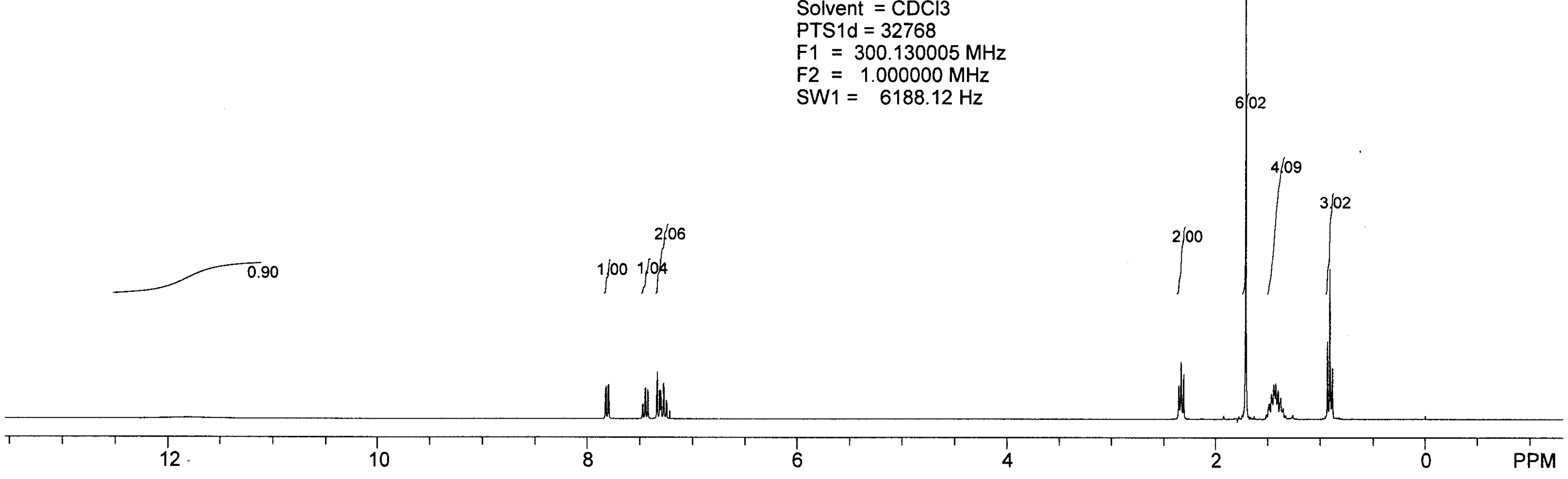

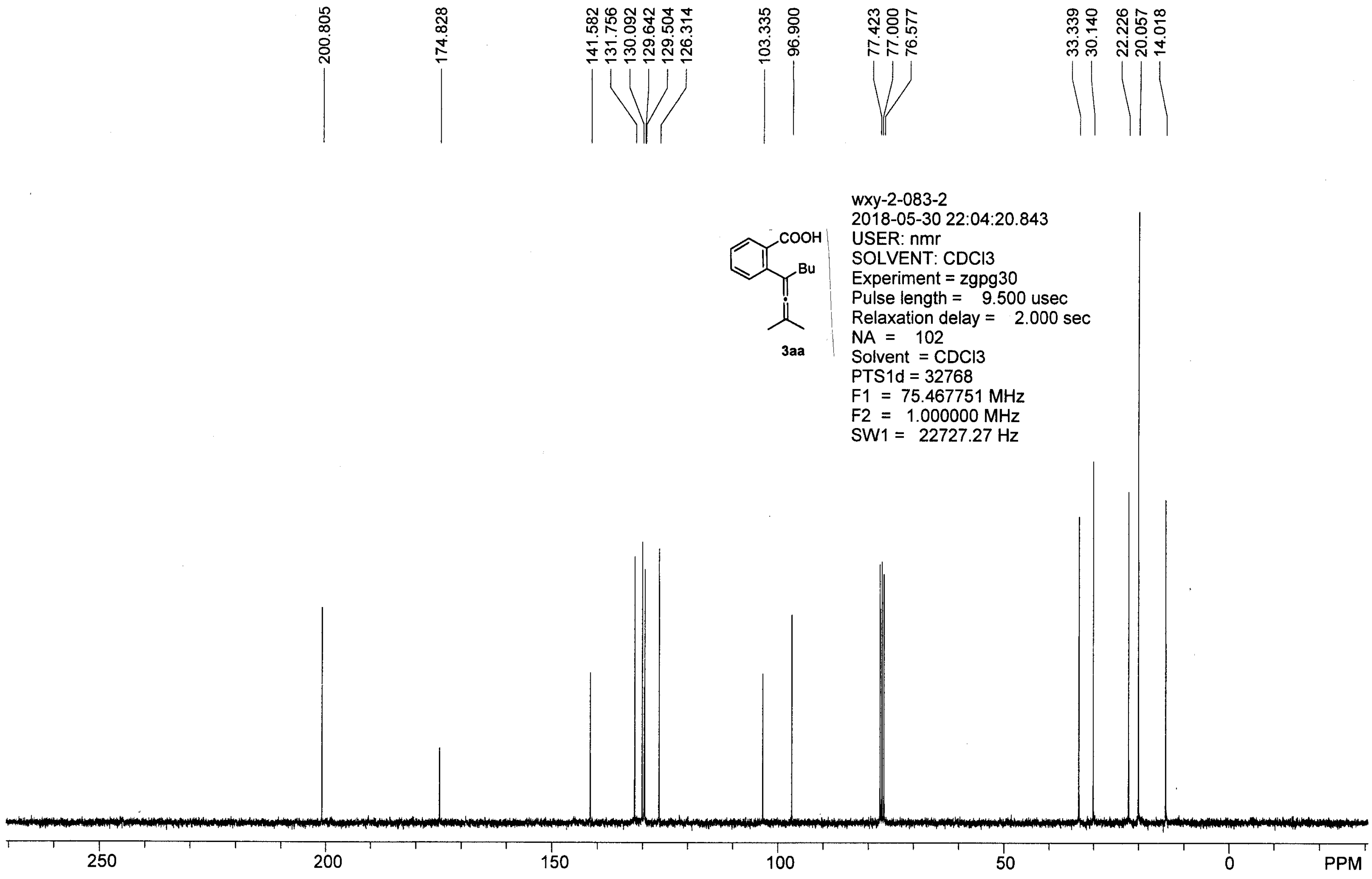
200.805
174.828
141.582
131.756
130.092
129.642
129.504
126.314
103.335
96.900
77.423
77.000
76.577
33.339
30.140
22.226
20.057
14.018
wxy-2-083-2
2018-05-30 22:04:20.843
USER: nmr
SOLVENT: CDCl3
Experiment = zgpg30
Pulse length = 9.500 usec
Relaxation delay = 2.000 sec
NA = 102
Solvent = CDCl3
PTS1d = 32768
F1 = 75.467751 MHz
F2 = 1.000000 MHz
SW1 = 22727.27 Hz
COOH
Bu
3aa
250
200
150
100
50
0
PPM

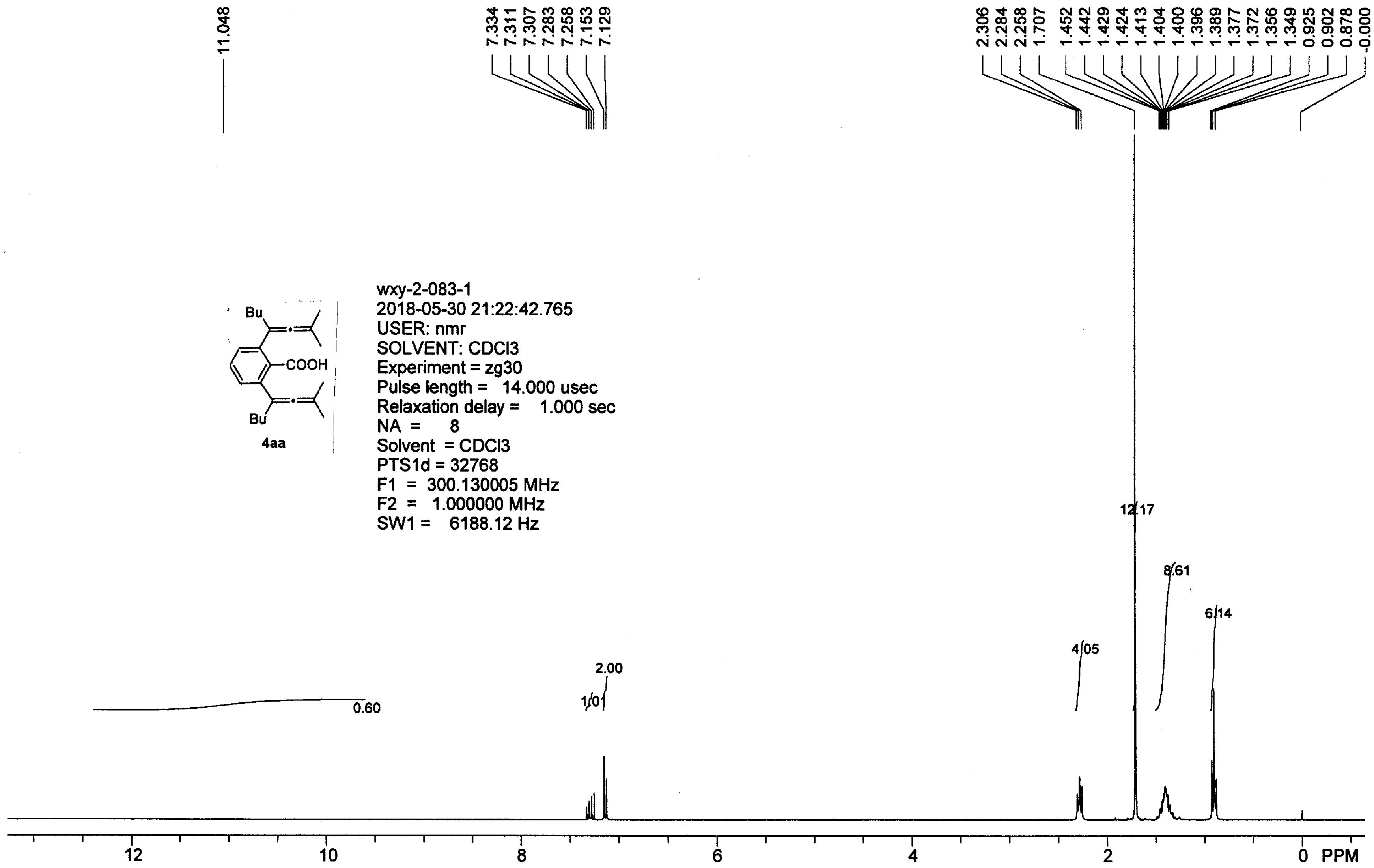

4aa

wxy-2-083-1
2018-05-30 21:22:42.765
USER: nmr
SOLVENT: CDCl3
Experiment = zg30
Pulse length = 14.000 usec
Relaxation delay = 1.000 sec
NA = 8
Solvent = CDCl3
PTS1d = 32768
F1 = 300.130005 MHz
F2 = 1.000000 MHz
SW1 = 6188.12 Hz

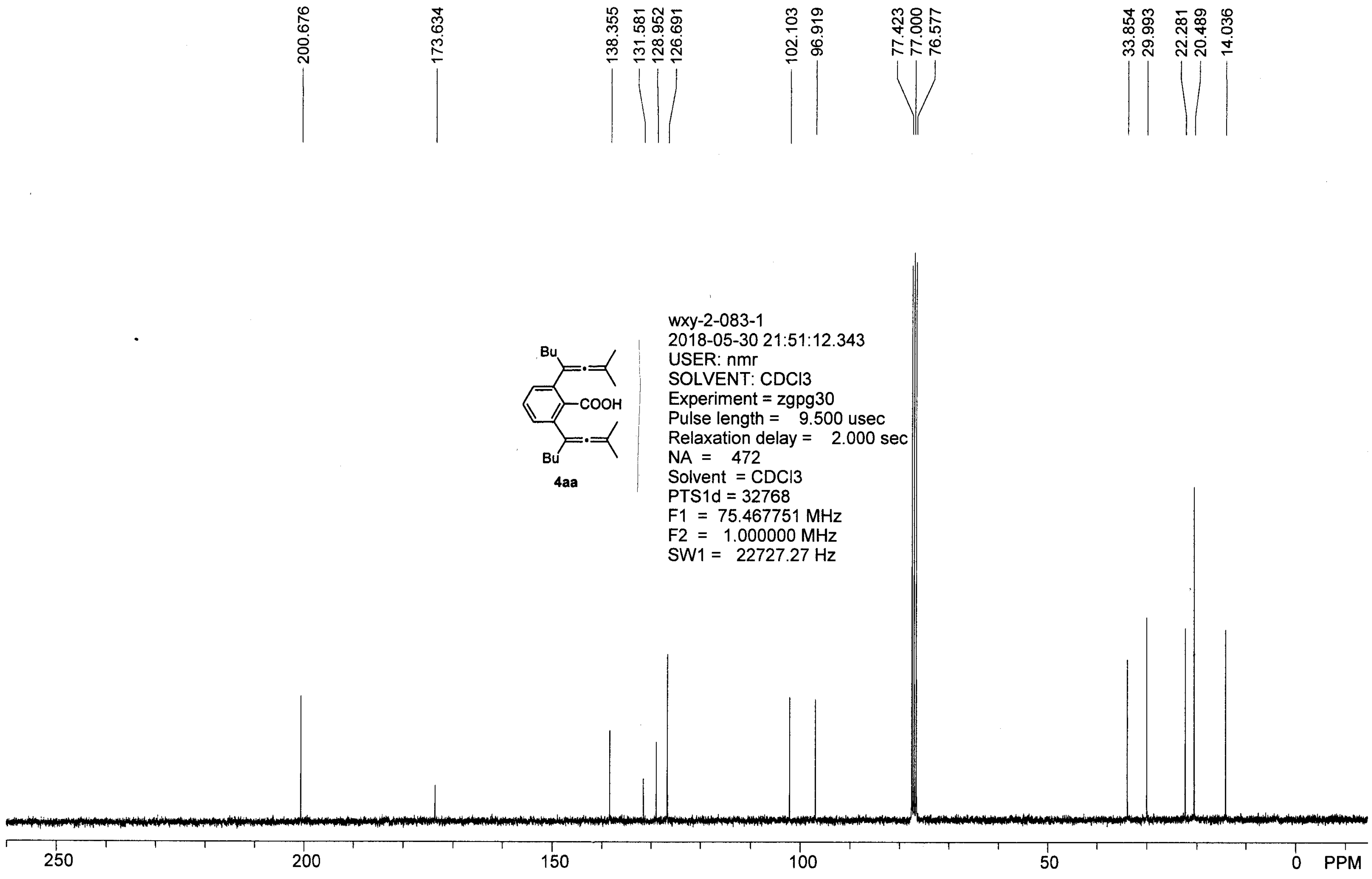

200.676
173.634
138.355
131.581
128.952
126.691
102.103
96.919
77.423
77.000
76.577
33.854
29.993
22.281
20.489
14.036
Bu
COOH
Bu
wxy-2-083-1
2018-05-30 21:51:12.343
USER: nmr
SOLVENT: CDCl3
Experiment = zgpg30
Pulse length = 9.500 usec
Relaxation delay = 2.000 sec
NA = 472
Solvent = CDCl3
PTS1d = 32768
F1 = 75.467751 MHz
F2 = 1.000000 MHz
SW1 = 22727.27 Hz
250
200
150
100
50
0
PPM

4aa

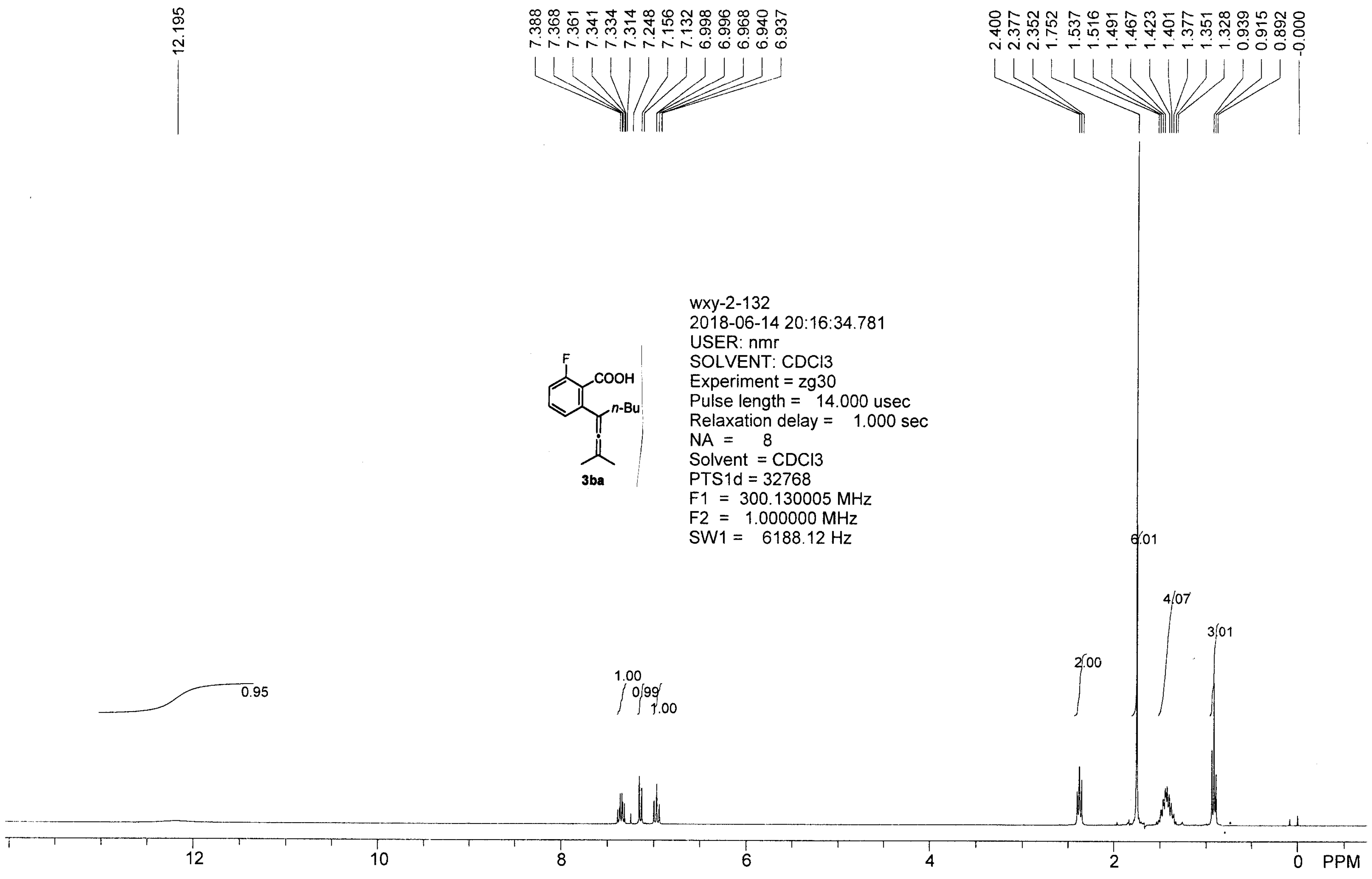
12.195
7.388
7.368
7.361
7.341
7.334
7.314
7.248
7.156
7.132
6.998
6.996
6.968
6.940
6.937
2.400
2.377
2.352
1.752
1.537
1.516
1.491
1.467
1.423
1.401
1.377
1.351
1.328
0.939
0.915
0.892
-0.000
F
COOH
n-Bu
3ba
wxy-2-132
2018-06-14 20:16:34.781
USER: nmr
SOLVENT: CDCl3
Experiment = zg30
Pulse length = 14.000 usec
Relaxation delay = 1.000 sec
NA = 8
Solvent = CDCl3
PTS1d = 32768
F1 = 300.130005 MHz
F2 = 1.000000 MHz
SW1 = 6188.12 Hz
0.95
1.00
0.99
1.00
2.00
6.01
4.07
3.01
12
10
8
6
4
2
0
PPM

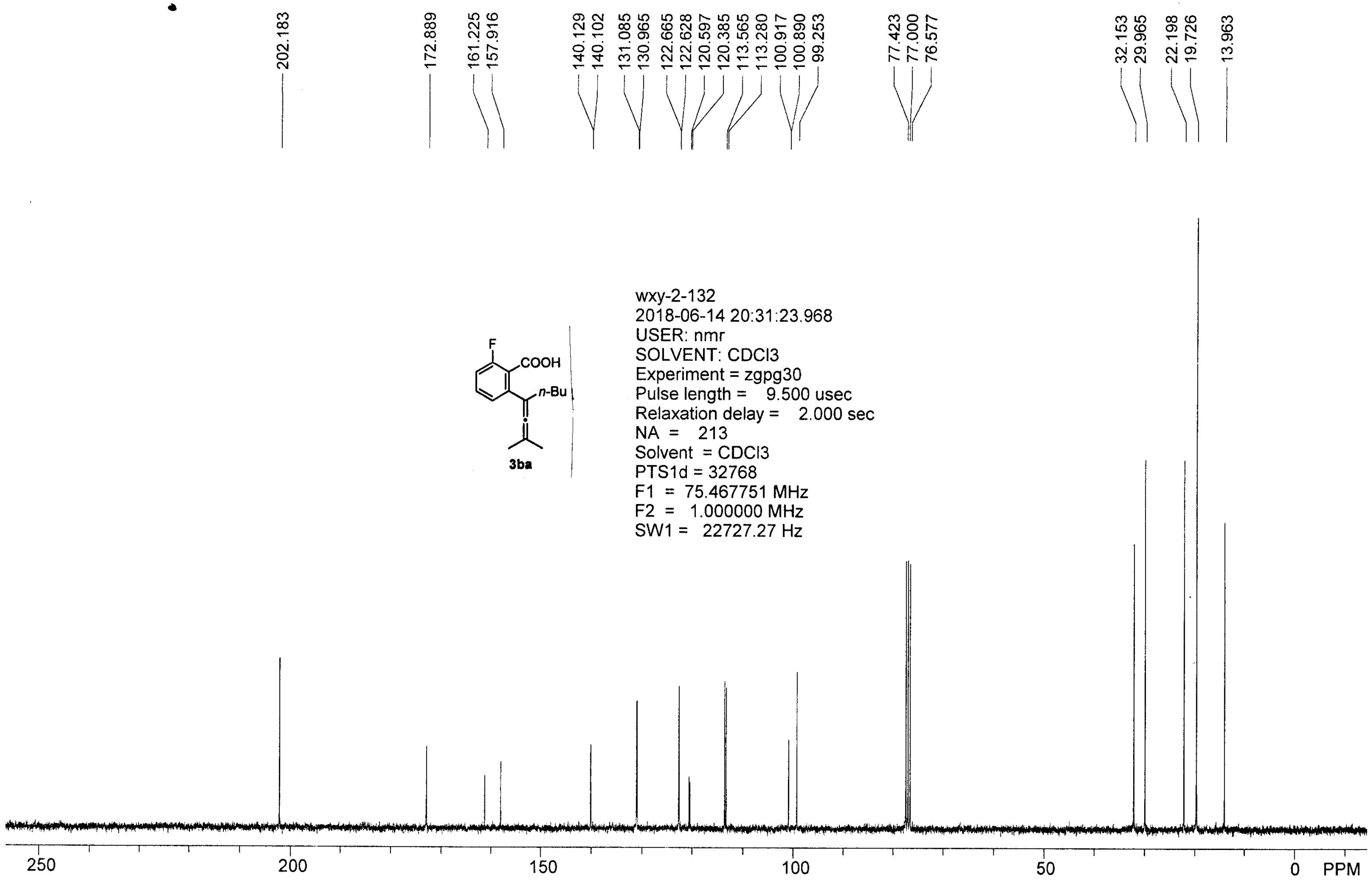
202.183
172.889
161.225
157.916
140.129
140.102
131.085
130.965
122.665
122.628
120.597
120.385
113.565
113.280
100.917
100.890
99.253
77.423
77.000
76.577
32.153
29.965
22.198
19.726
13.963
F
COOH
n-Bu
3ba
wxy-2-132
2018-06-14 20:31:23.968
USER: nmr
SOLVENT: CDCl3
Experiment = zgpg30
Pulse length = 9.500 usec
Relaxation delay = 2.000 sec
NA = 213
Solvent = CDCl3
PTS1d = 32768
F1 = 75.467751 MHz
F2 = 1.000000 MHz
SW1 = 22727.27 Hz
250
200
150
100
50
0
PPM

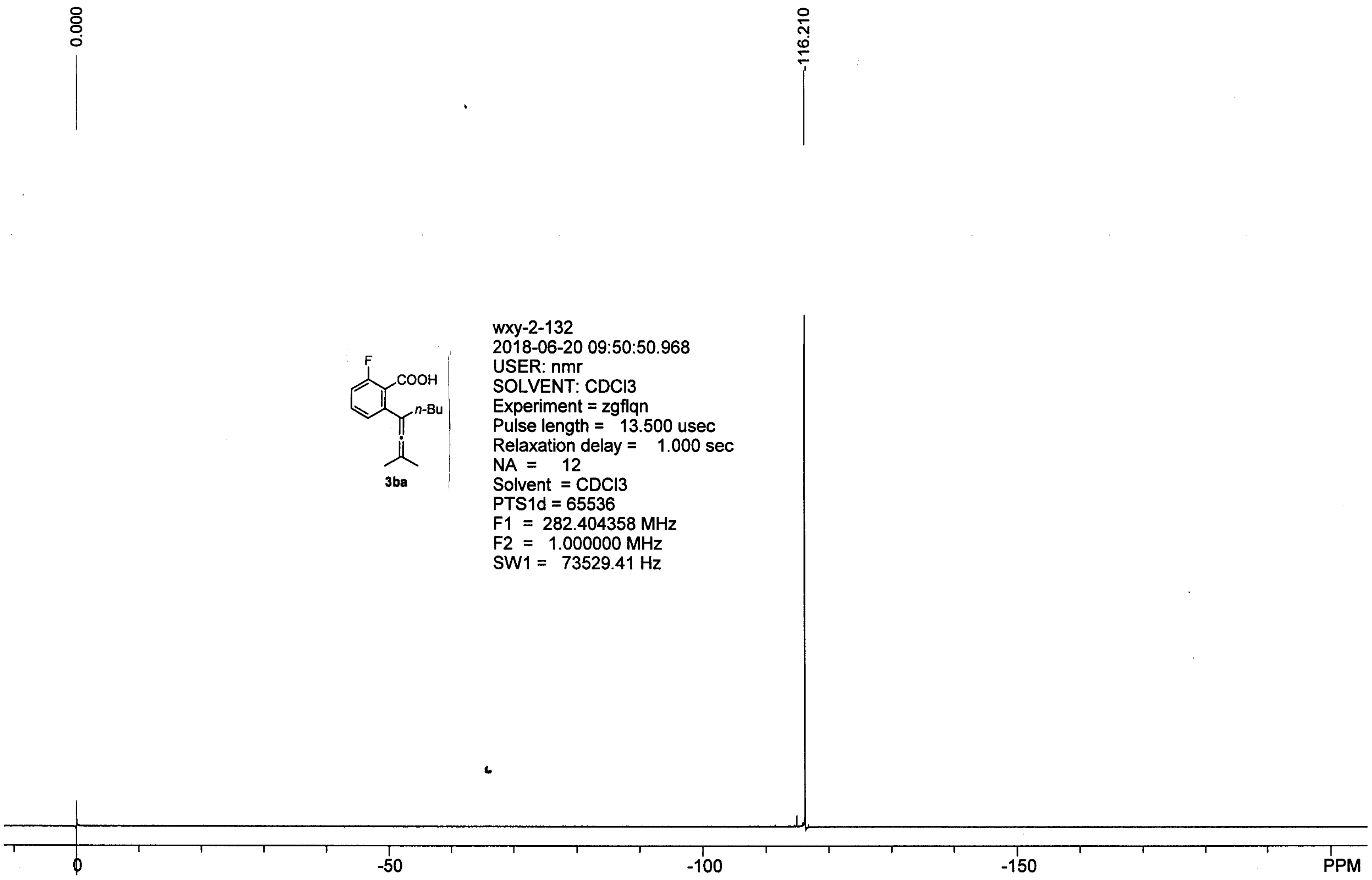
0.000
116.210
F
COOH
n-Bu
3ba
wxy-2-132
2018-06-20 09:50:50.968
USER: nmr
SOLVENT: CDCl3
Experiment = zgflqn
Pulse length = 13.500 usec
Relaxation delay = 1.000 sec
NA = 12
Solvent = CDCl3
PTS1d = 65536
F1 = 282.404358 MHz
F2 = 1.000000 MHz
SW1 = 73529.41 Hz
0
-50
-100
-150
PPM

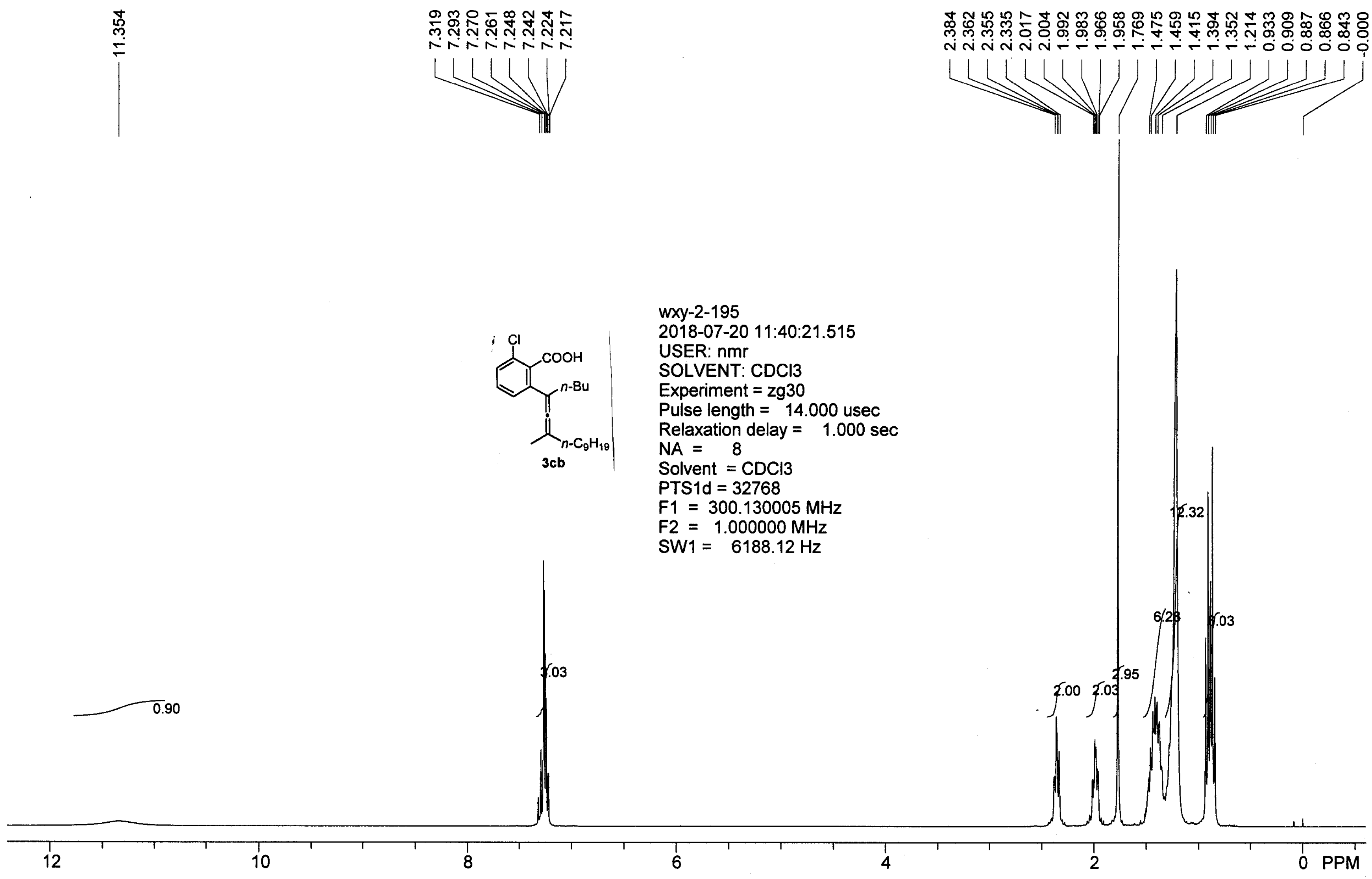
11.354
7.319
7.293
7.270
7.261
7.248
7.242
7.224
7.217
2.384
2.362
2.355
2.335
2.017
2.004
1.992
1.983
1.966
1.958
1.769
1.475
1.459
1.415
1.394
1.352
1.214
0.933
0.909
0.887
0.866
0.843
-0.000
Cl
COOH
n-Bu
n-C9H19
3cb
wxy-2-195
2018-07-20 11:40:21.515
USER: nmr
SOLVENT: CDCl3
Experiment = zg30
Pulse length = 14.000 usec
Relaxation delay = 1.000 sec
NA = 8
Solvent = CDCl3
PTS1d = 32768
F1 = 300.130005 MHz
F2 = 1.000000 MHz
SW1 = 6188.12 Hz
0.90
3.03
2.00
2.03
2.95
6.28
12.32
9.03
12
10
8
6
4
2
0 PPM

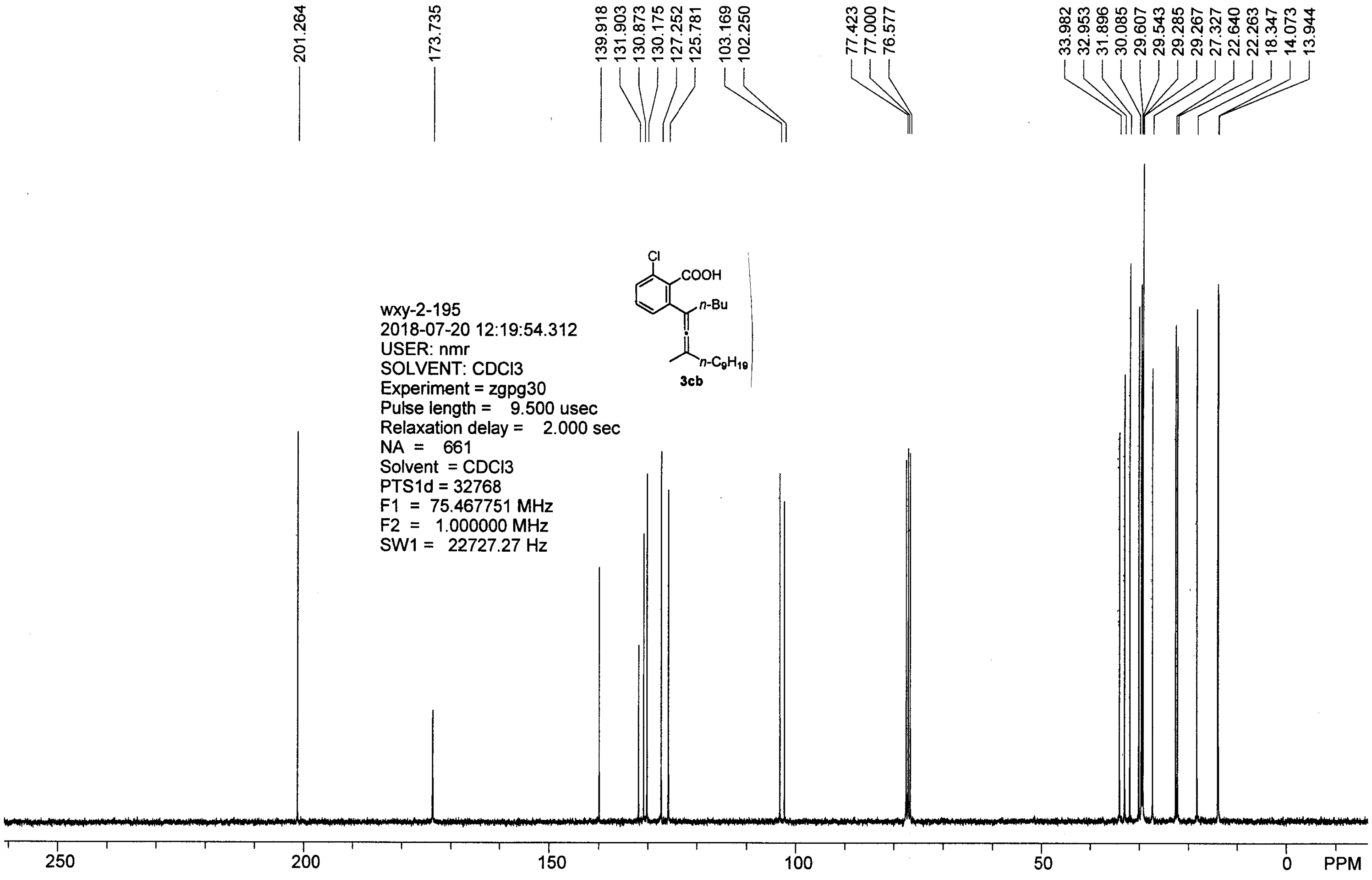
201.264
173.735
139.918
131.903
130.873
130.175
127.252
125.781
103.169
102.250
77.423
77.000
76.577
33.982
32.953
31.896
30.085
29.607
29.543
29.285
29.267
27.327
22.640
22.263
18.347
14.073
13.944
COOH
Cl
n-Bu
n-C9H19
3cb
wxy-2-195
2018-07-20 12:19:54.312
USER: nmr
SOLVENT: CDCl3
Experiment = zgpg30
Pulse length = 9.500 usec
Relaxation delay = 2.000 sec
NA = 661
Solvent = CDCl3
PTS1d = 32768
F1 = 75.467751 MHz
F2 = 1.000000 MHz
SW1 = 22727.27 Hz
250
200
150
100
50
0
PPM

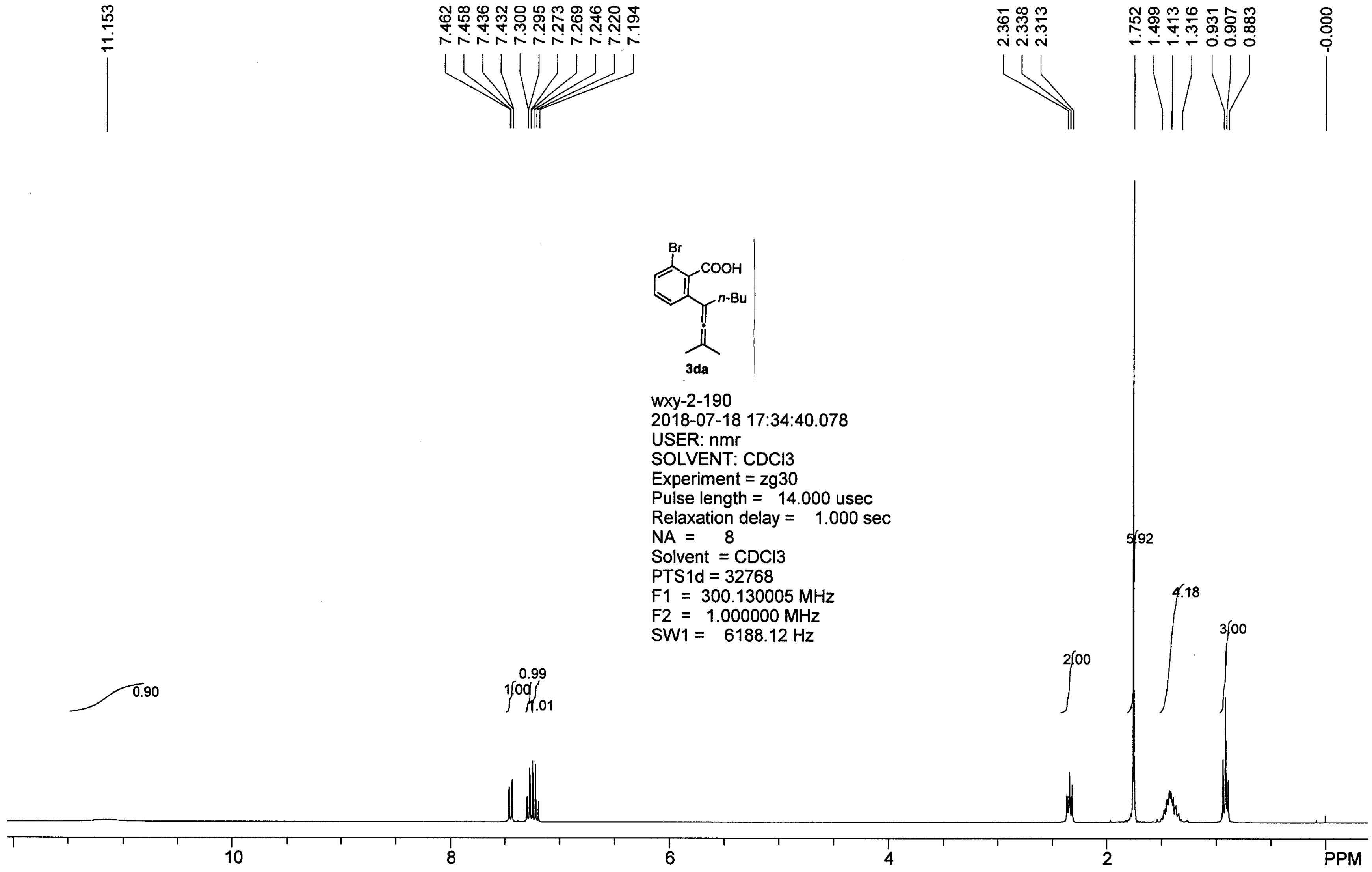
11.153
7.462
7.458
7.436
7.432
7.300
7.295
7.273
7.269
7.246
7.220
7.194
2.361
2.338
2.313
1.752
1.499
1.413
1.316
0.931
0.907
0.883
-0.000
Br
COOH
n-Bu
3da
wxy-2-190
2018-07-18 17:34:40.078
USER: nmr
SOLVENT: CDCl3
Experiment = zg30
Pulse length = 14.000 usec
Relaxation delay = 1.000 sec
NA = 8
Solvent = CDCl3
PTS1d = 32768
F1 = 300.130005 MHz
F2 = 1.000000 MHz
SW1 = 6188.12 Hz
0.90
1.00
0.99
1.01
2.00
5.92
4.18
3.00
10
8
6
4
2
PPM

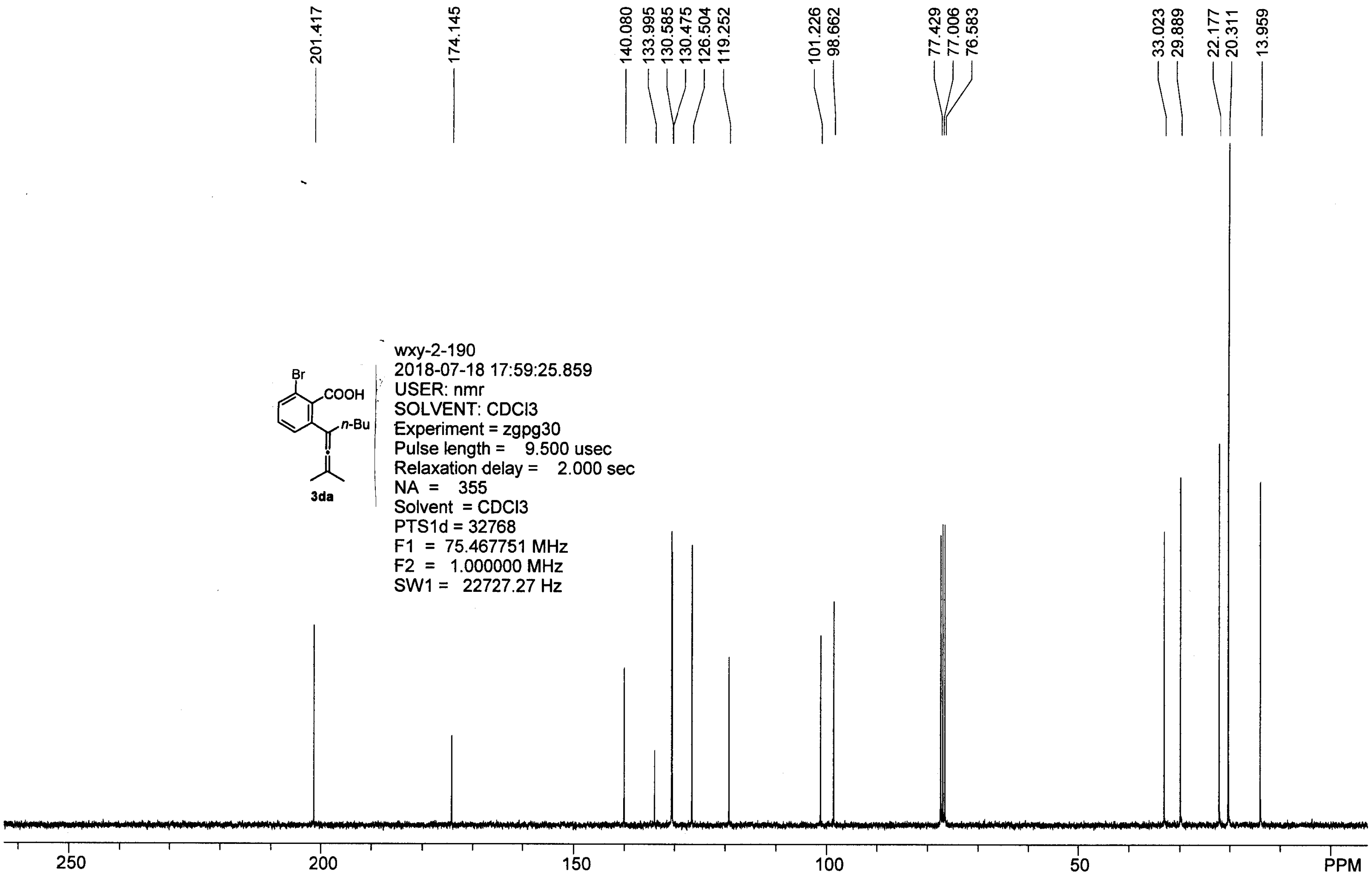
201.417
174.145
140.080
133.995
130.585
130.475
126.504
119.252
101.226
98.662
77.429
77.006
76.583
33.023
29.889
22.177
20.311
13.959
Br
COOH
n-Bu
3da
wxy-2-190
2018-07-18 17:59:25.859
USER: nmr
SOLVENT: CDCl3
Experiment = zgpg30
Pulse length = 9.500 usec
Relaxation delay = 2.000 sec
NA = 355
Solvent = CDCl3
PTS1d = 32768
F1 = 75.467751 MHz
F2 = 1.000000 MHz
SW1 = 22727.27 Hz
250
200
150
100
50
PPM

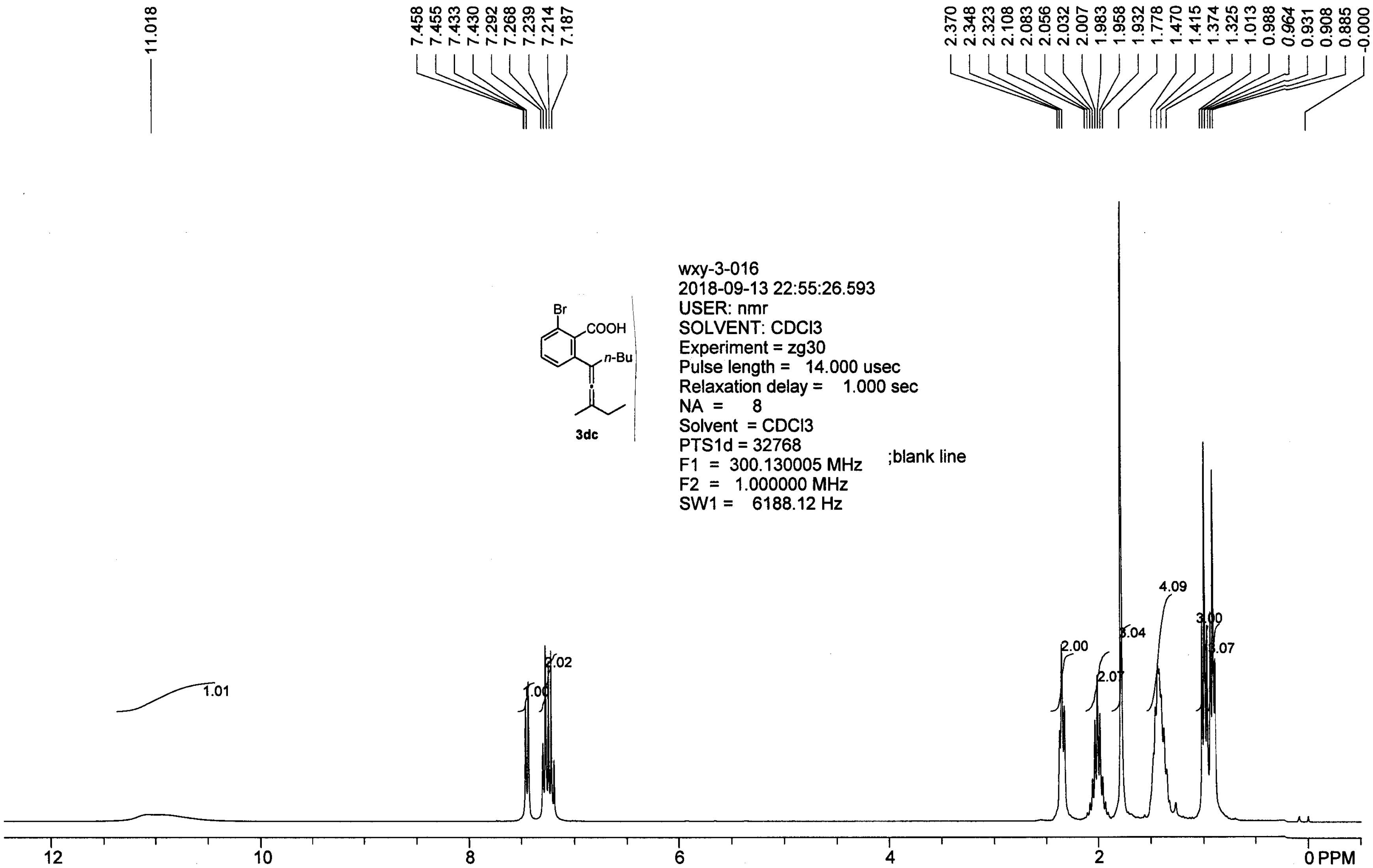
11.018
7.458 7.455 7.433 7.430 7.292 7.268 7.239 7.214 7.187
2.370 2.348 2.323 2.108 2.083 2.056 2.032 2.007 1.983 1.958 1.932 1.778 1.470 1.415 1.374 1.325 1.013 0.988 0.964 0.931 0.908 0.885 -0.000
Br
COOH
n-Bu
3dc
wxy-3-016
2018-09-13 22:55:26.593
USER: nmr
SOLVENT: CDCl3
Experiment = zg30
Pulse length = 14.000 usec
Relaxation delay = 1.000 sec
NA = 8
Solvent = CDCl3
PTS1d = 32768
F1 = 300.130005 MHz
F2 = 1.000000 MHz
SW1 = 6188.12 Hz
;blank line
1.01
1.00
2.02
2.00
2.07
3.04
4.09
3.00
3.07
12
10
8
6
4
2
0 PPM

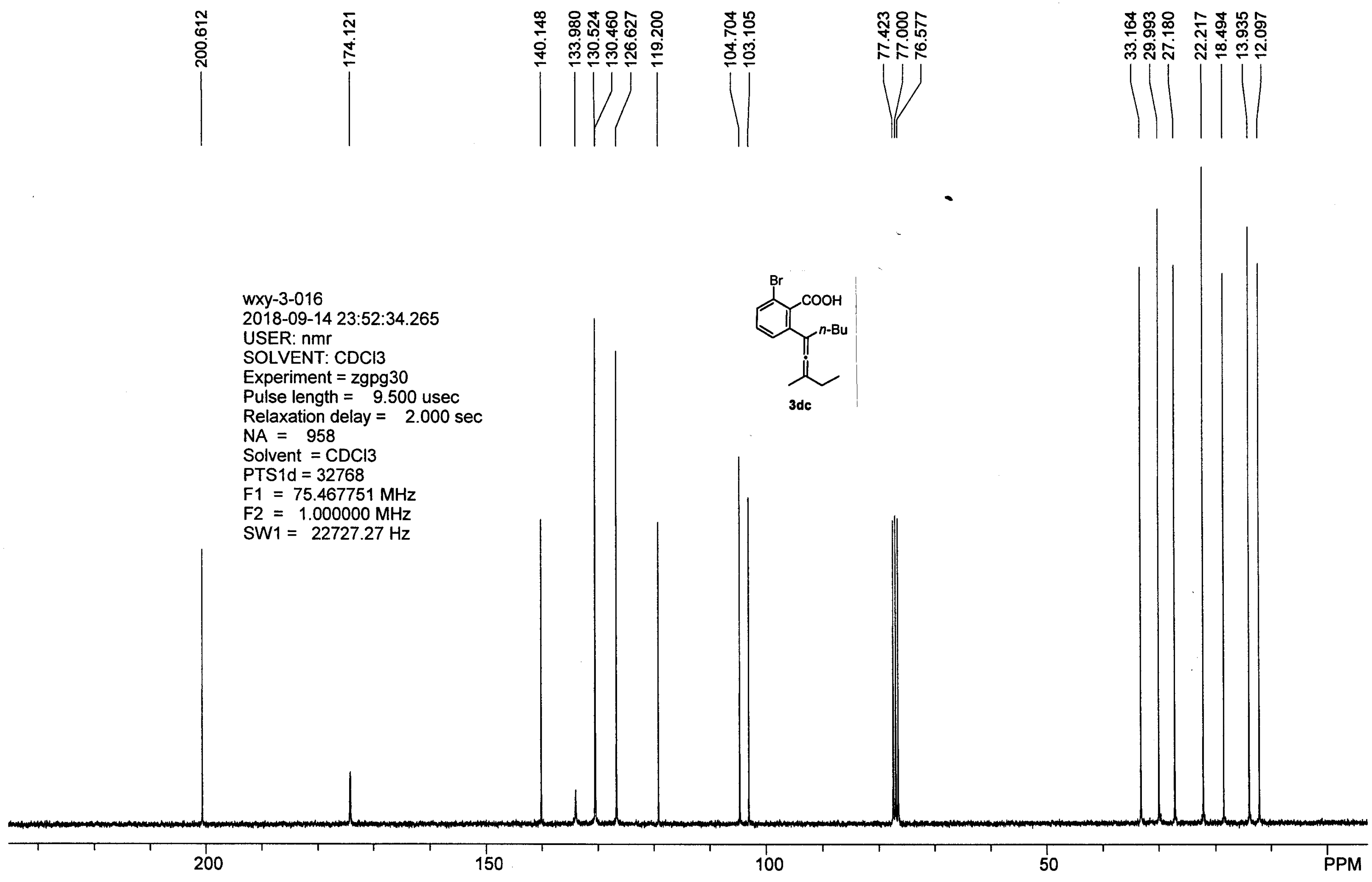
200.612
174.121
140.148
133.980
130.524
130.460
126.627
119.200
104.704
103.105
77.423
77.000
76.577
33.164
29.993
27.180
22.217
18.494
13.935
12.097
wxy-3-016
2018-09-14 23:52:34.265
USER: nmr
SOLVENT: CDCl3
Experiment = zgpg30
Pulse length = 9.500 usec
Relaxation delay = 2.000 sec
NA = 958
Solvent = CDCl3
PTS1d = 32768
F1 = 75.467751 MHz
F2 = 1.000000 MHz
SW1 = 22727.27 Hz
Br
COOH
n-Bu
3dc
200
150
100
50
PPM

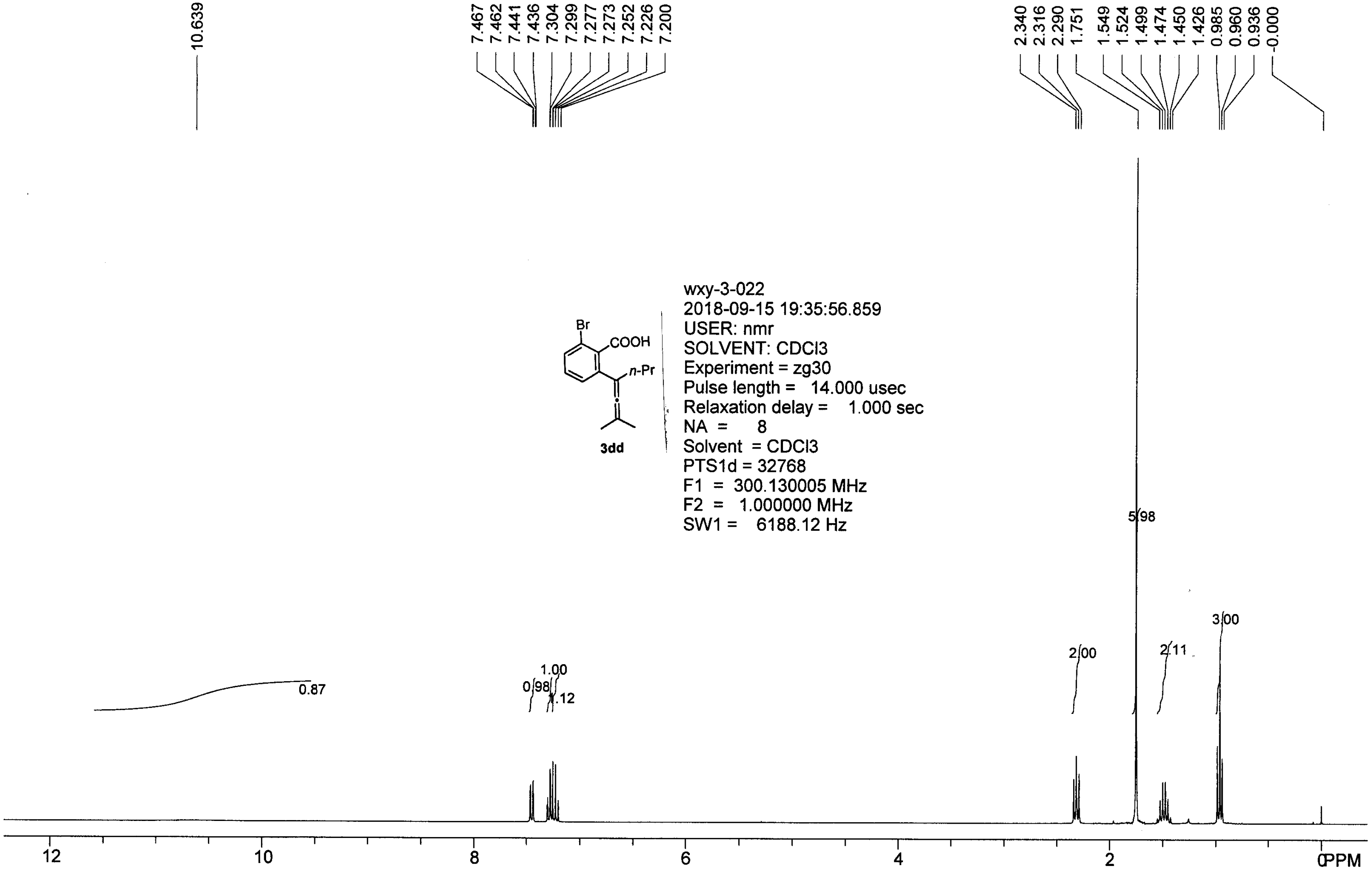

3dd

wxy-3-022
2018-09-15 19:35:56.859
USER: nmr
SOLVENT: CDCl3
Experiment = zg30
Pulse length = 14.000 usec
Relaxation delay = 1.000 sec
NA = 8
Solvent = CDCl3
PTS1d = 32768
F1 = 300.130005 MHz
F2 = 1.000000 MHz
SW1 = 6188.12 Hz

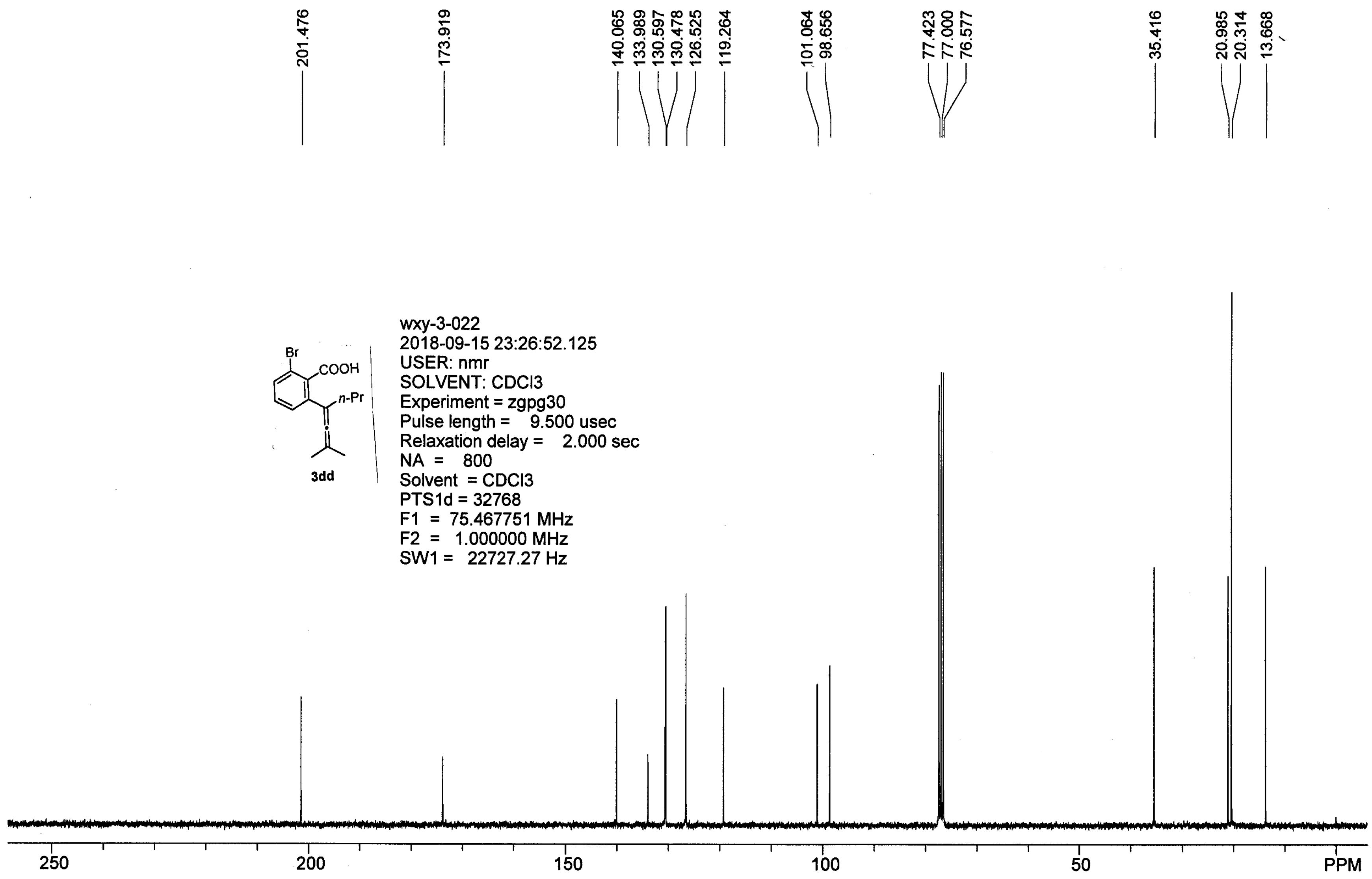
201.476
173.919
140.065
133.989
130.597
130.478
126.525
119.264
101.064
98.656
77.423
77.000
76.577
35.416
20.985
20.314
13.668
Br
COOH
n-Pr
3dd
wxy-3-022
2018-09-15 23:26:52.125
USER: nmr
SOLVENT: CDCl3
Experiment = zgpg30
Pulse length = 9.500 usec
Relaxation delay = 2.000 sec
NA = 800
Solvent = CDCl3
PTS1d = 32768
F1 = 75.467751 MHz
F2 = 1.000000 MHz
SW1 = 22727.27 Hz
250
200
150
100
50
PPM

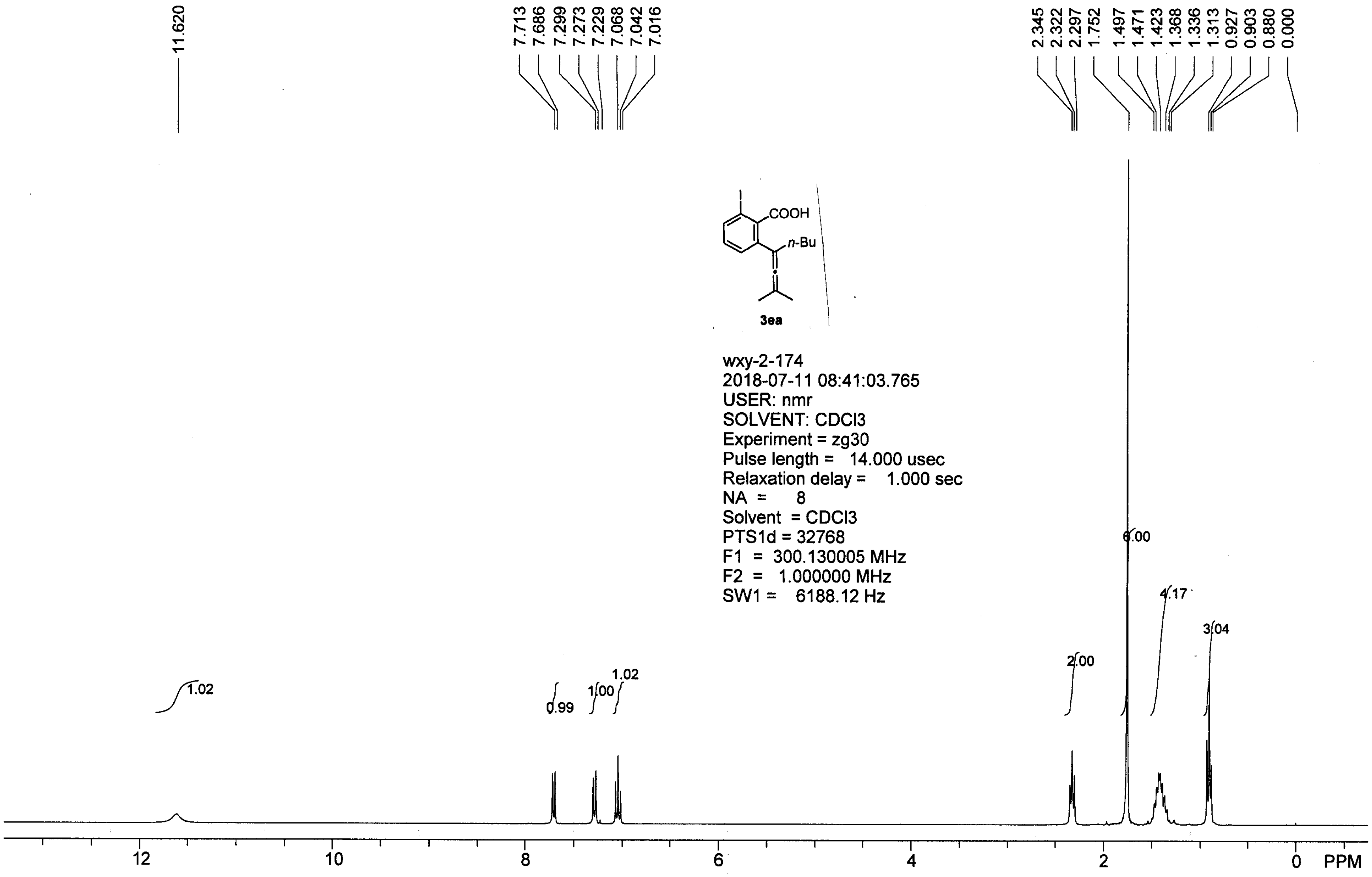
11.620
7.713
7.686
7.299
7.273
7.229
7.068
7.042
7.016
2.345
2.322
2.297
1.752
1.497
1.471
1.423
1.368
1.336
1.313
0.927
0.903
0.880
0.000
COOH
n-Bu
3ea
wxy-2-174
2018-07-11 08:41:03.765
USER: nmr
SOLVENT: CDCl3
Experiment = zg30
Pulse length = 14.000 usec
Relaxation delay = 1.000 sec
NA = 8
Solvent = CDCl3
PTS1d = 32768
F1 = 300.130005 MHz
F2 = 1.000000 MHz
SW1 = 6188.12 Hz
6.00
4.17
3.04
2.00
1.02
1.00
1.02
0.99
12
10
8
6
4
2
0
PPM

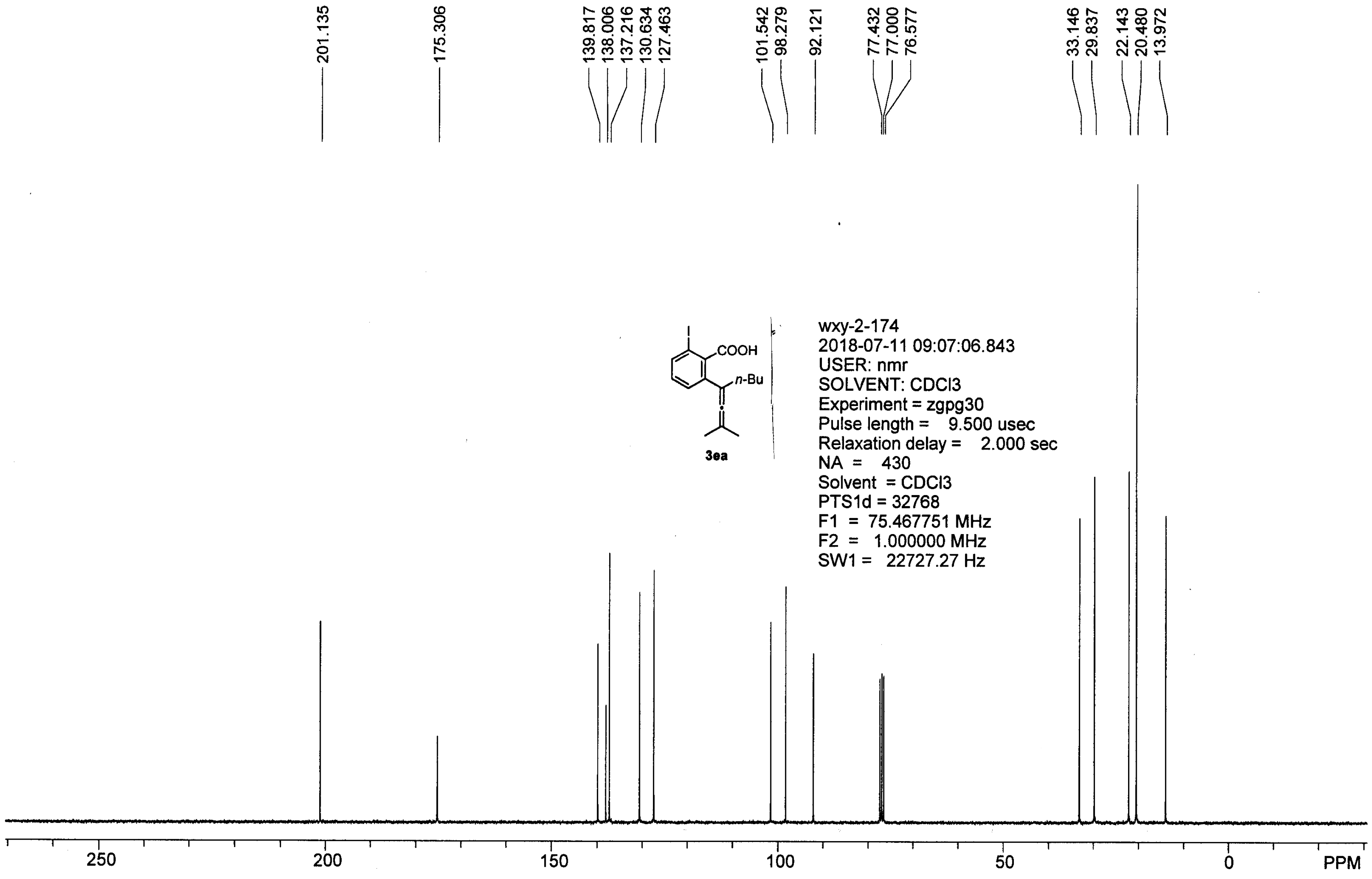
201.135
175.306
139.817
138.006
137.216
130.634
127.463
101.542
98.279
92.121
77.432
77.000
76.577
33.146
29.837
22.143
20.480
13.972
COOH
n-Bu
3ea
wxy-2-174
2018-07-11 09:07:06.843
USER: nmr
SOLVENT: CDCl3
Experiment = zgpg30
Pulse length = 9.500 usec
Relaxation delay = 2.000 sec
NA = 430
Solvent = CDCl3
PTS1d = 32768
F1 = 75.467751 MHz
F2 = 1.000000 MHz
SW1 = 22727.27 Hz
250
200
150
100
50
0
PPM

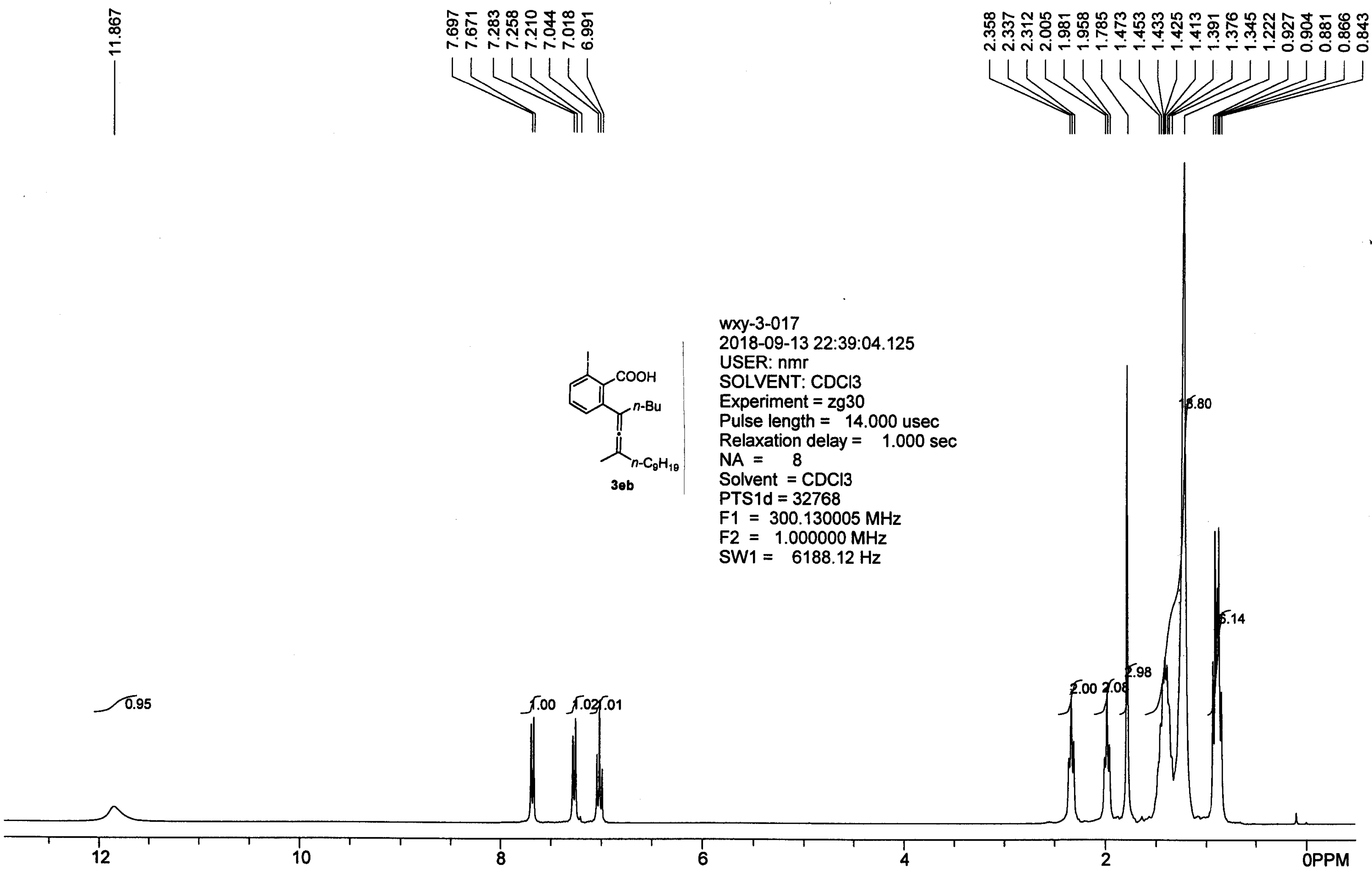
11.867
7.697
7.671
7.283
7.258
7.210
7.044
7.018
6.991
2.358
2.337
2.312
2.005
1.981
1.958
1.785
1.473
1.453
1.433
1.425
1.413
1.391
1.376
1.345
1.222
0.927
0.904
0.881
0.866
0.843
COOH
n-Bu
n-C_9H_{19}
3eb
wxy-3-017
2018-09-13 22:39:04.125
USER: nmr
SOLVENT: CDCl3
Experiment = zg30
Pulse length = 14.000 usec
Relaxation delay = 1.000 sec
NA = 8
Solvent = CDCl3
PTS1d = 32768
F1 = 300.130005 MHz
F2 = 1.000000 MHz
SW1 = 6188.12 Hz
0.95
1.00
1.02
1.01
2.00
2.08
2.98
6.14
12
10
8
6
4
2
0PPM

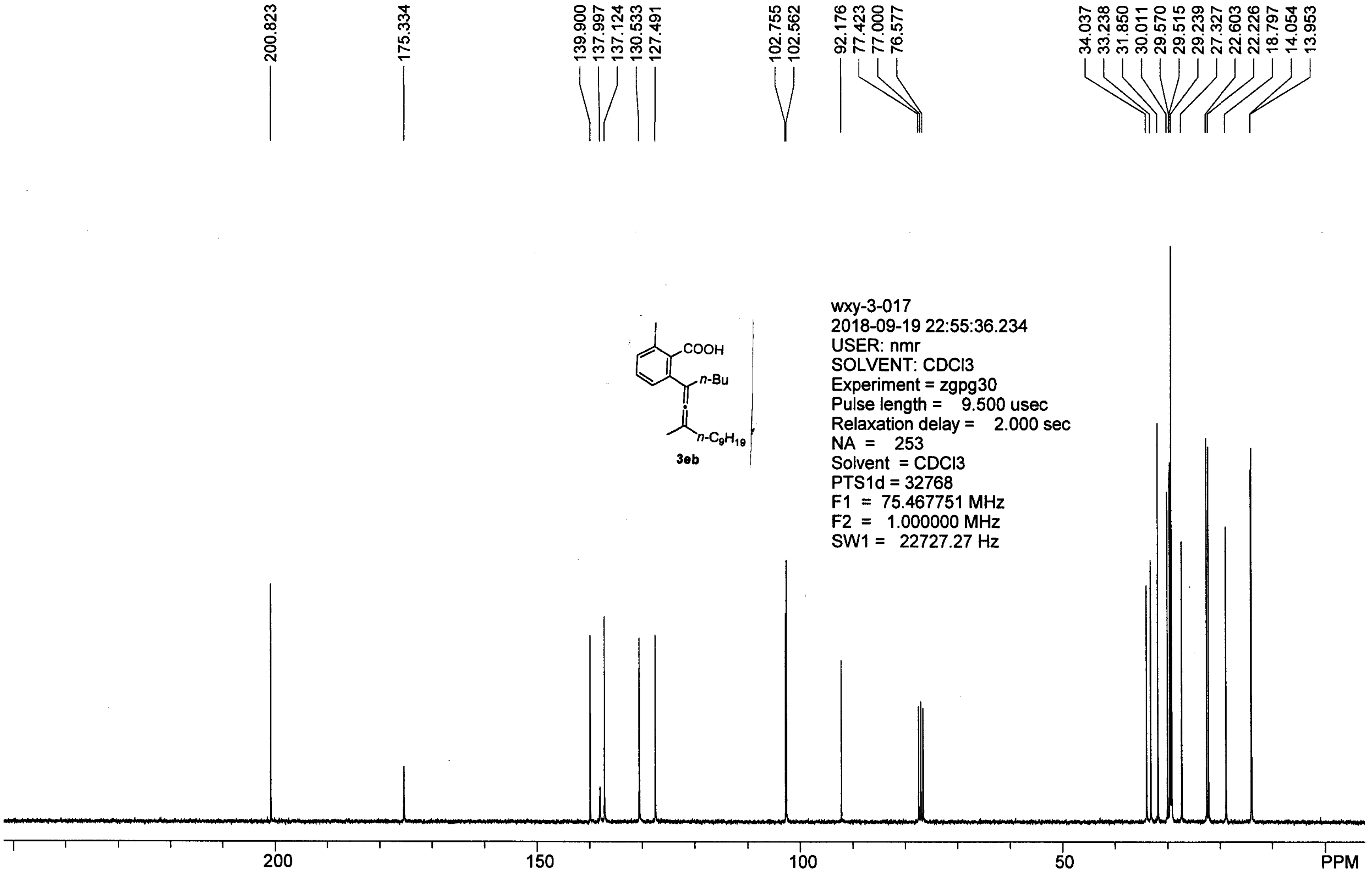
200.823
175.334
139.900
137.997
137.124
130.533
127.491
102.755
102.562
92.176
77.423
77.000
76.577
34.037
33.238
31.850
30.011
29.570
29.515
29.239
27.327
22.603
22.226
18.797
14.054
13.953
COOH
n-Bu
n-C9H19
3eb
wxy-3-017
2018-09-19 22:55:36.234
USER: nmr
SOLVENT: CDCl3
Experiment = zgpg30
Pulse length = 9.500 usec
Relaxation delay = 2.000 sec
NA = 253
Solvent = CDCl3
PTS1d = 32768
F1 = 75.467751 MHz
F2 = 1.000000 MHz
SW1 = 22727.27 Hz
200
150
100
50
PPM

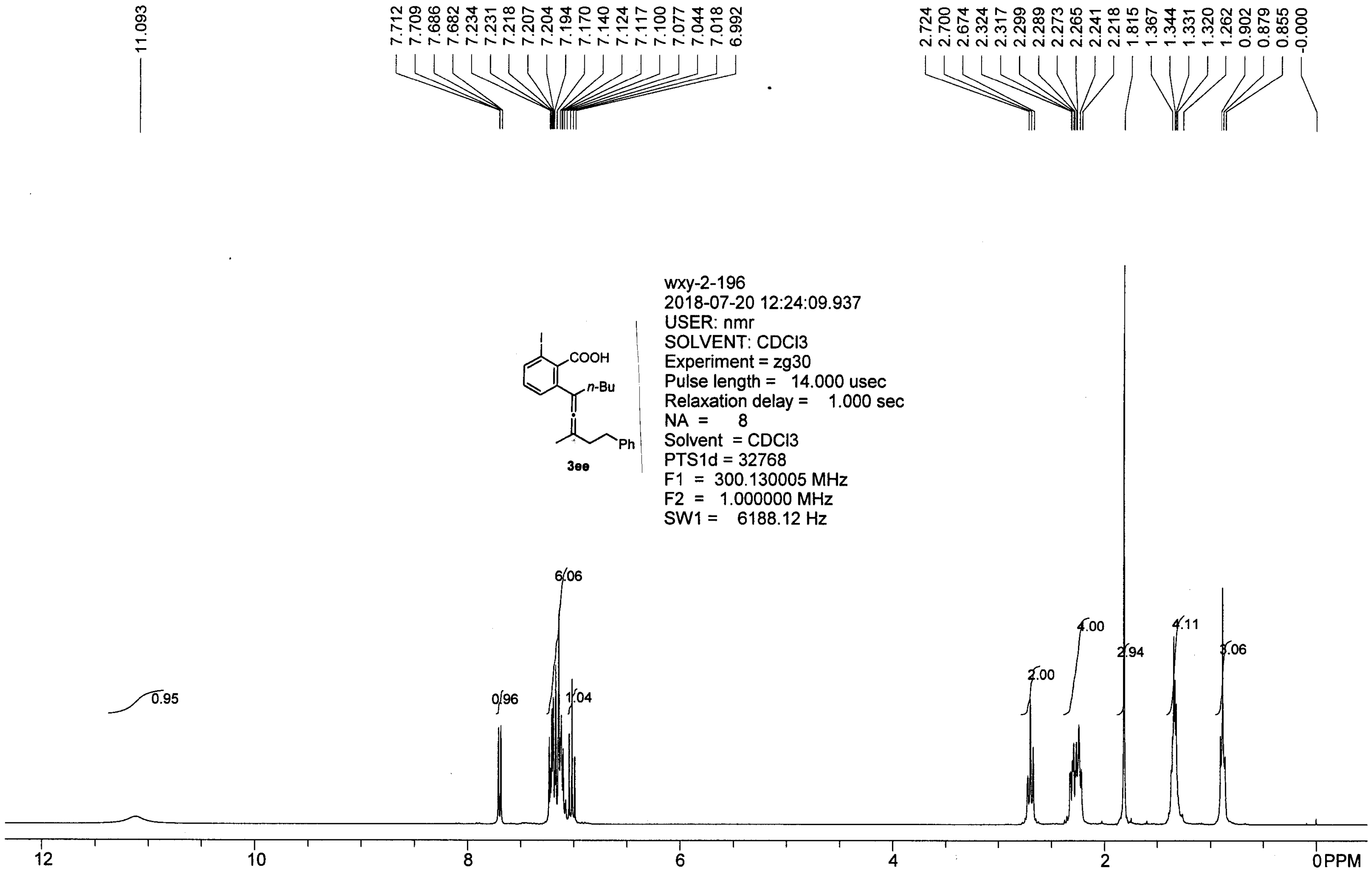
11.093
7.712
7.709
7.686
7.682
7.234
7.231
7.218
7.207
7.204
7.194
7.170
7.140
7.124
7.117
7.100
7.077
7.044
7.018
6.992
2.724
2.700
2.674
2.324
2.317
2.299
2.289
2.273
2.265
2.241
2.218
1.815
1.367
1.344
1.331
1.320
1.262
0.902
0.879
0.855
-0.000
wxy-2-196
2018-07-20 12:24:09.937
USER: nmr
SOLVENT: CDCl3
Experiment = zg30
Pulse length = 14.000 usec
Relaxation delay = 1.000 sec
NA = 8
Solvent = CDCl3
PTS1d = 32768
F1 = 300.130005 MHz
F2 = 1.000000 MHz
SW1 = 6188.12 Hz
COOH
n-Bu
Ph
3ee
0.95
0.96
6.06
1.04
2.00
4.00
2.94
4.11
3.06
12
10
8
6
4
2
0 PPM

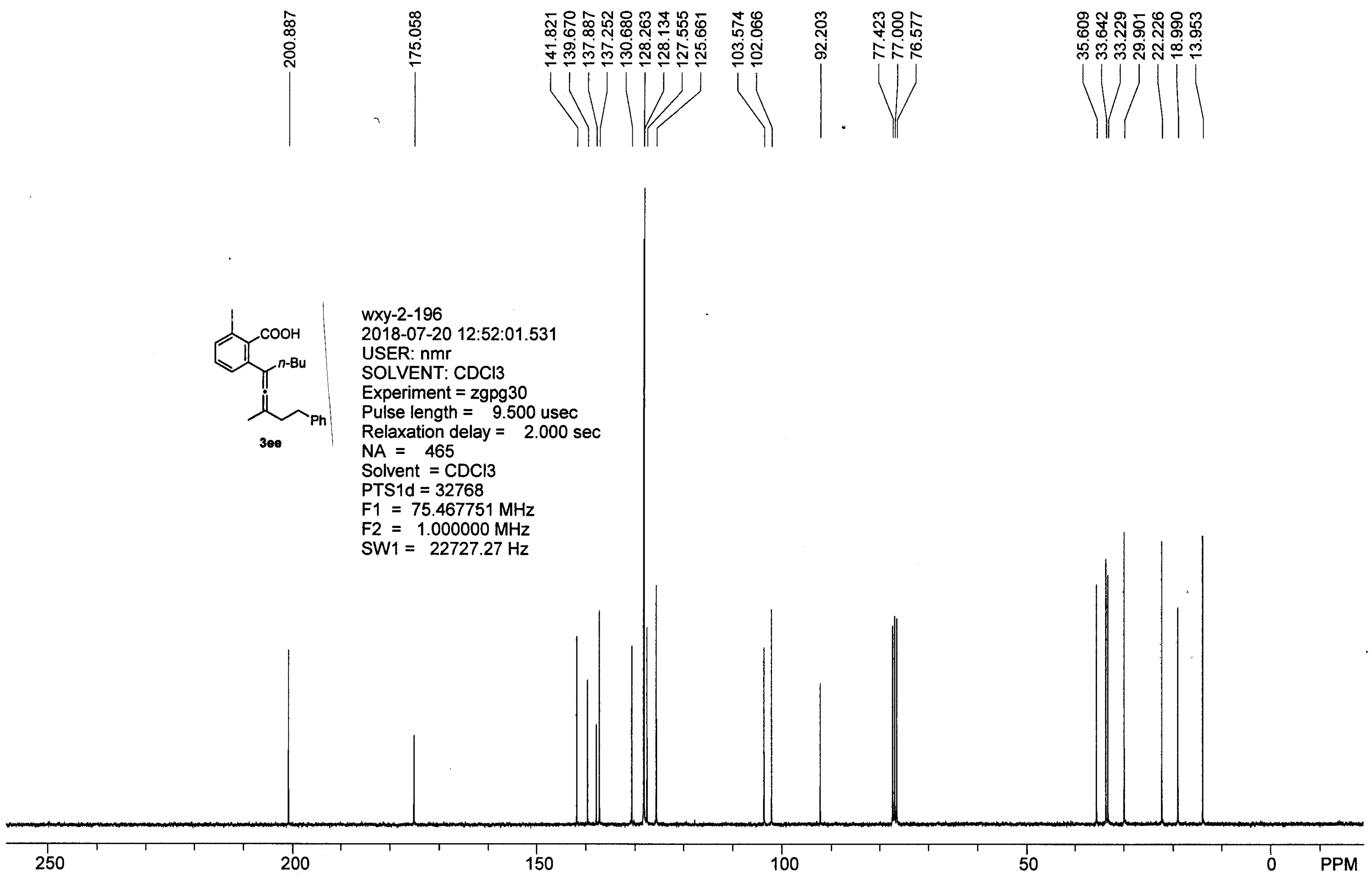
200.887
175.058
141.821
139.670
137.887
137.252
130.680
128.263
128.134
127.555
125.661
103.574
102.066
92.203
77.423
77.000
76.577
35.609
33.642
33.229
29.901
22.226
18.990
13.953
COOH
n-Bu
Ph
3ee
wxy-2-196
2018-07-20 12:52:01.531
USER: nmr
SOLVENT: CDCl3
Experiment = zgpg30
Pulse length = 9.500 usec
Relaxation delay = 2.000 sec
NA = 465
Solvent = CDCl3
PTS1d = 32768
F1 = 75.467751 MHz
F2 = 1.000000 MHz
SW1 = 22727.27 Hz
250
200
150
100
50
0
PPM

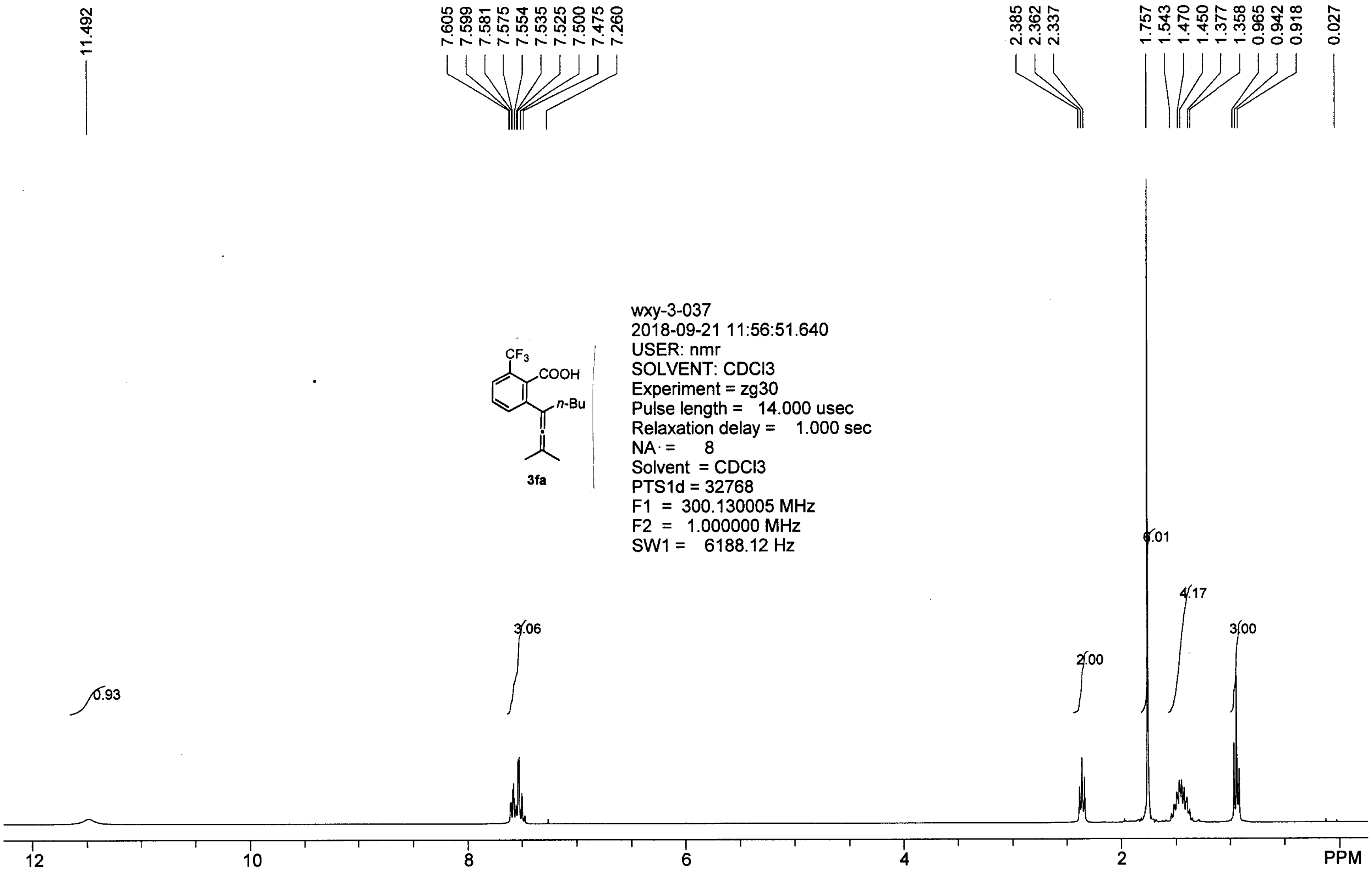

11.492
7.605
7.599
7.581
7.575
7.554
7.535
7.525
7.500
7.475
7.260
2.385
2.362
2.337
1.757
1.543
1.470
1.450
1.377
1.358
0.965
0.942
0.918
0.027
CF3
COOH
n-Bu
3fa
wxy-3-037
2018-09-21 11:56:51.640
USER: nmr
SOLVENT: CDCl3
Experiment = zg30
Pulse length = 14.000 usec
Relaxation delay = 1.000 sec
NA = 8
Solvent = CDCl3
PTS1d = 32768
F1 = 300.130005 MHz
F2 = 1.000000 MHz
SW1 = 6188.12 Hz
0.93
3.06
2.00
6.01
4.17
3.00
12
10
8
6
4
2
PPM

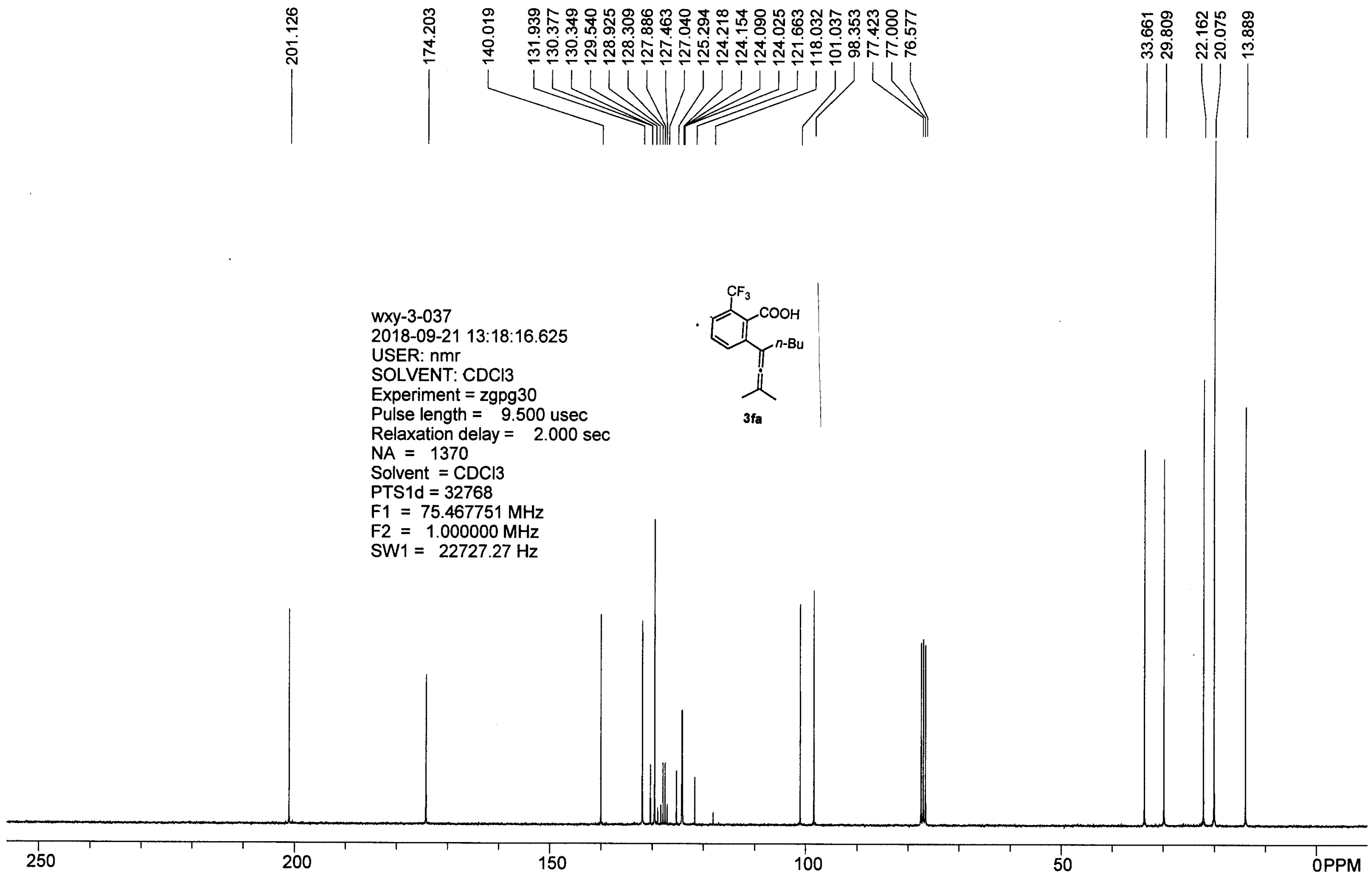
201.126
174.203
140.019
131.939
130.377
130.349
129.540
128.925
128.309
127.886
127.463
127.040
125.294
124.218
124.154
124.090
124.025
121.663
118.032
101.037
98.353
77.423
77.000
76.577
33.661
29.809
22.162
20.075
13.889
wxy-3-037
2018-09-21 13:18:16.625
USER: nmr
SOLVENT: CDCl3
Experiment = zgpg30
Pulse length = 9.500 usec
Relaxation delay = 2.000 sec
NA = 1370
Solvent = CDCl3
PTS1d = 32768
F1 = 75.467751 MHz
F2 = 1.000000 MHz
SW1 = 22727.27 Hz
CF3
COOH
n-Bu
3fa
250
200
150
100
50
0PPM

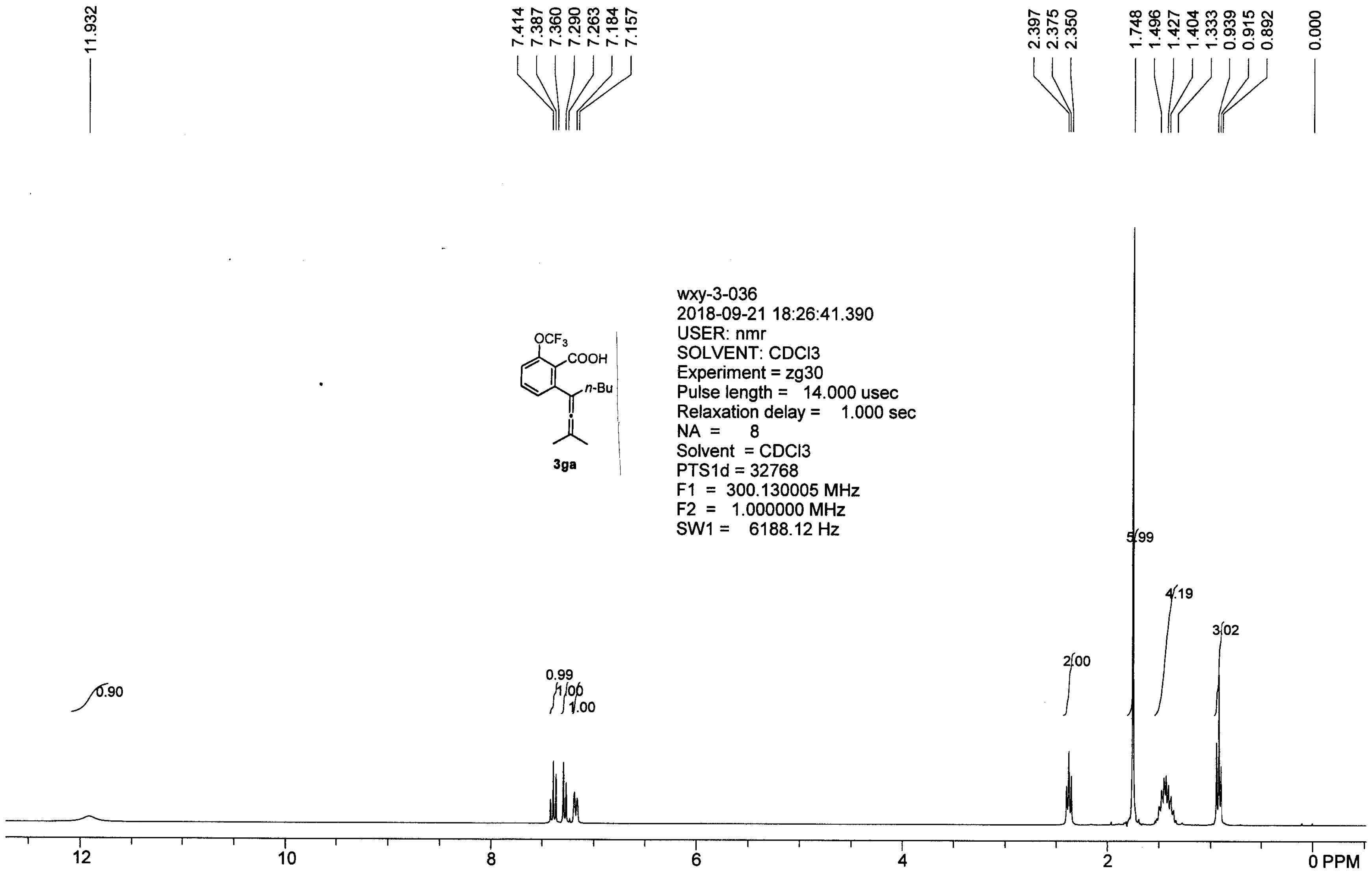
11.932
7.414
7.387
7.360
7.290
7.263
7.184
7.157
2.397
2.375
2.350
1.748
1.496
1.427
1.404
1.333
0.939
0.915
0.892
0.000
wxy-3-036
2018-09-21 18:26:41.390
USER: nmr
SOLVENT: CDCl3
Experiment = zg30
Pulse length = 14.000 usec
Relaxation delay = 1.000 sec
NA = 8
Solvent = CDCl3
PTS1d = 32768
F1 = 300.130005 MHz
F2 = 1.000000 MHz
SW1 = 6188.12 Hz
0.90
0.99
1.00
1.00
2.00
5.99
4.19
3.02
12
10
8
6
4
2
0 PPM
OCF3
COOH
n-Bu

3ga

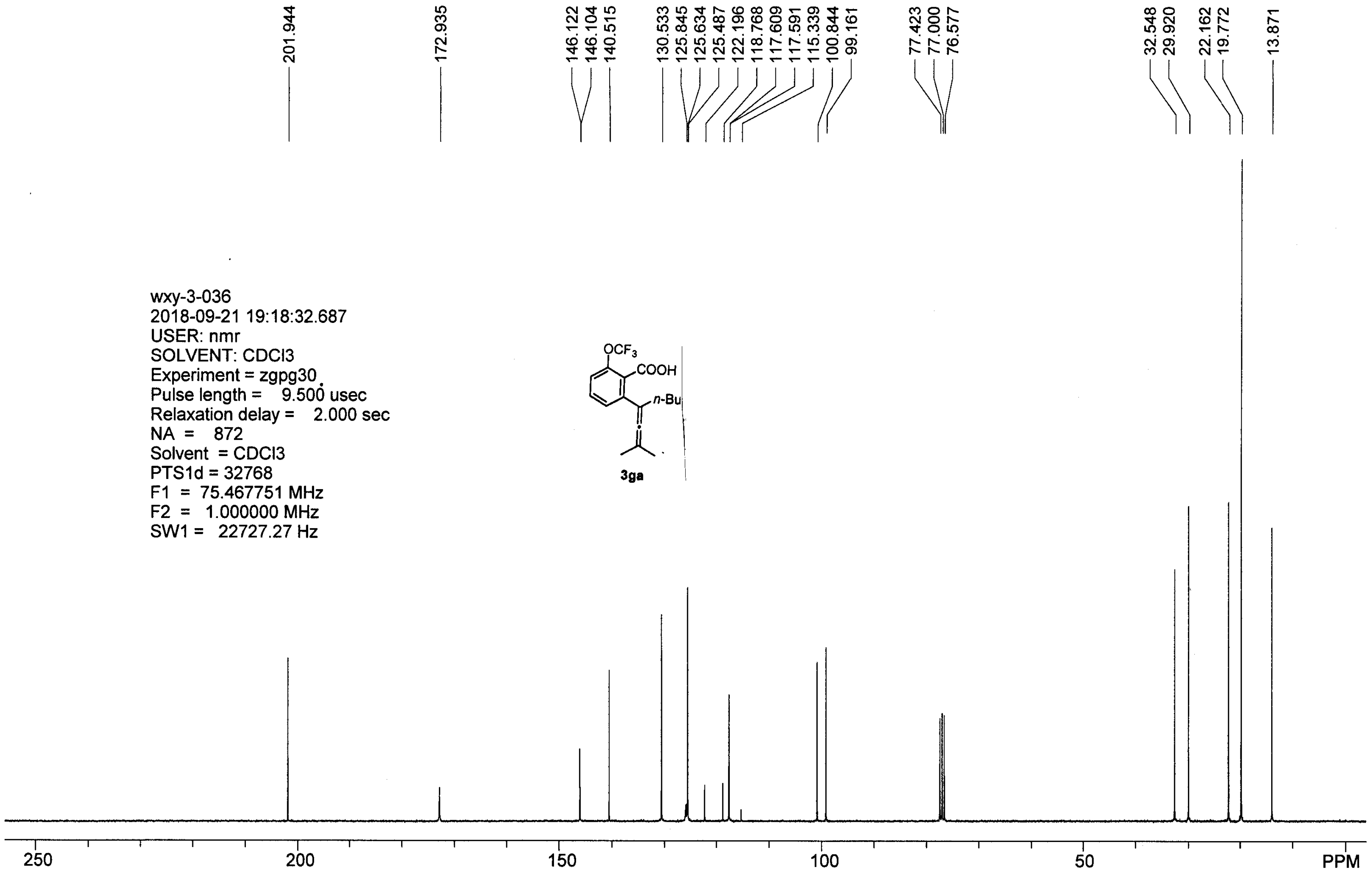
201.944
172.935
146.122
146.104
140.515
130.533
125.845
125.634
125.487
122.196
118.768
117.609
117.591
115.339
100.844
99.161
77.423
77.000
76.577
32.548
29.920
22.162
19.772
13.871
wxy-3-036
2018-09-21 19:18:32.687
USER: nmr
SOLVENT: CDCl3
Experiment = zgpg30
Pulse length = 9.500 usec
Relaxation delay = 2.000 sec
NA = 872
Solvent = CDCl3
PTS1d = 32768
F1 = 75.467751 MHz
F2 = 1.000000 MHz
SW1 = 22727.27 Hz
OCF3
COOH
n-Bu
3ga
250
200
150
100
50
PPM

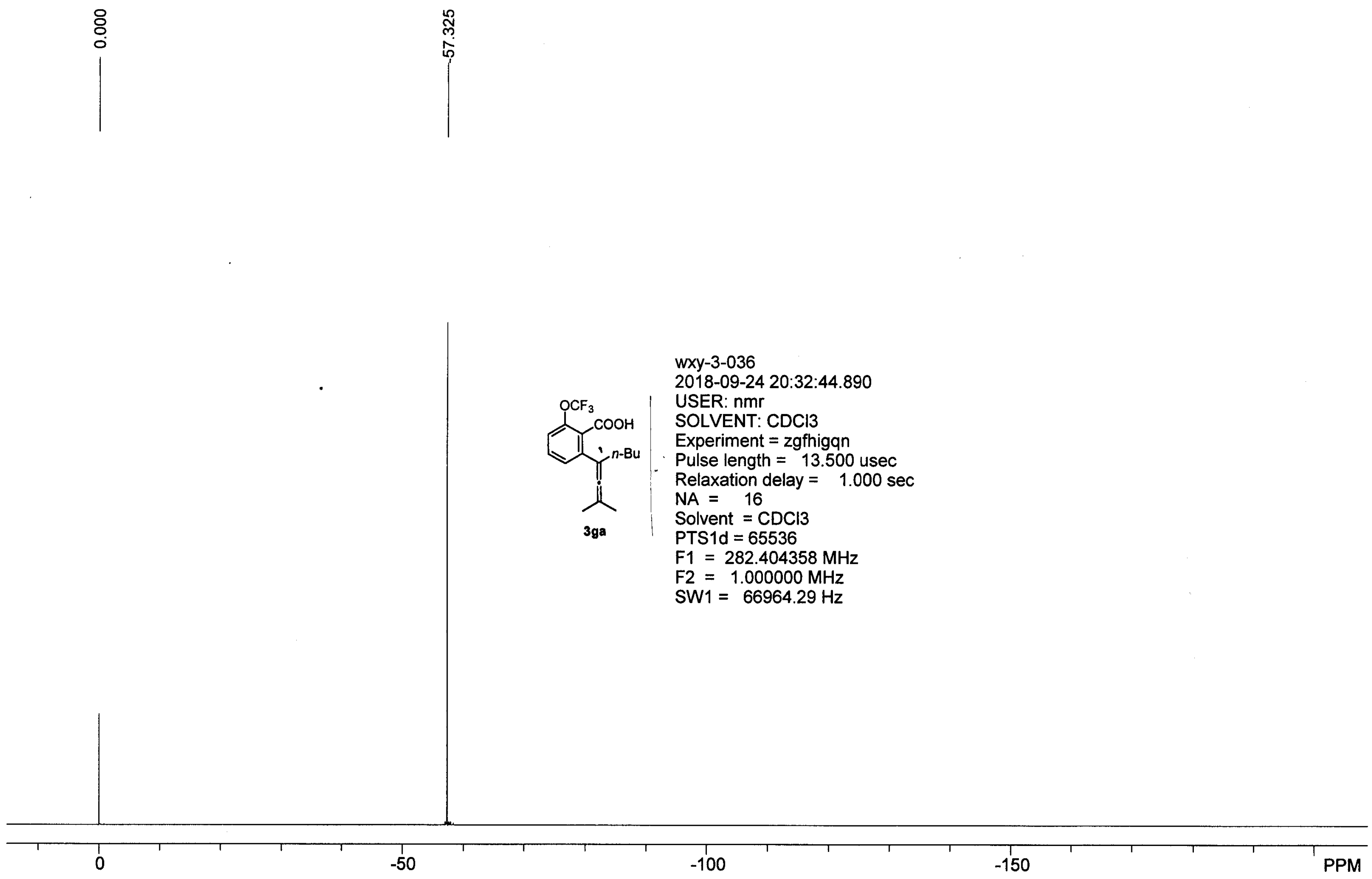

0.000
-57.325
OCF3
COOH
n-Bu
3ga
wxy-3-036
2018-09-24 20:32:44.890
USER: nmr
SOLVENT: CDCl3
Experiment = zgfhigqn
Pulse length = 13.500 usec
Relaxation delay = 1.000 sec
NA = 16
Solvent = CDCl3
PTS1d = 65536
F1 = 282.404358 MHz
F2 = 1.000000 MHz
SW1 = 66964.29 Hz
0
-50
-100
-150
PPM

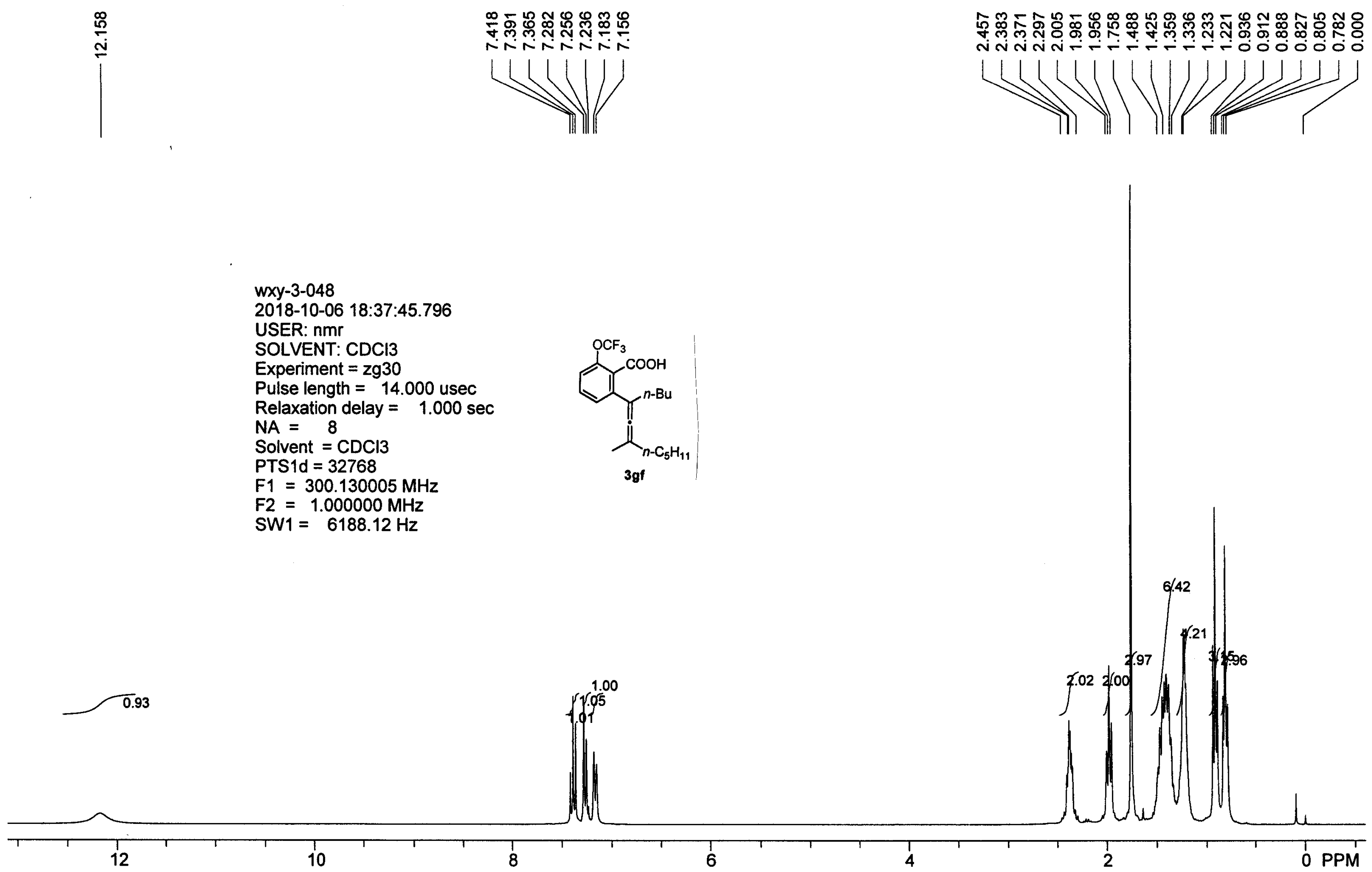
12.158
7.418
7.391
7.365
7.282
7.256
7.236
7.183
7.156
2.457
2.383
2.371
2.297
2.005
1.981
1.956
1.758
1.488
1.425
1.359
1.336
1.233
1.221
0.936
0.912
0.888
0.827
0.805
0.782
0.000
wxy-3-048
2018-10-06 18:37:45.796
USER: nmr
SOLVENT: CDCl3
Experiment = zg30
Pulse length = 14.000 usec
Relaxation delay = 1.000 sec
NA = 8
Solvent = CDCl3
PTS1d = 32768
F1 = 300.130005 MHz
F2 = 1.000000 MHz
SW1 = 6188.12 Hz
OCF3
COOH
n-Bu
n-C5H11
3gf
0.93
1.00
1.05
1.01
2.02
2.00
2.97
6.42
4.21
3.15
2.96
12
10
8
6
4
2
0 PPM

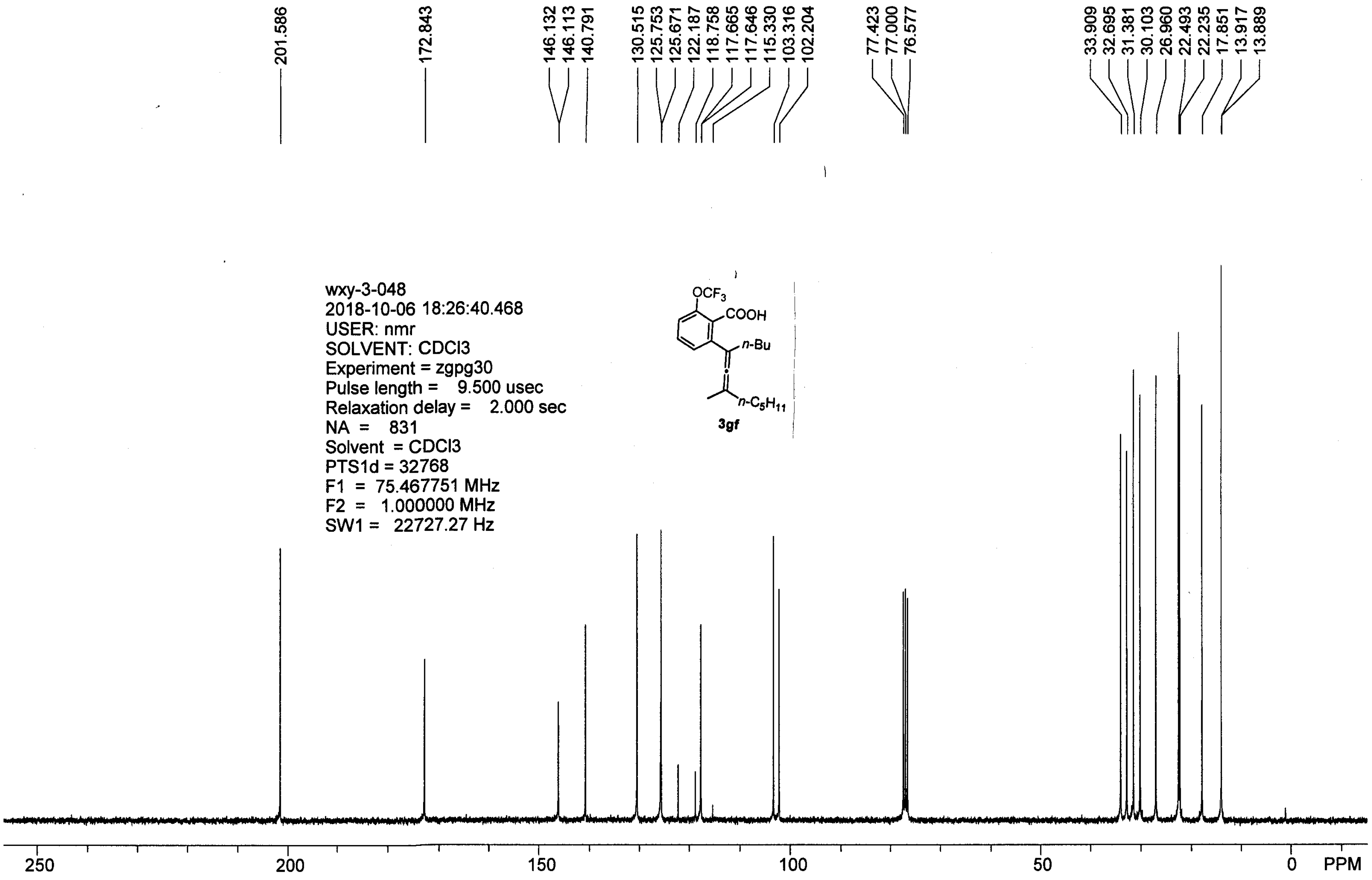
201.586
172.843
146.132
146.113
140.791
130.515
125.753
125.671
122.187
118.758
117.665
117.646
115.330
103.316
102.204
77.423
77.000
76.577
33.909
32.695
31.381
30.103
26.960
22.493
22.235
17.851
13.917
13.889
wxy-3-048
2018-10-06 18:26:40.468
USER: nmr
SOLVENT: CDCl3
Experiment = zgpg30
Pulse length = 9.500 usec
Relaxation delay = 2.000 sec
NA = 831
Solvent = CDCl3
PTS1d = 32768
F1 = 75.467751 MHz
F2 = 1.000000 MHz
SW1 = 22727.27 Hz
250
200
150
100
50
0
PPM
OCF3
COOH
n-Bu
n-C5H11

3gf

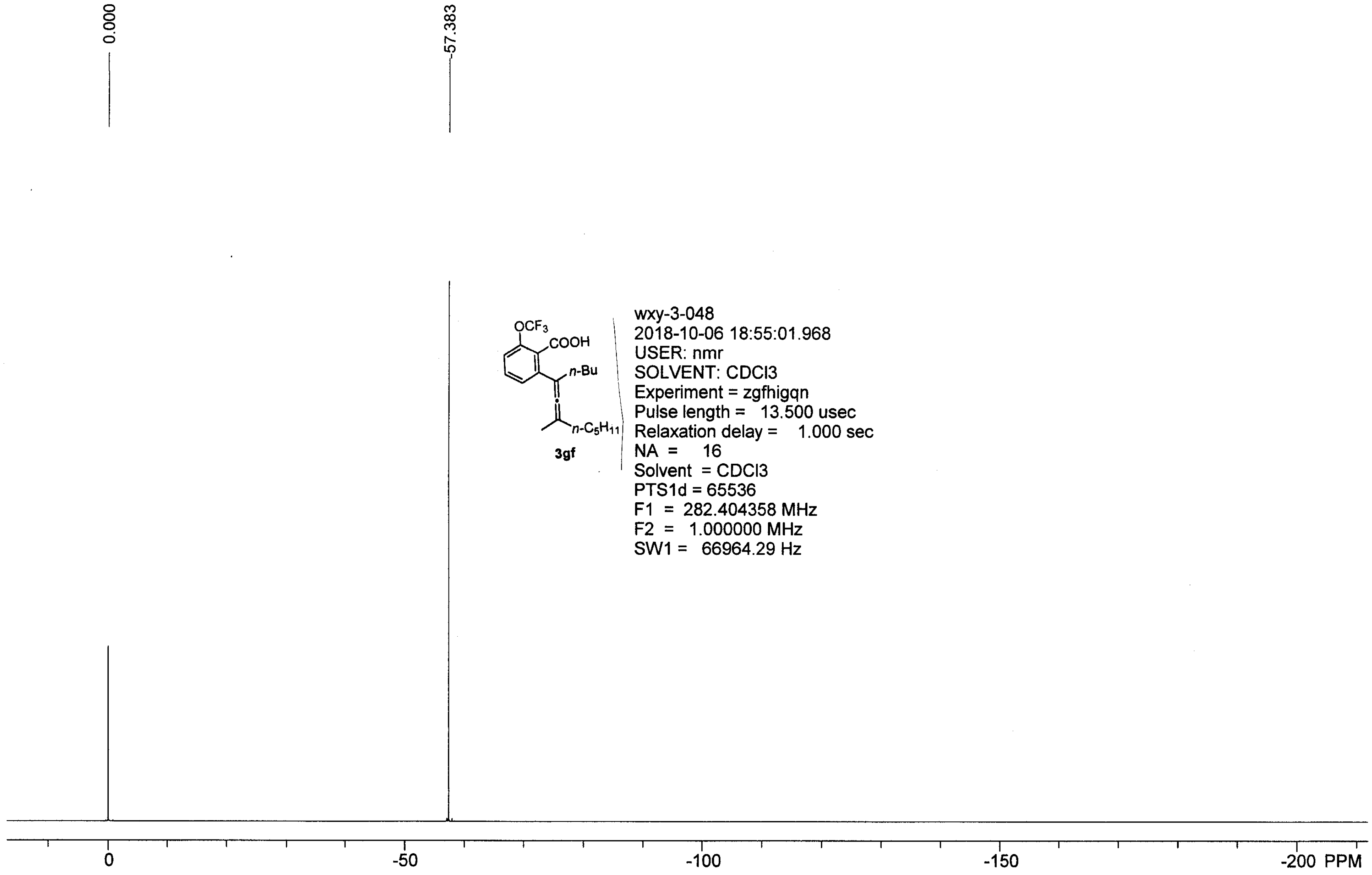
0.000
-57.383
OCF3
COOH
n-Bu
n-C5H11
3gf
wxy-3-048
2018-10-06 18:55:01.968
USER: nmr
SOLVENT: CDCl3
Experiment = zgfhigqn
Pulse length = 13.500 usec
Relaxation delay = 1.000 sec
NA = 16
Solvent = CDCl3
PTS1d = 65536
F1 = 282.404358 MHz
F2 = 1.000000 MHz
SW1 = 66964.29 Hz
0
-50
-100
-150
-200 PPM

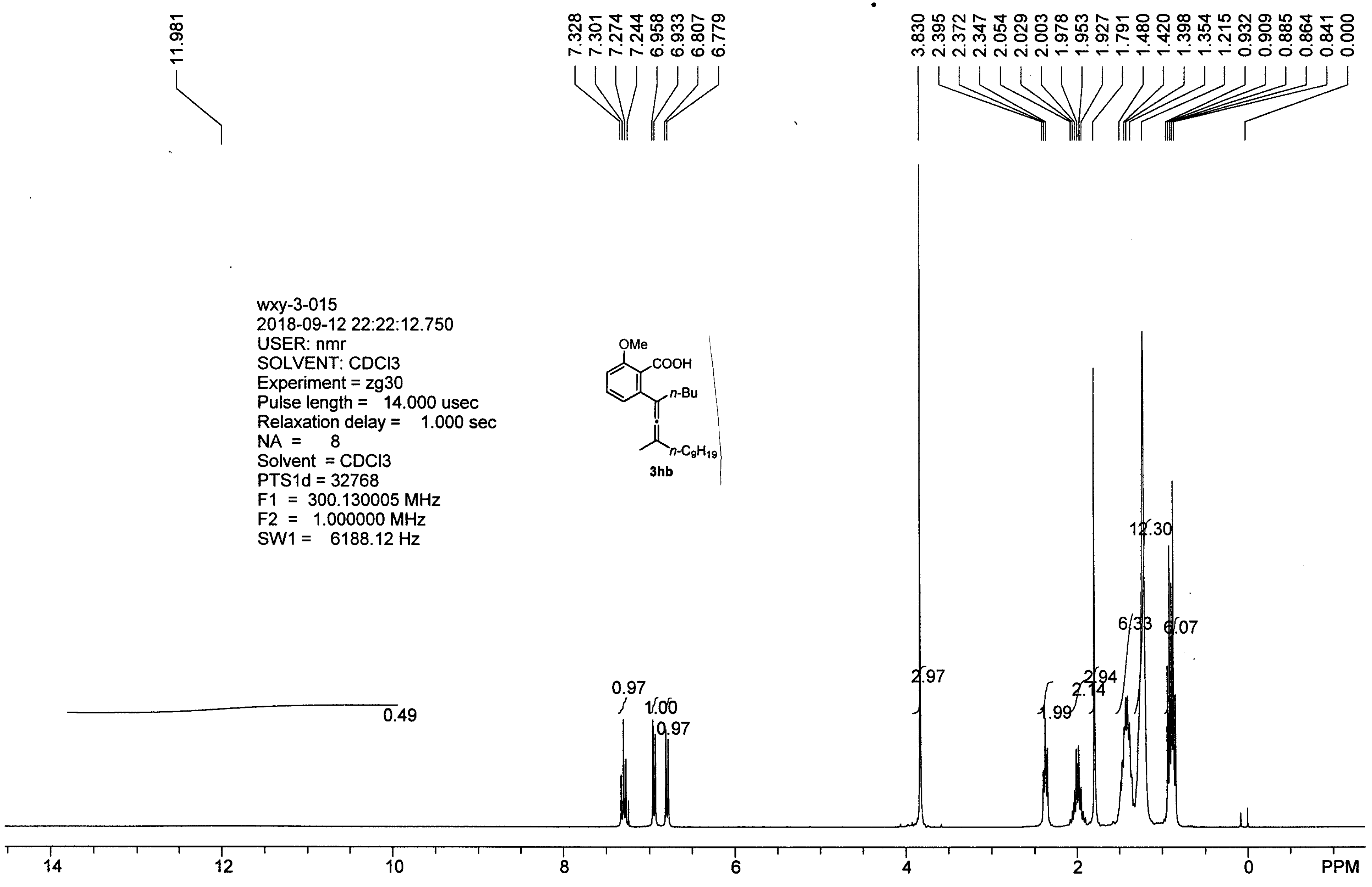
11.981
7.328
7.301
7.274
7.244
6.958
6.933
6.807
6.779
3.830
2.395
2.372
2.347
2.054
2.029
2.003
1.978
1.953
1.927
1.791
1.480
1.420
1.398
1.354
1.215
0.932
0.909
0.885
0.864
0.841
0.000
wxy-3-015
2018-09-12 22:22:12.750
USER: nmr
SOLVENT: CDCl3
Experiment = zg30
Pulse length = 14.000 usec
Relaxation delay = 1.000 sec
NA = 8
Solvent = CDCl3
PTS1d = 32768
F1 = 300.130005 MHz
F2 = 1.000000 MHz
SW1 = 6188.12 Hz
OMe
COOH
n-Bu
n-C9H19
3hb
0.49
0.97
1.00
0.97
2.97
1.99
2.14
2.94
6.33
12.30
6.07
14
12
10
8
6
4
2
0
PPM

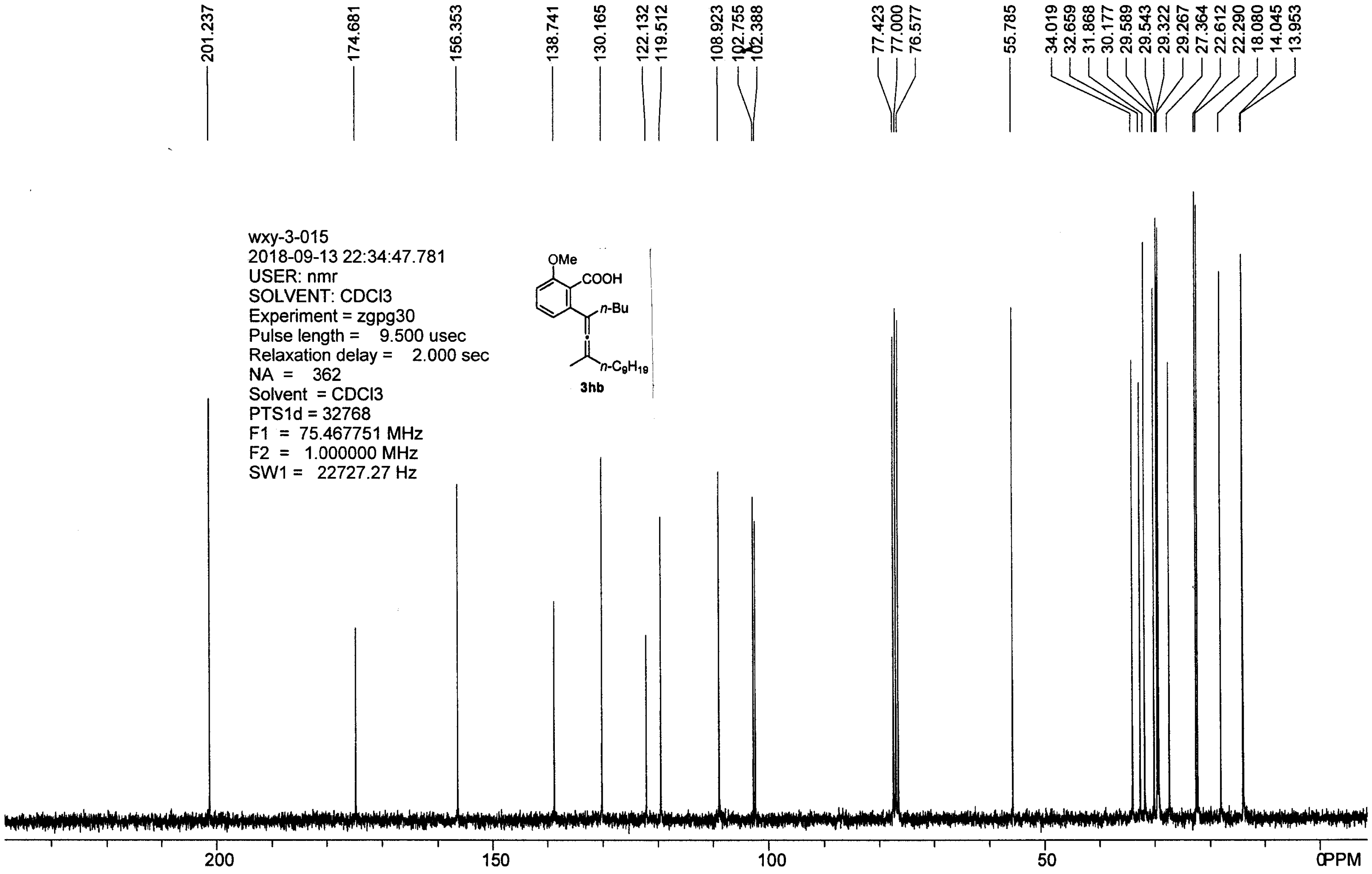
201.237
174.681
156.353
138.741
130.165
122.132
119.512
108.923
102.755
102.388
77.423
77.000
76.577
55.785
34.019
32.659
31.868
30.177
29.589
29.543
29.322
29.267
27.364
22.612
22.290
18.080
14.045
13.953
wxy-3-015
2018-09-13 22:34:47.781
USER: nmr
SOLVENT: CDCl3
Experiment = zgpg30
Pulse length = 9.500 usec
Relaxation delay = 2.000 sec
NA = 362
Solvent = CDCl3
PTS1d = 32768
F1 = 75.467751 MHz
F2 = 1.000000 MHz
SW1 = 22727.27 Hz
OMe
COOH
n-Bu
n-C9H19
3hb
200
150
100
50
0PPM

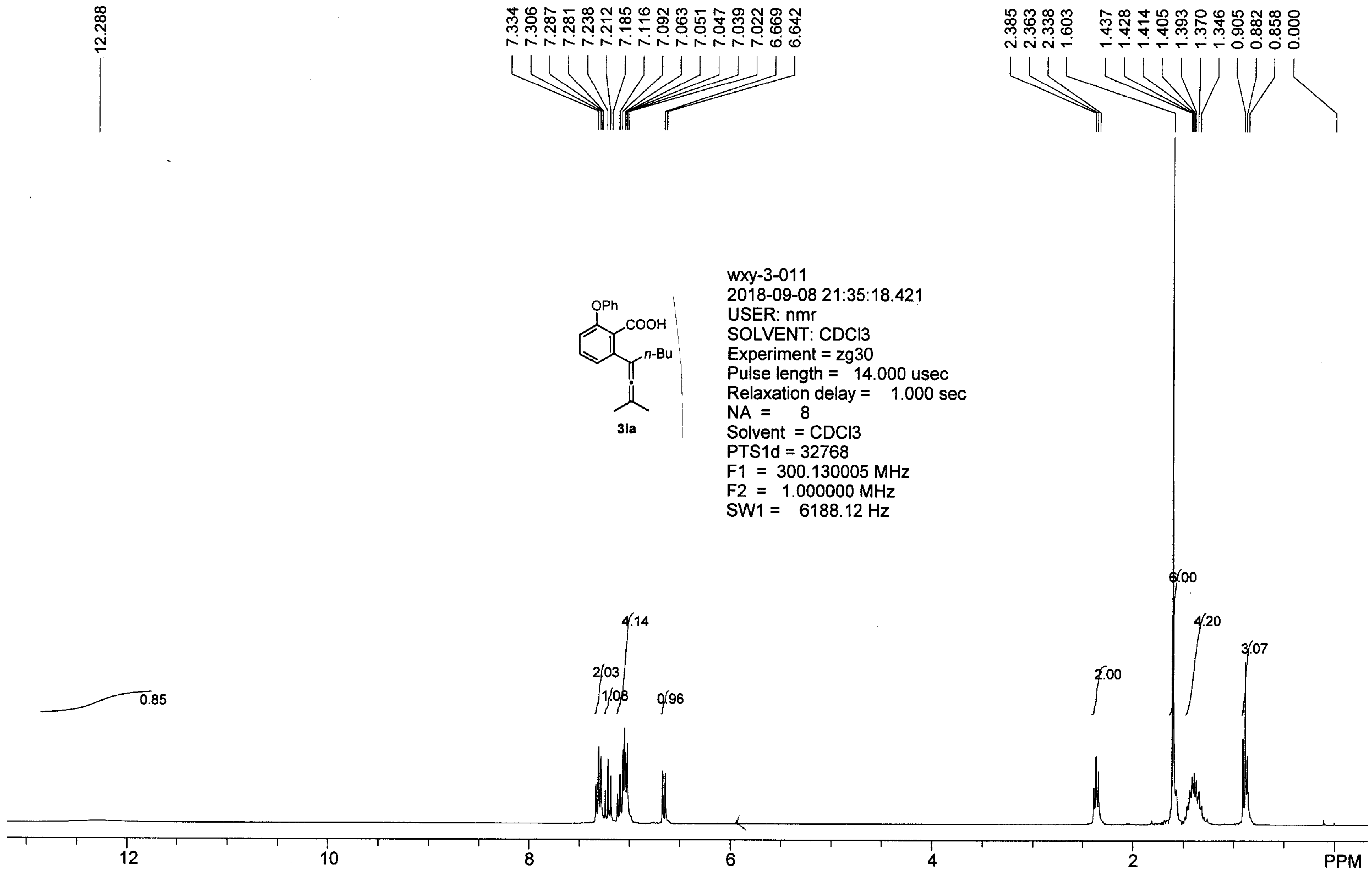
12.288
7.334
7.306
7.287
7.281
7.238
7.212
7.185
7.116
7.092
7.063
7.051
7.047
7.039
7.022
6.669
6.642
2.385
2.363
2.338
1.603
1.437
1.428
1.414
1.405
1.393
1.370
1.346
0.905
0.882
0.858
0.000
OPh
COOH
n-Bu
3la
wxy-3-011
2018-09-08 21:35:18.421
USER: nmr
SOLVENT: CDCl3
Experiment = zg30
Pulse length = 14.000 usec
Relaxation delay = 1.000 sec
NA = 8
Solvent = CDCl3
PTS1d = 32768
F1 = 300.130005 MHz
F2 = 1.000000 MHz
SW1 = 6188.12 Hz
0.85
2.03
1.08
4.14
0.96
2.00
6.00
4.20
3.07
12
10
8
6
4
2
PPM

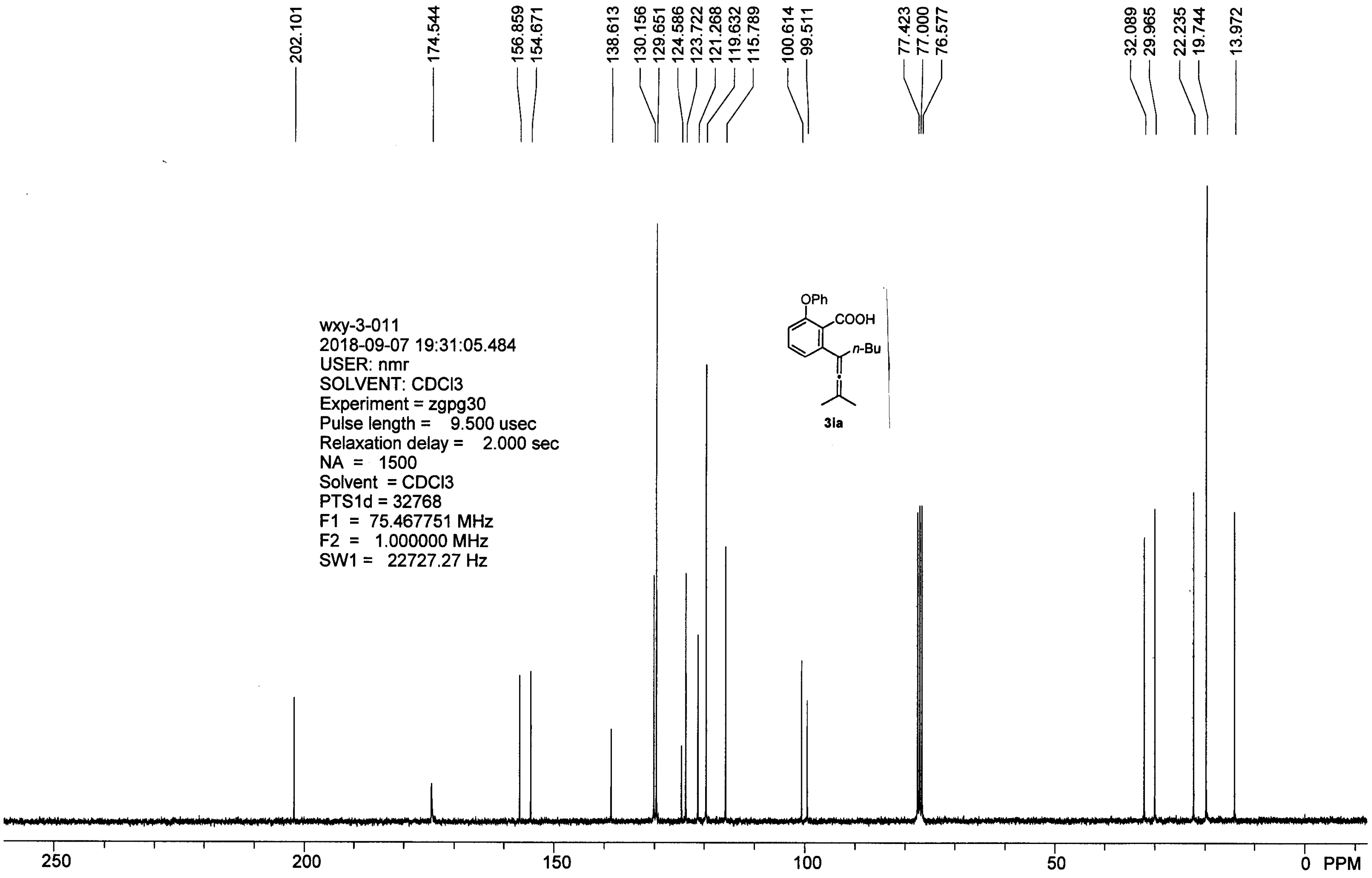
202.101
174.544
156.859
154.671
138.613
130.156
129.651
124.586
123.722
121.268
119.632
115.789
100.614
99.511
77.423
77.000
76.577
32.089
29.965
22.235
19.744
13.972
wxy-3-011
2018-09-07 19:31:05.484
USER: nmr
SOLVENT: CDCl3
Experiment = zgpg30
Pulse length = 9.500 usec
Relaxation delay = 2.000 sec
NA = 1500
Solvent = CDCl3
PTS1d = 32768
F1 = 75.467751 MHz
F2 = 1.000000 MHz
SW1 = 22727.27 Hz
OPh
COOH
n-Bu
3la
250
200
150
100
50
0 PPM

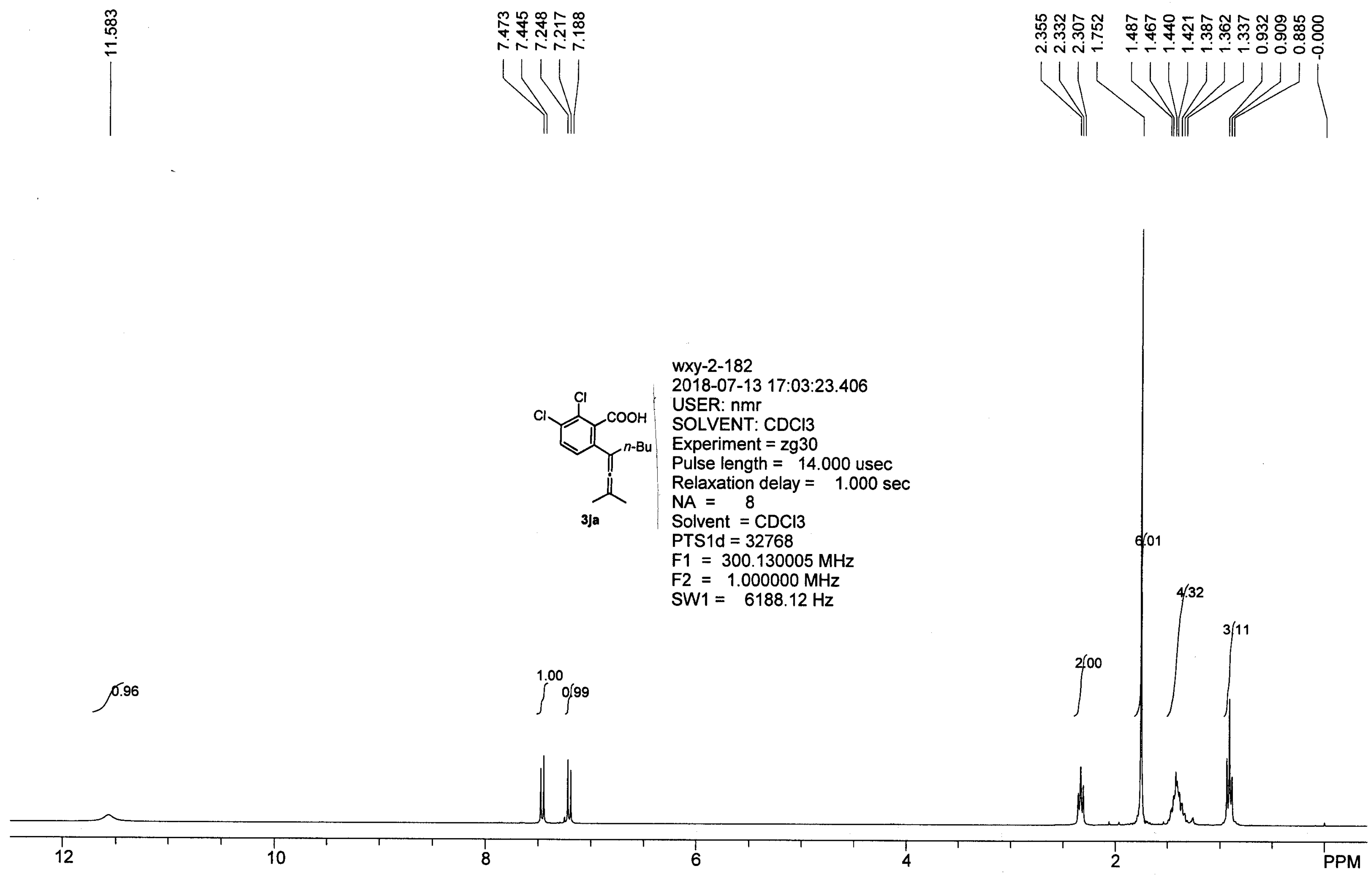
11.583
7.473
7.445
7.248
7.217
7.188
2.355
2.332
2.307
1.752
1.487
1.467
1.440
1.421
1.387
1.362
1.337
0.932
0.909
0.885
-0.000
wxy-2-182
2018-07-13 17:03:23.406
USER: nmr
SOLVENT: CDCl3
Experiment = zg30
Pulse length = 14.000 usec
Relaxation delay = 1.000 sec
NA = 8
Solvent = CDCl3
PTS1d = 32768
F1 = 300.130005 MHz
F2 = 1.000000 MHz
SW1 = 6188.12 Hz
Cl
Cl
COOH
n-Bu
3ja
0.96
1.00
0.99
2.00
6.01
4.32
3.11
12
10
8
6
4
2
PPM

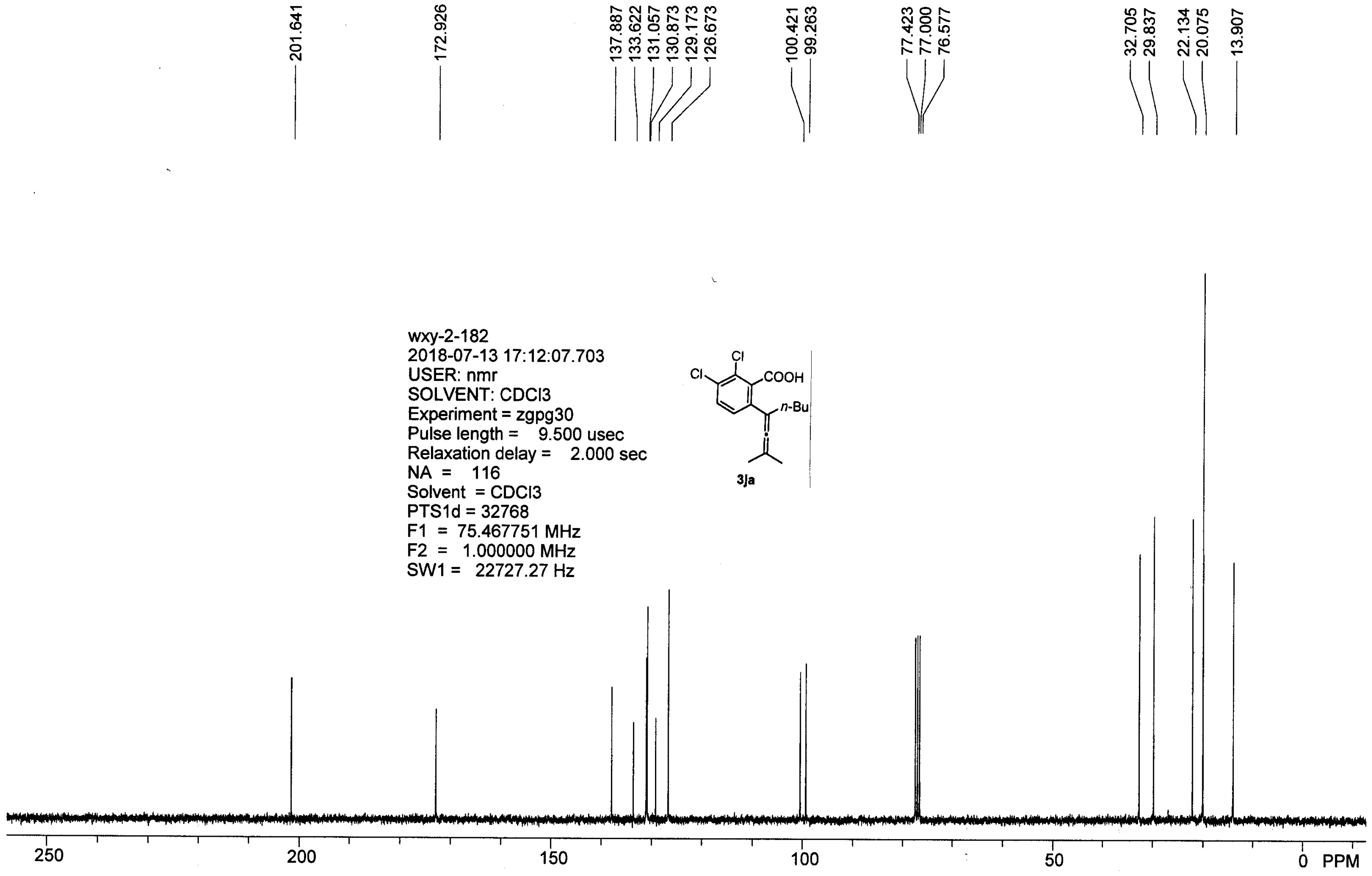
201.641
172.926
137.887
133.622
131.057
130.873
129.173
126.673
100.421
99.263
77.423
77.000
76.577
32.705
29.837
22.134
20.075
13.907
wxy-2-182
2018-07-13 17:12:07.703
USER: nmr
SOLVENT: CDCl3
Experiment = zgpg30
Pulse length = 9.500 usec
Relaxation delay = 2.000 sec
NA = 116
Solvent = CDCl3
PTS1d = 32768
F1 = 75.467751 MHz
F2 = 1.000000 MHz
SW1 = 22727.27 Hz
Cl
Cl
COOH
n-Bu
3ja
250
200
150
100
50
0 PPM

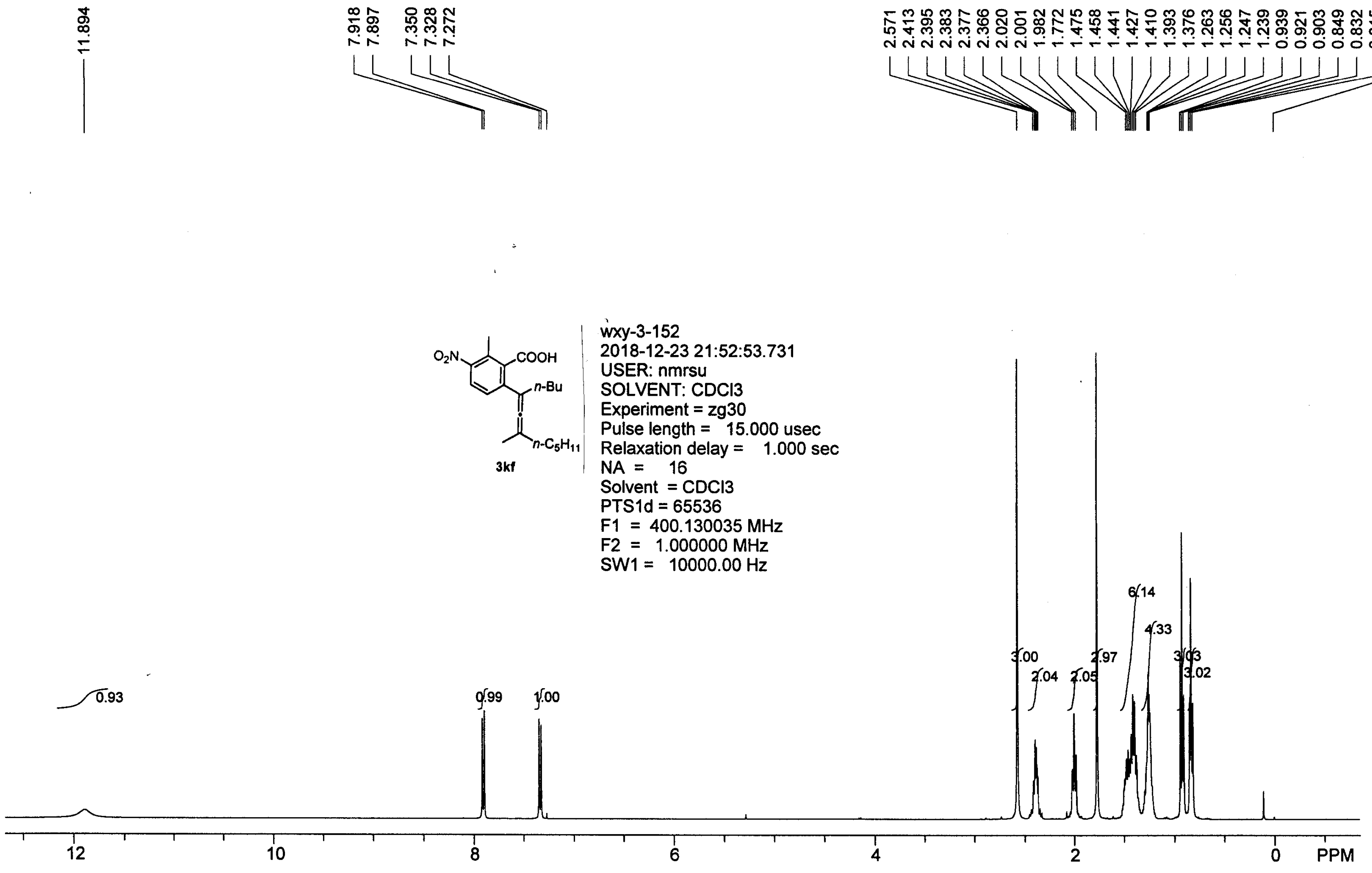
11.894
7.918
7.897
7.350
7.328
7.272
2.571
2.413
2.395
2.383
2.377
2.366
2.020
2.001
1.982
1.772
1.475
1.458
1.441
1.427
1.410
1.393
1.376
1.263
1.256
1.247
1.239
0.939
0.921
0.903
0.849
0.832
0.815
[illegible]
O_2N
COOH
n-Bu
n-C_5H_{11}
3kf
wxy-3-152
2018-12-23 21:52:53.731
USER: nmrsu
SOLVENT: CDCl3
Experiment = zg30
Pulse length = 15.000 usec
Relaxation delay = 1.000 sec
NA = 16
Solvent = CDCl3
PTS1d = 65536
F1 = 400.130035 MHz
F2 = 1.000000 MHz
SW1 = 10000.00 Hz
0.93
0.99
1.00
3.00
2.04
2.05
2.97
6.14
4.33
3.03
3.02
12
10
8
6
4
2
0
PPM

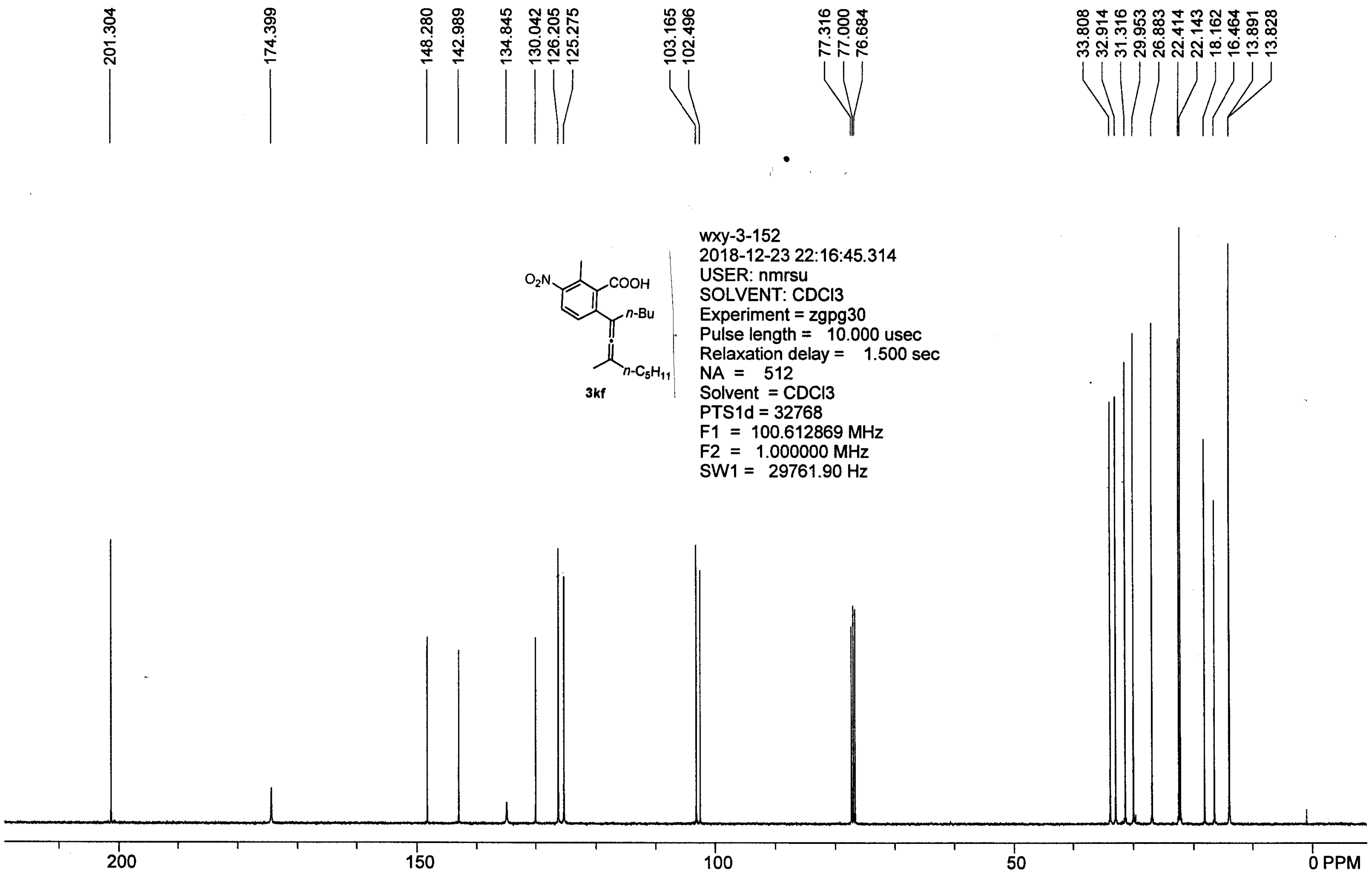
201.304
174.399
148.280
142.989
134.845
130.042
126.205
125.275
103.165
102.496
77.316
77.000
76.684
33.808
32.914
31.316
29.953
26.883
22.414
22.143
18.162
16.464
13.891
13.828
O_2N
COOH
n-Bu
n-C_5H_{11}
3kf
wxy-3-152
2018-12-23 22:16:45.314
USER: nmrsu
SOLVENT: CDCl3
Experiment = zgpg30
Pulse length = 10.000 usec
Relaxation delay = 1.500 sec
NA = 512
Solvent = CDCl3
PTS1d = 32768
F1 = 100.612869 MHz
F2 = 1.000000 MHz
SW1 = 29761.90 Hz
200
150
100
50
0 PPM

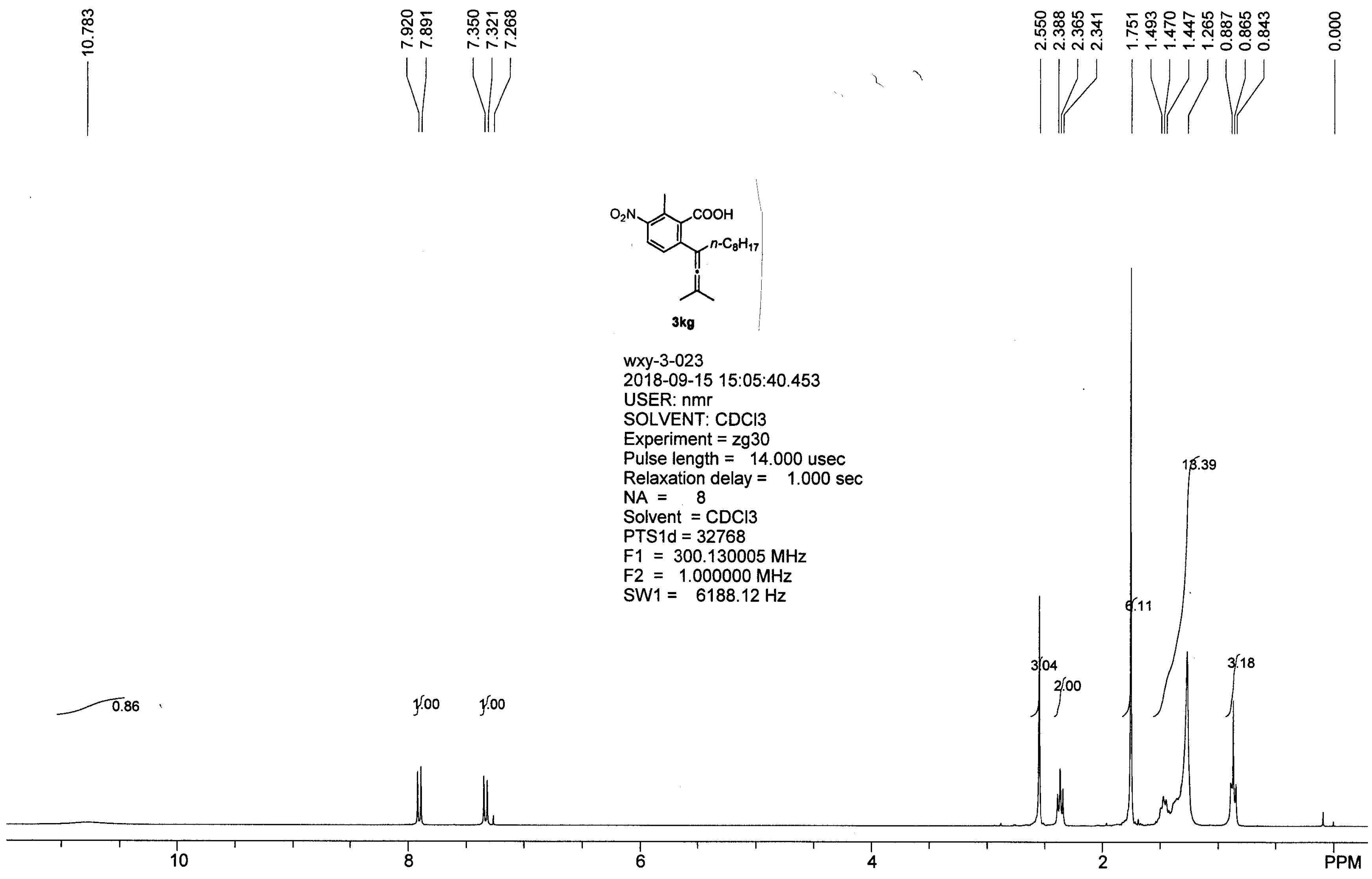

3kg

wxy-3-023
2018-09-15 15:05:40.453
USER: nmr
SOLVENT: CDCl3
Experiment = zg30
Pulse length = 14.000 usec
Relaxation delay = 1.000 sec
NA = 8
Solvent = CDCl3
PTS1d = 32768
F1 = 300.130005 MHz
F2 = 1.000000 MHz
SW1 = 6188.12 Hz

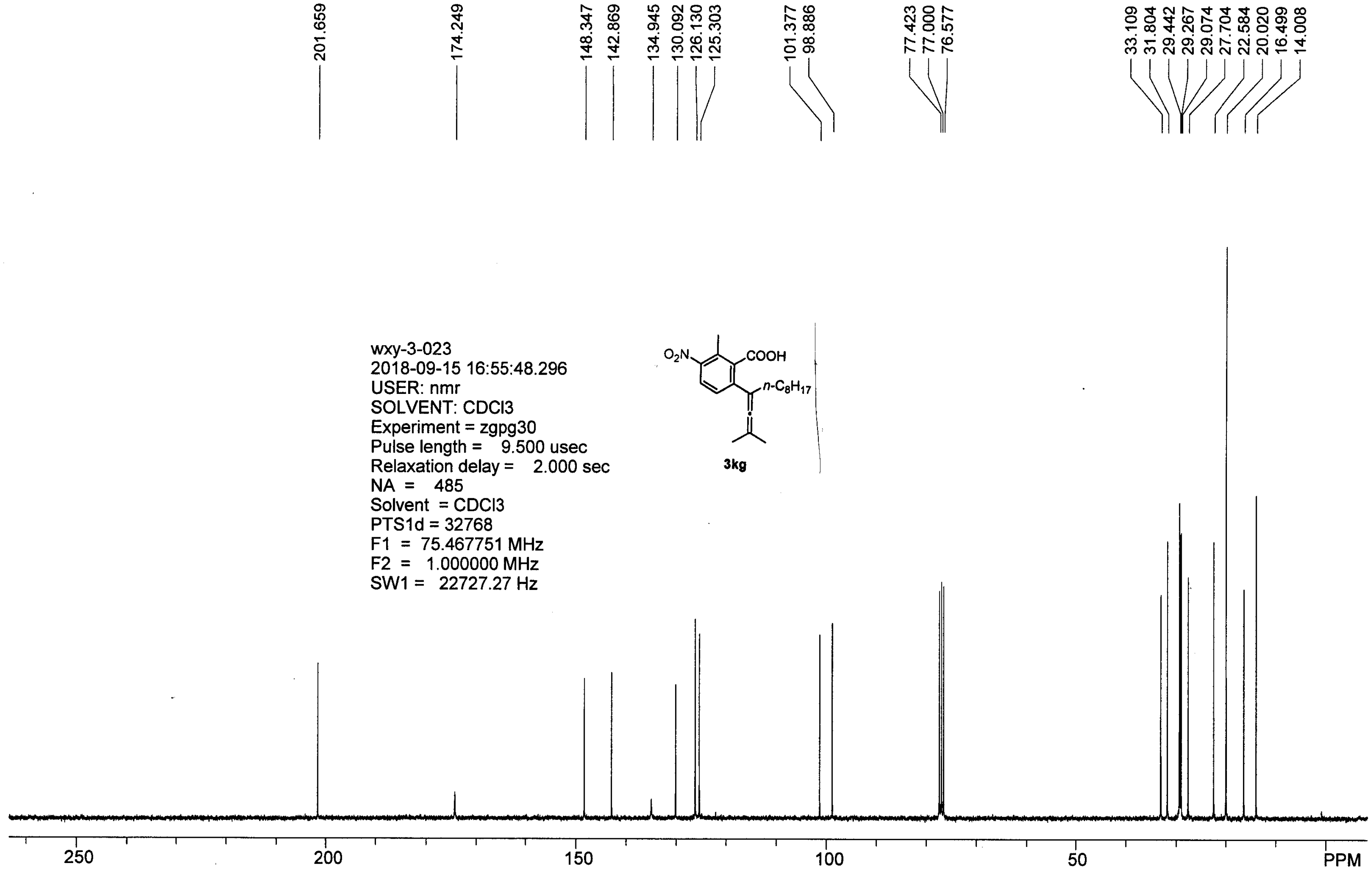
201.659
174.249
148.347
142.869
134.945
130.092
126.130
125.303
101.377
98.886
77.423
77.000
76.577
33.109
31.804
29.442
29.267
29.074
27.704
22.584
20.020
16.499
14.008
wxy-3-023
2018-09-15 16:55:48.296
USER: nmr
SOLVENT: CDCl3
Experiment = zgpg30
Pulse length = 9.500 usec
Relaxation delay = 2.000 sec
NA = 485
Solvent = CDCl3
PTS1d = 32768
F1 = 75.467751 MHz
F2 = 1.000000 MHz
SW1 = 22727.27 Hz
O_2N
COOH
n-C_8H_{17}
3kg
250
200
150
100
50
PPM

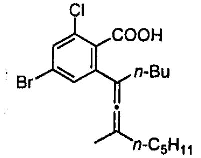

3lf

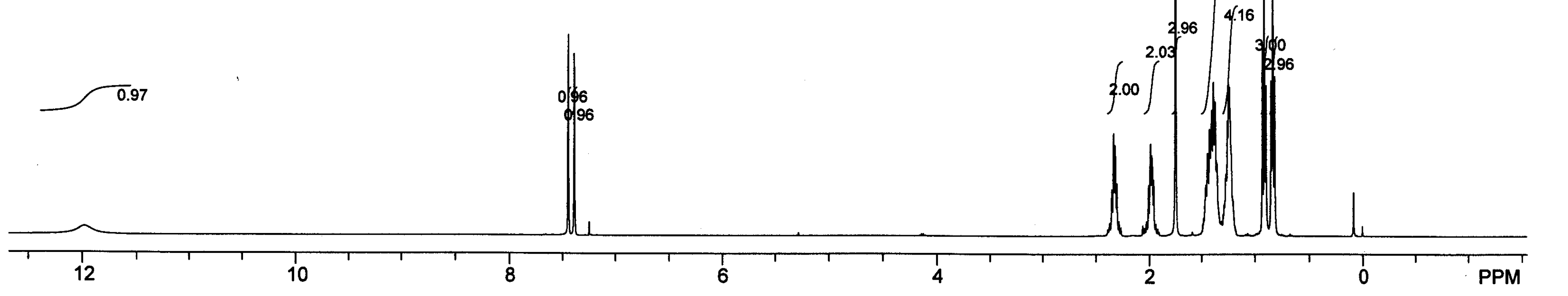

wxy-3-153
2018-12-24 05:01:23.294
USER: nmrsu
SOLVENT: CDCl3
Experiment = zg30
Pulse length = 15.000 usec
Relaxation delay = 1.000 sec
NA = 16
Solvent = CDCl3
PTS1d = 65536
F1 = 400.130035 MHz
F2 = 1.000000 MHz
SW1 = 10000.00 Hz

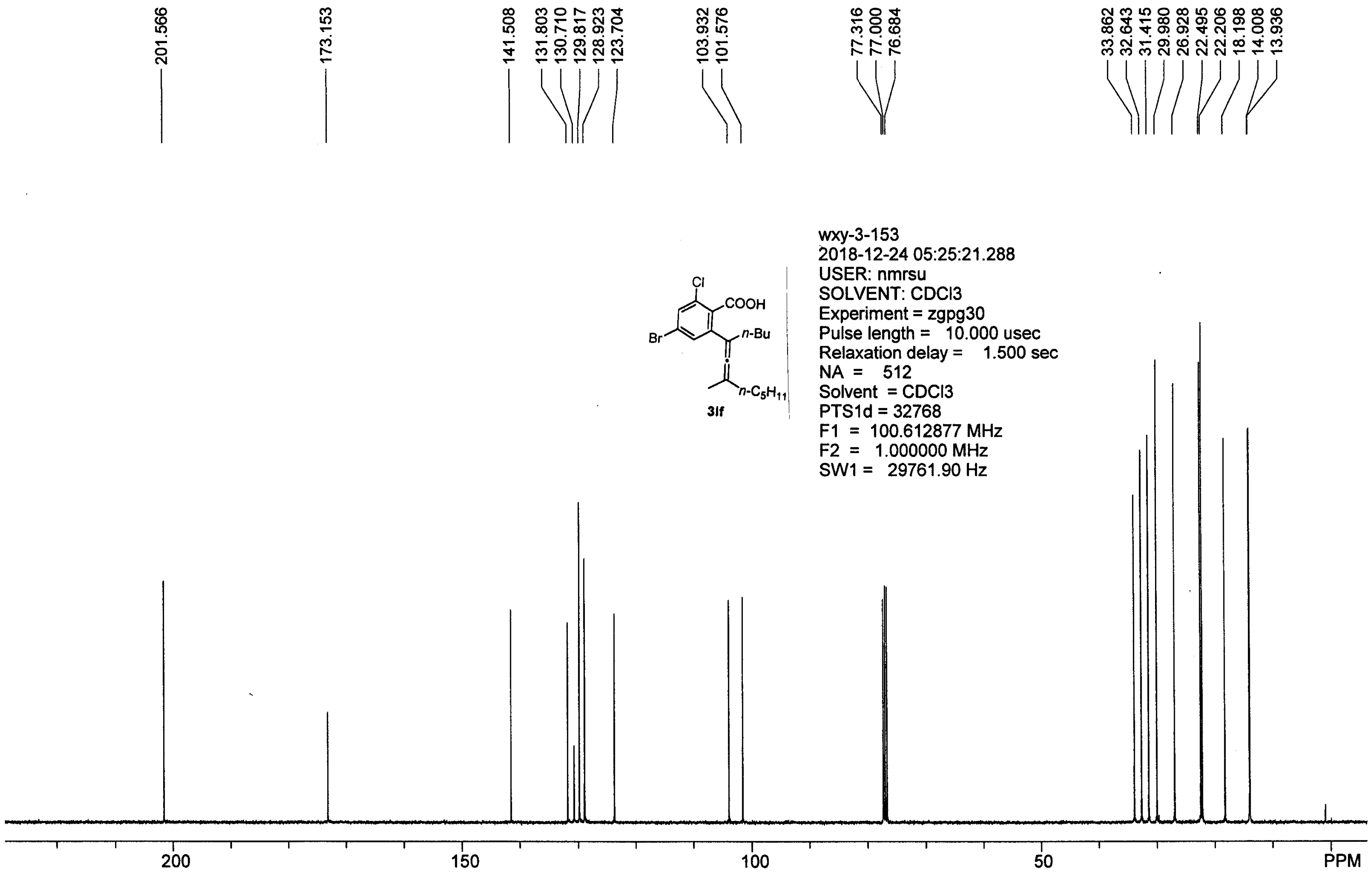

3lf

201.566
173.153
141.508
131.803
130.710
129.817
128.923
123.704
103.932
101.576
77.316
77.000
76.684
33.862
32.643
31.415
29.980
26.928
22.495
22.206
18.198
14.008
13.936
wxy-3-153
2018-12-24 05:25:21.288
USER: nmrsu
SOLVENT: CDCl3
Experiment = zgpg30
Pulse length = 10.000 usec
Relaxation delay = 1.500 sec
NA = 512
Solvent = CDCl3
PTS1d = 32768
F1 = 100.612877 MHz
F2 = 1.000000 MHz
SW1 = 29761.90 Hz
200
150
100
50
PPM

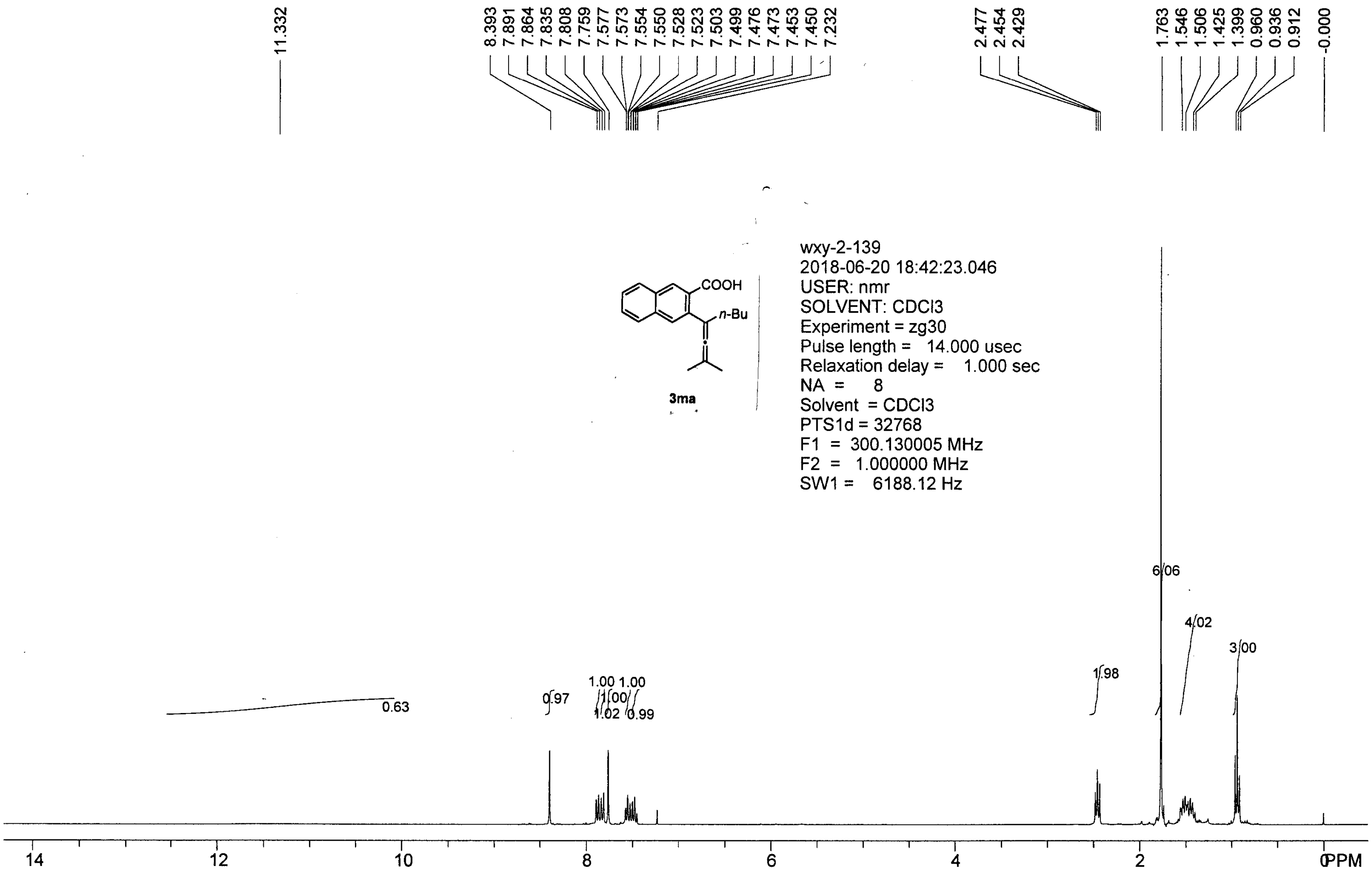
11.332
8.393
7.891
7.864
7.835
7.808
7.759
7.577
7.573
7.554
7.550
7.528
7.523
7.503
7.499
7.476
7.473
7.453
7.450
7.232
2.477
2.454
2.429
1.763
1.546
1.506
1.425
1.399
0.960
0.936
0.912
-0.000
COOH
n-Bu
3ma
wxy-2-139
2018-06-20 18:42:23.046
USER: nmr
SOLVENT: CDCl3
Experiment = zg30
Pulse length = 14.000 usec
Relaxation delay = 1.000 sec
NA = 8
Solvent = CDCl3
PTS1d = 32768
F1 = 300.130005 MHz
F2 = 1.000000 MHz
SW1 = 6188.12 Hz
6.06
4.02
3.00
1.98
0.97
1.00 1.00
1.00
1.02 0.99
0.63
14
12
10
8
6
4
2
0
PPM

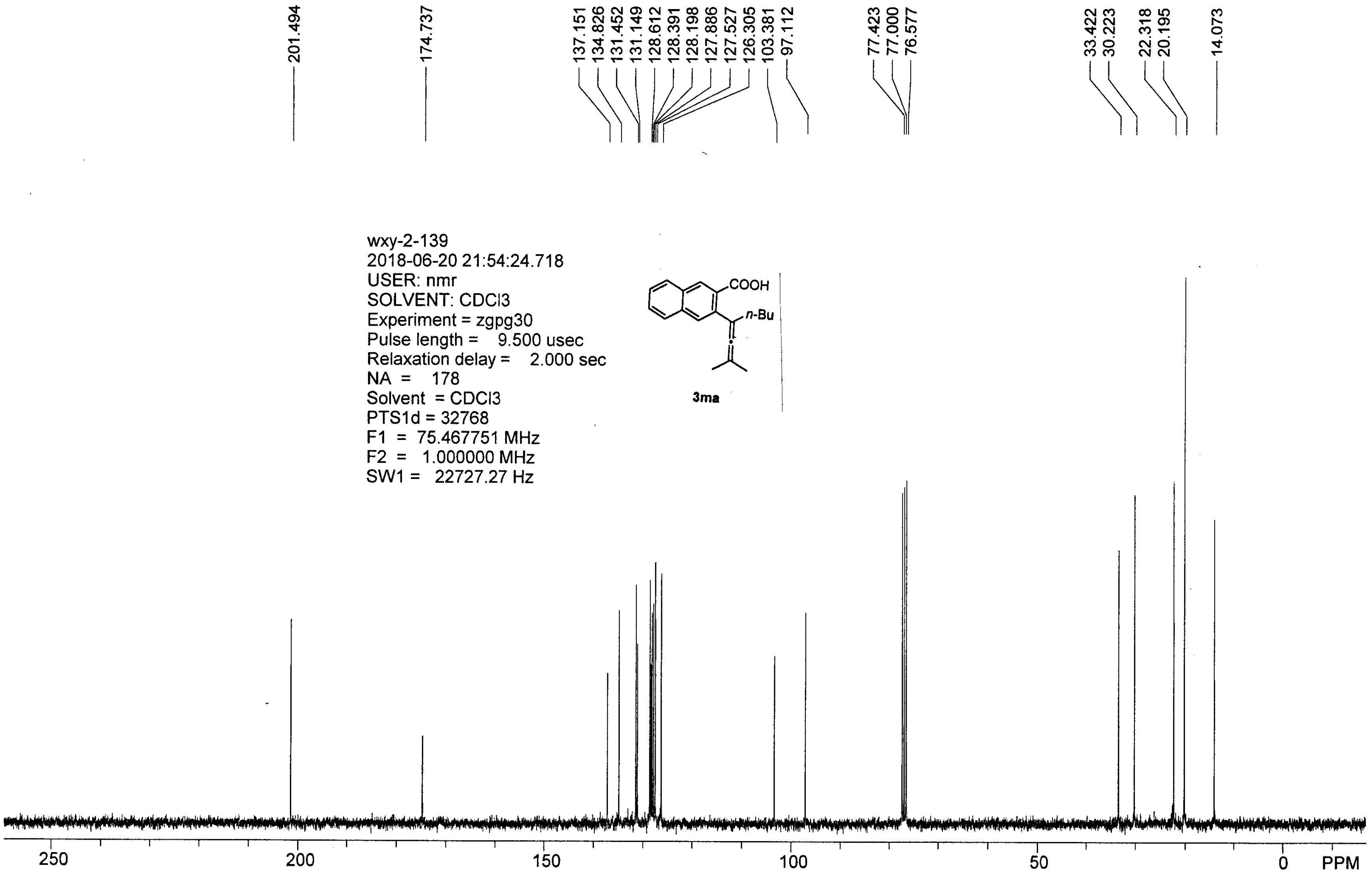
201.494
174.737
137.151
134.826
131.452
131.149
128.612
128.391
128.198
127.886
127.527
126.305
103.381
97.112
77.423
77.000
76.577
33.422
30.223
22.318
20.195
14.073
wxy-2-139
2018-06-20 21:54:24.718
USER: nmr
SOLVENT: CDCl3
Experiment = zgpg30
Pulse length = 9.500 usec
Relaxation delay = 2.000 sec
NA = 178
Solvent = CDCl3
PTS1d = 32768
F1 = 75.467751 MHz
F2 = 1.000000 MHz
SW1 = 22727.27 Hz
COOH
n-Bu
3ma
250
200
150
100
50
0
PPM

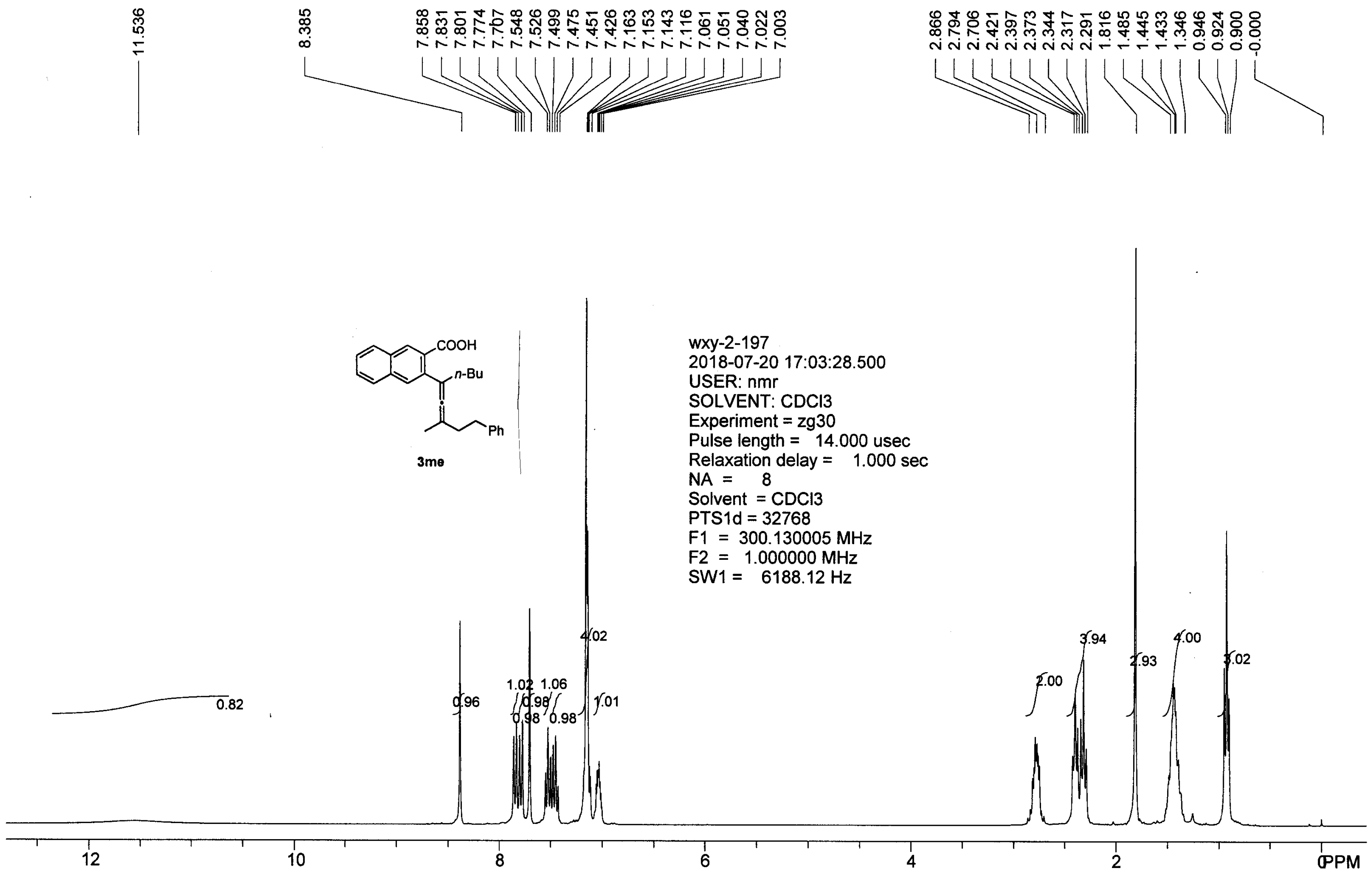
11.536
8.385
7.858
7.831
7.801
7.774
7.707
7.548
7.526
7.499
7.475
7.451
7.426
7.163
7.153
7.143
7.116
7.061
7.051
7.040
7.022
7.003
2.866
2.794
2.706
2.421
2.397
2.373
2.344
2.317
2.291
1.816
1.485
1.445
1.433
1.346
0.946
0.924
0.900
-0.000
COOH
n-Bu
Ph
3me
wxy-2-197
2018-07-20 17:03:28.500
USER: nmr
SOLVENT: CDCl3
Experiment = zg30
Pulse length = 14.000 usec
Relaxation delay = 1.000 sec
NA = 8
Solvent = CDCl3
PTS1d = 32768
F1 = 300.130005 MHz
F2 = 1.000000 MHz
SW1 = 6188.12 Hz
0.82
0.96
1.02
1.06
0.98
0.98
0.98
4.02
1.01
2.00
3.94
2.93
4.00
3.02
12
10
8
6
4
2
0
PPM

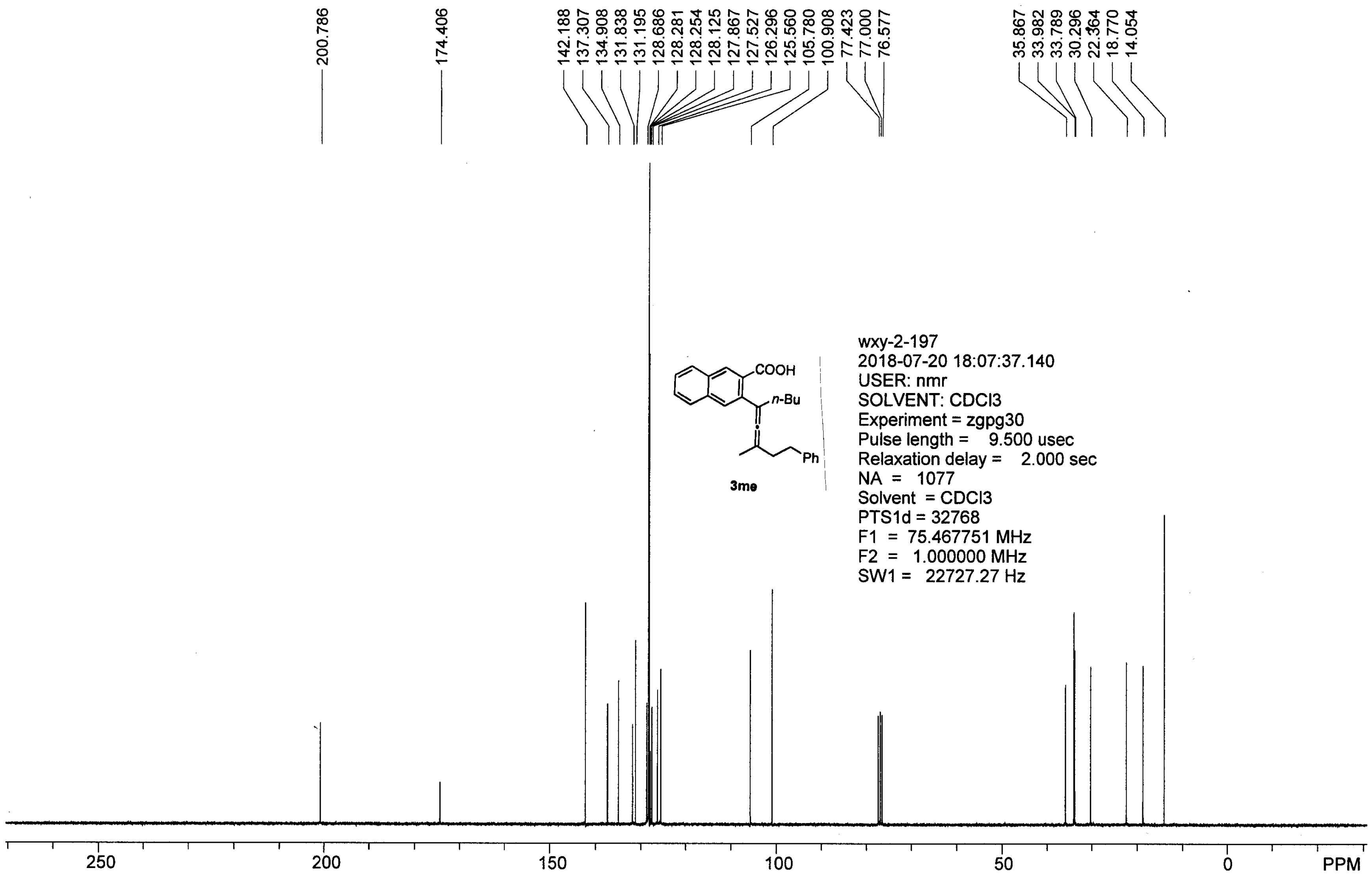
200.786
174.406
142.188
137.307
134.908
131.838
131.195
128.686
128.281
128.254
128.125
127.867
127.527
126.296
125.560
105.780
100.908
77.423
77.000
76.577
35.867
33.982
33.789
30.296
22.364
18.770
14.054
COOH
n-Bu
Ph
3me
wxy-2-197
2018-07-20 18:07:37.140
USER: nmr
SOLVENT: CDCl3
Experiment = zgpg30
Pulse length = 9.500 usec
Relaxation delay = 2.000 sec
NA = 1077
Solvent = CDCl3
PTS1d = 32768
F1 = 75.467751 MHz
F2 = 1.000000 MHz
SW1 = 22727.27 Hz
250
200
150
100
50
0
PPM

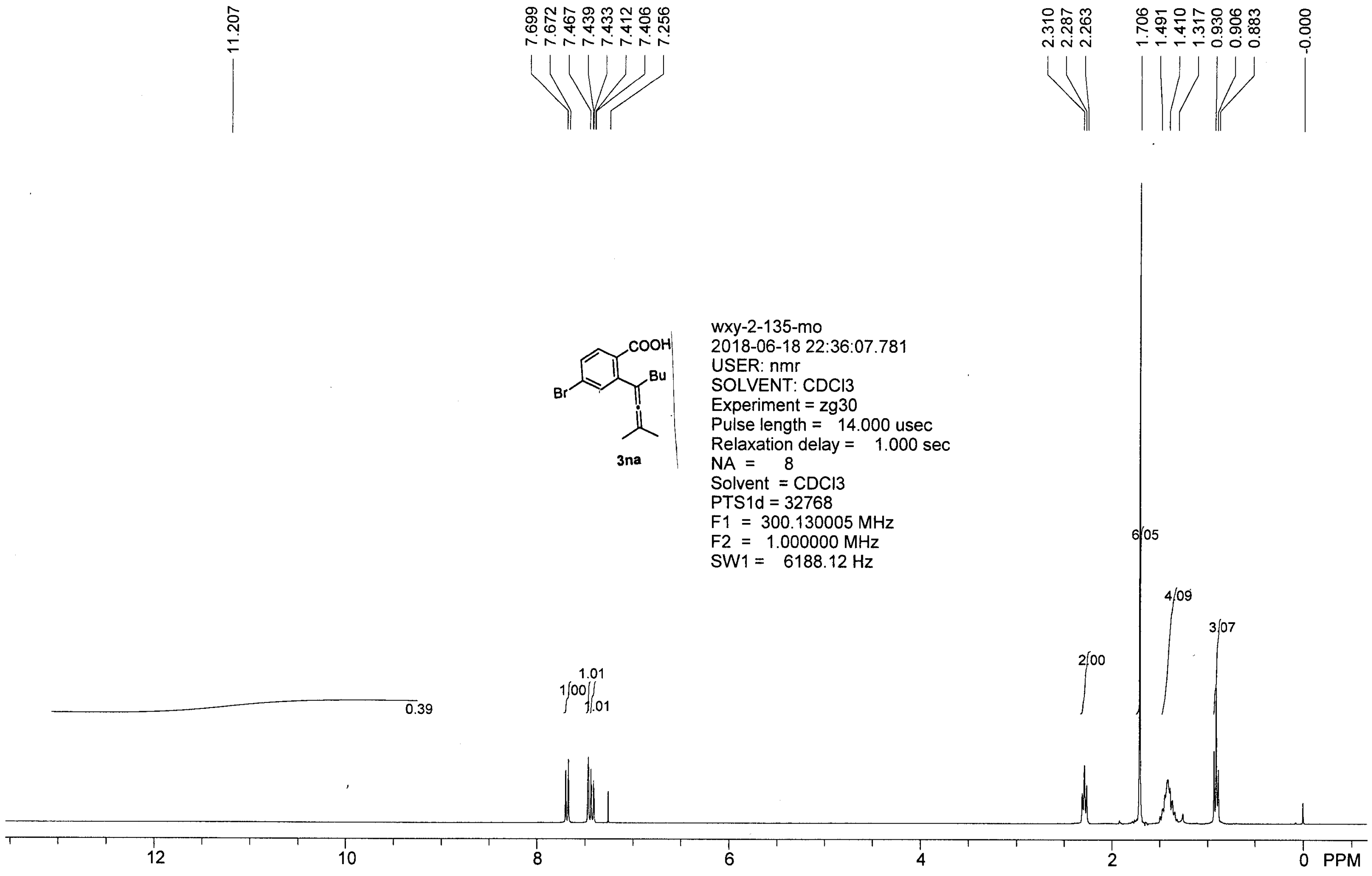

11.207
7.699
7.672
7.467
7.439
7.433
7.412
7.406
7.256
2.310
2.287
2.263
1.706
1.491
1.410
1.317
0.930
0.906
0.883
-0.000
COOH
Bu
Br
3na
wxy-2-135-mo
2018-06-18 22:36:07.781
USER: nmr
SOLVENT: CDCl3
Experiment = zg30
Pulse length = 14.000 usec
Relaxation delay = 1.000 sec
NA = 8
Solvent = CDCl3
PTS1d = 32768
F1 = 300.130005 MHz
F2 = 1.000000 MHz
SW1 = 6188.12 Hz
0.39
1.00
1.01
1.01
2.00
6.05
4.09
3.07
12
10
8
6
4
2
0 PPM

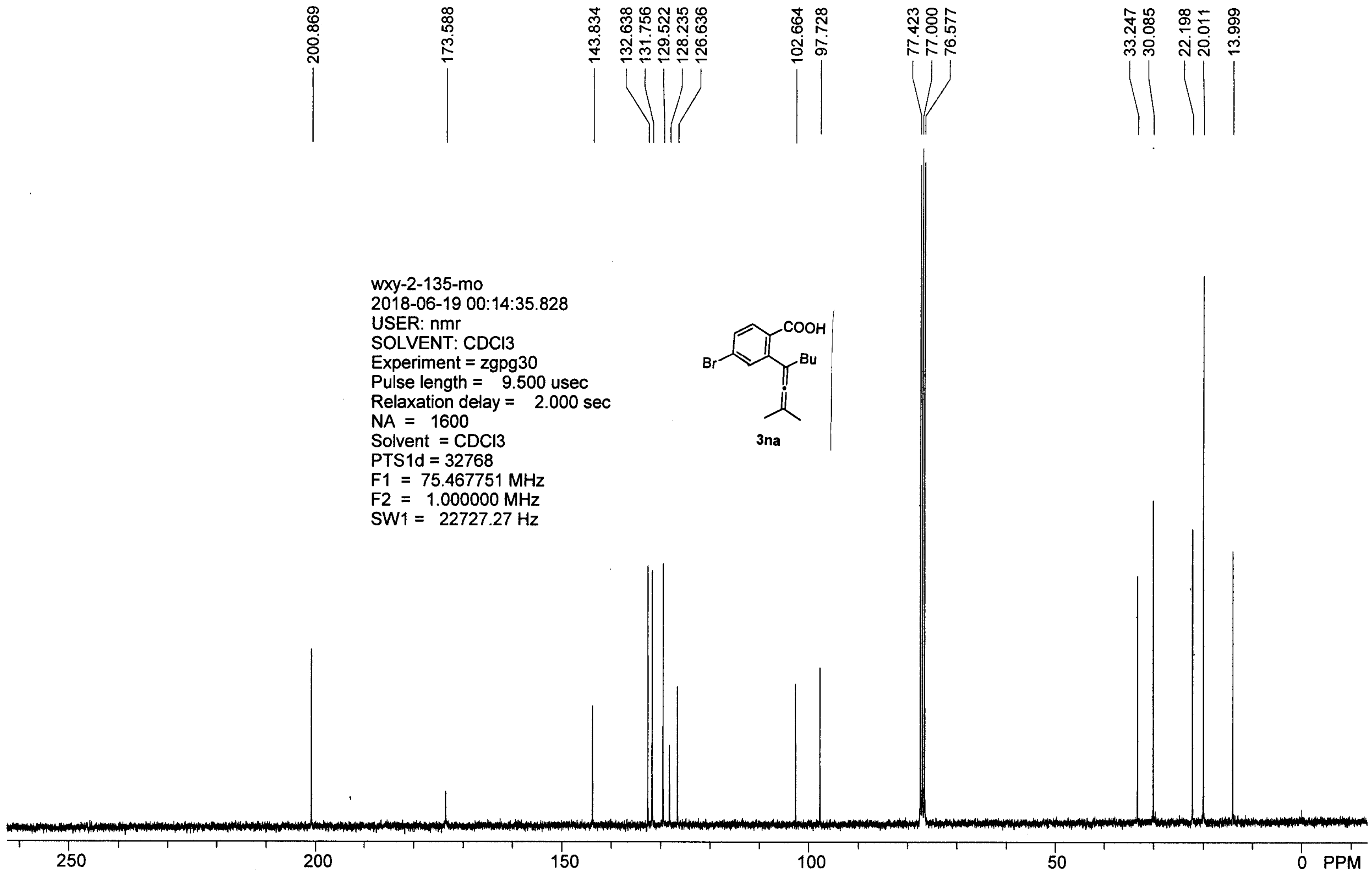
200.869
173.588
143.834
132.638
131.756
129.522
128.235
126.636
102.664
97.728
77.423
77.000
76.577
33.247
30.085
22.198
20.011
13.999
wxy-2-135-mo
2018-06-19 00:14:35.828
USER: nmr
SOLVENT: CDCl3
Experiment = zgpg30
Pulse length = 9.500 usec
Relaxation delay = 2.000 sec
NA = 1600
Solvent = CDCl3
PTS1d = 32768
F1 = 75.467751 MHz
F2 = 1.000000 MHz
SW1 = 22727.27 Hz
COOH
Br
Bu
3na
250
200
150
100
50
0 PPM

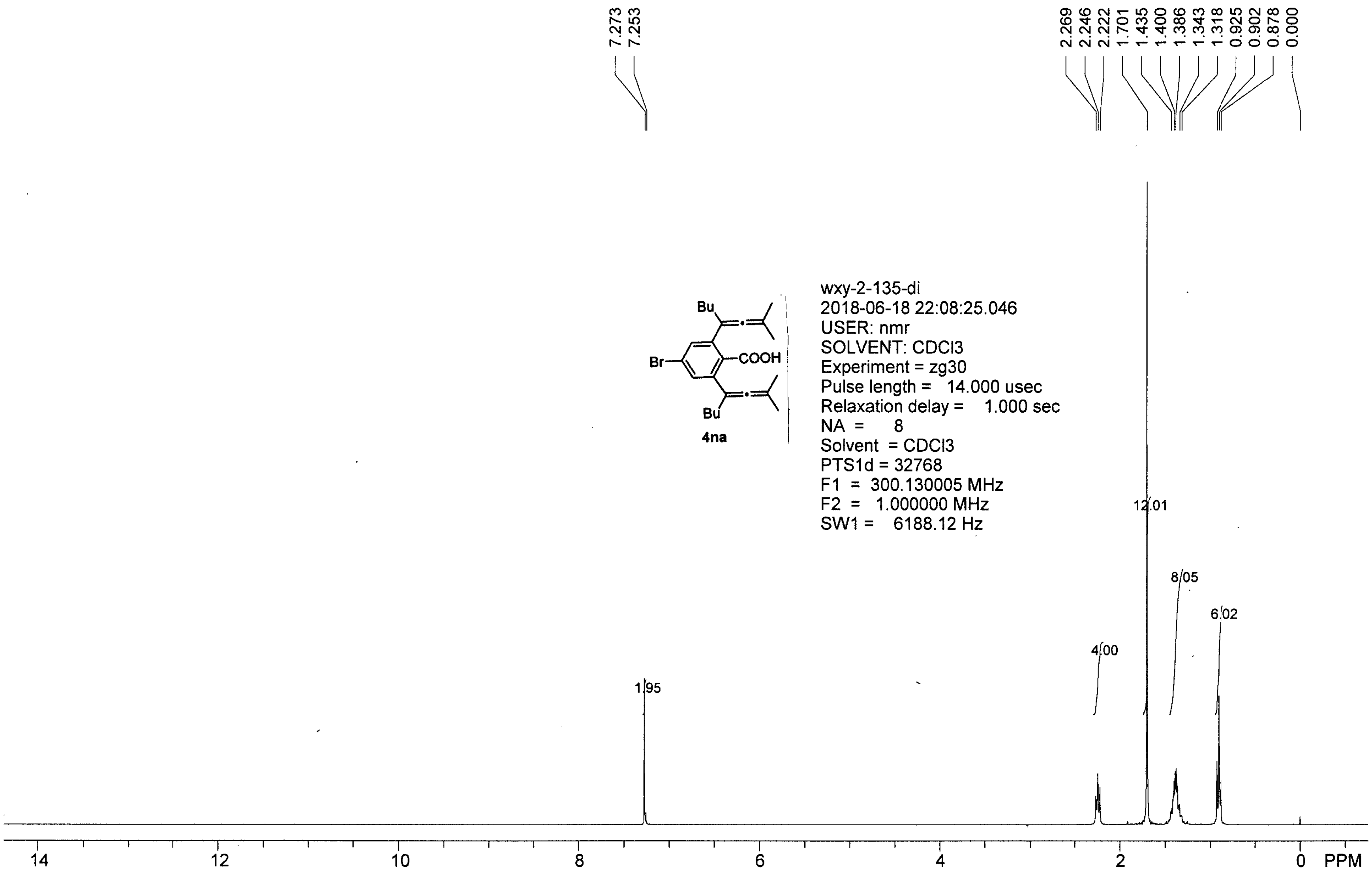
7.273
7.253
2.269
2.246
2.222
1.701
1.435
1.400
1.386
1.343
1.318
0.925
0.902
0.878
0.000
Bu
Br
COOH
Bu
4na
wxy-2-135-di
2018-06-18 22:08:25.046
USER: nmr
SOLVENT: CDCl3
Experiment = zg30
Pulse length = 14.000 usec
Relaxation delay = 1.000 sec
NA = 8
Solvent = CDCl3
PTS1d = 32768
F1 = 300.130005 MHz
F2 = 1.000000 MHz
SW1 = 6188.12 Hz
12.01
8.05
6.02
4.00
1.95
14
12
10
8
6
4
2
0
PPM

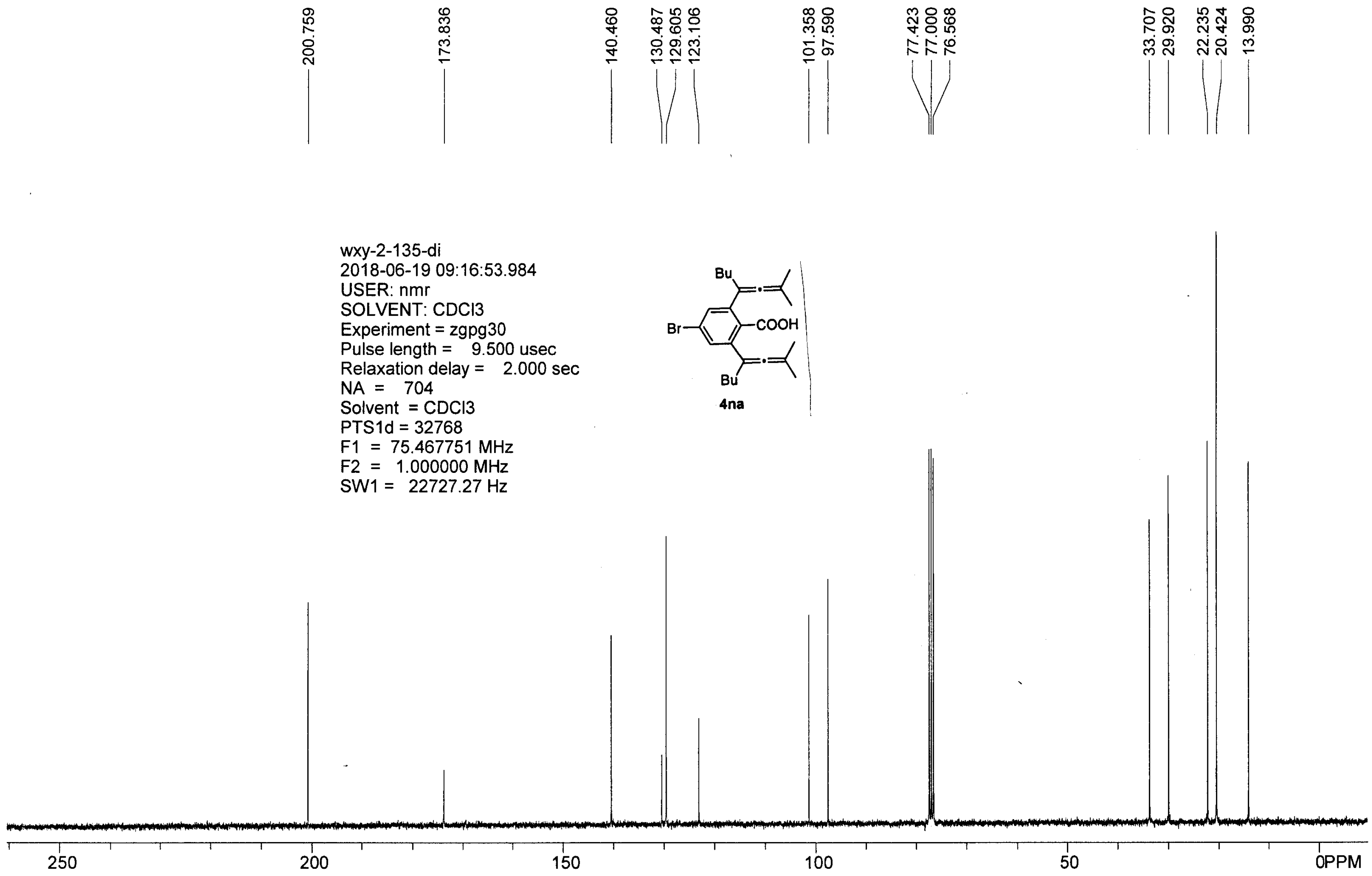
200.759
173.836
140.460
130.487
129.605
123.106
101.358
97.590
77.423
77.000
76.568
33.707
29.920
22.235
20.424
13.990
wxy-2-135-di
2018-06-19 09:16:53.984
USER: nmr
SOLVENT: CDCl3
Experiment = zgpg30
Pulse length = 9.500 usec
Relaxation delay = 2.000 sec
NA = 704
Solvent = CDCl3
PTS1d = 32768
F1 = 75.467751 MHz
F2 = 1.000000 MHz
SW1 = 22727.27 Hz
250
200
150
100
50
0PPM
Bu
Br
COOH
Bu

4na

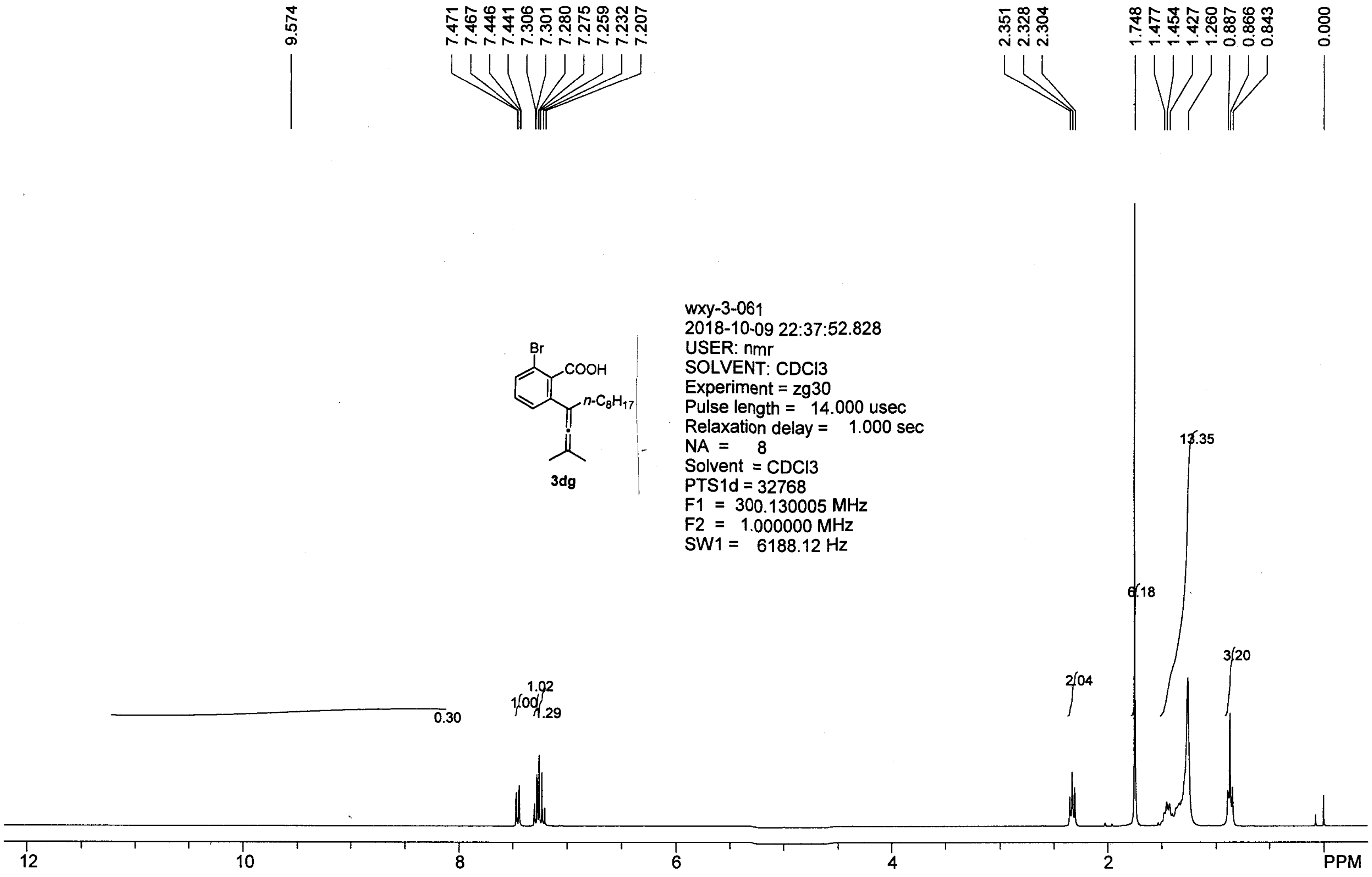
9.574
7.471
7.467
7.446
7.441
7.306
7.301
7.280
7.275
7.259
7.232
7.207
2.351
2.328
2.304
1.748
1.477
1.454
1.427
1.260
0.887
0.866
0.843
0.000
Br
COOH
n-C8H17
3dg
wxy-3-061
2018-10-09 22:37:52.828
USER: nmr
SOLVENT: CDCl3
Experiment = zg30
Pulse length = 14.000 usec
Relaxation delay = 1.000 sec
NA = 8
Solvent = CDCl3
PTS1d = 32768
F1 = 300.130005 MHz
F2 = 1.000000 MHz
SW1 = 6188.12 Hz
13.35
6.18
3.20
2.04
1.02
1.00
1.29
0.30
12
10
8
6
4
2
PPM

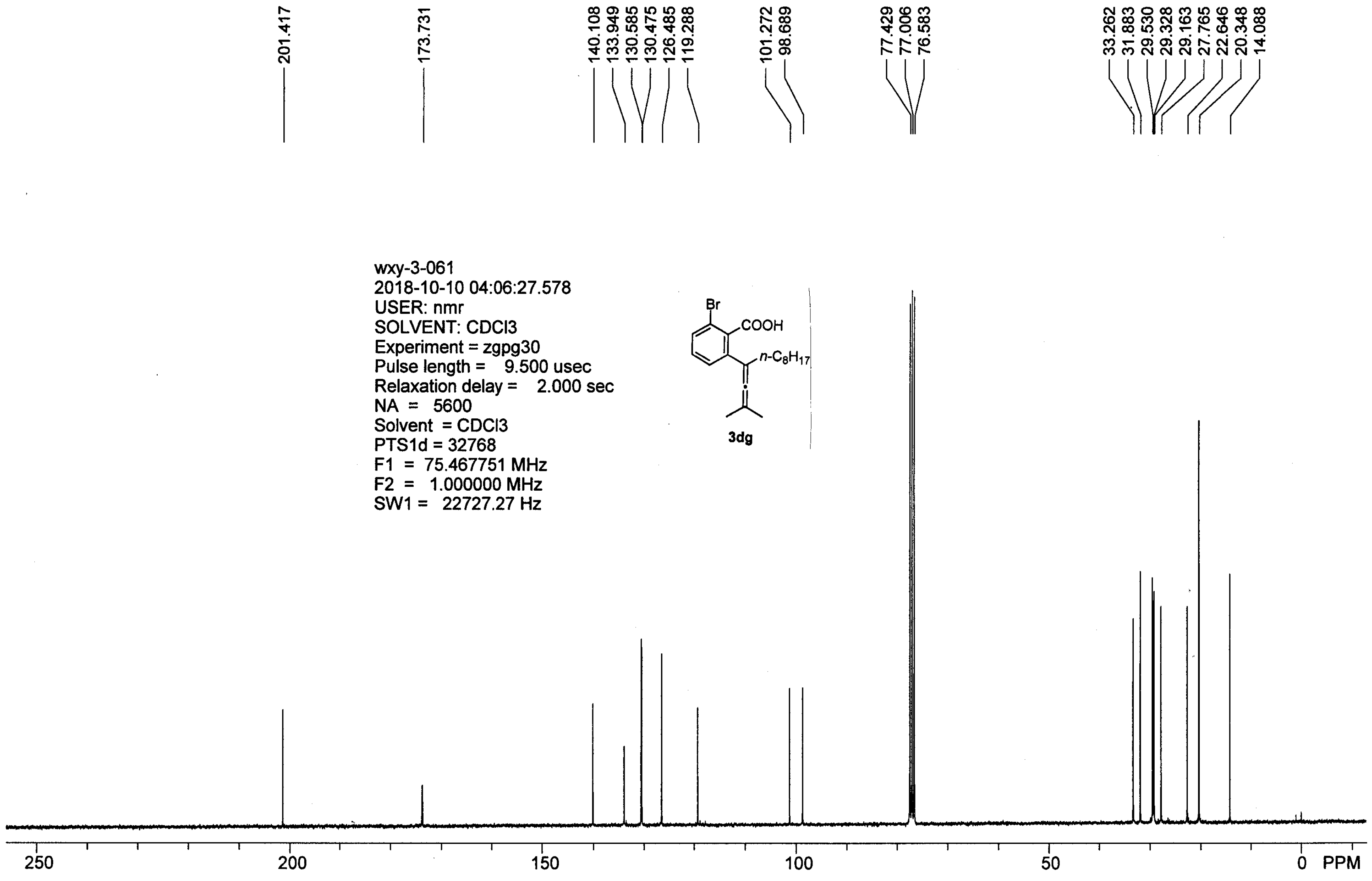
201.417
173.731
140.108
133.949
130.585
130.475
126.485
119.288
101.272
98.689
77.429
77.006
76.583
33.262
31.883
29.530
29.328
29.163
27.765
22.646
20.348
14.088
wxy-3-061
2018-10-10 04:06:27.578
USER: nmr
SOLVENT: CDCl3
Experiment = zgpg30
Pulse length = 9.500 usec
Relaxation delay = 2.000 sec
NA = 5600
Solvent = CDCl3
PTS1d = 32768
F1 = 75.467751 MHz
F2 = 1.000000 MHz
SW1 = 22727.27 Hz
Br
COOH
n-C8H17
3dg
250
200
150
100
50
0 PPM

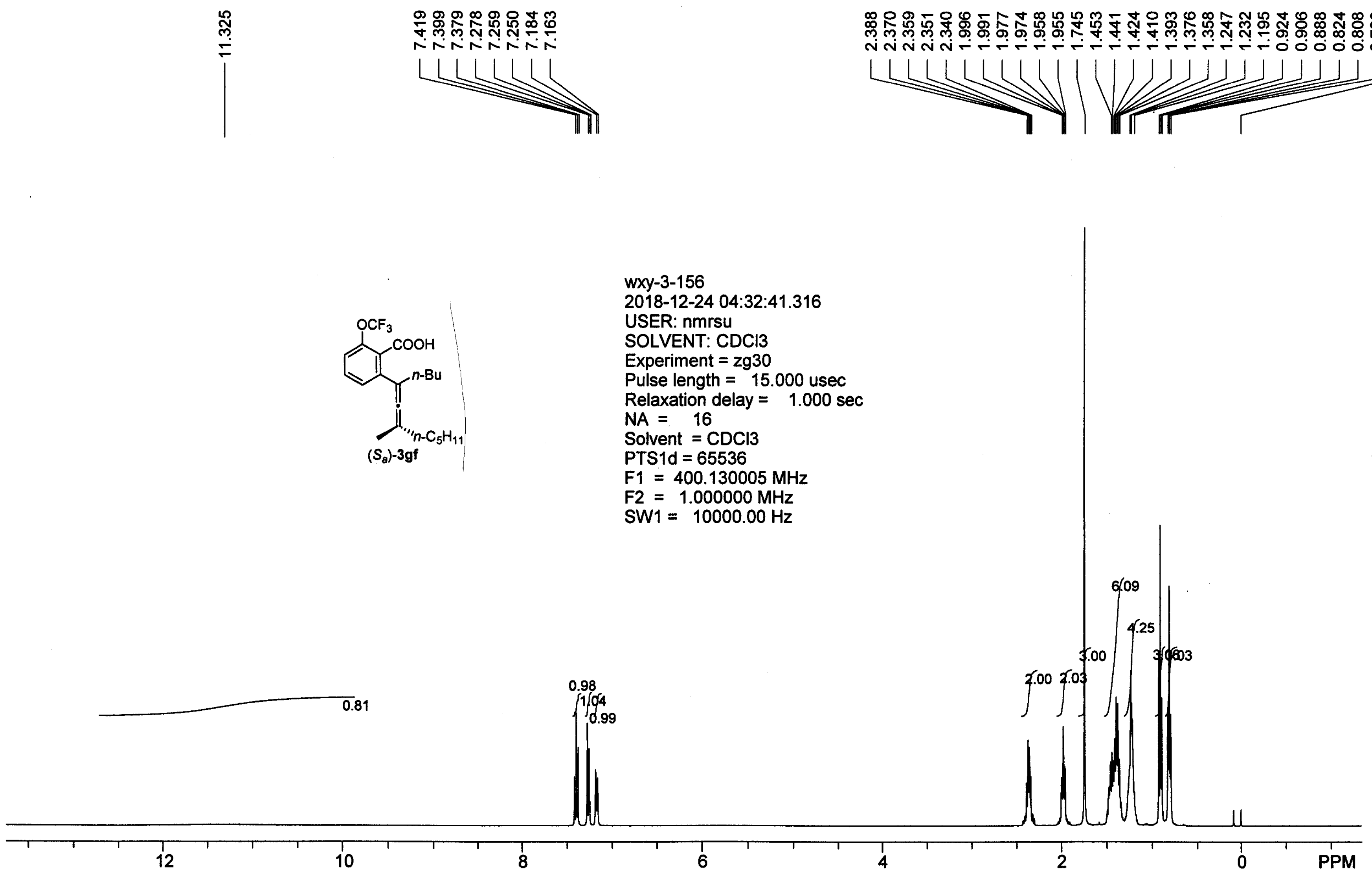
11.325
7.419 7.399 7.379 7.278 7.259 7.250 7.184 7.163
2.388 2.370 2.359 2.351 2.340 1.996 1.991 1.977 1.974 1.958 1.955 1.745 1.453 1.441 1.424 1.410 1.393 1.376 1.358 1.247 1.232 1.195 0.924 0.906 0.888 0.824 0.808 0.790 0.000
OCF3
COOH
n-Bu
n-C5H11
(Sa)-3gf
wxy-3-156
2018-12-24 04:32:41.316
USER: nmrsu
SOLVENT: CDCl3
Experiment = zg30
Pulse length = 15.000 usec
Relaxation delay = 1.000 sec
NA = 16
Solvent = CDCl3
PTS1d = 65536
F1 = 400.130005 MHz
F2 = 1.000000 MHz
SW1 = 10000.00 Hz
0.81
0.98
1.04
0.99
2.00
2.03
3.00
6.09
4.25
12
10
8
6
4
2
0
PPM

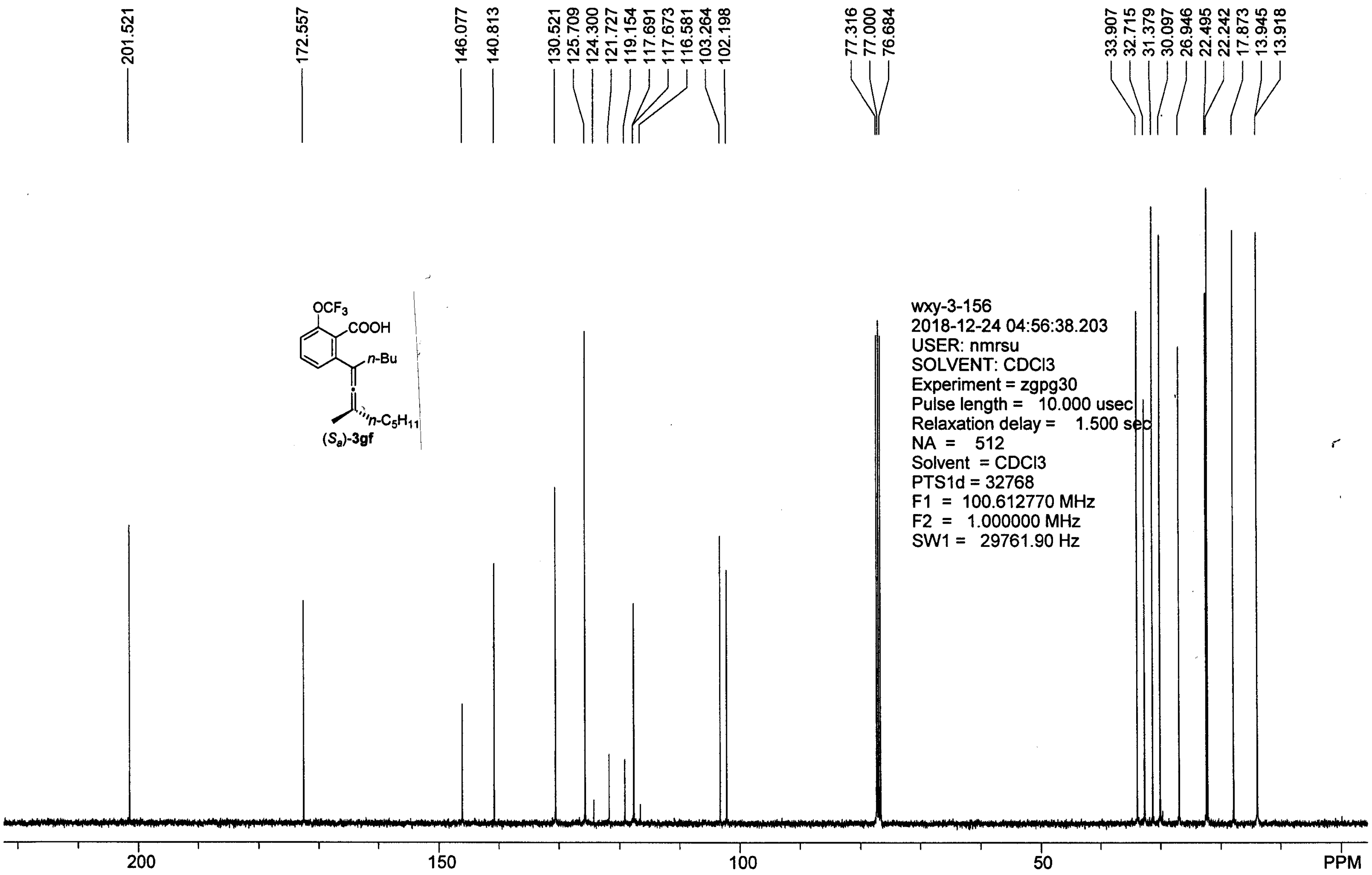
201.521
172.557
146.077
140.813
130.521
125.709
124.300
121.727
119.154
117.691
117.673
116.581
103.264
102.198
77.316
77.000
76.684
33.907
32.715
31.379
30.097
26.946
22.495
22.242
17.873
13.945
13.918
OCF3
COOH
n-Bu
n-C5H11
(Sa)-3gf
wxy-3-156
2018-12-24 04:56:38.203
USER: nmrsu
SOLVENT: CDCl3
Experiment = zgpg30
Pulse length = 10.000 usec
Relaxation delay = 1.500 sec
NA = 512
Solvent = CDCl3
PTS1d = 32768
F1 = 100.612770 MHz
F2 = 1.000000 MHz
SW1 = 29761.90 Hz
200
150
100
50
PPM

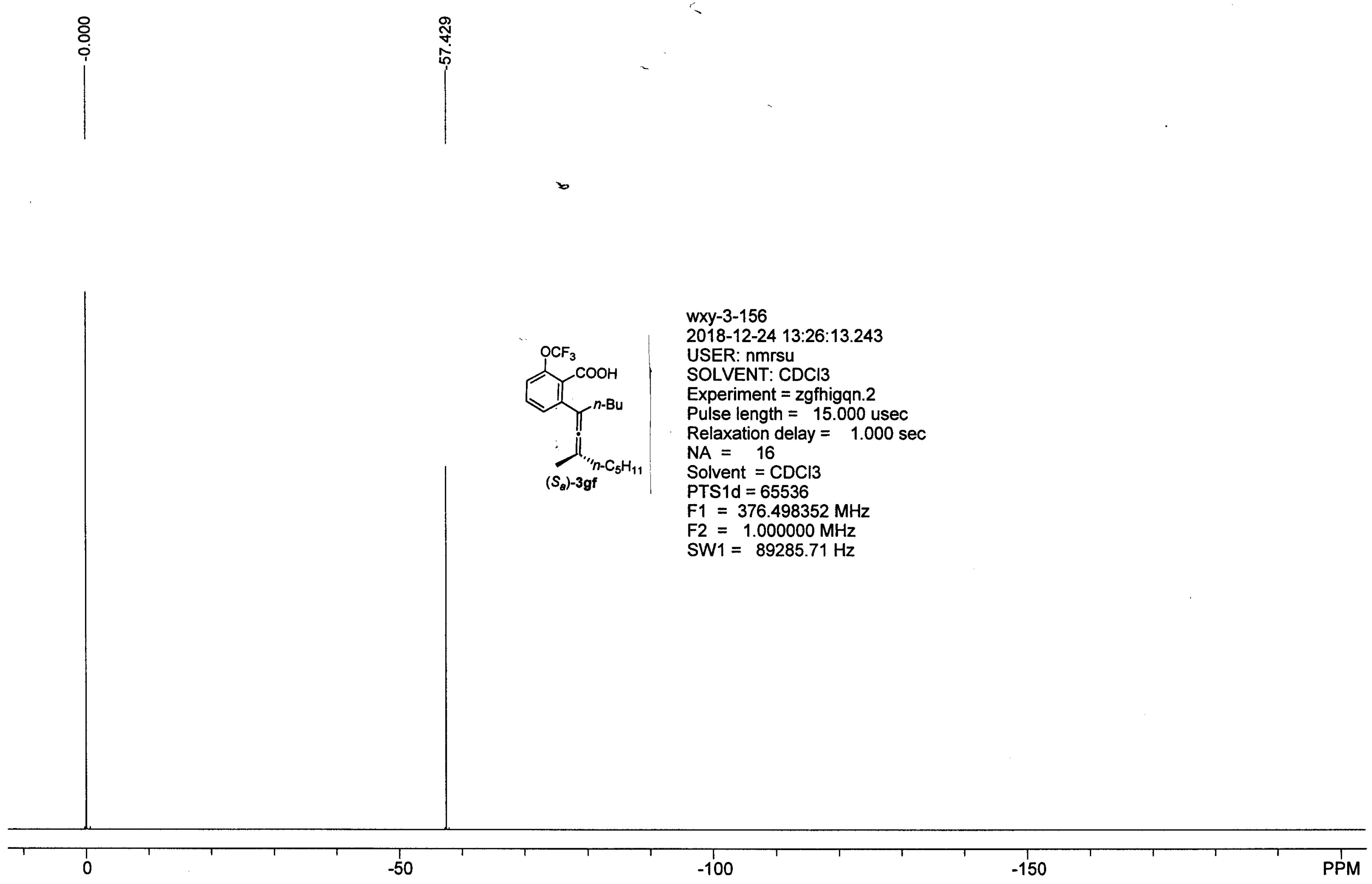
-0.000
-57.429
OCF3
COOH
n-Bu
n-C5H11
(Sa)-3gf
wxy-3-156
2018-12-24 13:26:13.243
USER: nmrsu
SOLVENT: CDCl3
Experiment = zgfhigqn.2
Pulse length = 15.000 usec
Relaxation delay = 1.000 sec
NA = 16
Solvent = CDCl3
PTS1d = 65536
F1 = 376.498352 MHz
F2 = 1.000000 MHz
SW1 = 89285.71 Hz
0
-50
-100
-150
PPM

Empower 3

Default Individual Report

SAMPLE INFORMATION

Sample Name:	wxy-3-048-rac-oj-3-98-2	Acquired By:	System
Sample Type:	Unknown	Sample Set Name	
Vial:	1:D,2	Acq. Method Set:	test1
Injection #:	2	Processing Method	TEST
Injection Volume:	0.50 ul	Channel Name:	PDA Ch2 254nm@4.8nm
Run Time:	50.0 Minutes	Proc. Chnl. Descr.:	PDA Ch2 254nm@4.8nm
Date Acquired:	1/21/2019 10:11:06 AM CST		
Date Processed:	1/21/2019 4:20:48 PM CST		

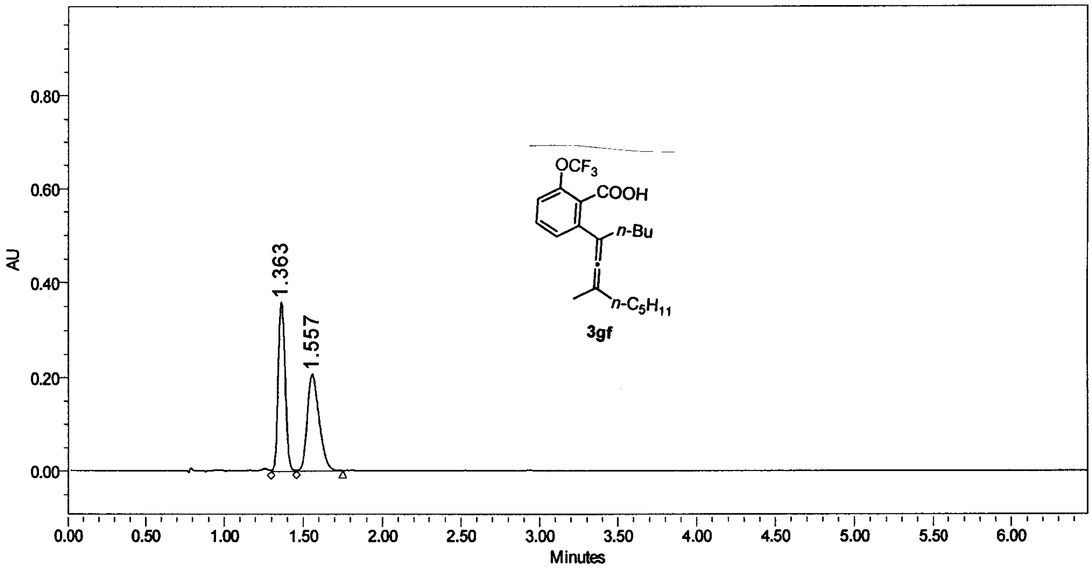

	RT	Peak Type	Height	Width (sec)	Area	% Area
1	1.363	Unknown	359850	9.600	1093604	50.27
2	1.557	Unknown	207147	17.500	1081703	49.73

Reported by User: System
Report Method: Default Individual Report
Report Method ID 9006
Page: 1 of 1

Project Name: TEST
Date Printed:
1/21/2019
4:22:33 PM PRC

Empower 3

Default Individual Report

SAMPLE INFORMATION

Sample Name:	wxy-3-156	Acquired By:	System
Sample Type:	Unknown	Sample Set Name	
Vial:	1:D,5	Acq. Method Set:	test1
Injection #:	1	Processing Method	TEST
Injection Volume:	1.00 ul	Channel Name:	PDA Ch2 254nm@4.8nm
Run Time:	50.0 Minutes	Proc. Chnl. Descr.:	PDA Ch2 254nm@4.8nm
Date Acquired:	1/21/2019 10:41:55 AM CST		
Date Processed:	1/21/2019 4:22:18 PM CST		

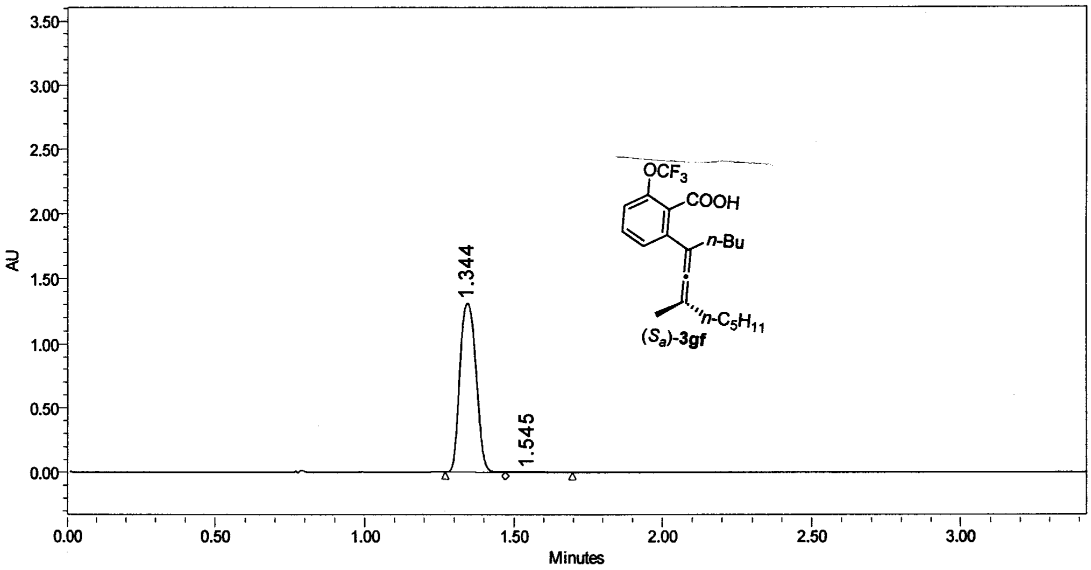

	RT	Peak Type	Height	Width (sec)	Area	% Area
1	1.344	Unknown	1308604	12.100	4947258	99.74
2	1.545	Unknown	2011	13.550	13023	0.26

Reported by User: System
Report Method: Default Individual Report
Report Method ID 9006
Page: 1 of 1

Project Name: TEST
Date Printed:
1/21/2019
4:23:11 PM PRC

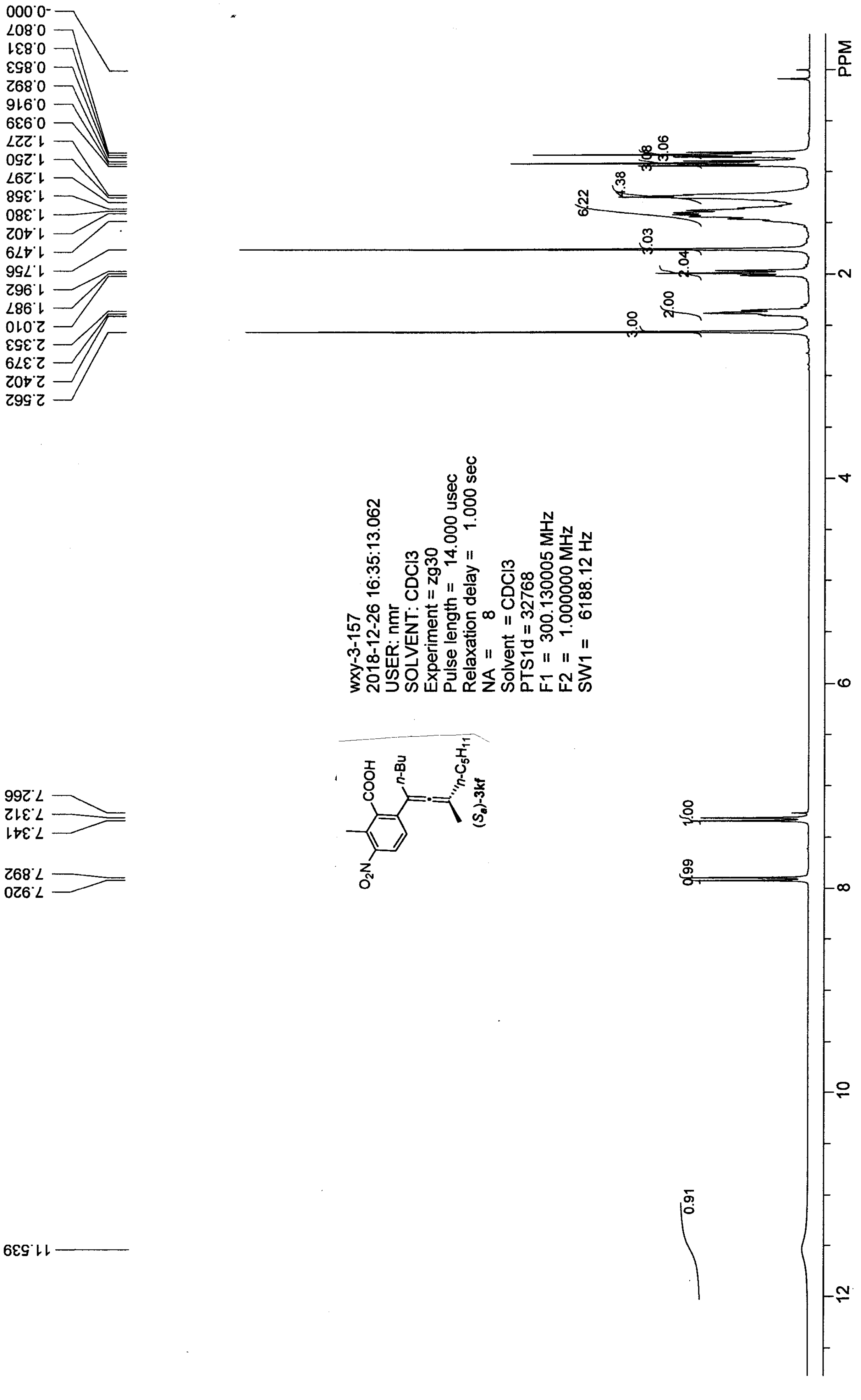
wxy-3-157
2018-12-26 16:35:13.062
USER: nmr
SOLVENT: CDCl3
Experiment = zg30
Pulse length = 14.000 usec
Relaxation delay = 1.000 sec
NA = 8
Solvent = CDCl3
PTS1d = 32768
F1 = 300.130005 MHz
F2 = 1.000000 MHz
SW1 = 6188.12 Hz
COOH
n-Bu
n-C5H11
(Sa)-3kf
O2N
-0.000
0.807
0.831
0.853
0.892
0.916
0.939
1.227
1.250
1.297
1.358
1.380
1.402
1.479
1.756
1.962
1.987
2.010
2.353
2.379
2.402
2.562
7.266
7.312
7.341
7.892
7.920
11.539
PPM

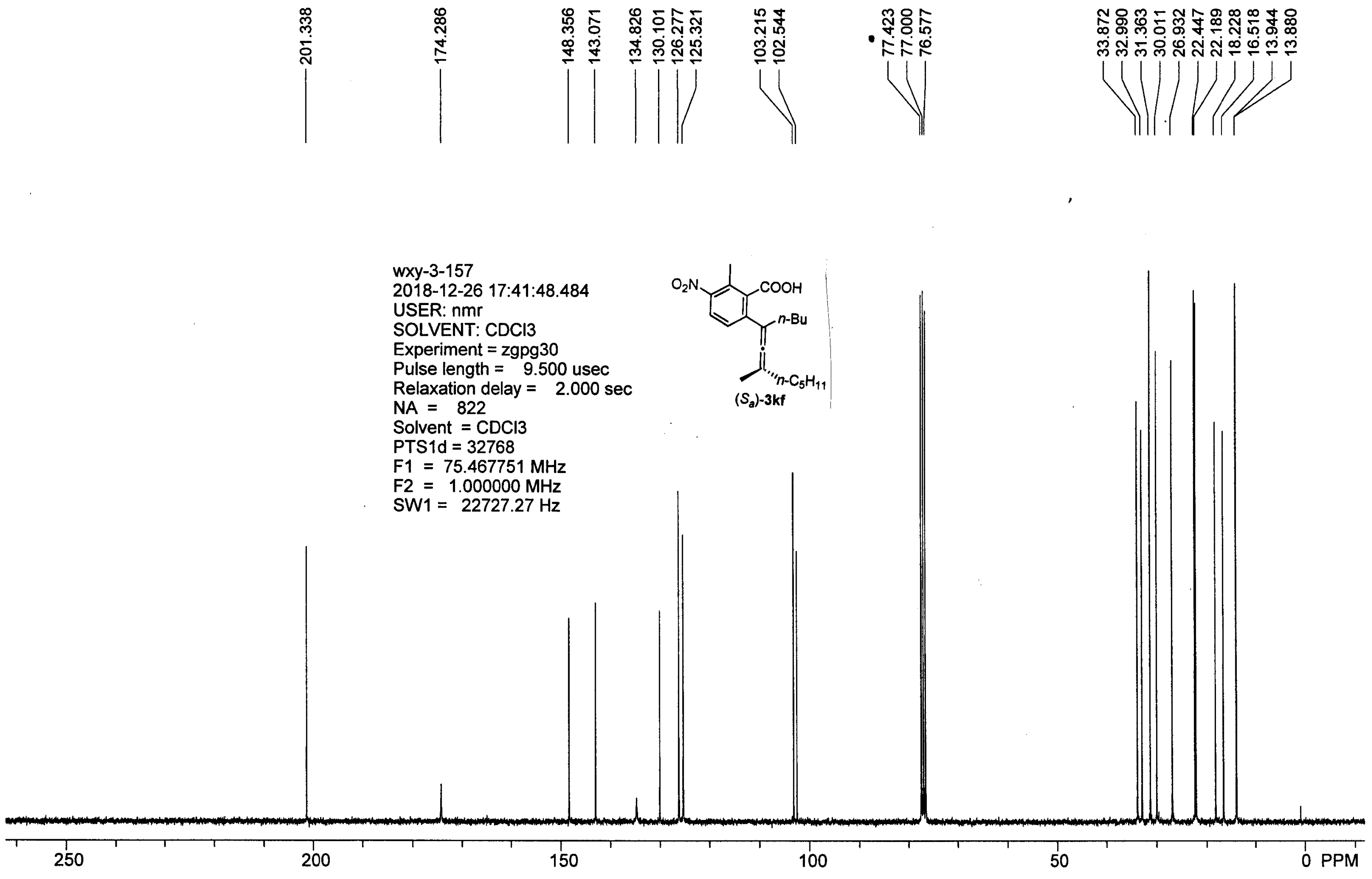
201.338
174.286
148.356
143.071
134.826
130.101
126.277
125.321
103.215
102.544
77.423
77.000
76.577
33.872
32.990
31.363
30.011
26.932
22.447
22.189
18.228
16.518
13.944
13.880
wxy-3-157
2018-12-26 17:41:48.484
USER: nmr
SOLVENT: CDCl3
Experiment = zgpg30
Pulse length = 9.500 usec
Relaxation delay = 2.000 sec
NA = 822
Solvent = CDCl3
PTS1d = 32768
F1 = 75.467751 MHz
F2 = 1.000000 MHz
SW1 = 22727.27 Hz
O2N
COOH
n-Bu
n-C5H11
(Sa)-3kf
250
200
150
100
50
0 PPM

wxy-3-152-oz-h-100-1-1-214

实验时间：2018-12-29, 11:10:52　　　　报告时间：2018-12-29, 18:52:14
谱图文件:D:\zhuguangjiong\wxy\20181228\wxy-3-152-oz-h-100-1-1-214.org

实验内容简介：

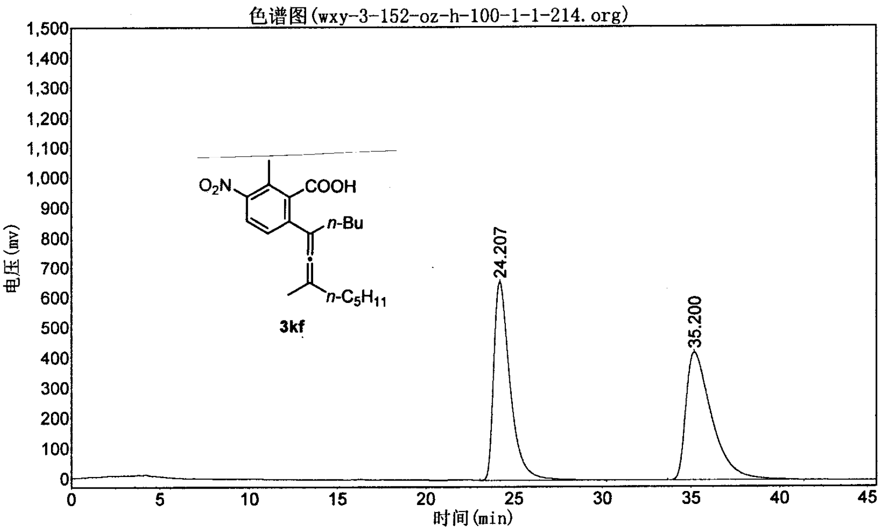

分析结果表

峰号	峰名	保留时间	峰高	峰面积	含量
1		24.207	657798.938	42242948.000	49.6882
2		35.200	422149.750	42773028.000	50.3118
总计			1079948.688	85015976.000	100.0000

wxy-3-157-oz-h-100-1-1-214

实验时间：2018-12-29，11:57:06　　　　报告时间：2018-12-29，18:53:21
谱图文件:D:\zhuguangjiong\wxy\20181228\wxy-3-157-oz-h-100-1-1-214.org

实验内容简介：

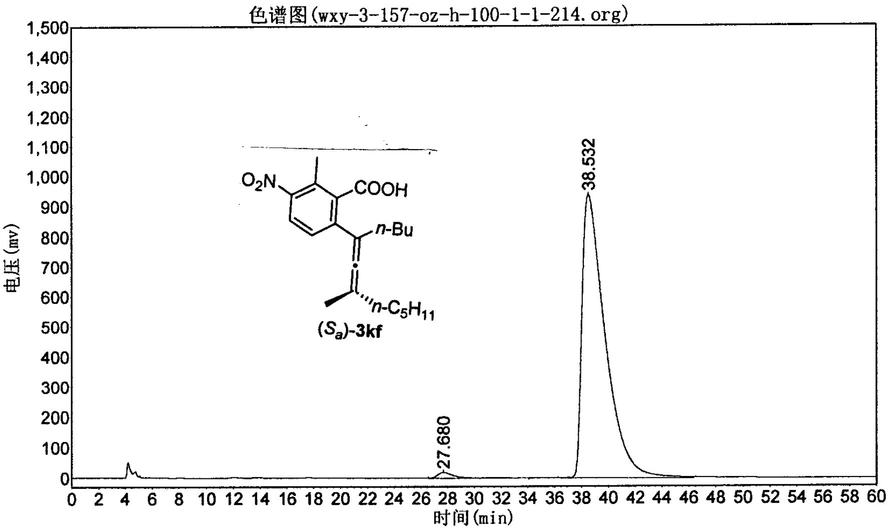

分析结果表

峰号	峰名	保留时间	峰高	峰面积	含量
1		27.680	19245.367	1573623.125	1.3856
2		38.532	940428.125	111992480.000	98.6144
总计			959673.492	113566103.125	100.0000

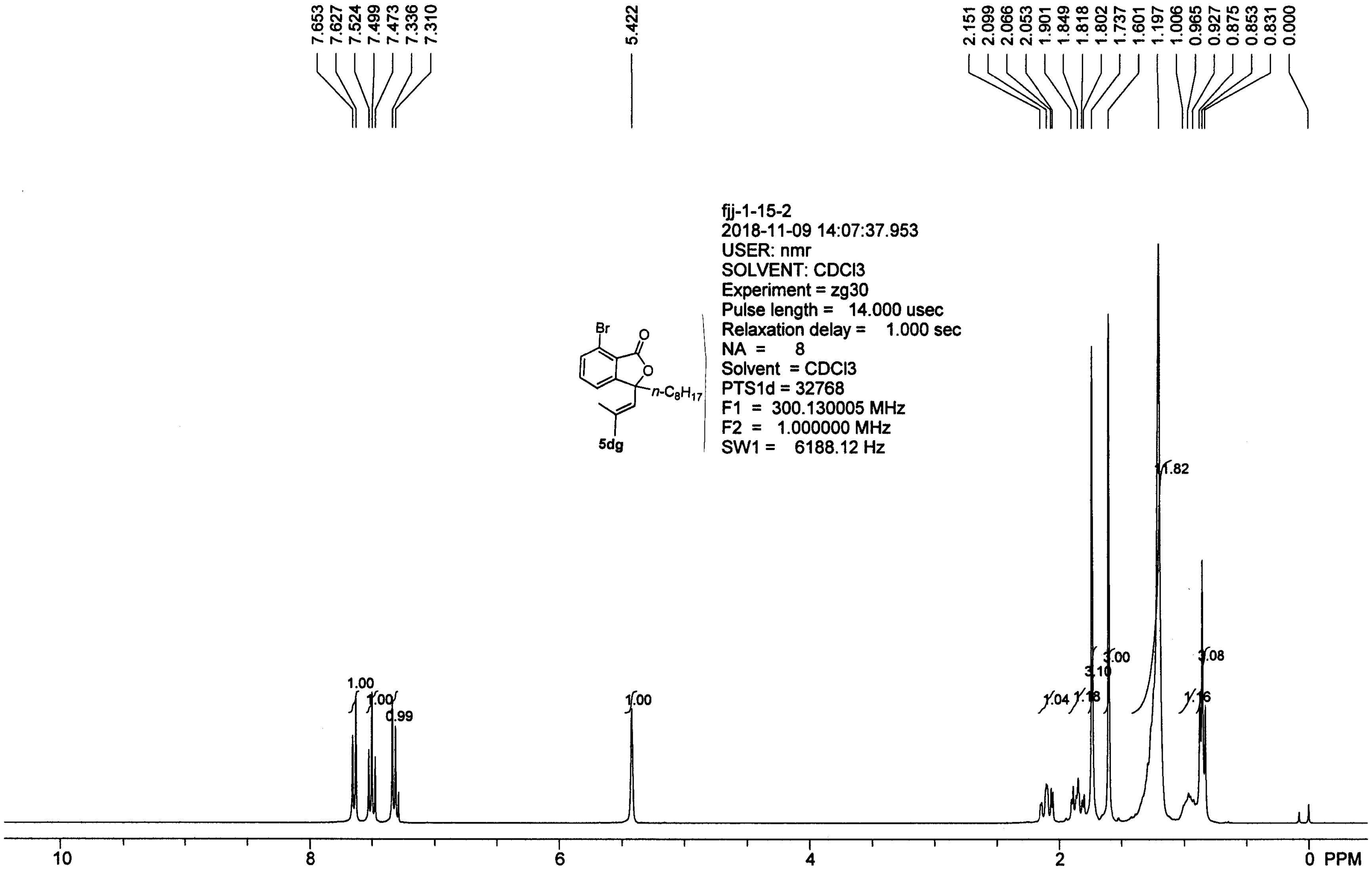
7.653
7.627
7.524
7.499
7.473
7.336
7.310
5.422
2.151
2.099
2.066
2.053
1.901
1.849
1.818
1.802
1.737
1.601
1.197
1.006
0.965
0.927
0.875
0.853
0.831
0.000
fjj-1-15-2
2018-11-09 14:07:37.953
USER: nmr
SOLVENT: CDCl3
Experiment = zg30
Pulse length = 14.000 usec
Relaxation delay = 1.000 sec
NA = 8
Solvent = CDCl3
PTS1d = 32768
F1 = 300.130005 MHz
F2 = 1.000000 MHz
SW1 = 6188.12 Hz
Br
O
O
n-C8H17
5dg
1.00
1.00
0.99
1.00
1.04
1.18
3.10
3.00
11.82
3.08
1.16
10
8
6
4
2
0 PPM

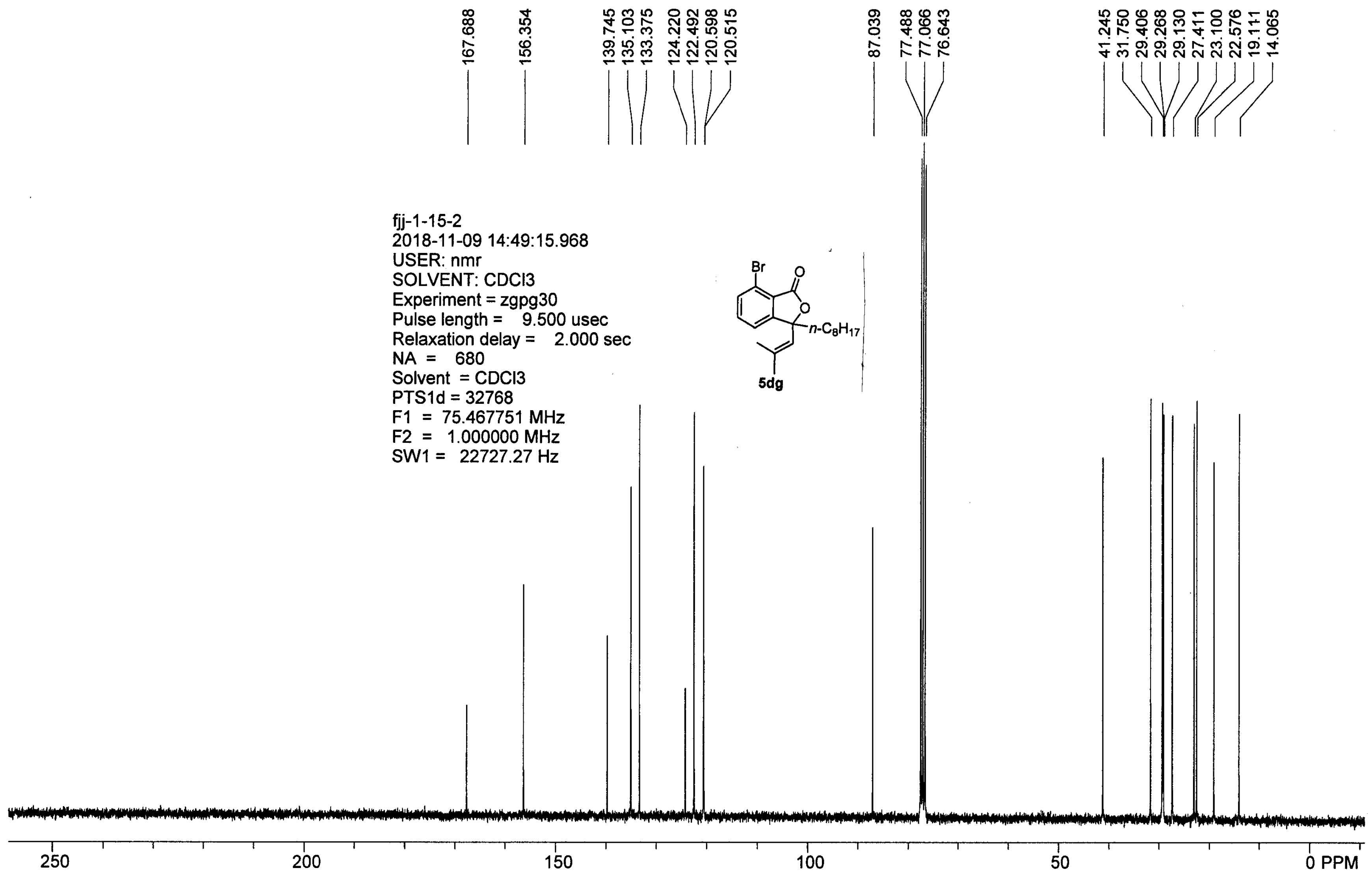
167.688
156.354
139.745
135.103
133.375
124.220
122.492
120.598
120.515
87.039
77.488
77.066
76.643
41.245
31.750
29.406
29.268
29.130
27.411
23.100
22.576
19.111
14.065
fjj-1-15-2
2018-11-09 14:49:15.968
USER: nmr
SOLVENT: CDCl3
Experiment = zgpg30
Pulse length = 9.500 usec
Relaxation delay = 2.000 sec
NA = 680
Solvent = CDCl3
PTS1d = 32768
F1 = 75.467751 MHz
F2 = 1.000000 MHz
SW1 = 22727.27 Hz
Br
O
O
n-C8H17
5dg
250
200
150
100
50
0 PPM

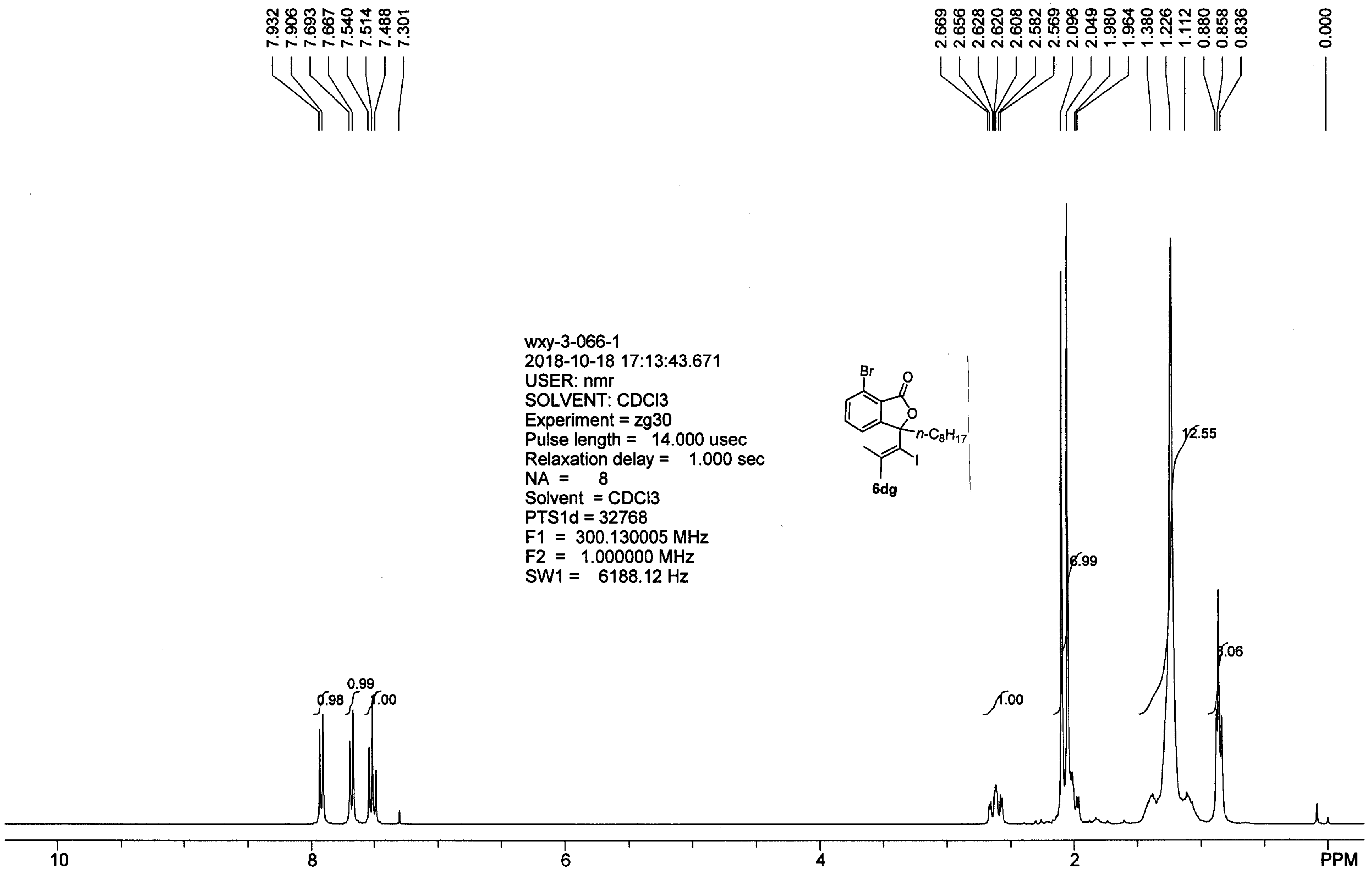
7.932
7.906
7.693
7.667
7.540
7.514
7.488
7.301
2.669
2.656
2.628
2.620
2.608
2.582
2.569
2.096
2.049
1.980
1.964
1.380
1.226
1.112
0.880
0.858
0.836
0.000
wxy-3-066-1
2018-10-18 17:13:43.671
USER: nmr
SOLVENT: CDCl3
Experiment = zg30
Pulse length = 14.000 usec
Relaxation delay = 1.000 sec
NA = 8
Solvent = CDCl3
PTS1d = 32768
F1 = 300.130005 MHz
F2 = 1.000000 MHz
SW1 = 6188.12 Hz
Br
O
O
n-C8H17
I
6dg
12.55
6.99
3.06
1.00
0.98
0.99
1.00
10
8
6
4
2
PPM

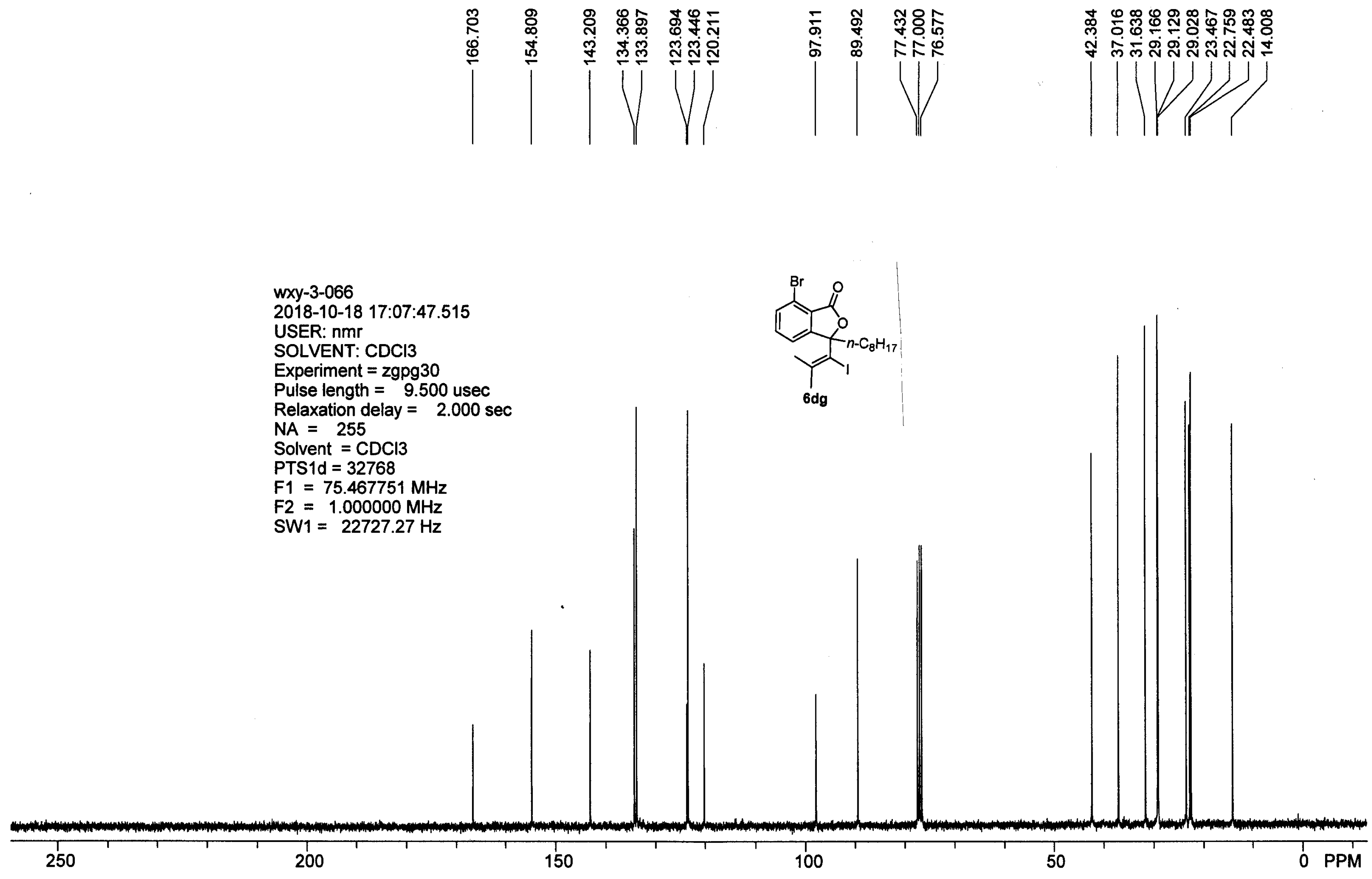
166.703
154.809
143.209
134.366
133.897
123.694
123.446
120.211
97.911
89.492
77.432
77.000
76.577
42.384
37.016
31.638
29.166
29.129
29.028
23.467
22.759
22.483
14.008
wxy-3-066
2018-10-18 17:07:47.515
USER: nmr
SOLVENT: CDCl3
Experiment = zgpg30
Pulse length = 9.500 usec
Relaxation delay = 2.000 sec
NA = 255
Solvent = CDCl3
PTS1d = 32768
F1 = 75.467751 MHz
F2 = 1.000000 MHz
SW1 = 22727.27 Hz
Br
O
O
n-C8H17
I
6dg
250
200
150
100
50
0 PPM

7.921
7.896
7.684
7.659
7.521
7.495
7.468
6.803
-0.000

Purity (98%) is determined by mesitylene (11.0 μL, 0.239 mmol) as the intrenal standard in 120.9 mg sample.

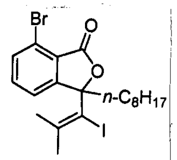

6dg

wxy-3-066-1-purity
2018-11-15 19:25:16.406
USER: nmr
SOLVENT: CDCl3
Experiment = zg30
Pulse length = 14.000 usec
Relaxation delay = 1.000 sec
NA = 8
Solvent = CDCl3
PTS1d = 32768
F1 = 300.130005 MHz
F2 = 1.000000 MHz
SW1 = 6188.12 Hz

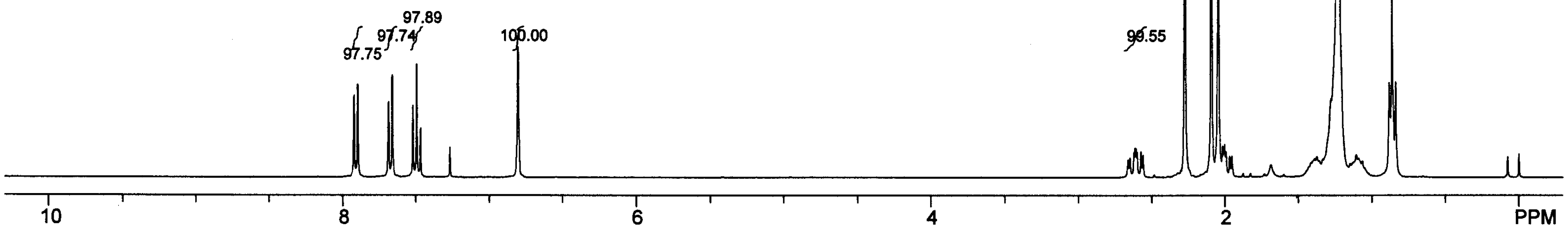

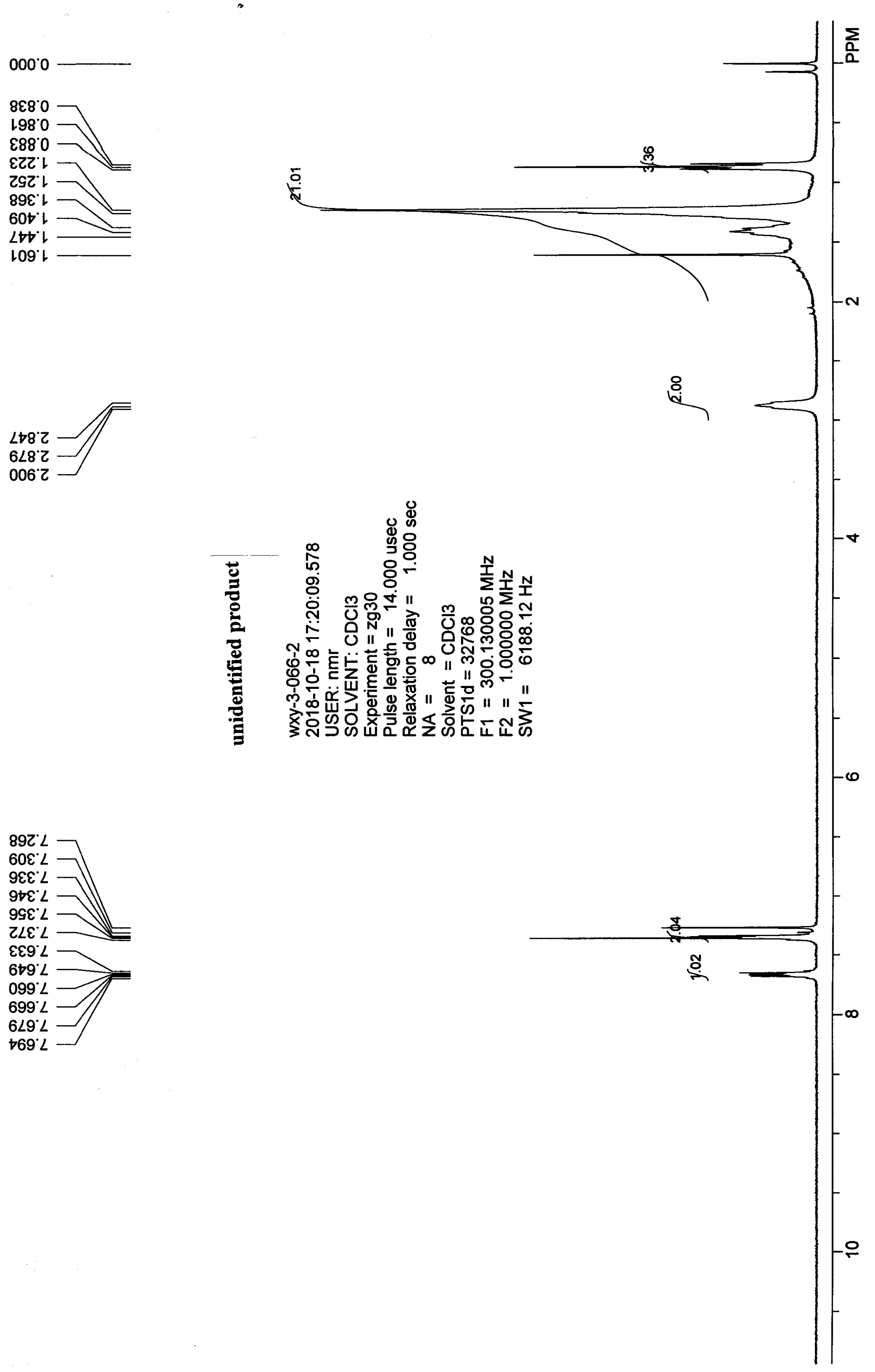
unidentified product
wxy-3-066-2
2018-10-18 17:20:09.578
USER: nmr
SOLVENT: CDCl3
Experiment = zg30
Pulse length = 14.000 usec
Relaxation delay = 1.000 sec
NA = 8
Solvent = CDCl3
PTS1d = 32768
F1 = 300.130005 MHz
F2 = 1.000000 MHz
SW1 = 6188.12 Hz
7.694
7.679
7.669
7.660
7.649
7.633
7.372
7.366
7.346
7.336
7.309
7.268
2.900
2.879
2.847
1.601
1.447
1.409
1.368
1.252
1.223
0.883
0.861
0.838
0.000
1.02
2.04
2.00
21.01
3.36
PPM
10
8
6
4
2

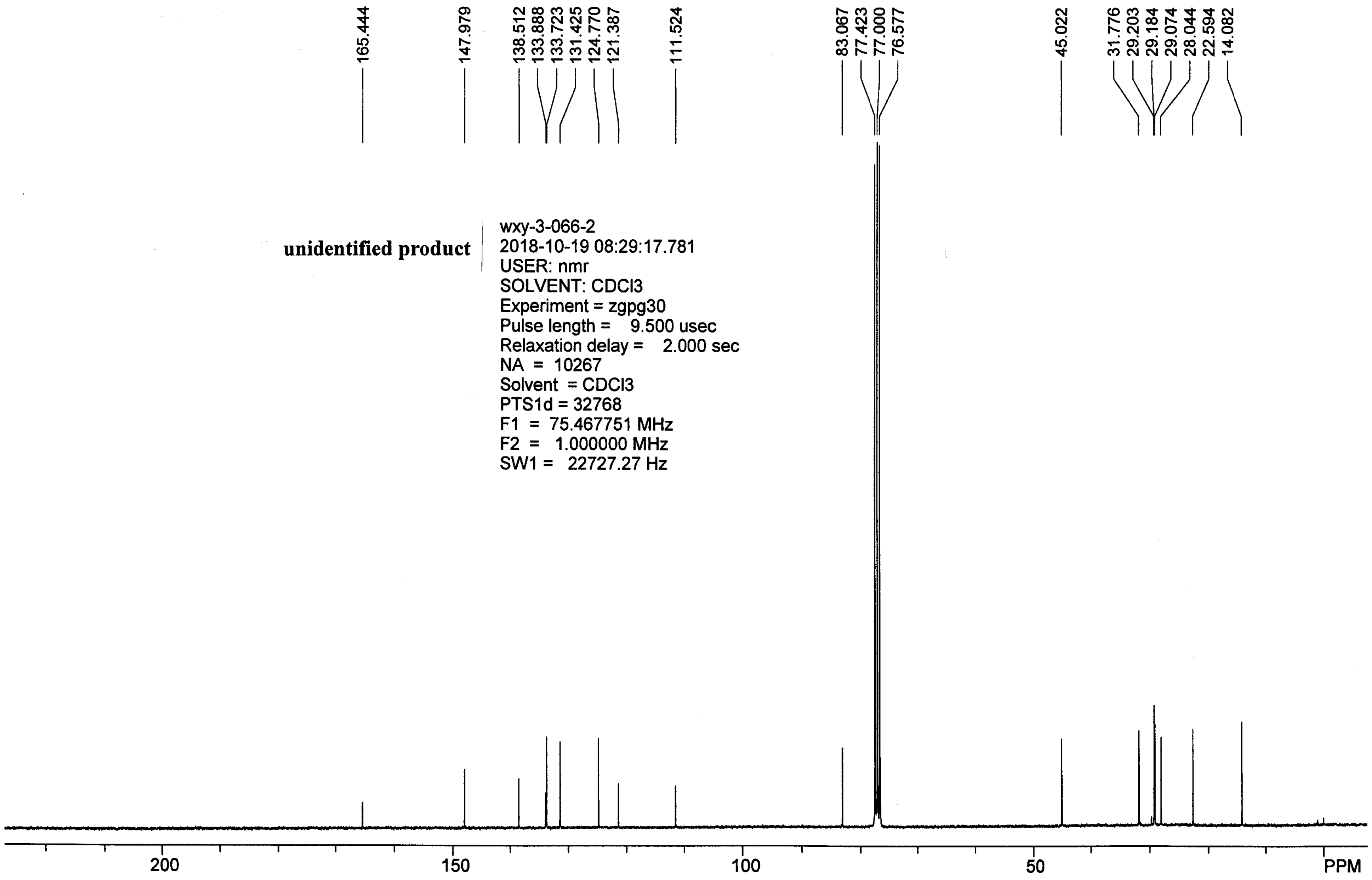
165.444
147.979
138.512
133.888
133.723
131.425
124.770
121.387
111.524
83.067
77.423
77.000
76.577
45.022
31.776
29.203
29.184
29.074
28.044
22.594
14.082
unidentified product
wxy-3-066-2
2018-10-19 08:29:17.781
USER: nmr
SOLVENT: CDCl3
Experiment = zgpg30
Pulse length = 9.500 usec
Relaxation delay = 2.000 sec
NA = 10267
Solvent = CDCl3
PTS1d = 32768
F1 = 75.467751 MHz
F2 = 1.000000 MHz
SW1 = 22727.27 Hz
200
150
100
50
PPM

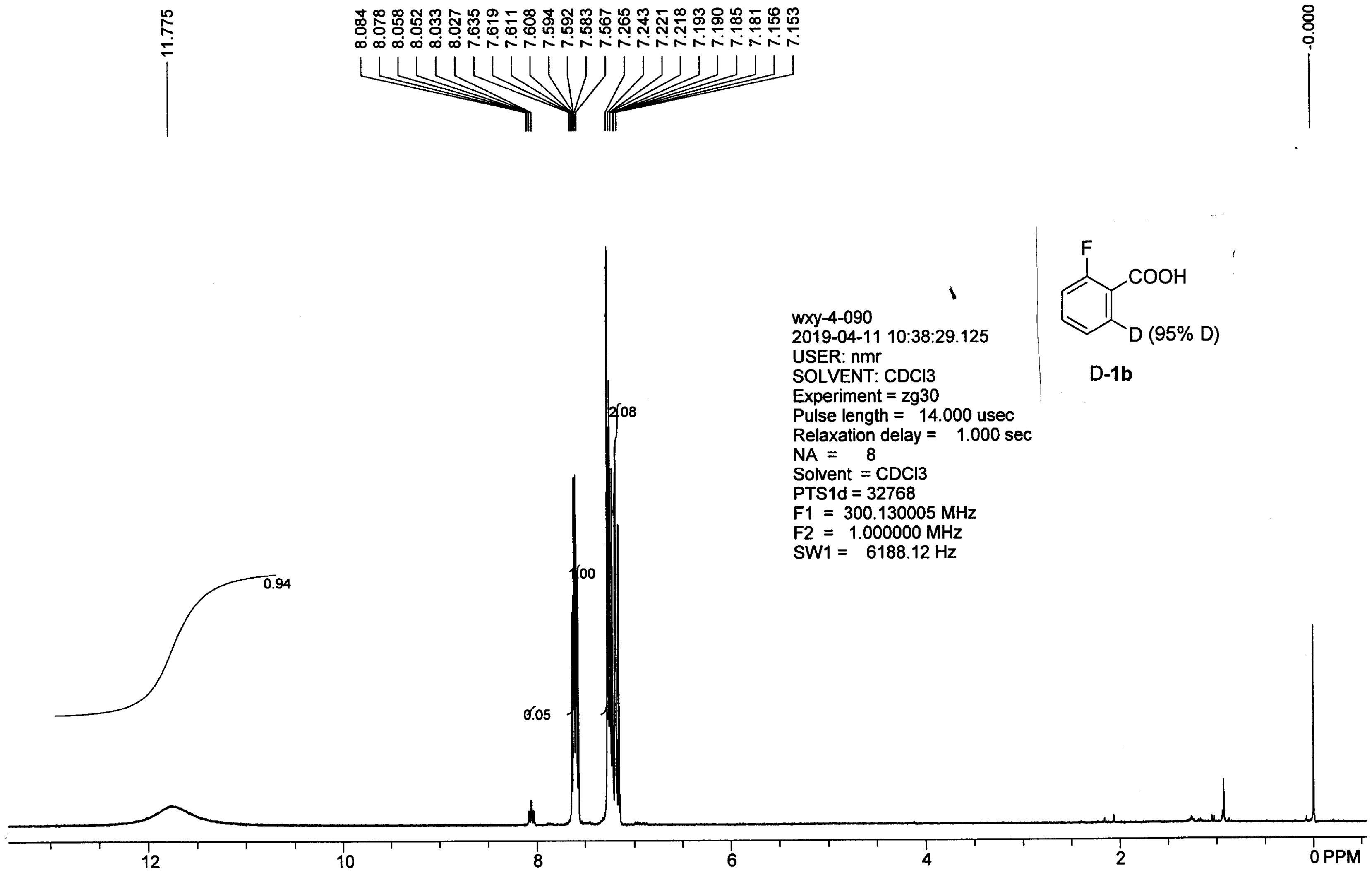
11.775
8.084
8.078
8.058
8.052
8.033
8.027
7.635
7.619
7.611
7.608
7.594
7.592
7.583
7.567
7.265
7.243
7.221
7.218
7.193
7.190
7.185
7.181
7.156
7.153
-0.000
wxy-4-090
2019-04-11 10:38:29.125
USER: nmr
SOLVENT: CDCl3
Experiment = zg30
Pulse length = 14.000 usec
Relaxation delay = 1.000 sec
NA = 8
Solvent = CDCl3
PTS1d = 32768
F1 = 300.130005 MHz
F2 = 1.000000 MHz
SW1 = 6188.12 Hz
0.94
2.08
1.00
0.05
12
10
8
6
4
2
0 PPM
F
COOH
D (95% D)

D-1b

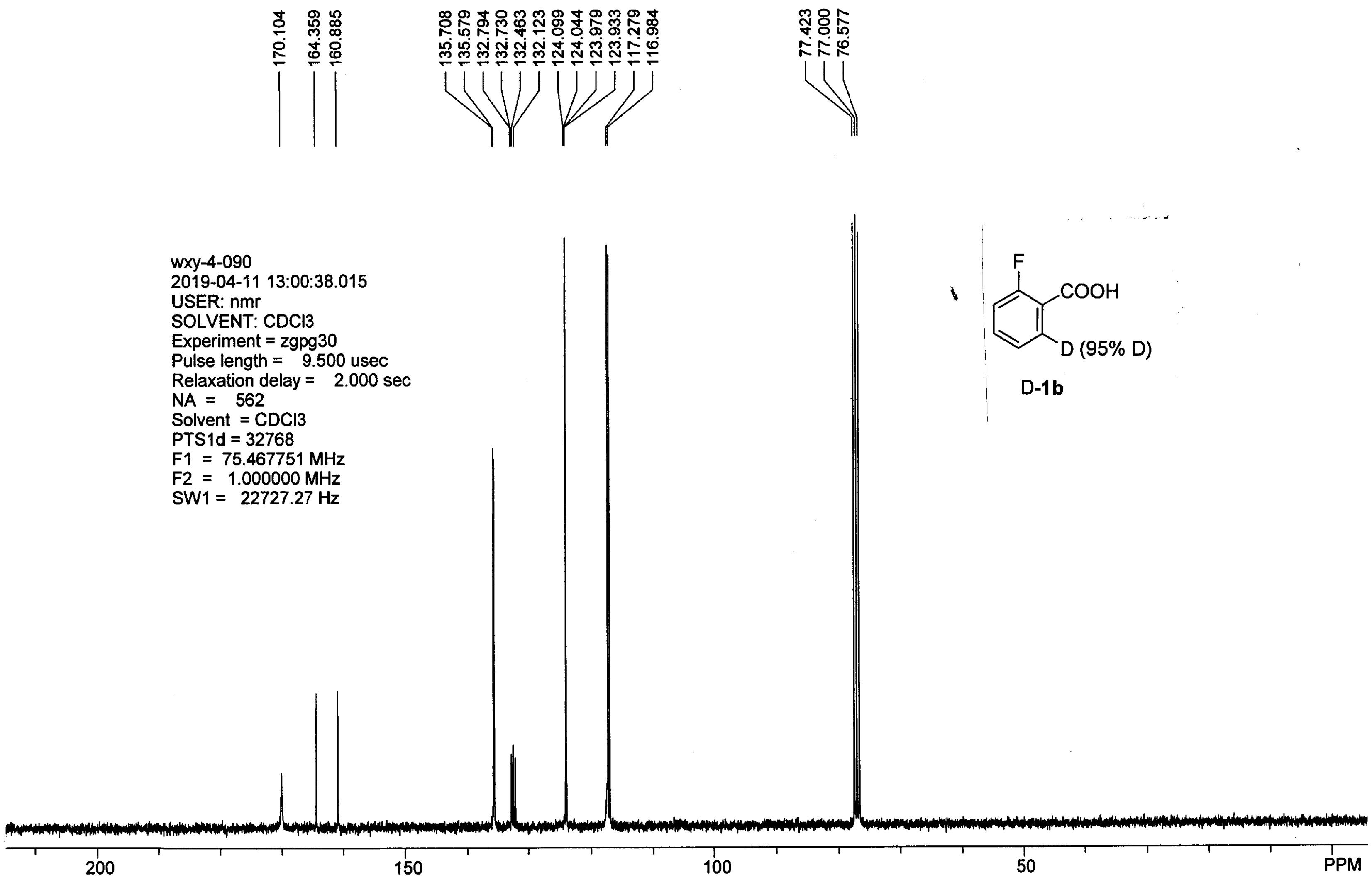
170.104
164.359
160.885
135.708
135.579
132.794
132.730
132.463
132.123
124.099
124.044
123.979
123.933
117.279
116.984
77.423
77.000
76.577
wxy-4-090
2019-04-11 13:00:38.015
USER: nmr
SOLVENT: CDCl3
Experiment = zgpg30
Pulse length = 9.500 usec
Relaxation delay = 2.000 sec
NA = 562
Solvent = CDCl3
PTS1d = 32768
F1 = 75.467751 MHz
F2 = 1.000000 MHz
SW1 = 22727.27 Hz
200
150
100
50
PPM
F
COOH
D (95% D)

D-**1b**

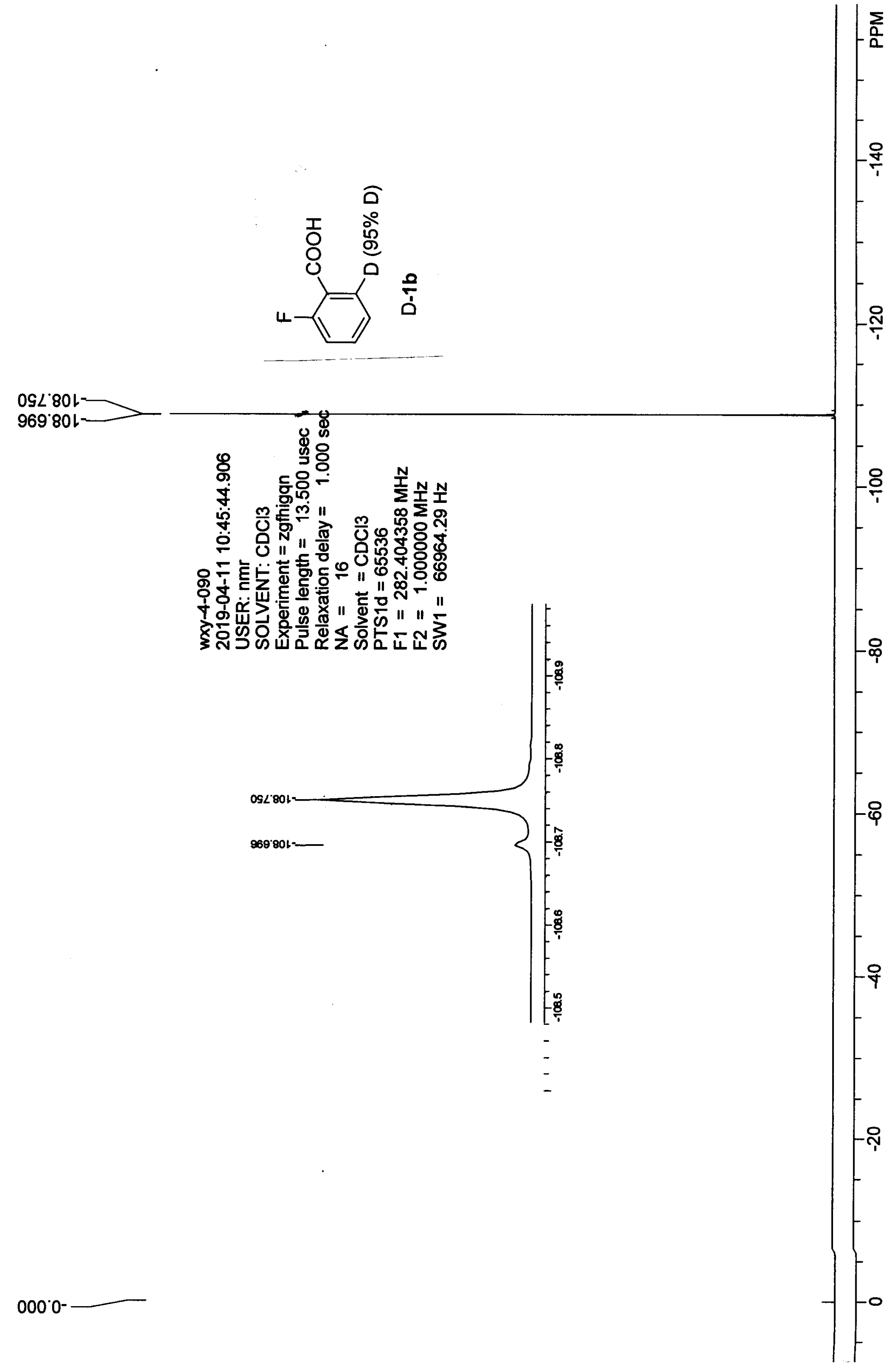

-108.750
-108.696
-0.000
D (95% D)
COOH
F
D-1b
wxy-4-090
2019-04-11 10:45:44.906
USER: nmr
SOLVENT: CDCl3
Experiment = zgfhigqn
Pulse length = 13.500 usec
Relaxation delay = 1.000 sec
NA = 16
Solvent = CDCl3
PTS1d = 65536
F1 = 282.404358 MHz
F2 = 1.000000 MHz
SW1 = 66964.29 Hz
-108.9
-108.8
-108.7
-108.6
-108.5
PPM
-140
-120
-100
-80
-60
-40
-20
0

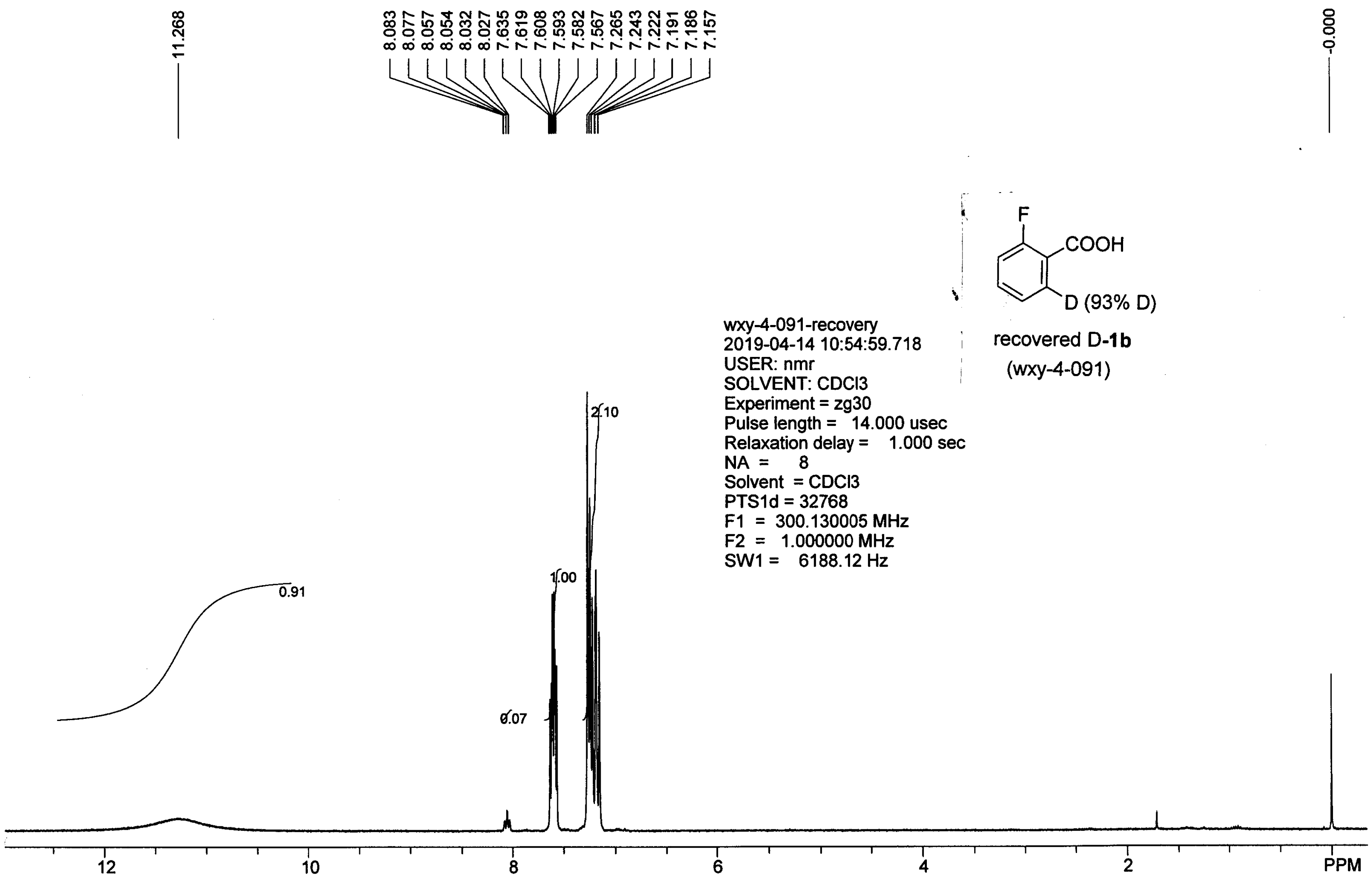
11.268
8.083
8.077
8.057
8.054
8.032
8.027
7.635
7.619
7.608
7.593
7.582
7.567
7.265
7.243
7.222
7.191
7.186
7.157
-0.000
F
COOH
D (93% D)
recovered D-1b
(wxy-4-091)
wxy-4-091-recovery
2019-04-14 10:54:59.718
USER: nmr
SOLVENT: CDCl3
Experiment = zg30
Pulse length = 14.000 usec
Relaxation delay = 1.000 sec
NA = 8
Solvent = CDCl3
PTS1d = 32768
F1 = 300.130005 MHz
F2 = 1.000000 MHz
SW1 = 6188.12 Hz
2.10
1.00
0.07
0.91
12
10
8
6
4
2
PPM

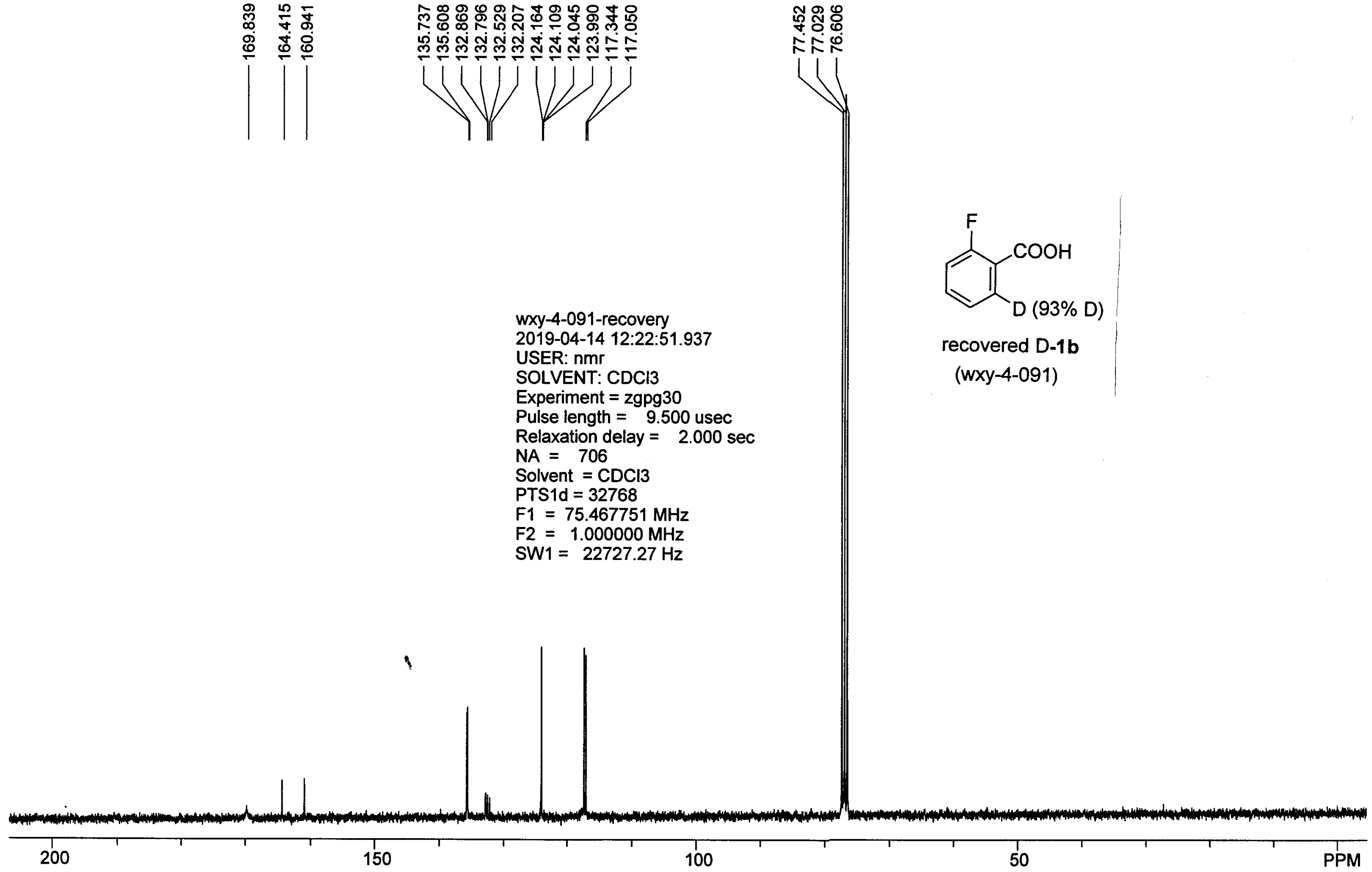
169.839
164.415
160.941
135.737
135.608
132.869
132.796
132.529
132.207
124.164
124.109
124.045
123.990
117.344
117.050
77.452
77.029
76.606
F
COOH
D (93% D)
recovered D-1b
(wxy-4-091)
wxy-4-091-recovery
2019-04-14 12:22:51.937
USER: nmr
SOLVENT: CDCl3
Experiment = zgpg30
Pulse length = 9.500 usec
Relaxation delay = 2.000 sec
NA = 706
Solvent = CDCl3
PTS1d = 32768
F1 = 75.467751 MHz
F2 = 1.000000 MHz
SW1 = 22727.27 Hz
200
150
100
50
PPM

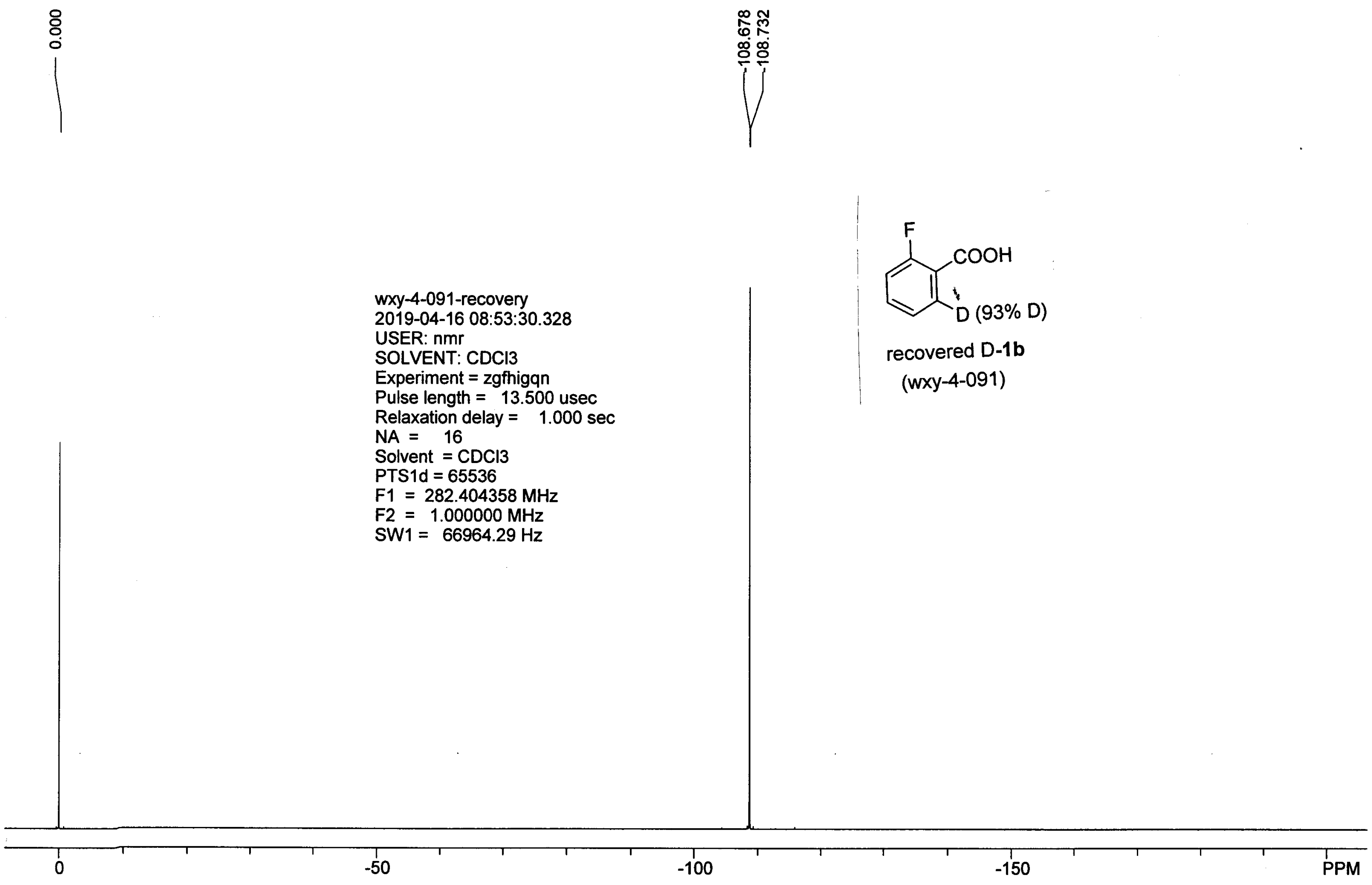
0.000
-108.678
-108.732
wxy-4-091-recovery
2019-04-16 08:53:30.328
USER: nmr
SOLVENT: CDCl3
Experiment = zgfhigqn
Pulse length = 13.500 usec
Relaxation delay = 1.000 sec
NA = 16
Solvent = CDCl3
PTS1d = 65536
F1 = 282.404358 MHz
F2 = 1.000000 MHz
SW1 = 66964.29 Hz
F
COOH
D (93% D)
recovered D-1b
(wxy-4-091)
0
-50
-100
-150
PPM
